# P300/SP1 complex mediating elevated METTL1 regulates CDK14 mRNA stability via internal m7G modification in CRPC

**DOI:** 10.1186/s13046-023-02777-z

**Published:** 2023-08-21

**Authors:** Mingpeng Zhang, Duo Kan, Boya Zhang, Xueqiao Chen, Chun Wang, Songmao Chen, Wenlong Gao, Zhao Yang, Yang Li, Yutong Chen, Shimiao Zhu, Simeng Wen, Yuanjie Niu, Zhiqun Shang

**Affiliations:** 1 Tianjin Institute of Urology, the Second Hospital of Tianjin Medical University, Tianjin, 300211 China; 2 Bone and Soft Tissue Department, The Affiliated Cancer Hospital of Zhengzhou University & Henan Cancer Hospital, Zhengzhou, 450000 China

**Keywords:** METTL1, m7G, P300/SP1 complex, CDK14, CRPC

## Abstract

**Background:**

N7-methylguanosine (m7G) modification is, a more common epigenetic modification in addition to m6A modification, mainly found in mRNA capsids, mRNA interiors, transfer RNA (tRNA), pri-miRNA, and ribosomal RNA (rRNA). It has been found that m7G modifications play an important role in mRNA transcription, tRNA stability, rRNA processing maturation, and miRNA biosynthesis. However, the role of m7G modifications within mRNA and its “writer” methyltransferase 1(METTL1) in tumors, particularly prostate cancer (PCa), has not been revealed.

**Methods:**

The differential expression level of METTL1 between hormone-sensitive prostate cancer (HSPC) and castrate-resistant prostate cancer (CRPC) was evaluated via RNA-seq and in vitro experiments. The effects of METTL1 on CRPC progression were investigated through in vitro and in vivo assays. The upstream molecular mechanism of METTL1 expression upregulation and the downstream mechanism of its action were explored via Chromatin Immunoprecipitation quantitative reverse transcription polymerase chain reaction (CHIP-qPCR), Co-immunoprecipitation (Co-IP), luciferase reporter assay, transcriptome-sequencing, m7G AlkAniline-Seq, and mRNA degradation experiments, etc.

**Results and conclusion:**

Here, we found that METTL1 was elevated in CRPC and that patients with METTL1 elevation tended to have a poor prognosis. Functionally, the knockdown of METTL1 in CRPC cells significantly limited cell proliferation and invasive capacity. Mechanistically, we unveiled that P300 can form a complex with SP1 and bind to the promoter region of the METTL1 gene via SP1, thereby mediating METTL1 transcriptional upregulation in CRPC. Subsequently, our findings indicated that METTL1 leads to enhanced mRNA stability of CDK14 by adding m7G modifications inside its mRNA, ultimately promoting CRPC progression.

**Supplementary Information:**

The online version contains supplementary material available at 10.1186/s13046-023-02777-z.

## Background

Prostate cancer (PCa) is the most common cancer in men, accounting for the second-highest number of male cancer deaths worldwide. The mainstay of treatment for PCa remains endocrine therapy. The most common form of endocrine therapy is androgen deprivation (ADT), which initially benefits patients with HSPC, but most patients inevitably progress to CRPC, which remains a difficult treatment option for clinicians due to the complexity and heterogeneity of the disease.

Epigenetics is the discipline of causing genetically modifiable changes in gene expression without altering the DNA sequence. More than 170 types of epigenetic modifications have been identified on RNA, including N6-methyladenosine (m6A), N1-methyladenosine (m1A), 5-methylcytosine (m5C), and N7-methylguanosine (m7G), which account for more than half of all epigenetic modifications [1]. As a more common form of epigenetic modification, m7G was found to be present in nearly all types of RNA molecules, including mRNA 5’ cap structures, mRNA internal, pri-miRNA, transfer RNA (tRNA), and ribosomal RNA (rRNA), and such modifications play an important role in mRNA transcription, miRNA biosynthesis, tRNA stability, intranuclear processing and maturation of 18 S rRNA, etc [2–4]. We know that epigenetic modification is a reversible modification process mediated by different enzymes. It has been shown that m7G modification of mammalian RNA is mediated by METTL1. For several years, researchers have revealed that METTL1 is aberrantly expressed in various solid tumors and regulates tumorigenesis and progression in an m7G-dependent manner [5–8]. However, the role and function of m7G modifications within mRNAs in PCa, particularly CRPC, remains unexplored.

P300 is an acetyltransferase that on one hand is able to participate in the regulation of gene expression by acetylating histones and non-histones; on the other hand, P300 acts as a transcriptional co-activator and acts as a scaffold/bridge between transcription factors and the basic transcriptional complex [9, 10]. SP1 is a very critical transcription factor in mammals. It is involved in the progression of many tumors, including PCa. Studies have shown that SP1 is involved in the regulation of transcription and the formation of the histone acetyltransferase complex is inextricably linked. It has been found that in eukaryotes, SP1 can recruit histone acetylases and deacetylases to activate or repress the expression of target genes via dynamically regulating the acetylation transitions of histones [11–13]. In PCa, especially in CRPC, the involvement of SP1 and P300 complexes in the gene transcription regulation is still rarely reported and needs to be further revealed.

Members of the cell cycle protein-dependent kinase family include transcription-associated CDKs, cell cycle-associated CDKs, and non-classical CDKs. The function of CDK14, a member of the non-classical CDKs, is gradually being uncovered [14]. In triple-negative breast cancer, the CDK14/CCNY complex phosphorylates the membrane receptor LRP6 and regulates the activity of breast cancer stem cells through the Wnt/β-catenin signaling pathway, ultimately promoting the progression and metastasis of triple-negative breast cancer [15]. In esophageal squamous cell carcinoma, CDK14 can be used as one of the predictors of tumor prognosis and chemotherapy sensitivity [16]. In addition, in solid tumors such as non-small cell lung cancer, colorectal cancer, gastric cancer, etc., CDK14 often plays a tumor-promoting role as an oncogenic factor and is associated with poor patient prognosis [17–20]. However, in PCa, the biological functions and significance of CDK14 have not been illuminated.

In our study, we combined bioinformatic predictions and cellular experiments to confirm that SP1 forms a complex with P300 and binds to the promoter region of METTL1, and promotes METTL1 transcription in CRPC. Finally, RNA degradation assays and rescue experiments concluded that METTL1 makes CDK14 mRNA more stable by adding m7G modifications to its interior and ultimately facilitates CRPC progression.

## Materials and methods

### Clinical specimens

#### Informed consent

was obtained from patients at the Second Hospital of Tianjin Medical University (Tianjin, China) prior to collection of all clinical samples. Inclusion criteria: Standard-weight male patients over 50 with a confirmed diagnosis of HSPC and CRPC. Exclusion criteria: Combination of other potentially life-threatening illnesses in the short term. Ethical consent was approved by the Ethics Committee of the Second Hospital of Tianjin Medical University.

### Cell culture and transfection

HSPC cells and CRPC cell lines except LNCaP-AI were cultured in RPMI-1640 medium containing 10% fetal bovine serum (Gibco, Waltham, MA, USA). LNCaP-AI cells were cultured in RPMI-1640 medium supplemented with 10% fetal bovine serum under androgen-deprived conditions. Transfection of LNCaP-AI and C4-2 cells reaching 60% confluency with siRNAs, shRNA, or plasmids was carried out using Lipofectamine 3000 (Invitrogen, Waltham, MA, USA) or X-tremeGENE HP Transfection Reagent (Roche, Indianapolis, IN, USA), respectively, according to the manufacturer’s instructions. Target sequences for siRNAs, shRNA, and overexpression plasmids sequences involved in this study are listed in Supplementary Table 1.

### Tissue microarray (TMA) and immunohistochemistry (IHC)

TMA designed and manufactured by Shanghai Outdo Biotech Company (Cat No. T17–1413 TMA) in this work contains HSPC (n = 31) and CRPC (n = 18) clinical samples. The tissue microarrays were then subjected to immunohistochemical staining experiments according to detailed procedures as described previous [34]. The staining results are scored according to the shade and extent of positive cell staining: 0 for negative, 1 for weak positive, 2 for positive, and 3 for strong positive.

### Western blot

Firstly, the protein lysate RIPA (Solarbio, R0010, China) and protease inhibitor were mixed in a 100:1 ratio and the configured mixture was used to lyse the cells to extract the proteins. Subsequently, the protein concentration was measured using Bradford (Solarbio, PC0010, China) at 595 nm on an enzymatic calibrator. 30 µg protein samples were added to the SDS-PAGE gel well and electrophoresis was performed at a constant voltage of 90 volts. At the end of electrophoresis, electrotransfer operations were carried out at a constant current of 250 mA for 2 h. After electrotransfer, the PVDF membrane (Millipore, ISEQ00010, USA) was blocked for 1 h on a shaker at room temperature using 5% skimmed milk. PVDF membranes were then incubated overnight at 4℃ using a specific primary antibody. The next day, secondary antibodies cross-linked with HRP were used to incubate PVDF membranes for 1 h at room temperature. Finally, capture the image for visualization using an electrochemiluminescence imaging system. Antibodies and drugs involved in this study and dilution ratio are listed in Supplementary Tables 2 and Table 3.

### Real-time quantitative polymerase chain reaction (RT-qPCR)

Trizol reagent (Invitrogen, USA) was utilized to extract total RNA from the cells. After detecting the concentration, the total RNA was then transformed into cDNA using a reverse transcription kit (Thermo Scientific, USA) following the operating instructions. Finally, a 10uL reaction system consisting of 5uL SYBR Green PCR Master Mix (Roche), 3uL enzyme-free water, 1uL primers, and 1uL cDNA was used for RT-qPCR. GAPDH was used as an interior control. After the experiment is finished, the multiplicity of gene changes is calculated via the formula 2^−ΔΔCt^. All primers related to this work are listed in Supplementary Table 4.

### MTT and colony formation assay

MTT and colony formation assays are used to detect the proliferative capacity of the cells. Cells in good growth transitions were first made into cell suspensions and inoculated in 96-well plates and 6-well plates at a certain density. For MTT assays, cells were incubated with 5 mg/mL MTT solution (Solarbio, M8180, China) for 2 h on days 1, 2, and 3, respectively, and the absorbance values of the cells were measured at 490 nm and cell growth curves were plotted. For colony formation, after 2 weeks of culture, cells were sequentially fixed with 4% paraformaldehyde, stained with crystal violet, and photographs were captured to calculate the number of clonal colonies.

### Transwell assay

Transwell experiment was performed to evaluate the invasion capacity of cells. Briefly, cells were first seeded in transwell chambers for three days. Subsequently, cells were fixed using a pre-cooled cell fixative. Then, a crystal violet working solution was applied to stain the cells. Finally, images were captured under a light microscope and subjected to statistical analysis.

### RNA m7G dot blot

RNA was extracted and concentrations were determined. The RNA was then diluted to 50 ng/uL, 100 ng/uL, 200 ng/uL, and 400 ng/uL. The RNA was denatured by heating at 95 °C for 5 min and 2 uL of each of these concentrations was then dropped onto a nitrocellulose membrane (Amersham, GE Healthcare, USA). Subsequently, the membranes were cross-linked using a UV crosslinker, blocked with 5% skimmed milk, incubated with m7G antibody overnight at 4 °C, and incubated with HRP-conjugated secondary antibody. Finally, the chemiluminescence system was utilized to visualize the membrane. The membrane stained with methylene blue (MB) was used as a control.

### Co-immunoprecipitations assay (Co-IP)

Co-immunoprecipitations assay was carried out in LNCaP-AI and C4-2 cells with Pierce Classic Magnetic IP/Co-IP Kit (ThermoFisher Scientific, USA) according to the operating instructions. In short, cold IP lysis was used to lysis the cells. 500 µg of protein was obtained according to the protein extraction procedure and fixed to 500 uL using IP lysate. 5 µg of SP1 or P300 antibody was used to incubate the protein to form an antigen-antibody complex. Protein A/G magnetic beads and antigen-antibody complexes were then mixed and incubated at room temperature for 1 h. After sufficient washing, the antigen-antibody complexes were eluted using an elution buffer. Finally, products after elution were subjected to western blot to detect P300 or SP1 protein.

### Chromatin immunoprecipitation assay (CHIP)

The CHIP assay was performed using CHIP Kit (ThermoFisher Scientific, USA) according to the operating manual. Briefly, 1% formaldehyde was used to crosslink the protein and DNA; After cell lysis, chromatin was digested into small pieces with micrococcal nuclease; 5ug antibody was used to immunoprecipitated the DNA-protein complex; NaCl and proteinase K treatment to undo DNA-protein crosslinking; DNA purification and recovery; Finally, the obtained DNA was subjected to RT-qPCR for further analysis. Fold enrichment was calculated after normalizing to 1% input.

### Luciferase reporter assay

Cos7 cells were co-transfected with METTL1 3′-UTR mild report plasmid and SP1 or/and P300 overexpression plasmids using X-tremeGENE HP DNA (Roche, USA) following the manufacturer’s instruction. After 24 h of culture in the incubator, renilla and firefly luciferase activities were determined using a luciferase reporter assay system (Promega, Massachusetts, USA).

### m7G RNA immunoprecipitation assay (MeRIP)

m7G RNA immunoprecipitation assay (MeRIP) experimental methods and detailed procedures were carried out as described previously [35]. Magna RIP Kit (Millipore, USA) was utilized to perform the MeRIP experiment. It should be noted that the LNCaP-AI and C4-2 cells in this assay are 1.0 × 10^7^. 10ug m7G antibody was used to gather the m7G modified mRNA. Finally, the obtained mRNA was subjected to RT-qPCR. Fold enrichment was calculated after normalizing to 1% input.

### mRNA stability

Transcriptional inhibitor actinomycin D (MCE, HY-17,559, USA) was used to inhibit RNA synthesis. After treatment with actinomycin D, LNCaP-AI and C4-2 cells were harvested at 2, 4, 6, and 8 h time points, and the RNA was then extracted. The remaining mRNA of CDK14 was detected through RT-qPCR.

### In vivo experiment

The immunodeficient nude mice involved in this work were purchased from HFK Bioscience Co. Ltd (Beijing, China) and the experimental process was approved by the Ethics Committee of the Second Hospital of Tianjin Medical University (Tianjin, China). In short, 5 × 10^6^ C4-2 cells were subcutaneously implanted to form subcutaneous transplant tumors in nude mice. Nude mice were divided into 4 groups based on the application of the P300 agonist CTPB and adeno-associated virus (AAV) targeting METTL1. CTPB was mainly given via oral feeding at 10 mg/kg in 1% carboxymethyl cellulose, 0.1% Tween-80, and 5% DMSO. The tumor volume was monitored every other day with the formula V = 1/2 × length × width^2^. After in vivo assay was finished, nude mice were euthanized and subcutaneous graft tumors were removed and embedded with paraffin, and subjected to immunohistochemistry analysis.

### Statistical analysis

Data in this work were analyzed using GraphPad Prism 8 software and presented as mean ± S.D. All data were obtained after three biological replicates. The difference between the two groups was analyzed through Student’s t-test. Log-rank P value was used in survival analysis. P value < 0.05 was considered to indicate a statistically significant difference. *P < 0.05; **P < 0.01; ***P < 0.001; ****P < 0.0001.

## Results

### Elevated METTL1 expression correlates with poor prognosis of patients with CRPC

**Fig. 1:**
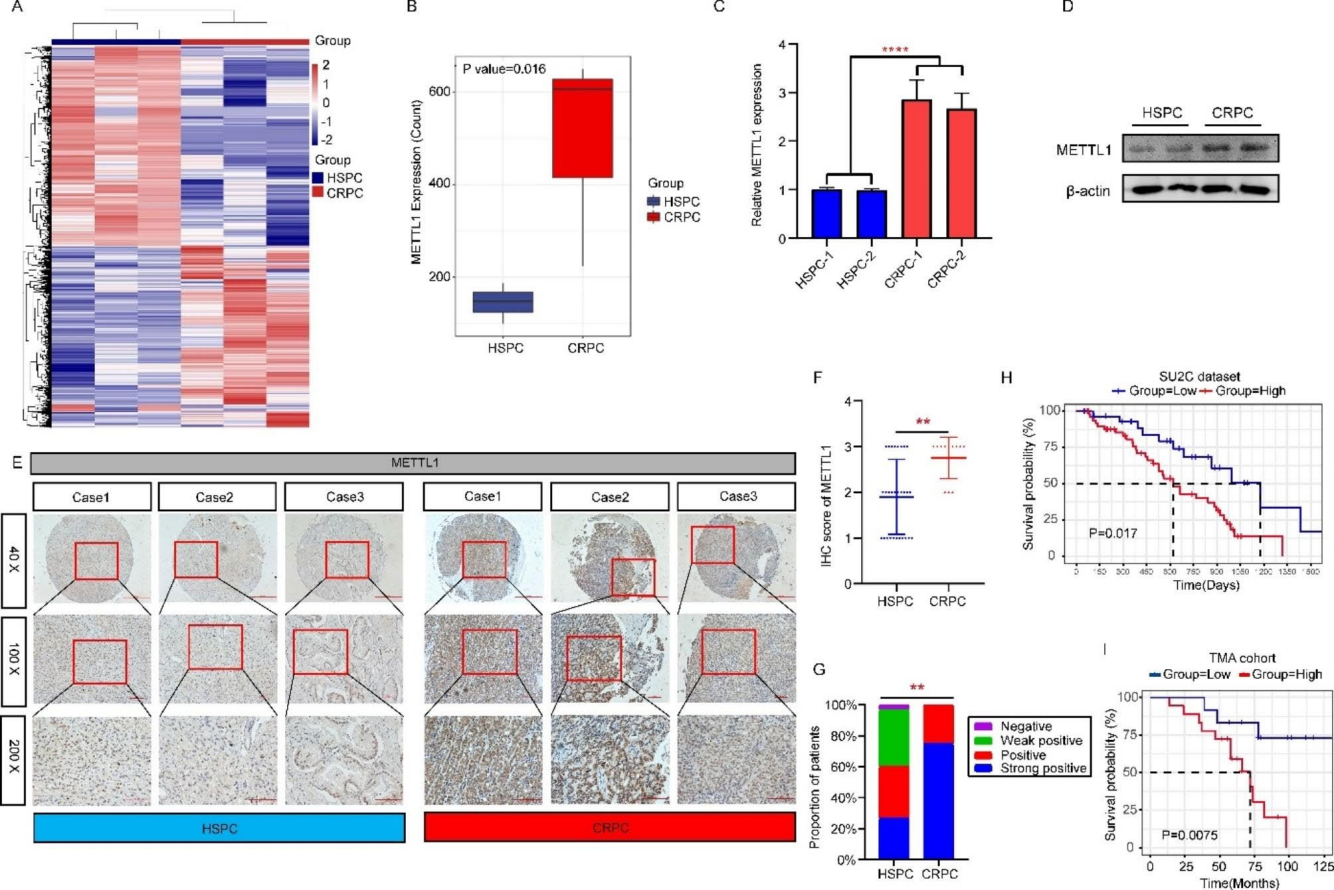
Elevated METTL1 expression correlates with poor prognosis of patients with CRPC. **A** Heat map of transcriptome sequencing indicates differentially expressed genes between HSPC and CRPC patient tissues. The screening thresholds:|Fold change|>2 and p-value < 0.05. **B** Differential expression of METTL1 between HSPC and CRPC tissue via transcriptome sequencing. **C-D** RT-qPCR and western blot were performed to detect the mRNA and protein level of METTL1 between HSPC and CRPC patients’ samples. **E** Immunohistochemistry (IHC) assay was carried out to determine the METTL1 expression on a tissue microarray (TMA). Objective magnification: 40X, 100X, and 200X. **F-G** Immunohistochemical staining scores of METTL1 for each HSPC and CRPC patient in tissue microarrays (F) and the proportion of patients with different levels of METTL1 staining (G). **H-I** Disease-free survival of patients in SU2C database and TMA using Kaplan–Meier survival analysis. **P < 0.01; ****P < 0.0001

We performed whole transcriptome sequencing on tumor tissue samples from three CRPC and three HSPC patients. The differentially expressed genes (DEGs) were screened according to the criteria of |Fold change| > 2 and p-value < 0.05, and the DEGs were displayed in a heat map (Fig. 1A, Supplementary Table 5). Based on the results of differential genes, we found that METTL1 expression was abnormally elevated in CRPC compared to HSPC (Fig. 1B). To verify the accuracy of the sequencing results, we obtained tumor tissues and extracted mRNA and protein from HSPC and CRPC patients, and examined the differential expression of METTL1 at the mRNA and protein levels, respectively. The results showed that METTL1 was expressed at a higher level in CRPC patients compared to HSPC patients (Fig. 1C and D). Subsequently, we carried out immunohistochemical staining experiments on tissue microarrays containing 18 CRPC patients and 31 HSPC patients and quantified the staining results, and found that the overall expression level of METTL1 was higher in CRPC (Fig. 1E and F). The proportion of patients with “positive” and “strong positive” immunohistochemical staining was significantly higher in CRPC than in HSPC patients (Fig. 1G). Finally, we divided CRPC patients into high and low METTL1 expression groups based on the METTL1 mRNA expression values in the SU2C database and discovered that CRPC patients with high METTL1 expression had worse survival when combined with patient survival data (Fig. 1H). Similarly, we classified patients with “positive” and “strong positive” METTL1 staining results as the high expression group based on tissue microarray immunohistochemical staining, and CRPC patients with “negative” and “weak positive” staining results were defined as the low METTL1 expression group. Based on patients’ survival transitions and survival times, we also found that patients in the high METTL1 expression group had worse survival and prognosis (Fig. 1I), which was consistent with the SU2C database results. Taken together, we reach a decision that METTL1 expression is abnormally upregulated in CRPC and patients with METTL1 high expression have worse survival.

### Knockdown of METTL1 inhibits CRPC cell proliferation and invasion

**Fig. 2:**
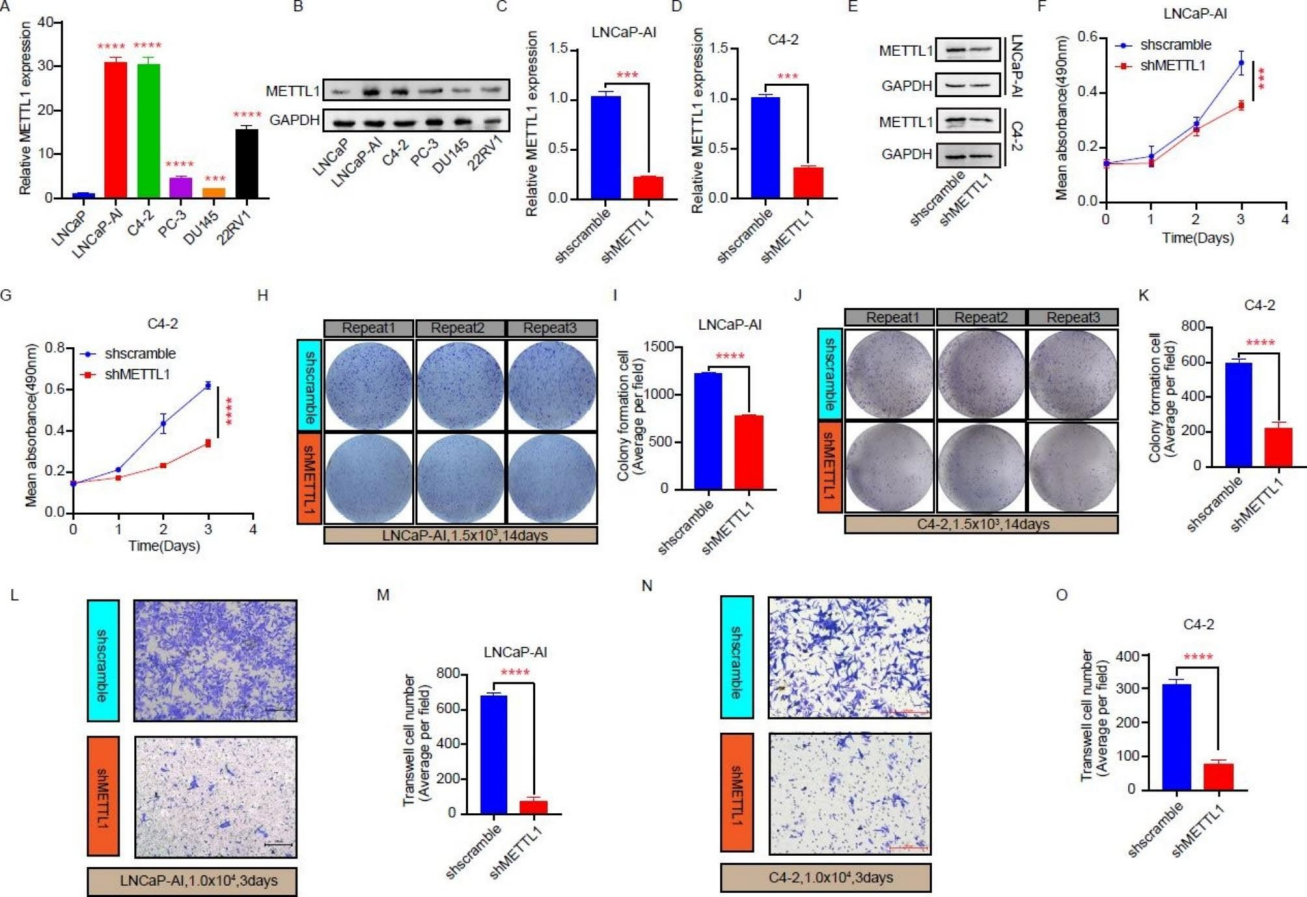
Knockdown of METTL1 inhibits CRPC cell proliferation and invasion. **A-B** Detection of METTL1 mRNA and protein level between the HSPC and CRPC cell lines. **C-E** METTL1 knockdown efficiency in LNCaP-AI and C4-2 cells via RT-qPCR and western blot. **F-K** Validation of proliferation ability change after METTL1 was inhibited in LNCaP-AI and C4-2 cells through MTT (F, G) and colony formation (H-K) assays. **L-O** Transwell was performed to determine the invasion capacity variation of LNCaP-AI and C4-2 cells caused by METTL1 suppression. Three biological replicates of each experiment were performed. ***P < 0.001; ****P < 0.0001

To elucidate the effect of METTL1 on the malignant biological behavior of CRPC, we implemented phenotypic experiments in prostate cancer cell lines. We first detected the differences in METTL1 level between HSPC and CRPC cell lines and found that METTL1 was differentially elevated in CRPC cell lines compared to the HSPC cell line LNCaP, with LNCaP-AI and C4-2 being the most prominent (Fig. 2A and B). Subsequently, we used LNCaP-AI and C4-2 cells as the study vectors to construct cell lines with stable silencing of METTL1 (Fig. 2C–E). To investigate the effect of METTL1 on the proliferative capacity of CRPC cells, we performed MTT assays and clone formation assays. The results implied that the knockdown of METTL1 reduced the proliferation viability of the cells, decreased the number of clonal colony formations, and significantly inhibited the proliferation ability (Fig. 2F–K). Finally, we carried out transwell assays in LNCaP-AI cells and C4-2 cells and found that inhibition of METTL1 expression suppressed the invasive ability of the cells (Fig. 2L–O). In summary, we tentatively conclude that METTL1 expression is associated with the poor biological behavior of CRPC cells. Impose restrictions on METTL1 expression significantly limited both the proliferation and invasive ability of the cells.

### Increased P300 mediates the high expression of METTL1 via H3K27 acetylation modification

**Fig. 3:**
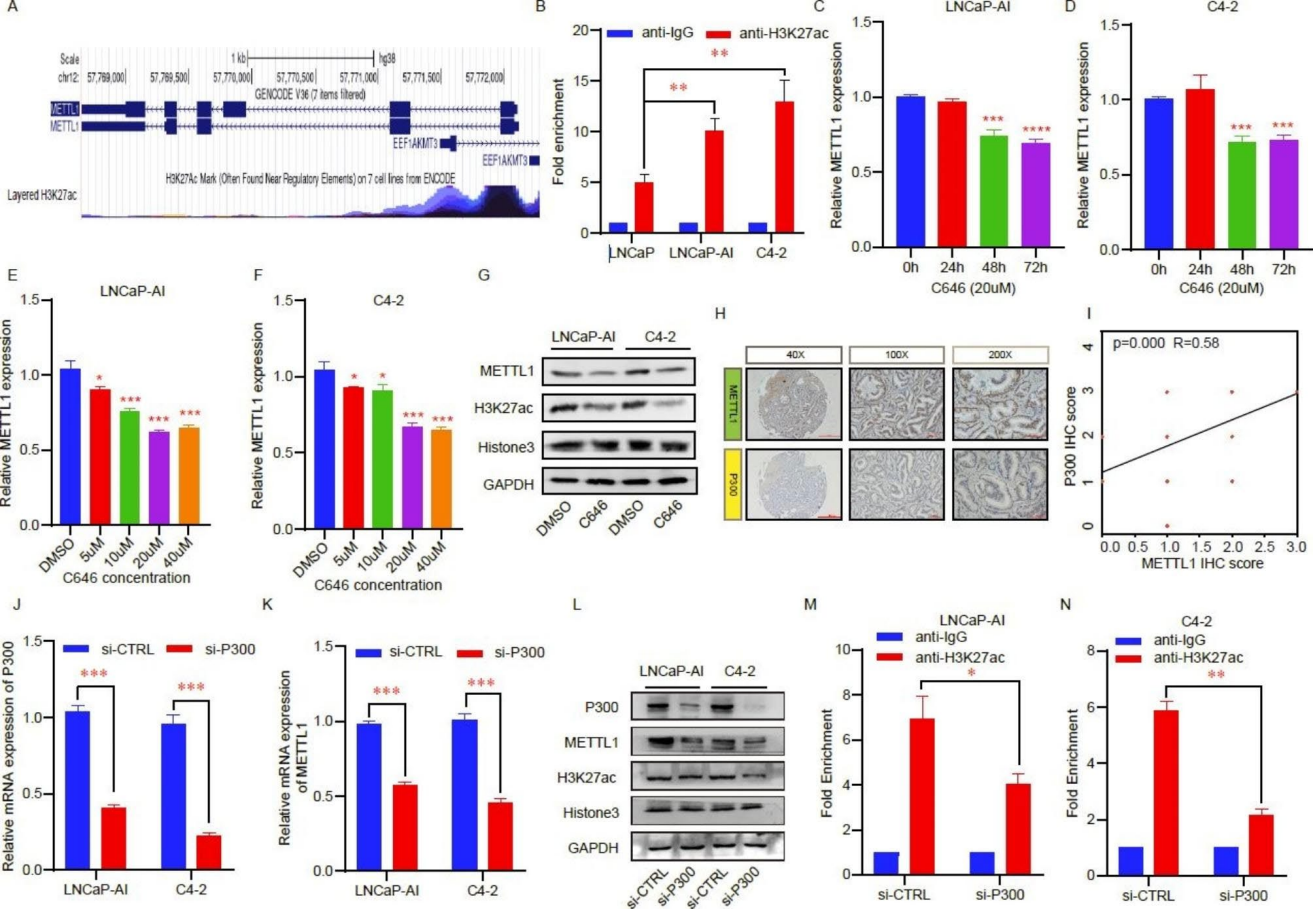
Increased P300 mediates the high expression of METTL1 via H3K27 acetylation modification. **A** Data from the UCSC genome bioinformatics site indicated high H3K27ac enrichment in the promoter of METTL1. **B** CHIP-qPCR was used to confirm the difference in the H3K17ac-modified METTL1 promoter region between HSPC and CRPC cells. **C-D** The mRNA levels of METTL1 in C646 (20 µM)-treated LNCaP-AI (C), and C4-2 (D) cells at the indicated time points were measured by RT-qPCR. **E-F** The mRNA levels of METTL1 in different concentrations of C646 (48 h)-treated LNCaP-AI (E), and C4-2 (F) cells were measured by RT-qPCR. **G** The METTL1 and H3K27ac protein levels were measured via western blot assay after C646 treatment (20 µM) for 48 h. **H** IHC was carried out to detect the P300 level in TMA containing HSPC and CRPC patients’ samples. **I** Pearson correlation between METTL1 and P300 (R = 0.58). **J-K** The mRNA change of METTL1 after P300 was inhibited using small interfering RNA (siRNA). **L** The P300, METTL1, and H3K27ac protein levels were measured via western blot assay after P300 suppression. **M-N** CHIP-qPCR was used to determine the enrichment of H3K27ac at the promoter of METTL1 in control or P300 deficiency LNCaP-AI and C4-2 cells. *P < 0.05; **P < 0.01; ***P < 0.001. H3K27ac, H3K27 acetylation; CHIP, chromatin immunoprecipitation

To explore the potential molecular mechanisms underlying the high METTL1 expression in CRPC, we first visualized the modification form in the promoter of the METTL1 gene via the UCSC online website (http://genome.ucsc.edu/). We found abundant histone acetylation modification peaks (H3K27ac) in the promoter region of the METTL1 gene, suggesting that METTL1 might be modulated via chromatin acetylation (Fig. 3A). Next, we performed chromatin immunoprecipitation experiments with H3K27ac antibody and found that the abundance of H3K27ac modification in the METTL1 promoter region was higher in LNCaP-AI and C4-2 cells than in LNCaP cells (Fig. 3B). This suggested that the abnormal upregulation of METTL1 levels in CRPC may be related to the high H3K27ac modification in the promoter region of the METTL1 gene. Given that P300 is a key enzyme mediating H3K27 acetylation modification, we treated LNCaP-AI and C4-2 cells with the P300 inhibitor C646 and found that a significant decrease in METTL1 mRNA expression was seen after 48 h when cells were treated with a 20 μM concentration of C646 (Fig. 3C and D). Immediately following, we treated cells with different concentration gradients of C646, and at the 48 h time point, we discovered that the most distinct reduction in METTL1 mRNA levels was seen when the C646 concentration was 20 μM (Fig. 3E and F). Subsequently, we also confirmed at the protein level a decline in both histone H3K27ac and METTL1 following the treatment of the P300 inhibitor C646 (20 μM,48 h) (Fig. 3G). Next, we performed a staining analysis using tissue microarrays and found a positive correlation between P300 and METTL1 (R = 0.58) (Fig. 3H and I). In addition, we designed siRNA to inhibit P300 expression (Fig. 3J) and confirmed that the knockdown of P300 could inhibit METTL1 expression (Fig. 3K and L). To further demonstrate that the reduction in METTL1 mRNA levels due to the knockdown of P300 was caused by reduced H3K27ac level in the METTL1 promoter region, we performed chromatin immunoprecipitation experiments. The results indicated that suppression of P300 also significantly reduced the level of H3K27ac in the promoter region of the METTL1 gene (Fig. 3M and N). The above experimental results provide preliminary evidence that the P300-mediated modification of H3K27ac in the promoter region of METTL1 is an important mechanism leading to the upregulation of METTL1 mRNA in CRPC.

### The P300/SP1 complex regulates the transcriptional activity of METTL1

**Fig. 4:**
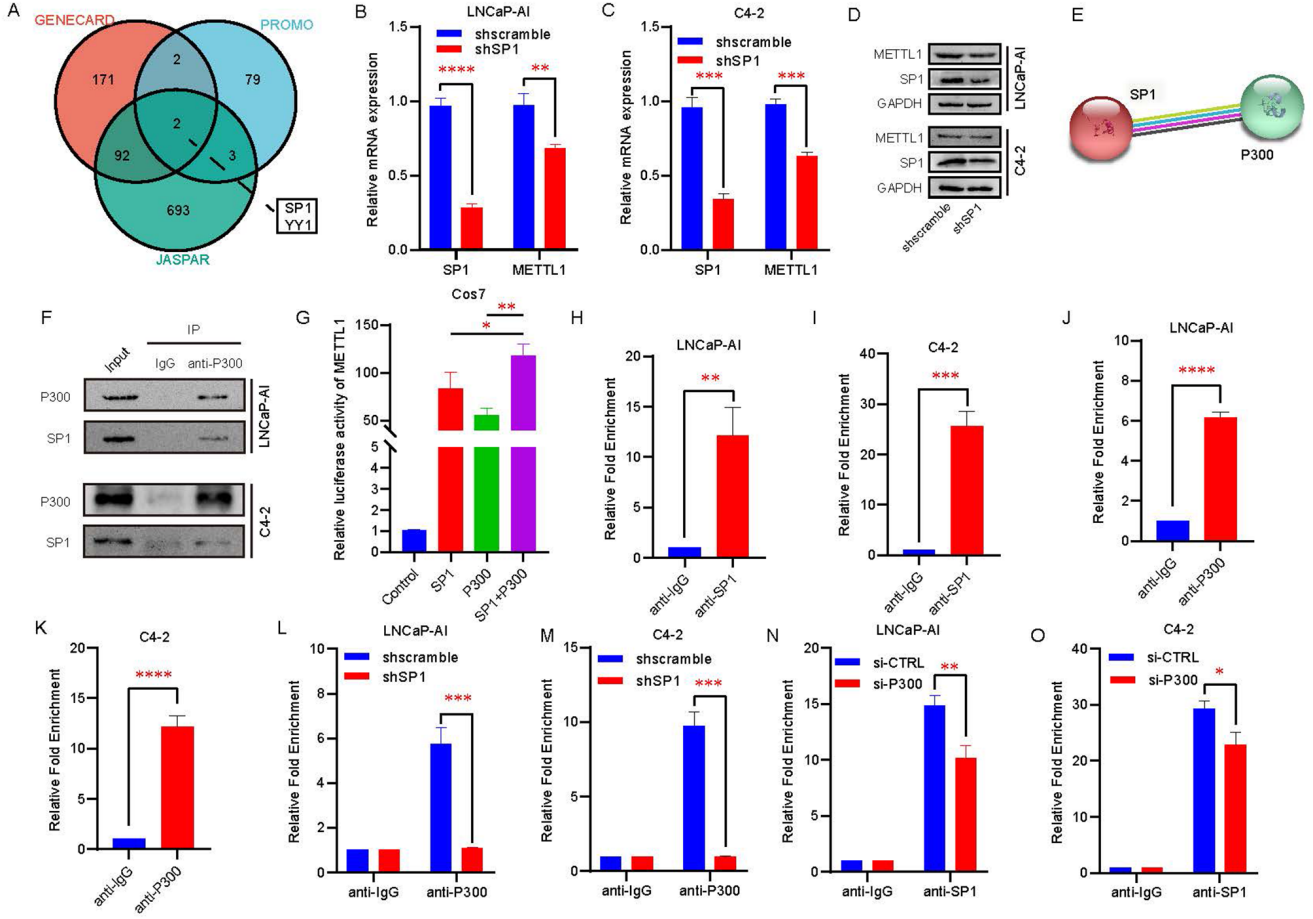
P300/SP1 complex regulates the transcriptional activity of METTL1. **A** GENECARD, PROMO, and JASPAR databases were used to predict the potential transcription factor (TF) of METTL1. **B-D** Effect of SP1 knockdown on METTL1 mRNA (B-C) and protein (D) levels. **E** Predicting the combination of SP1 and P300 with the online tool STRING. **F** Co-immunoprecipitations assay (Co-IP) was performed to verify the interaction between SP1 and P300. **G** Transcriptional regulation of SP1 and/or P300 on METTL1 expression was performed using a luciferase reporter assay in Cos7 cells. **H-K** CHIP-qPCR was implemented to prove the combination between SP1 (H-I) or P300 (J-K) and METTL1 promoter region in LNCaP-AI and C4-2 cells directly and indirectly. **L-O** CHIP-qPCR was used to determine the enrichment of P300 (L-M) or SP1 (N-O) at the promoter of METTL1 in control and SP1 or P300 deficiency LNCaP-AI and C4-2 cells. **P < 0.01; ***P < 0.001; ****P < 0.0001

We know that P300-mediated H3K27ac modification is an important marker of chromatin accessibility and that elevated levels of H3K27ac tend to suggest that the genome presents a relatively permissive chromatin environment in which transcription factors and other transcription-related factors are more likely to bind to binding motifs of relevant gene promoter regions, thereby regulating gene transcription. To further delve into the molecular mechanisms leading to transcriptional upregulation of METTL1, we first predicted potential transcription factors of METTL1 through GENECARD (https://www.genecards.org/), PROMO (https://alggen.lsi.upc.es/cgi-bin/promo_v3/promo/promoinit.cgi?dirDB=TF_8.3), and JASPAR (https://jaspar.genereg.net/) websites (Supplementary Table 6). SP1 and YY1 were found to be shared by all three databases (Fig. 4A). In view of the important role of SP1 in the transcriptional regulation of genes crucial for prostate cancer, we constructed cell lines with stable knockdown of SP1 in LNCaP-AI and C4-2 and found that silencing of SP1 decreased the expression level of METTL1 (Fig. 4B–D). In addition to its acetyltransferase activity, P300, as an important transcriptional co-activator, is often involved in the transcriptional regulation of genes through different modes of action. Here, we predicted via bioinformatic means that SP1 and P300 may interact with each other (Fig. 4E). Subsequently, we confirmed the existence of mutual binding by immunoprecipitation experiments (Fig. 4F). Next, we verified via luciferase reporter assay in COS7 cells that the transcriptional activity of METTL1 was stronger when both SP1 and P300 were present than when they were present alone (Fig. 4G). To clarify the manner in which the SP1 and P300 complexes bind to the METTL1 promoter region, a series of CHIP-qPCR experiments were performed. We found on one hand that SP1 and P300 can bind to the METTL1 promoter region either directly or indirectly (Fig. 4H - K). On the other hand, we suggested that after silencing SP1, P300 almost ceased to bind to the METTL1 promoter region (Fig. 4L and M). However, after the suppression of P300, SP1 binding to the METTL1 promoter region was diminished, but the binding was still present (Fig. 4N and O). These results demonstrate that the transcription factor SP1 is able to form a complex with P300 to mediate transcriptional activation of METTL1 and that SP1, but not P300, can bind directly to the METTL1 promoter region to initiate transcription.

### METTL1 stabilizes CDK14 mRNA by mediating internal m7G modification

**Fig. 5:**
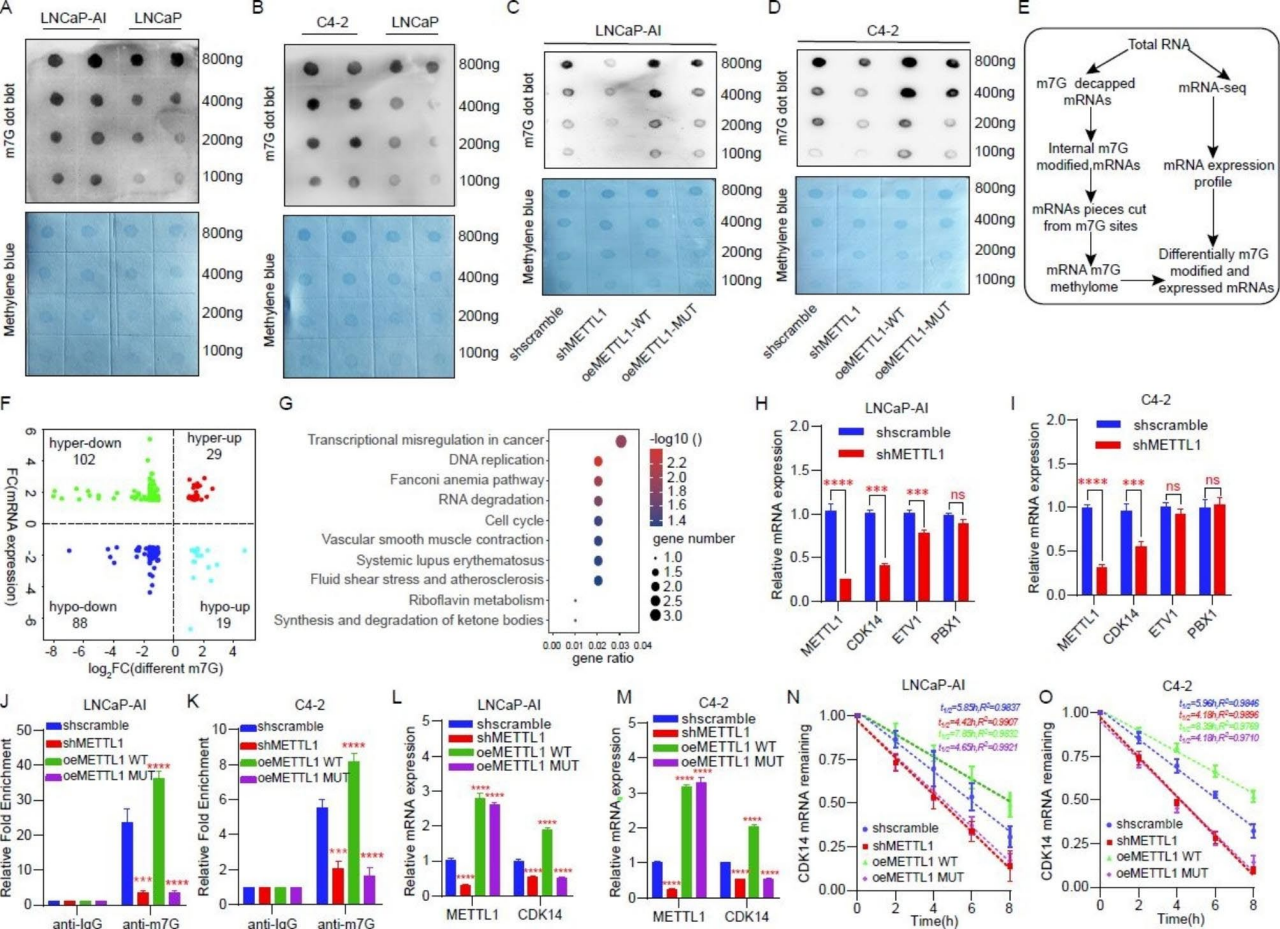
METTL1 stabilizes CDK14 mRNA by mediating internal m7G modification. **A-B** The RNA dot blot assay was performed to detect the different mRNA internal m7G modification levels between CPRC and HSPC cells. Methylene blue was used as a control. **C-D** Changes in m7G level after METTL1 knockdown, wild or mutant METTL1 overexpression in LNCaP-AI and C4-2 cells. **E** Flowchart of RNA transcriptome sequencing (RNA-seq) and mRNA internal m7G AlkAniline-Seq in control and METTL1 knockdown LNCaP-AI cells. **F** Quadrant diagram showing genes with differential mRNA expression (up or down) and significantly altered m7G (hyper or hypo) after METTL1 inhibition. The screening thresholds:|Fold change|>1.5 and p-value < 0.05. **G** KEGG enrichment analysis of differential genes with down-regulated levels of both mRNA and m7G. **H-I** RT-qPCR was used to validate the related genes change in the “Transcriptional misregulation in cancer” pathway in LNCaP-AI and C4-2 cells. **J-K** The CDK14 mRNA internal m7G level was measured by m7G RNA immunoprecipitation assay (MeRIP) using a 10ug m7G specific antibody among groups with different treatments as indicated in CRPC cells lines LNCaP-AI and C4-2. **L-M** CDK14 mRNA level was detected via RT-qPCR among groups with different treatments as shown. **N-O** The remaining CDK14 mRNA level in actinomycin D (ACTD) treated LNCaP-AI and C4-2 cells at the indicated time points were measured by RT-qPCR. ***P < 0.001; ****P < 0.0001

To further explore the molecular mechanisms by which METTL1 affects the proliferation and invasive capacity of CRPC cell lines, we first verify its biological function in CRPC. Considering that METTL1 is a methyltransferase responsible for catalyzing m7G modifications in a variety of RNAs including the interior of mRNA molecules. We examined the difference in m7G levels inside mRNAs between HSPC and CRPC cells. After treating the cells with a decapping enzyme, we found that m7G modification levels inside mRNAs were more abundant in LNCaP-AI and C4-2 cells compared to LNCaP cells (Fig. 5A and B). Next, METTL1 wild-type and enzyme activity mutant overexpression plasmids were constructed. We discovered that m7G levels within the mRNA of CRPC cells were significantly reduced after METTL1 silencing via dot blot assays and that m7G levels within the mRNA were restored after transfection with the METTL1 wild-type overexpression plasmid, but no increase in m7G levels was seen in cells transfected with the METTL1 enzymatic activity mutant plasmid (Fig. 5C and D). These results suggest that m7G modification within mRNA is catalyzed by METTL1. To further elucidate the biological role played by METTL1-catalyzed m7G modification within mRNA, we performed a combined RNA-seq and mRNA internal m7G AlkAniline-Seq analysis and screened for differential genes using cells with stably knocked-down METTL1 and control cells (Fig. 5E and F, Supplementary Table 7). We then carried out functional enrichment analysis of genes whose mRNA and m7G levels were down-regulated by knockdown of METTL1 and found that these genes were mainly enriched in transcriptional dysregulation in cancer, etc. pathways (Fig. 5G, Supplementary Table 8). We then verified that CDK14 in this pathway was most significantly regulated by METTL1 (Fig. 5H and I). To figure out and confirm that the regulation of CDK14 by METTL1 is dependent on methyltransferase activity, on one hand, we elaborated via MeRIP-qPCR that m7G levels within CDK14 mRNA decreased after silencing METTL1, and increased after transfection of METTL1 wild-type plasmids, while the m7G levels were not significantly altered after transfection with the mutant METTL1 plasmid (Fig. 5J and K). On the other hand, CDK14 mRNA levels were changed in line with its m7G levels (Fig. 5L and M). In order to explore more closely the reasons for the consistent changes in CDK14 mRNA and m7G levels within mRNA, we performed an RNA degradation assay. The experimental results indicated that the silencing of METTL1 resulted in a shortened CDK14 mRNA half-life. Overexpression of the METTL1 wild-type plasmid prolonged the CDK14 mRNA half-life, while overexpression of the mutant METTL1 plasmid did not show a significant change in CDK14 mRNA half-life (Fig. 5N and O). These results suggest that METTL1, a methyltransferase, protects CDK14 mRNA from degradation by causing m7G modification within the CDK14 mRNA.

### METTL1 regulates CRPC proliferation and invasion by modulating the expression of CDK14

**Fig. 6:**
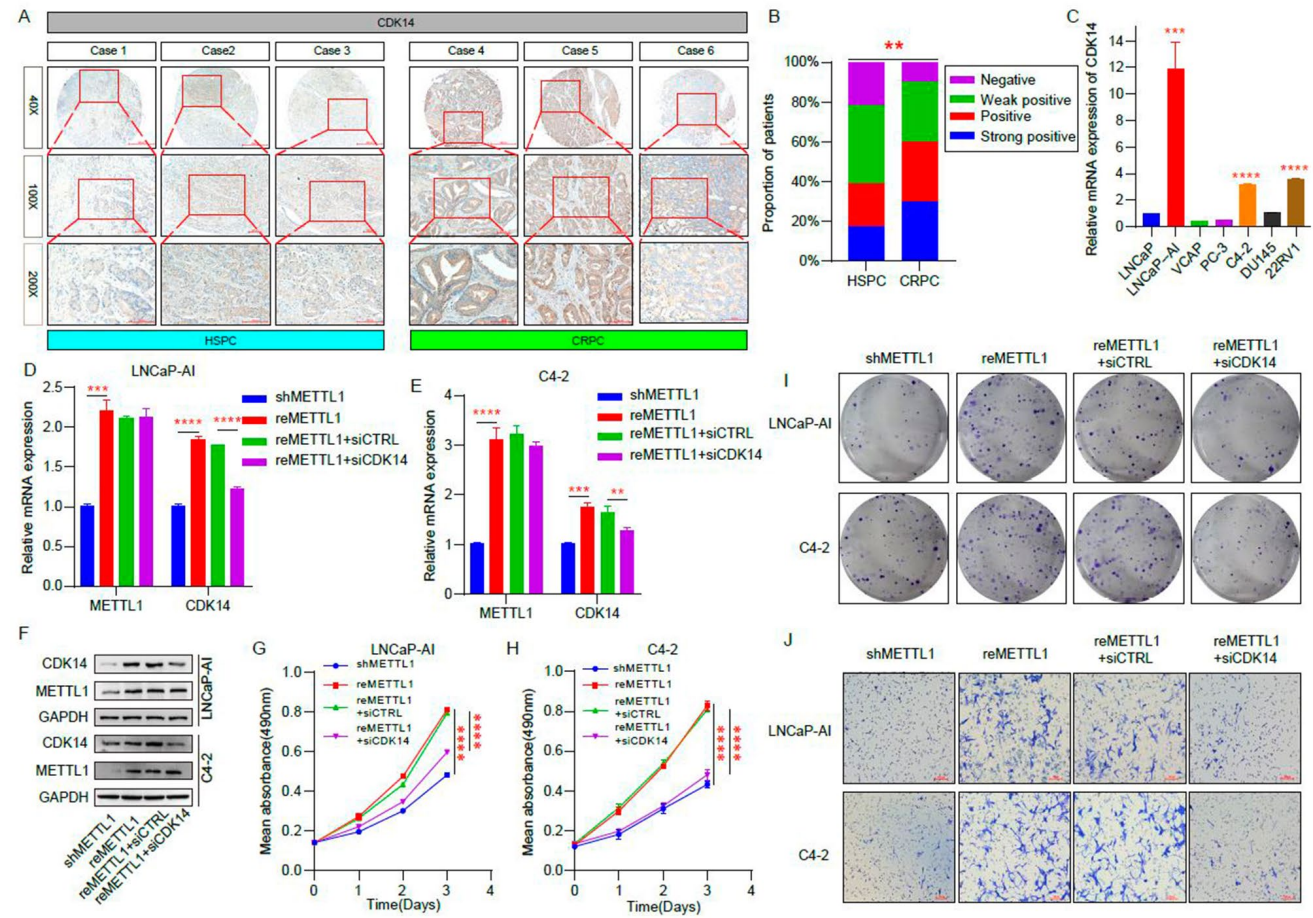
METTL1 regulates CRPC proliferation and invasion by modulating the expression of CDK14. **A** Validation of CDK14 expression in TMA containing HSPC and CRPC tissues using IHC assay. Objective magnification: 40X, 100X, and 200X. **B** The proportion of patients with different CDK14 levels according to the IHC staining result. **C** The different mRNA expressions of CDK14 between HSPC and CRPC cell lines by RT-qPCR. **D-F** RT-qPCR and western blot were performed to detect the CDK14 level after METTL1 was rescued and inhibition of CDK14 using siRNA or not. **G-I** Proliferation ability of LNCaP-AI and C4-2 were assessed through MTT and colony formation experiments after METTL1 was rescued and CDK14 was suppressed. **J** Invasion capacity of LNCaP-AI and C4-2 were evaluated via transwell experiments after METTL1 was rescued and CDK14 was suppressed. **P < 0.01; ****P < 0.0001

To further confirm that METTL1 can make a difference in the malignant biology of CRPC by regulating CDK14 expression, we first examined the expression of CDK14 between HSPC and CRPC tissues, and the immunohistochemical staining results demonstrated that CDK14 level was higher in CRPC compared to HSPC patients (Fig. 6A and B). In addition, we also found that CDK14 was significantly upregulated in LNCaP-AI, C4-2, and 22RV1 in cellular assays (Fig. 6C). We then overexpressed METTL1 (reMETTL1) in cells with stably knocked down METTL1 (shMETTL1) and simultaneously inhibited CDK14 expression using siRNA (reMETTL1 + siCDK14) and compared the differences in proliferation and invasion ability among the different groups (Fig. 6D–F). MTT and cloning formation assays indicated that cell proliferation was enhanced after overexpression of METTL1 compared to cells in the knockdown METTL1 group, However, inhibition of CDK14 expression on this basis reduced cell proliferation (Fig. 6G–I). Similarly, the transwell assay showed that the invasive ability of CRPC cells was enhanced after overexpression of METTL1, while impaired by inhibition of CDK14 expression (Fig. 6J). The above results show that METTL1 can indirectly regulate the proliferation and invasion of CRPC cells through CDK14.

### METTL1 promotes CRPC progression in vivo

**Fig. 7:**
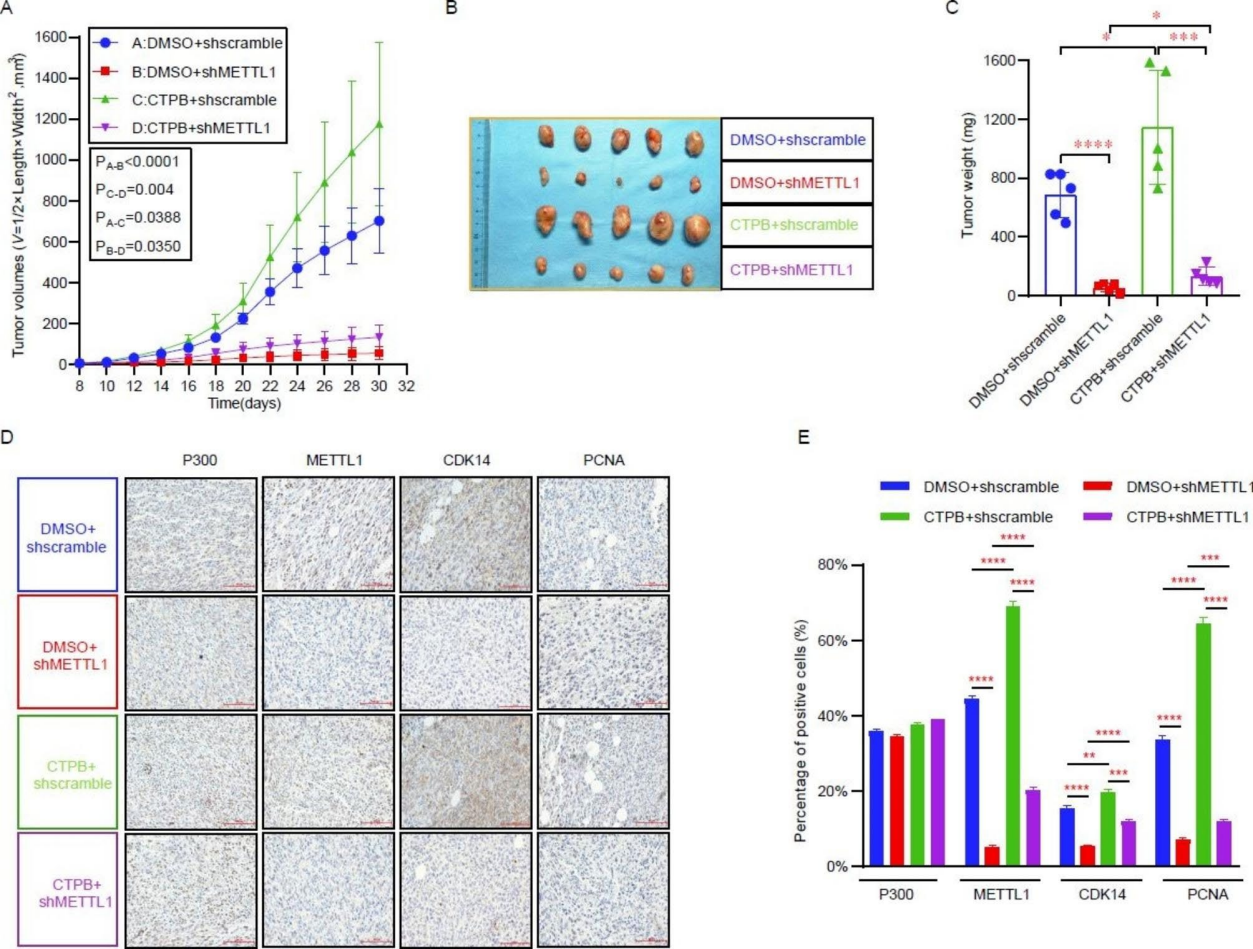
METTL1 promotes CRPC progression in vivo. **A** Tumor volume growth curves of nude mice in different treatment groups. The tumor volume was calculated according to the formula V=(length*width^2^)/2 every two days. Volume unit: mm^3^. CTPB: P300 activator. **B-C** Tumor size and weight (mg) of nude mice in different treatment groups. **D** P300, METTL1, CDK14, and PCNA expression were detected by IHC in different treatment groups of nude mice. Scale bars = 100 μm. **E** Quantitative analysis of IHC staining results using ImageJ software

To determine the roles of METTL1 in facilitating CRPC progression in vivo, we used immunodeficient nude mice for subcutaneous transplantation tumor experiments. During the in vivo experiments, after the mice had formed subcutaneous graft tumors, we fed the experimental (shMETTL1) and control (shscramble) groups of nude mice with P300 activator or DMSO-treated water and food from day 8 onwards and calculated the tumors volume according to the formula *V*=(length*width^2^)/2 every two days until the end of the experiment. The results indicated that the subcutaneous transplanted tumors in the knockdown METTL1 group grew more slowly and had a smaller final volume and mass compared to the control group. The control group of nude mice fed with CTPB (CTPB + shscramble) had the fastest growth rate and the largest volume and weight of subcutaneous transplanted tumors. At the same time, the knockdown group (CTPB + shMETTL1) was faster growing and had larger tumor volume and mass compared to the knockdown METTL1 group (Fig. 7A–C). At the end of the experiment, the subcutaneous transplanted tumors were removed and embedded using paraffin wax. After sectioning and immunohistochemical staining of paraffin-embedded tissues, we found that both CDK14 and the cell proliferation marker PCNA were reduced after METTL1 suppression. Compared to the shscramble and shMETTL1 groups, CDK14 and PCNA expression were elevated in nude mice raised with CTPB, which is consistent with our previous cellular experiments (Fig. 7D and E). The in vivo experiments more rigorously confirmed the important role of METTL1 in promoting CRPC progression.

## Discussion

In recent years, epigenetic modifications on RNA molecules have been increasingly identified. Among these RNA modifications, the m6A molecule is the most well-known. In addition, besides m7G modifications at the cap of mRNA, m7G present in tRNA, miRNA, rRNA, and within mRNA are gradually being uncovered [3]. Correspondingly, the important biological functions played by m7G modifications are also gradually attracting attention. A series of studies have shown that m7G modifications are involved in different transactivation processes of RNA molecules, including tRNA stability, miRNA processing and maturation, and mRNA translation and stability. Like m6A modification, m7G is also a reversible modification process. m7G on mammalian RNAs is mainly carried out by the METTL1/WDR4 complex as a “writer”, in which METTL1 plays a catalytic role as a methyltransferase [4]. Recently, METTL1 and its mediated m7G modifications have been found to play an essential role in neurodegenerative diseases, including Alzheimer’s disease, and in the malignant progression of tumors [3, 7, 8, 21–23].

**Fig. 8:**
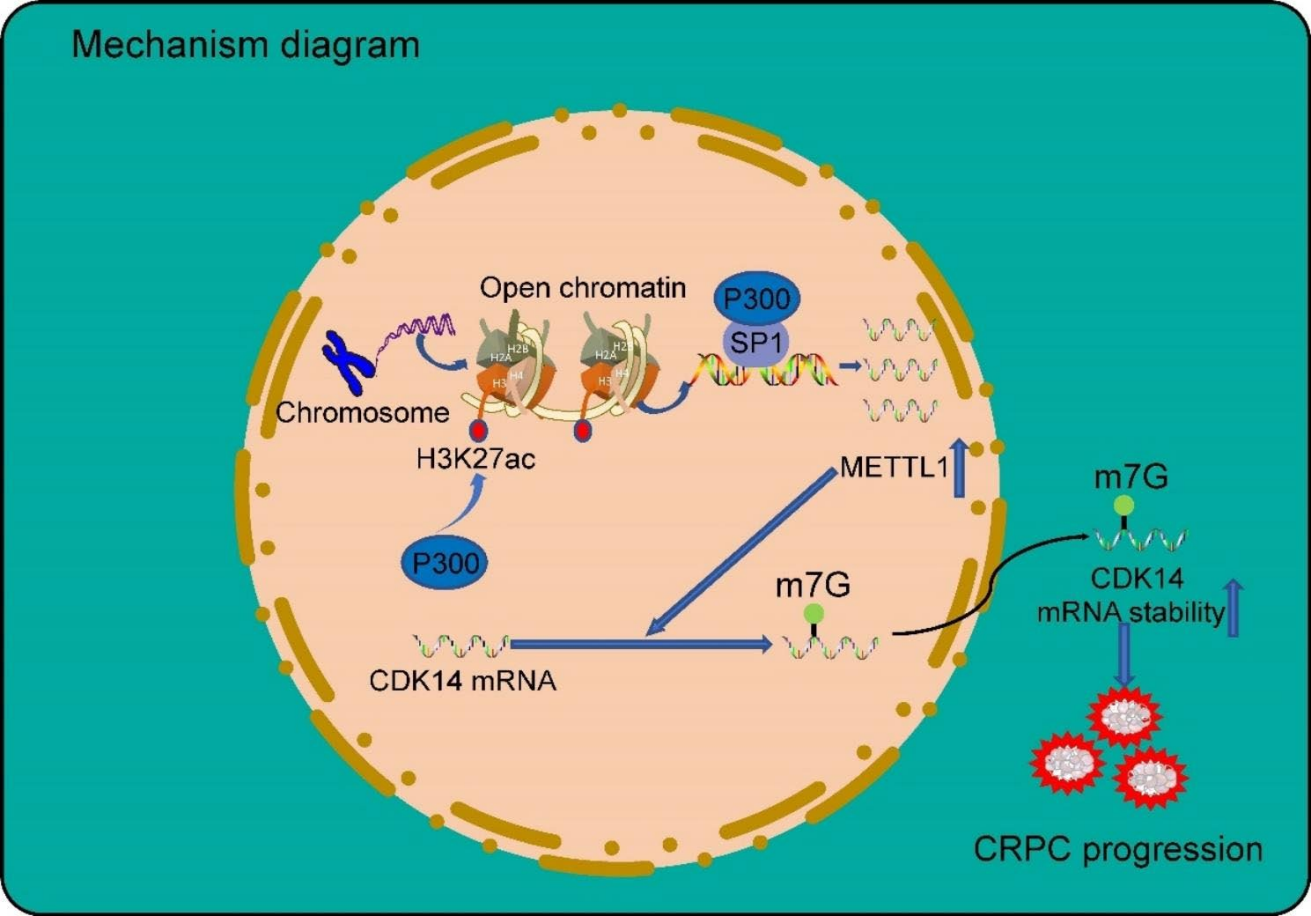
Mechanism diagram. P300 and the transcription factor SP1 form a complex that binds to the promoter region of the METTL1 gene to regulate its transcriptional activation, leading to aberrant high METTL1 expression in CRPC. Furthermore, METTL1 protects CDK14 mRNA from degradation by adding m7G modifications within it and ultimately leading to CRPC progression

In the present study, we found that the histone acetyltransferase P300 can mediate H3K27 acetylation modifications that are heavily enriched in the promoter region of METTL1, an important marker for chromatin accessibility. Subsequently, P300 and the transcription factor SP1 form a complex that binds to the promoter region of the METTL1 gene to regulate its transcriptional activation, resulting in abnormally elevated METTL1 expression in CRPC. Furthermore, Combining RNA-seq and m7G AlkAniline-Seq, as well as cellular experimental validation, we revealed that METTL1 enhances the stability of CDK14 mRNA by adding m7G modifications within it, ultimately leading to CRPC progression (Fig. 8).

As a transcriptional cofactor, P300 often acts as a bridge, a scaffold, or relies on its acetylase activity to play a key bridging role in the transcription of genes. Previous studies have found that P300 can collaborate with transcription factors, including SP1, to promote transcriptional activation of downstream target genes, which in turn can play a role in tumorigenesis and progression [24, 25]. In multiple myeloma, SP1 and P300 form a complex that regulates the transcription of IQGAP1 and thus promotes tumor cell proliferation [13]. A recent study published by Zeng Lei’s team also found that the transcriptional regulator BRD4-NUT was able to recruit P300 to initiate the transcription of downstream genes [26]. Our study reveals for the first time that in CRPC, P300 is able to bind to SP1 to form a complex, which subsequently binds to the promoter region of the METTL1 gene through its SP1 component to initiate its transcription, leading to an abnormal elevation of METTL1 in CRPC. In terms of clinical application, considering the significant role of P300 in tumors, small molecule inhibitors targeting P300 have been developed and gradually put into clinical use and have achieved certain efficacy. However, due to the wide range of downstream molecules regulated by P300 and their functional importance, these existing inhibitors have more or less toxic side effects. Our study reveals that METTL1, one of the downstream molecules of P300, plays an important role in the progression of CRPC. This suggests that the later clinical development of related drugs targeting METTL1 rather than P300 may be of benefit to patients by reducing the side effects of CRPC treatment.

Previous studies have shown that METTL1 plays an important oncogenic role in the progression of various solid tumors by mediating m7G modification of RNA. In tumors including nasopharyngeal carcinoma, lung cancer, hepatocellular carcinoma, and squamous cell carcinoma of the head and neck, METTL1 is able to mediate tRNA m7G modifications that regulate the translation process and ultimately lead to tumor progression [7, 8, 22, 27, 28]. Moreover, the METTL1/WDR4 complex promotes the maturation of miRNA let-7e processing through its m7G methyltransferase activity, which subsequently module the ability of cell migration [29]. However, there are no reports of METTL1 being associated with tumors in prostate cancer. In our study, we revealed for the first time the relationship between m7G modification inside mRNA and mRNA degradation through combined analysis of RNA-seq and m7G AlkAniline-Seq inside mRNA as well as preliminary experimental validation, leading to the important conclusion that METTL1 can add m7G modification inside CDK14 mRNA, which in turn makes its mRNA more stable and ultimately promotes CRPC progression.

Nevertheless, there are still some shortcomings in our study. It has been shown that in prostate cancer, P300 is able to bind to the androgen receptor (AR) and acetylate it for modification, ultimately leading to enhanced transcription of AR downstream target genes [30–33]. Our findings provide preliminary evidence that SP1 and P300 form a complex with enhanced transcriptional activity of METTL1, but whether this enhanced transcriptional activity correlates with the acetyltransferase activity of P300 has not been further elucidated. In addition, we have confirmed that METTL1 increases the stability of CDK14 mRNA by adding m7G modifications to its interior based on high-throughput sequencing results and preliminary experiments. Whether the relationship between m7G modifications within mRNA and molecular stability is universal needs to be further confirmed. Finally, the role of CDK14, the most atypical cell cycle-dependent kinase, in prostate cancer and its mechanism of action still needs to be further explored.

Given this, we plan to continue to explore the role of P300 in the regulation of acetylation of non-histone SP1 and to further unravel the potential molecular mechanisms underlying the role of CDK14 as an oncogenic factor in CRPC. We hope that the results of this study will provide new perspectives and theoretical support for the clinical management of patients with refractory CRPC.

## Conclusions

Overall, our findings reveal that METTL1 plays an oncogenic role in the progression of CRPC. Mechanistically, the P300/SP1 complex facilitates the transcriptional activation of METTL1, leading to aberrant high METTL1 expression in CRPC. Finally, as an m7G methyltransferase, METTL1 protects CDK14 mRNA from degradation by adding m7G modifications within it and ultimately leading to CRPC progression.

### Electronic supplementary material

Below is the link to the electronic supplementary material.

Supplementary Material 1

## Data Availability

The authors confirm that the data and material supporting the findings of this study are available within the article [and/or] its supplementary materials.

## Abbreviations

m7G: N7-methylguanosine
METTL1: methyltransferase 1
PCa: prostate cancer
HSPC: hormone-sensitive prostate cancer
CRPC: castrate-resistant prostate cancer
ADT: androgen deprivation
DEG: differentially expressed genes
m6A: N6-methyladenosine
m1A: N1-methyladenosine
