# Supplementary material for: P300/SP1 complex mediating elevated METTL1 regulates CDK14 mRNA stability via internal m7G modification in CRPC
Source: J Exp Clin Cancer Res. 2023 Aug 21;42:215. doi: 10.1186/s13046-023-02777-z (PMC10440916; doi:10.1186/s13046-023-02777-z)
Supplement: Supplementary file 1 — Supplementary Material 1 [file 13046_2023_2777_MOESM1_ESM.docx]

Table1 siRNA/shRNA target sequences

| Oligonucleotide Names | Sequences (5'-3') |
| --- | --- |
| siP300 | GCAUCAGAUCUGUGUCCUUTT |
| siCDK14 | GGAAGUUGGUAGCUCUGAATT |
| shMETTL1 | GGAATGTAGTCCATCTAAACTCG  AAAGTTTAGATGGACTACATTCC |
| shSP1 | CCACTCCTTCAGCCCTTATTA |

Table2 Antibodies involved in this work

| Names | Corporation | Cat No | Application | Dilution ratio/Dosage  Concentration |
| --- | --- | --- | --- | --- |
| GAPDH | Abcam | Ab181602 | WB | 1:5000 |
| β-actin | Abcam | Ab6276 | WB | 1:5000 |
| METTL1 | Proteintech | 14994-1-AP | IHC/WB | 1:1000/1:2000 |
| H3K27ac | Abcam | Ab4729 | WB | 1 µg/mL |
| H3K27ac | Abcam | Ab4729 | CHIP | 5 µg |
| Histone 3 | Abcam | Ab1791 | WB | 1:2000 |
| P300 | Abcam | Ab275379 | IHC | 1:1000 |
| P300 | Abcam | Ab275378 | WB/CHIP/IP | 1:1000/5µg/5µg |
| SP1 | Abcam | Ab227383 | WB/CHIP/IP | 1:5000/5µg/5µg |
| m7G | MBL | RN017M | Dot blot/  MeRIP | 1 µg/mL/10µg |
| CDK14 | Abcam | Ab224098 | IHC | 1:20 |
| CDK14 | Abcam | Ab167928 | WB | 1:1000 |
| PCNA | Abcam | Ab92552 | IHC | 1:500 |

Table3 Drugs

| Drug Names | Corporation | Cat No |
| --- | --- | --- |
| C646 | Selleckchem | S7152 |
| Decapping Enzyme | Enzymax | KY 40503 |
| Actinomycin D | MCE | HY-17559 |
| CTPB | Abcam | Ab142224 |

Table4 Primers for RT-qPCR.

| Primer Names | Sequences (5'-3') |
| --- | --- |
| GAPDH Forward | ACAACTTTGGTATCGTGGAAGG |
| GAPDH Reverse | GCCATCACGCCACAGTTTC |
| CDK14 Forward | CACCAGCGTTATATTTTGCACAG |
| CDK14 Reverse | ATCTGCCAGCTTTAACTCCCC |
| METTL1 Forward | CGCCTGTCTCCGTAGCAAT |
| METTL1 Reverse | GGACTGATGATTCGCCACTTG |
| SP1 Forward | AGTTCCAGACCGTTGATGGG |
| SP1 Reverse | GTTTGCACCTGGTATGATCTGT |
| P300 Forward | GCTTCAGACAAGTCTTGGCAT |
| P300 Reverse | ACTACCAGATCGCAGCAATTC |
| ETV1 Forward | CTGAACCCTGTAACTCCTTTCC |
| ETV1 Reverse | AGACATCTGGCGTTGGTACATA |
| PBX1 Forward | CATGCTGTTAGCGGAAGGC |
| PBX1 Reverse | CTCCACTGAGTTGTCTGAACC |

Table5 Differentially expressed genes of whole transcriptome resequencing of CRPC versus HSPC

| Gene | logFC | logCPM | PValue | FDR |
| --- | --- | --- | --- | --- |
| FABP7 | 12.20733 | 6.261537 | 1.55E-07 | 0.000156 |
| FRG2DP | 11.84667 | 2.44681 | 3.33E-05 | 0.006607 |
| GABRA6 | 11.60382 | 2.20605 | 7.29E-06 | 0.002431 |
| FAM9C | 11.53685 | 2.139573 | 0.000287 | 0.026382 |
| UGT2A3 | 11.44584 | 2.049767 | 0.000301 | 0.027182 |
| CFC1 | 10.93824 | 1.548522 | 0.000126 | 0.015754 |
| AL121949.1 | 10.79088 | 1.403478 | 0.000513 | 0.037262 |
| UGT2B17 | 10.50062 | 1.118481 | 0.0002 | 0.02149 |
| AL121949.2 | 10.47444 | 1.092844 | 0.000692 | 0.04449 |
| AC120498.4 | 9.968244 | 0.598966 | 0.001183 | 0.061555 |
| AC027369.3 | 9.895216 | 0.527378 | 2.13E-05 | 0.004972 |
| AC064834.1 | 9.724489 | 0.362831 | 0.001597 | 0.072666 |
| LINC02030 | 9.706851 | 0.345173 | 5.51E-05 | 0.009377 |
| AC110800.1 | 9.563976 | 0.207066 | 0.001978 | 0.082451 |
| CES5A | 9.524552 | 0.170127 | 0.000924 | 0.052282 |
| AC099524.1 | 9.389305 | 0.039277 | 0.00246 | 0.095025 |
| NPM1P28 | 9.376442 | 0.026954 | 0.002493 | 0.095481 |
| GRAMD4P5 | 9.303569 | -0.04277 | 0.002724 | 0.100592 |
| ZNF560 | 9.273521 | 1.716249 | 1.64E-05 | 0.00396 |
| HMGB1P38 | 9.258339 | -0.08477 | 0.002828 | 0.102889 |
| TRGJP1 | 9.124119 | -0.21259 | 0.00028 | 0.026292 |
| DUSP21 | 9.107346 | -0.22973 | 0.003578 | 0.116716 |
| AP005121.1 | 9.094883 | 1.53565 | 4.64E-06 | 0.001734 |
| AC245036.5 | 9.010603 | -0.3214 | 0.001984 | 0.082519 |
| TEX19 | 8.838565 | -0.48346 | 0.000822 | 0.049037 |
| TM4SF20 | 8.831385 | 6.439504 | 3.00E-05 | 0.006244 |
| LINC01069 | 8.685338 | -0.62719 | 0.006493 | 0.156951 |
| LINC02326 | 8.650385 | -0.6585 | 0.000712 | 0.045331 |
| LINC00560 | 8.597939 | -0.70681 | 0.007252 | 0.164639 |
| CXADRP1 | 8.542558 | -0.75996 | 0.004182 | 0.125666 |
| LINC02163 | 8.509376 | -0.78885 | 0.008273 | 0.176607 |
| AP001476.1 | 8.499432 | -0.79816 | 0.002777 | 0.101569 |
| LINC01443 | 8.487517 | -0.80914 | 0.003139 | 0.10957 |
| CASC9 | 8.474711 | -0.82086 | 0.008679 | 0.182216 |
| MKNK2P1 | 8.472168 | -0.82513 | 0.008892 | 0.18403 |
| OR5T3 | 8.447782 | -0.84764 | 0.009191 | 0.187701 |
| CYP1A1 | 8.365455 | -0.92248 | 0.001951 | 0.081607 |
| AC124856.1 | 8.347788 | -0.93902 | 0.001549 | 0.07179 |
| AC093904.4 | 8.339964 | -0.9447 | 0.010683 | 0.200708 |
| AC009901.2 | 8.339964 | -0.9447 | 0.01069 | 0.200708 |
| LINC02445 | 8.274292 | -1.0047 | 0.01172 | 0.212324 |
| CR769775.1 | 8.26443 | -1.01511 | 0.000767 | 0.047 |
| SLC17A2 | 8.233403 | -1.04194 | 0.012448 | 0.217493 |
| AC087379.2 | 8.219512 | -1.05457 | 0.012686 | 0.219302 |
| HCFC2P1 | 8.147977 | -1.11942 | 0.014155 | 0.232075 |
| TEKT4 | 8.138043 | -1.12877 | 0.001329 | 0.065769 |
| PON1 | 8.044653 | 5.017174 | 4.82E-06 | 0.001782 |
| AL390061.1 | 7.9713 | -1.28076 | 0.01872 | 0.268576 |
| PITX2 | 7.960041 | 0.355137 | 2.77E-06 | 0.001294 |
| LINC01221 | 7.954089 | -1.29615 | 0.019235 | 0.271665 |
| AC104135.1 | 7.951539 | -1.29817 | 0.00369 | 0.118552 |
| C10orf62 | 7.948356 | -1.29906 | 0.005697 | 0.147485 |
| HBQ1 | 7.907549 | -1.33605 | 0.000381 | 0.031419 |
| AC087258.1 | 7.893228 | -1.34792 | 0.007497 | 0.167397 |
| AC097512.1 | 7.83831 | -1.39642 | 0.021871 | 0.291411 |
| AC090503.2 | 7.827496 | -1.40869 | 0.022929 | 0.298689 |
| ANKRD20A2 | 7.808469 | -1.4255 | 0.023557 | 0.301139 |
| AC091043.1 | 7.801483 | -1.42892 | 0.023221 | 0.300391 |
| IRX1 | 7.789188 | -1.44252 | 0.024168 | 0.30441 |
| ZNF971P | 7.726211 | -1.49638 | 0.026126 | 0.315499 |
| RPRML | 7.718093 | -1.50303 | 0.006448 | 0.156814 |
| LINC00163 | 7.705648 | -1.51438 | 0.003022 | 0.107337 |
| SIGLEC14 | 7.642411 | -1.56946 | 0.001364 | 0.066732 |
| SNRPGP13 | 7.622884 | -1.5865 | 0.030089 | 0.336223 |
| AC012668.3 | 7.620179 | 0.122288 | 0.005078 | 0.138928 |
| LINC02151 | 7.6013 | -1.60522 | 0.031002 | 0.340778 |
| AC121764.2 | 7.587004 | -1.61809 | 0.009026 | 0.185643 |
| AC021146.1 | 7.58441 | -1.61878 | 0.012117 | 0.215026 |
| COL20A1 | 7.580725 | -1.62466 | 0.03245 | 0.347236 |
| RN7SKP257 | 7.580725 | -1.62466 | 0.032449 | 0.347236 |
| AC093904.2 | 7.58058 | -1.6218 | 0.0187 | 0.268576 |
| RPS27P1 | 7.448087 | -1.73587 | 0.015162 | 0.240123 |
| DPP3P2 | 7.447354 | -0.05251 | 0.00108 | 0.058432 |
| AL713998.2 | 7.443466 | -1.7394 | 0.038677 | 0.377203 |
| ELL3 | 7.439809 | -1.74403 | 0.001759 | 0.075917 |
| HNRNPA1P12 | 7.433943 | 1.126587 | 0.000633 | 0.04175 |
| AC147055.4 | 7.419363 | -1.75991 | 0.039772 | 0.383746 |
| LINC00659 | 7.37477 | -1.79818 | 0.017253 | 0.258002 |
| IGLV8-61 | 7.372632 | -1.79984 | 0.016597 | 0.252804 |
| MYO16-AS1 | 7.35269 | -1.81709 | 0.015949 | 0.246659 |
| AC025580.1 | 7.295296 | 2.325182 | 1.49E-05 | 0.003656 |
| NPAP1P2 | 7.286649 | -1.87356 | 0.047251 | 0.414589 |
| AC106779.1 | 7.252404 | -1.90181 | 0.003672 | 0.118207 |
| RAF1P1 | 7.24496 | -1.90747 | 0.007842 | 0.171764 |
| AC099066.1 | 7.235669 | -1.9165 | 0.01953 | 0.274031 |
| GRAMD4P7 | 7.183246 | -0.2989 | 0.002891 | 0.10409 |
| GTF2IP13 | 7.155996 | -1.98312 | 0.008682 | 0.182216 |
| OTOF | 7.155478 | -0.3705 | 0.003166 | 0.109723 |
| AC087879.1 | 7.153698 | -1.98256 | 0.034249 | 0.357142 |
| AC084816.1 | 7.150804 | 1.418253 | 0.000131 | 0.016077 |
| AP000523.1 | 7.121626 | 0.408428 | 0.001113 | 0.059565 |
| RN7SL455P | 7.105002 | -2.02361 | 0.006802 | 0.160305 |
| GPHA2 | 7.099172 | -2.02791 | 0.012405 | 0.217394 |
| GRID2 | 7.098719 | 5.052916 | 0.003154 | 0.109723 |
| AC068014.1 | 7.065599 | -2.05505 | 0.027795 | 0.325852 |
| KRT18P55 | 7.064403 | -2.05836 | 0.007373 | 0.165955 |
| AC121764.3 | 7.06313 | -2.05939 | 0.023419 | 0.30089 |
| AC111200.2 | 7.061188 | -2.06076 | 0.0075 | 0.167397 |
| AC008163.1 | 7.055092 | -2.06532 | 0.023457 | 0.301087 |
| DUSP5P1 | 7.038854 | 0.773506 | 0.0002 | 0.02149 |
| BX005266.2 | 7.037538 | -2.08079 | 0.025953 | 0.314449 |
| LINC01293 | 7.037048 | -0.48358 | 0.000879 | 0.050858 |
| AC084125.2 | 7.036537 | -2.07892 | 0.026092 | 0.315468 |
| DNAJB5P1 | 7.021094 | -2.09281 | 0.007316 | 0.165406 |
| FAM95B1 | 7.009474 | -2.10404 | 0.042005 | 0.392769 |
| AURKAP2 | 7.006708 | -2.1034 | 0.010693 | 0.200708 |
| FGF7P1 | 6.940297 | 1.810997 | 1.11E-05 | 0.003092 |
| KC877373.1 | 6.903642 | -2.18596 | 0.036172 | 0.366186 |
| AC008464.1 | 6.891632 | -2.19754 | 0.031319 | 0.341465 |
| AC027237.1 | 6.848136 | -2.23148 | 0.011155 | 0.206331 |
| OR4S2 | 6.847243 | 0.158561 | 0.009249 | 0.187736 |
| RNU6-1219P | 6.838103 | -2.23854 | 0.035409 | 0.362429 |
| AC007881.2 | 6.79659 | -2.27086 | 0.040403 | 0.386034 |
| AC134312.4 | 6.769426 | -2.29317 | 0.011267 | 0.207406 |
| AGTR2 | 6.753647 | -2.30759 | 0.046085 | 0.409776 |
| PM20D1 | 6.749178 | 4.955193 | 0.002363 | 0.092805 |
| TMEM78 | 6.742553 | -2.31515 | 0.013891 | 0.230501 |
| LINC00993 | 6.738798 | 2.735972 | 0.001406 | 0.068007 |
| IGJP1 | 6.72953 | -2.3242 | 0.039524 | 0.382535 |
| WIZP1 | 6.713305 | -2.339 | 0.048435 | 0.419168 |
| MAN1B1-DT | 6.68696 | -2.35705 | 0.013476 | 0.226461 |
| AC131235.2 | 6.658651 | -2.37993 | 0.014329 | 0.233376 |
| AC103764.1 | 6.646208 | -2.38847 | 0.01685 | 0.255304 |
| LINC01551 | 6.642891 | -2.39064 | 0.043742 | 0.399687 |
| AC147055.3 | 6.633186 | -0.85444 | 0.007488 | 0.167397 |
| POM121L8P | 6.617306 | -2.41174 | 0.018796 | 0.26912 |
| BMS1P3 | 6.61646 | -2.41237 | 0.0475 | 0.415773 |
| RNU6-1054P | 6.596027 | -2.42614 | 0.021935 | 0.291691 |
| LINC01825 | 6.592042 | -2.42876 | 0.031994 | 0.344504 |
| LINC01906 | 6.587842 | 2.071476 | 0.000385 | 0.0316 |
| AL512625.2 | 6.582229 | -0.11412 | 0.000548 | 0.038039 |
| SPCS2P3 | 6.581655 | -2.4397 | 0.033533 | 0.352439 |
| TREML4 | 6.569593 | -2.44771 | 0.016898 | 0.255413 |
| RNU5E-4P | 6.560923 | -0.92322 | 0.000287 | 0.026382 |
| AC022730.2 | 6.552433 | -2.4592 | 0.023784 | 0.30246 |
| AL353807.2 | 6.524601 | -2.4817 | 0.017778 | 0.261695 |
| SYCE1 | 6.499792 | -0.97586 | 0.023528 | 0.301106 |
| AL445209.1 | 6.484463 | -2.51236 | 0.023524 | 0.301106 |
| IFNL1 | 6.466148 | -2.52438 | 0.026074 | 0.315362 |
| MAPRE1P3 | 6.430231 | -2.55212 | 0.023373 | 0.300575 |
| AC233723.1 | 6.422249 | -2.55731 | 0.020748 | 0.283034 |
| RNF225 | 6.420368 | -2.55851 | 0.030634 | 0.33889 |
| NETO1 | 6.398865 | 1.639806 | 1.34E-06 | 0.000744 |
| LINC00536 | 6.375819 | 1.596471 | 0.000518 | 0.037417 |
| AC006288.1 | 6.309854 | -2.63833 | 0.041136 | 0.38947 |
| AC105914.2 | 6.264002 | 1.008526 | 0.015435 | 0.242761 |
| AC009970.1 | 6.243633 | -1.2081 | 0.001639 | 0.073584 |
| SNX6P1 | 6.234183 | -1.17187 | 0.007741 | 0.170694 |
| AC003035.1 | 6.207392 | -2.71223 | 0.046713 | 0.412346 |
| MIR141 | 6.205616 | -2.71333 | 0.047564 | 0.416038 |
| AL022313.2 | 6.180488 | -1.22555 | 0.002667 | 0.099671 |
| CAMKV | 6.160623 | 0.791208 | 5.15E-05 | 0.008889 |
| ACTG1P15 | 6.158803 | -2.74737 | 0.04865 | 0.419715 |
| AC027338.1 | 6.154296 | -2.75008 | 0.041264 | 0.38947 |
| PFN1P11 | 6.153858 | -2.75034 | 0.033224 | 0.350925 |
| DYTN | 6.13715 | 2.45034 | 1.01E-06 | 0.000643 |
| NPPC | 6.123502 | -1.31528 | 0.001526 | 0.071 |
| RN7SKP220 | 6.114523 | -1.26661 | 0.037368 | 0.370913 |
| AP003550.1 | 6.089596 | -1.30915 | 0.001808 | 0.077452 |
| LGSN | 6.079588 | 1.577188 | 0.002365 | 0.092805 |
| TBX4 | 6.073555 | 0.534097 | 4.00E-05 | 0.0076 |
| AC010731.1 | 6.054146 | -1.32865 | 0.011353 | 0.208264 |
| GNG13 | 6.048295 | -0.67049 | 0.011228 | 0.20712 |
| AC104561.3 | 6.037757 | -2.83125 | 0.041033 | 0.38918 |
| AKR1D1 | 5.994322 | -0.64796 | 0.008856 | 0.183729 |
| AC019103.1 | 5.993116 | -1.42812 | 0.046632 | 0.411986 |
| ANKRD30BP1 | 5.983009 | 0.339773 | 0.000132 | 0.016077 |
| PABPC1L2B | 5.974143 | -1.39978 | 0.013141 | 0.224015 |
| RN7SL612P | 5.962088 | -2.88148 | 0.043563 | 0.399239 |
| BIN2P1 | 5.959121 | -2.88317 | 0.046517 | 0.411743 |
| PSMC1P2 | 5.957697 | -0.7528 | 0.006059 | 0.153076 |
| AC005011.1 | 5.95374 | -1.46241 | 0.048674 | 0.419721 |
| OR51D1 | 5.942667 | 1.710132 | 0.012081 | 0.215011 |
| CYP4F35P | 5.941662 | 0.878985 | 0.001589 | 0.072565 |
| RNF151 | 5.898883 | -2.92409 | 0.045997 | 0.409485 |
| PPP1R14C | 5.892555 | 4.346352 | 1.09E-05 | 0.003092 |
| OLA1P3 | 5.89245 | -2.9277 | 0.047509 | 0.415773 |
| ANKRD34B | 5.886108 | 2.144755 | 0.00017 | 0.019031 |
| AC090138.1 | 5.872418 | 0.366068 | 0.01439 | 0.233916 |
| AC093520.1 | 5.829116 | -1.56811 | 0.026893 | 0.320857 |
| QRSL1P1 | 5.770873 | -0.36335 | 0.016926 | 0.255561 |
| SLURP1 | 5.741564 | -1.60969 | 0.004108 | 0.124419 |
| LINC01291 | 5.674589 | 2.07049 | 1.93E-05 | 0.004533 |
| IGLV4-69 | 5.662654 | 1.460505 | 0.012036 | 0.21465 |
| RNF219-AS1 | 5.658995 | 1.408374 | 0.000229 | 0.023223 |
| SAR1AP1 | 5.653554 | -1.71767 | 0.031524 | 0.342118 |
| LRP2 | 5.636242 | 3.909557 | 0.005389 | 0.143487 |
| AC092851.1 | 5.621208 | 0.152333 | 0.028194 | 0.327429 |
| LINC02418 | 5.602419 | 2.846337 | 0.000448 | 0.034365 |
| AL354694.1 | 5.57911 | -1.74582 | 0.006635 | 0.158657 |
| AC083843.1 | 5.571164 | -0.59565 | 0.000547 | 0.038039 |
| C3orf85 | 5.560314 | 2.54686 | 0.01795 | 0.263043 |
| POTED | 5.517088 | 4.397479 | 0.010504 | 0.199191 |
| TEX15 | 5.514539 | 3.82704 | 0.000314 | 0.027936 |
| MCIDAS | 5.503984 | -1.8428 | 0.033789 | 0.354067 |
| ARC | 5.495401 | 0.447227 | 0.000716 | 0.045447 |
| PLA2G1B | 5.489096 | -1.857 | 0.010949 | 0.203842 |
| RPS28P7 | 5.44933 | 5.363427 | 1.21E-17 | 2.08E-13 |
| AC131902.1 | 5.428234 | 4.534414 | 7.34E-05 | 0.011571 |
| AL359711.2 | 5.425723 | -1.90811 | 0.021081 | 0.285351 |
| TDRD1 | 5.413197 | 6.35437 | 2.51E-06 | 0.001231 |
| AL138974.1 | 5.400754 | -0.36801 | 0.000768 | 0.047 |
| AC026391.1 | 5.357255 | 2.759626 | 0.009153 | 0.187395 |
| AC233289.1 | 5.353604 | -1.38071 | 0.024933 | 0.30873 |
| MYO16 | 5.329374 | 6.028689 | 0.001632 | 0.073453 |
| IGSF23 | 5.312073 | -2.0499 | 0.014774 | 0.237131 |
| WNT10B | 5.299011 | -0.17334 | 0.000857 | 0.050347 |
| MAGEA8 | 5.297396 | -2.0143 | 0.014425 | 0.23427 |
| CCDC27 | 5.284695 | -2.02372 | 0.014167 | 0.232165 |
| RNU5A-8P | 5.270279 | -0.85478 | 0.013941 | 0.230836 |
| VN1R53P | 5.248749 | 3.045252 | 0.002209 | 0.088537 |
| AP001574.1 | 5.244041 | -1.38861 | 0.025733 | 0.313156 |
| CYP2C8 | 5.235277 | 3.246358 | 0.000885 | 0.051098 |
| DAB1 | 5.235184 | 7.19349 | 0.000323 | 0.028333 |
| RNU1-122P | 5.205007 | -1.39856 | 0.002492 | 0.095481 |
| AL589743.1 | 5.16621 | -0.03978 | 0.019487 | 0.273834 |
| MT1M | 5.163978 | 1.848677 | 0.006428 | 0.156706 |
| SLC7A13 | 5.154625 | -1.0473 | 0.022035 | 0.292109 |
| C1QTNF12 | 5.129126 | -1.48871 | 0.031736 | 0.343333 |
| AC110285.1 | 5.121521 | -0.17568 | 0.001671 | 0.074024 |
| LINC02197 | 5.116994 | 1.169585 | 0.001846 | 0.078296 |
| AL157387.1 | 5.077183 | 2.815455 | 0.003914 | 0.121993 |
| AC124067.2 | 5.071983 | 0.850937 | 0.006197 | 0.154483 |
| VNN1 | 5.070419 | 5.906996 | 0.000759 | 0.046951 |
| AC004009.1 | 5.068875 | -0.66763 | 0.002558 | 0.096874 |
| CLRN3 | 5.066064 | 2.786041 | 0.007633 | 0.169371 |
| MAFA | 5.055998 | -2.2052 | 0.021951 | 0.291734 |
| ALG1L10P | 5.050051 | -1.06121 | 0.003342 | 0.112578 |
| AC090772.3 | 5.042846 | -2.17735 | 0.020489 | 0.280816 |
| DOC2A | 5.005564 | 1.900486 | 1.24E-05 | 0.003297 |
| RPL7P60 | 5.004155 | -2.20619 | 0.025549 | 0.312308 |
| AL592166.1 | 4.982411 | -2.26198 | 0.034648 | 0.359025 |
| AL596442.2 | 4.975475 | -1.61766 | 0.039834 | 0.384018 |
| ANKRD30B | 4.972671 | 5.067065 | 1.07E-06 | 0.000643 |
| AC145146.1 | 4.966951 | -0.80312 | 0.00131 | 0.065139 |
| LINC01460 | 4.929953 | -2.30121 | 0.033302 | 0.351427 |
| SFTPA2 | 4.926479 | 5.151464 | 5.14E-05 | 0.008889 |
| AC025575.1 | 4.897826 | -0.02173 | 0.034871 | 0.359131 |
| AC022325.1 | 4.874628 | 2.372215 | 0.02837 | 0.327753 |
| PAPPA2 | 4.853767 | 5.531077 | 0.003294 | 0.111701 |
| TMEM161BP1 | 4.840709 | 1.567795 | 0.011619 | 0.211171 |
| TMC2 | 4.827043 | 0.291212 | 0.000488 | 0.036225 |
| SUCNR1 | 4.821949 | 5.89023 | 0.000937 | 0.052596 |
| AC009053.3 | 4.810673 | -1.72558 | 0.021079 | 0.285351 |
| AC012414.2 | 4.789433 | 3.393045 | 0.030583 | 0.338654 |
| AC061961.1 | 4.777929 | 1.754225 | 0.000158 | 0.018136 |
| SLC25A41 | 4.76873 | -2.4229 | 0.043914 | 0.400672 |
| DPYSL5 | 4.75909 | 2.234473 | 0.000449 | 0.034365 |
| RHBDL1 | 4.757777 | 0.449872 | 0.00019 | 0.020689 |
| EPHA8 | 4.754115 | -0.52693 | 0.008117 | 0.174204 |
| TMEM105 | 4.746145 | 0.966332 | 0.001075 | 0.058432 |
| AC023301.1 | 4.729891 | -0.69152 | 0.003185 | 0.109941 |
| SPATA3-AS1 | 4.69594 | -0.98514 | 0.031703 | 0.343199 |
| AC055717.2 | 4.691017 | -0.58324 | 0.025839 | 0.313948 |
| ANKRD30A | 4.686205 | 6.586079 | 0.002348 | 0.092582 |
| MT1E | 4.683311 | 4.29333 | 0.000289 | 0.026382 |
| RLN2 | 4.673192 | 7.47548 | 0.011799 | 0.212405 |
| AC013268.3 | 4.665637 | 0.124326 | 0.00304 | 0.107337 |
| B3GNT3 | 4.650343 | 2.009063 | 0.00983 | 0.192944 |
| FEM1AP2 | 4.636997 | 1.73756 | 0.045367 | 0.407082 |
| CYP4F23P | 4.632419 | 0.490845 | 0.047593 | 0.416077 |
| TNNC2 | 4.62391 | -2.4894 | 0.049426 | 0.422933 |
| PRB3 | 4.623399 | 0.106173 | 0.002345 | 0.092582 |
| TEX45 | 4.619315 | -0.87587 | 0.014492 | 0.234765 |
| RNU6-316P | 4.610664 | 0.134471 | 0.023681 | 0.30183 |
| AC117386.2 | 4.594806 | -1.07859 | 0.019808 | 0.275678 |
| AC112907.2 | 4.560503 | -1.47847 | 0.018561 | 0.267662 |
| AC010319.1 | 4.54905 | 0.07856 | 0.037392 | 0.370913 |
| LINC01623 | 4.545013 | -0.62115 | 0.004946 | 0.137306 |
| AC009133.3 | 4.538731 | 2.929758 | 0.014563 | 0.235289 |
| AL390243.1 | 4.52184 | 1.682425 | 0.006282 | 0.155234 |
| PPP1R27 | 4.516042 | -0.7413 | 0.004018 | 0.123444 |
| ISG15 | 4.509189 | 5.671816 | 1.13E-06 | 0.000671 |
| RN7SL314P | 4.49774 | 1.189696 | 0.011773 | 0.212405 |
| POTEB2 | 4.478948 | 1.642126 | 0.046826 | 0.412812 |
| ZBED1 | 4.477227 | 3.572654 | 0.000329 | 0.028576 |
| CALN1 | 4.457752 | 5.995718 | 0.005889 | 0.150505 |
| LINC01220 | 4.450644 | -1.24673 | 0.027661 | 0.324938 |
| AC040173.1 | 4.435583 | -1.24641 | 0.008304 | 0.177049 |
| AC145141.2 | 4.434222 | -0.96686 | 0.009731 | 0.192322 |
| CXADRP2 | 4.434013 | 4.799982 | 0.004015 | 0.123444 |
| MIR5691 | 4.433586 | -2.02937 | 0.033811 | 0.354183 |
| ADORA2A | 4.418507 | -1.28122 | 0.008972 | 0.185094 |
| RNVU1-6 | 4.397566 | 0.371905 | 0.002624 | 0.098923 |
| EEF1A2 | 4.384413 | 7.743071 | 7.22E-11 | 2.25E-07 |
| AC125603.2 | 4.38272 | 0.205694 | 0.010082 | 0.194673 |
| AC011586.2 | 4.371642 | -2.07893 | 0.031295 | 0.34136 |
| FEZF1-AS1 | 4.363182 | 1.007537 | 0.000398 | 0.032238 |
| C17orf99 | 4.348385 | -1.07195 | 0.044376 | 0.402709 |
| LINC00870 | 4.337465 | -0.47423 | 0.043651 | 0.399631 |
| LILRA4 | 4.31305 | 1.449566 | 0.006028 | 0.152667 |
| AC087721.1 | 4.306502 | -2.13022 | 0.031782 | 0.343495 |
| RPL23AP15 | 4.301203 | -1.075 | 0.044358 | 0.402709 |
| AMN | 4.289972 | -1.42942 | 0.02265 | 0.296149 |
| LINC02506 | 4.269129 | 2.370692 | 0.016518 | 0.251725 |
| ARID3C | 4.263642 | -0.75612 | 0.010275 | 0.197617 |
| ADAM29 | 4.261088 | 2.078078 | 0.037577 | 0.372075 |
| AL445490.1 | 4.242363 | 0.147758 | 0.015584 | 0.243752 |
| AP002981.1 | 4.241413 | -0.78914 | 0.014304 | 0.23337 |
| TERC | 4.241097 | 7.752055 | 2.87E-09 | 4.92E-06 |
| Z92544.1 | 4.197307 | -2.21276 | 0.042416 | 0.394766 |
| STK4-AS1 | 4.189991 | -1.34418 | 0.040675 | 0.387608 |
| CHN1 | 4.18473 | 7.177795 | 0.001094 | 0.058902 |
| MIR3648-2 | 4.175053 | 6.997303 | 1.24E-12 | 8.50E-09 |
| MROH6 | 4.150997 | 1.52839 | 0.000805 | 0.048522 |
| GALNT13 | 4.149472 | 4.933775 | 0.001006 | 0.055367 |
| PKD2L1 | 4.136075 | -0.46259 | 0.013962 | 0.230903 |
| VN1R42P | 4.128214 | -2.26521 | 0.047722 | 0.416462 |
| RPL21P119 | 4.12756 | 1.151704 | 0.003365 | 0.112648 |
| FOXB1 | 4.122848 | -1.30687 | 0.028472 | 0.328293 |
| MIR3687-2 | 4.101628 | 7.111596 | 8.20E-12 | 3.95E-08 |
| HOXC11 | 4.092304 | -0.496 | 0.037715 | 0.37283 |
| AC073150.1 | 4.089637 | -1.59575 | 0.029014 | 0.331081 |
| BMP5 | 4.068445 | 1.978891 | 0.003623 | 0.117186 |
| LRRC77P | 4.064655 | 0.974601 | 0.035046 | 0.360206 |
| SLCO1A2 | 4.057161 | 4.571533 | 0.003503 | 0.115282 |
| Z99755.1 | 4.04871 | -0.48014 | 0.003942 | 0.122313 |
| AC008132.1 | 4.04468 | -1.97927 | 0.043752 | 0.399687 |
| UGT2B15 | 4.043288 | 5.369861 | 0.002377 | 0.092993 |
| PLA2G10 | 4.041816 | -1.37481 | 0.030725 | 0.339346 |
| DDN | 4.008171 | -0.47039 | 0.01031 | 0.198069 |
| SHISA2 | 4.002724 | 6.037051 | 0.002957 | 0.105901 |
| CCL11 | 3.990389 | 0.115921 | 0.002102 | 0.085548 |
| NF1P4 | 3.989326 | 3.543215 | 0.029572 | 0.333558 |
| AC125611.4 | 3.987438 | -1.94654 | 0.040283 | 0.38563 |
| ALOX15 | 3.982592 | 2.026488 | 0.004227 | 0.126435 |
| ADGRG6 | 3.976425 | 6.978096 | 0.001698 | 0.074504 |
| AC068987.3 | 3.95843 | -1.02065 | 0.01884 | 0.26935 |
| POU4F1 | 3.939304 | 0.565251 | 0.003151 | 0.109723 |
| LINC00637 | 3.938039 | -1.67809 | 0.03809 | 0.374456 |
| PANX2 | 3.935534 | 4.180002 | 2.83E-06 | 0.001294 |
| RPL21P134 | 3.932744 | -0.74636 | 0.004914 | 0.136783 |
| C15orf48 | 3.918765 | 2.065824 | 0.001435 | 0.068476 |
| ZNF730 | 3.907827 | 1.954125 | 0.021994 | 0.291829 |
| KCNB2 | 3.89889 | 0.660666 | 0.02162 | 0.289184 |
| RNU2-63P | 3.889624 | 2.004937 | 0.00023 | 0.02327 |
| RPL10P15 | 3.887693 | -1.73894 | 0.032641 | 0.348198 |
| GPR35 | 3.88089 | 2.058785 | 0.003504 | 0.115282 |
| GATA1 | 3.876011 | -2.05589 | 0.048697 | 0.419721 |
| SLC22A20P | 3.858115 | -0.48021 | 0.033213 | 0.350918 |
| NPY | 3.844808 | 6.192851 | 0.009574 | 0.191135 |
| SERPINA4 | 3.83915 | 1.802957 | 0.019296 | 0.272427 |
| SNORA74D | 3.833275 | 2.218641 | 9.04E-06 | 0.002734 |
| ALB | 3.827967 | 2.868088 | 0.003386 | 0.11282 |
| CA10 | 3.820136 | 1.684872 | 0.022774 | 0.297059 |
| MYT1 | 3.808332 | 2.664035 | 0.000799 | 0.048302 |
| PRAME | 3.807329 | 1.664632 | 0.031866 | 0.343883 |
| ULBP2 | 3.797252 | 0.622823 | 0.018063 | 0.263907 |
| RALYL | 3.790456 | 1.693075 | 0.010411 | 0.198777 |
| AC108749.1 | 3.784182 | -0.73435 | 0.046205 | 0.410106 |
| RPSAP47 | 3.776252 | 3.722927 | 8.16E-06 | 0.002573 |
| AC093510.1 | 3.773643 | -1.50439 | 0.043563 | 0.399239 |
| AL662890.1 | 3.767491 | 0.860019 | 0.033046 | 0.350557 |
| HAGHL | 3.761633 | 3.494368 | 4.33E-05 | 0.008086 |
| ARMC12 | 3.75853 | 1.000266 | 0.000531 | 0.037984 |
| P2RY8 | 3.757097 | -0.87112 | 0.020553 | 0.28144 |
| LINC00383 | 3.754005 | 0.405474 | 0.030882 | 0.3401 |
| AC073551.1 | 3.735833 | -0.19676 | 0.003245 | 0.110682 |
| KRT8P4 | 3.721048 | -1.54776 | 0.047522 | 0.415773 |
| MTFR2 | 3.709782 | 0.595669 | 0.006159 | 0.154356 |
| METTL7B | 3.701975 | 1.009839 | 0.012601 | 0.218706 |
| ZNF350-AS1 | 3.694466 | 5.741143 | 0.00923 | 0.187736 |
| AC013476.1 | 3.691581 | -1.61061 | 0.031097 | 0.340931 |
| SULT1B1 | 3.683207 | 2.444366 | 0.007041 | 0.16254 |
| AL512310.3 | 3.671672 | 3.909226 | 0.049327 | 0.422819 |
| ABCC8 | 3.671421 | 2.938108 | 0.026862 | 0.320601 |
| KCNJ3 | 3.671029 | 5.498515 | 7.27E-06 | 0.002431 |
| AC140479.2 | 3.667544 | 1.507922 | 0.001655 | 0.07393 |
| AC006033.1 | 3.666413 | -0.06165 | 0.003578 | 0.116716 |
| NTN5 | 3.651341 | 0.505816 | 0.017268 | 0.258117 |
| SYT4 | 3.651286 | 7.138735 | 0.018568 | 0.267662 |
| AC016205.1 | 3.648969 | -0.3017 | 0.003526 | 0.11568 |
| KIF1A | 3.619429 | 6.989748 | 0.000542 | 0.03802 |
| MATK | 3.59584 | 2.819727 | 0.000798 | 0.048302 |
| HMX1 | 3.583793 | 0.516834 | 0.03605 | 0.365594 |
| BST2 | 3.572651 | 6.558209 | 0.003213 | 0.110354 |
| PCSK1N | 3.568567 | 1.01001 | 0.000487 | 0.036225 |
| KIFC2 | 3.567321 | 4.600853 | 3.62E-08 | 4.44E-05 |
| FLJ13224 | 3.567051 | 1.256258 | 0.005326 | 0.142483 |
| TNNT1 | 3.553241 | -0.45658 | 0.019831 | 0.275783 |
| RAC3 | 3.549503 | 3.758865 | 8.96E-05 | 0.013317 |
| ANKRD20A5P | 3.546806 | 5.254498 | 0.0034 | 0.113066 |
| C9orf139 | 3.544766 | -0.20536 | 0.040467 | 0.386329 |
| GP6 | 3.534975 | -0.25987 | 0.006804 | 0.160305 |
| OR51E1 | 3.5331 | 7.200488 | 0.009564 | 0.191135 |
| COL9A3 | 3.53007 | 1.717853 | 0.003376 | 0.11282 |
| KL | 3.518372 | 4.506731 | 0.000102 | 0.014478 |
| OPRK1 | 3.511516 | 5.687814 | 0.000119 | 0.015353 |
| MTNR1A | 3.503871 | 0.26815 | 0.035202 | 0.36148 |
| SERPINA7 | 3.499863 | 0.985932 | 0.037102 | 0.370038 |
| HIST3H2BB | 3.496388 | 2.260888 | 0.000133 | 0.016123 |
| ITGAD | 3.495295 | 1.533864 | 0.00109 | 0.058752 |
| PALM3 | 3.48393 | 1.567463 | 0.025854 | 0.313958 |
| PIMREG | 3.471654 | 1.442763 | 0.00024 | 0.024077 |
| POTEC | 3.459042 | 3.722473 | 0.007402 | 0.166175 |
| SLC39A4 | 3.456082 | 3.231046 | 0.000118 | 0.015258 |
| LINC01136 | 3.453524 | 0.883151 | 0.031066 | 0.340878 |
| RNU6-8 | 3.447621 | -1.5826 | 0.04226 | 0.393919 |
| BOLA2B | 3.447541 | -0.05645 | 0.001519 | 0.070871 |
| GPT | 3.442082 | 2.163789 | 4.76E-05 | 0.008479 |
| AC131097.3 | 3.437568 | -1.11512 | 0.021447 | 0.287769 |
| MYB | 3.433999 | 5.058028 | 0.000235 | 0.023662 |
| CES3 | 3.430352 | 1.494018 | 0.030279 | 0.337349 |
| AC125618.1 | 3.425066 | -0.08059 | 0.019867 | 0.276107 |
| SCUBE2 | 3.422115 | 6.282746 | 0.00139 | 0.067448 |
| MMEL1 | 3.415834 | -0.83241 | 0.03387 | 0.354553 |
| ELOVL4 | 3.410834 | 4.645859 | 0.008894 | 0.18403 |
| F2RL3 | 3.40132 | 0.332597 | 0.013058 | 0.223646 |
| NAPRT | 3.399674 | 2.506872 | 0.000129 | 0.016011 |
| DDT | 3.387136 | 1.884137 | 0.001299 | 0.064939 |
| SIAH2-AS1 | 3.387058 | -0.65415 | 0.02094 | 0.284407 |
| ASB9 | 3.383534 | 4.613911 | 0.010909 | 0.20331 |
| RANBP20P | 3.3791 | -1.12698 | 0.037256 | 0.370677 |
| RNF223 | 3.361802 | -0.99225 | 0.041425 | 0.390049 |
| MYH16 | 3.35847 | 0.553817 | 0.007725 | 0.170649 |
| KLF2P1 | 3.357321 | 2.355647 | 0.001562 | 0.071971 |
| AC015983.2 | 3.349984 | -0.03245 | 0.008807 | 0.183581 |
| SLC26A3 | 3.341075 | 5.655495 | 0.036353 | 0.367018 |
| TNNT2 | 3.335198 | 0.937264 | 0.007703 | 0.170492 |
| SLC10A5 | 3.329604 | 5.627161 | 5.13E-07 | 0.000415 |
| AC021683.2 | 3.31965 | 3.525156 | 0.036216 | 0.366191 |
| ADGRB1 | 3.303522 | 2.987849 | 0.000386 | 0.031607 |
| FBLL1 | 3.302841 | -0.14207 | 0.018123 | 0.264226 |
| PRR7 | 3.302198 | 1.448731 | 0.000139 | 0.016719 |
| SNORA84 | 3.30071 | -0.86239 | 0.029722 | 0.333985 |
| NME2P1 | 3.298814 | 0.0403 | 0.012337 | 0.216717 |
| SSC4D | 3.298409 | -0.72045 | 0.026553 | 0.318458 |
| AP002992.1 | 3.294835 | -1.12477 | 0.03179 | 0.343495 |
| EIF4EBP1 | 3.290718 | 4.294721 | 0.000645 | 0.042422 |
| ULBP1 | 3.277981 | 0.212333 | 0.007975 | 0.173372 |
| GINS4 | 3.276759 | 2.40549 | 0.001574 | 0.072201 |
| CLEC18B | 3.266885 | -0.22405 | 0.027443 | 0.323711 |
| SGK2 | 3.253765 | 4.252367 | 0.006229 | 0.154706 |
| SLC6A11 | 3.250931 | 3.937585 | 2.89E-06 | 0.001303 |
| RN7SKP127 | 3.248376 | -0.0271 | 0.045141 | 0.406287 |
| VGF | 3.242264 | 2.262413 | 0.000546 | 0.038039 |
| AC092894.1 | 3.239308 | 1.538095 | 0.004839 | 0.135712 |
| HPGD | 3.237532 | 6.033541 | 0.008099 | 0.17417 |
| FAM110B | 3.236781 | 6.940706 | 2.92E-06 | 0.001303 |
| IFI27 | 3.232662 | 5.069599 | 0.002657 | 0.099612 |
| GOT2P2 | 3.225695 | -0.53356 | 0.007166 | 0.163776 |
| RNU1-120P | 3.22198 | 0.570454 | 0.001245 | 0.063203 |
| PRELID3A | 3.213072 | 3.025092 | 0.00017 | 0.019031 |
| HTR1F | 3.211166 | 3.14376 | 0.049048 | 0.421619 |
| AC138969.1 | 3.198753 | 1.443801 | 0.002388 | 0.093111 |
| LINC02469 | 3.195154 | 1.440596 | 0.005158 | 0.139887 |
| AC022113.2 | 3.191435 | 0.294861 | 0.003228 | 0.110539 |
| CDCA5 | 3.190187 | 3.346104 | 0.000115 | 0.015162 |
| FBN3 | 3.186784 | 0.285058 | 0.006355 | 0.156193 |
| GRB14 | 3.183036 | 2.746027 | 0.010393 | 0.198761 |
| PLCXD1 | 3.182952 | 0.476338 | 0.007155 | 0.163776 |
| SCNN1B | 3.170109 | 2.925704 | 0.002713 | 0.100592 |
| HES6 | 3.168298 | 1.267157 | 0.01162 | 0.211171 |
| DLX2 | 3.163341 | 0.234992 | 0.021079 | 0.285351 |
| HIST2H3C | 3.147211 | -1.13644 | 0.045629 | 0.408444 |
| MT1G | 3.137878 | 1.996629 | 0.035722 | 0.363815 |
| AC007952.4 | 3.135753 | 3.351566 | 0.000442 | 0.034109 |
| CHRNA7 | 3.123822 | 2.716184 | 0.005761 | 0.148467 |
| AC024940.2 | 3.117397 | 0.473863 | 0.002734 | 0.10065 |
| PFN1P8 | 3.110236 | 0.213054 | 0.020488 | 0.280816 |
| AL035458.2 | 3.094043 | -1.28747 | 0.043826 | 0.400154 |
| MGC27382 | 3.087166 | 1.263493 | 0.030667 | 0.339144 |
| AC012213.2 | 3.063429 | -1.37668 | 0.033945 | 0.354945 |
| PRR22 | 3.059457 | -0.75461 | 0.028761 | 0.330292 |
| RHPN1 | 3.054657 | 4.12123 | 3.02E-06 | 0.001329 |
| AC012640.2 | 3.045841 | 0.341203 | 0.005648 | 0.14687 |
| ADM | 3.039299 | 3.208205 | 0.003521 | 0.115629 |
| MAPK15 | 3.033969 | 1.730073 | 0.009035 | 0.185731 |
| AL391987.2 | 3.03276 | -0.05229 | 0.025125 | 0.309991 |
| CCDC78 | 3.02844 | 3.103875 | 3.93E-05 | 0.007546 |
| DEGS2 | 3.024893 | 2.629155 | 9.62E-05 | 0.013878 |
| CYSRT1 | 3.019289 | 0.07452 | 0.027415 | 0.323601 |
| AC022113.1 | 2.993675 | 0.568579 | 0.023524 | 0.301106 |
| SLC39A11 | 2.978519 | 5.465473 | 2.38E-05 | 0.005446 |
| RHPN1-AS1 | 2.977415 | -0.43792 | 0.025506 | 0.312219 |
| TUFMP1 | 2.967199 | -0.23481 | 0.027827 | 0.326109 |
| SLC25A6 | 2.961411 | 3.756319 | 0.000258 | 0.024875 |
| SNORD93 | 2.959511 | 0.090293 | 0.027848 | 0.326245 |
| AJM1 | 2.952732 | 1.121285 | 0.004546 | 0.13143 |
| KIFC1 | 2.952439 | 3.671882 | 5.62E-05 | 0.009506 |
| KCNG3 | 2.951463 | 2.594318 | 0.004116 | 0.124575 |
| CPNE7 | 2.948315 | 4.674128 | 2.51E-05 | 0.005622 |
| AC131902.2 | 2.933278 | 1.393343 | 0.030782 | 0.339652 |
| FAM72B | 2.931076 | 0.710502 | 0.015855 | 0.2459 |
| LINC01977 | 2.927595 | 1.076085 | 0.01294 | 0.222602 |
| OVOL1-AS1 | 2.925756 | -0.5701 | 0.031993 | 0.344504 |
| DUX4L50 | 2.919449 | 0.554798 | 0.014762 | 0.237131 |
| AKR1C1 | 2.917187 | 4.617257 | 0.010331 | 0.198223 |
| RHOU | 2.916565 | 8.588052 | 0.000153 | 0.01779 |
| TFCP2L1 | 2.910811 | 5.589076 | 0.001162 | 0.061101 |
| SLCO1B3 | 2.906829 | 0.511499 | 0.039592 | 0.382541 |
| RECQL4 | 2.906286 | 3.800467 | 9.28E-06 | 0.002734 |
| AKR1C3 | 2.905104 | 6.71575 | 2.76E-05 | 0.005928 |
| VWA2 | 2.900021 | 3.721984 | 0.000559 | 0.038403 |
| ISYNA1 | 2.899568 | 5.050191 | 0.000679 | 0.044054 |
| PLGRKT | 2.8945 | 4.863468 | 0.004541 | 0.131422 |
| CASC11 | 2.892361 | 0.006581 | 0.024942 | 0.308732 |
| MYO7A | 2.882856 | 3.794728 | 0.003062 | 0.107664 |
| MIR663AHG | 2.876859 | 7.296301 | 2.04E-07 | 0.000189 |
| CD274 | 2.876411 | 4.009978 | 0.011964 | 0.213708 |
| TM7SF2 | 2.874098 | 5.786456 | 5.69E-05 | 0.009569 |
| PDIA2 | 2.87324 | 2.212329 | 0.025193 | 0.310391 |
| SLC17A4 | 2.866335 | 4.462409 | 0.013328 | 0.225521 |
| KCNQ2 | 2.866142 | 3.629 | 0.000927 | 0.052282 |
| AC104350.1 | 2.860565 | 3.097802 | 0.002078 | 0.08488 |
| LINC00412 | 2.854192 | 0.202102 | 0.005254 | 0.141733 |
| IFI6 | 2.84996 | 6.342046 | 0.001288 | 0.064565 |
| ATP6V1C2 | 2.847001 | 3.103813 | 0.006715 | 0.159463 |
| PYCR3 | 2.831739 | 3.794498 | 0.000126 | 0.015754 |
| BMP6 | 2.82617 | 4.384965 | 0.00484 | 0.135712 |
| NAT8L | 2.825555 | 1.636848 | 0.006213 | 0.154647 |
| RPL7AP66 | 2.824041 | 1.261065 | 0.003189 | 0.109941 |
| KLF2P3 | 2.810766 | 2.301149 | 0.006775 | 0.159859 |
| PMCHL2 | 2.807507 | 1.057237 | 0.023285 | 0.300391 |
| AL163051.1 | 2.8075 | -0.2112 | 0.039981 | 0.384868 |
| LARGE2 | 2.802274 | 4.678268 | 3.32E-05 | 0.006607 |
| AC104667.2 | 2.800799 | 0.250026 | 0.03267 | 0.348198 |
| CRACR2A | 2.799732 | 4.859161 | 0.000144 | 0.017345 |
| AC138904.1 | 2.795862 | 0.799632 | 0.04309 | 0.398085 |
| AC078950.1 | 2.793653 | 1.789916 | 0.013804 | 0.229786 |
| TUBB4A | 2.786547 | 1.135251 | 0.024576 | 0.306333 |
| CELF5 | 2.783567 | 0.455046 | 0.041212 | 0.38947 |
| RPL21P75 | 2.782152 | 3.524402 | 0.005611 | 0.146453 |
| RMI2 | 2.777512 | 2.53425 | 0.000993 | 0.054812 |
| RNU5E-1 | 2.776804 | 3.85759 | 0.002203 | 0.088406 |
| EFNA3 | 2.775018 | 0.474439 | 0.024579 | 0.306333 |
| NPIPB6 | 2.773272 | -0.57002 | 0.027354 | 0.32311 |
| LINC01252 | 2.762409 | 2.45753 | 0.008646 | 0.181754 |
| MYC | 2.755161 | 7.757962 | 4.53E-05 | 0.008193 |
| AC004969.1 | 2.747039 | -0.60256 | 0.049588 | 0.423809 |
| PPP2R3B | 2.737875 | 0.757839 | 0.007113 | 0.162997 |
| OXTR | 2.735885 | 3.488747 | 0.006651 | 0.158657 |
| CLCN2 | 2.730589 | 3.186298 | 0.000925 | 0.052282 |
| HIST2H3D | 2.722782 | 0.552446 | 0.015628 | 0.244006 |
| AC112777.1 | 2.714933 | 0.610448 | 0.012677 | 0.219302 |
| AC253536.6 | 2.714355 | 1.394885 | 0.003853 | 0.12076 |
| SNORD14E | 2.706855 | 2.052989 | 0.002061 | 0.084269 |
| OVOL1 | 2.706335 | 2.020902 | 0.009675 | 0.19154 |
| RNVU1-15 | 2.69592 | 0.416872 | 0.006883 | 0.160585 |
| RN7SL559P | 2.691336 | 0.243653 | 0.046597 | 0.411986 |
| SNORA74A | 2.684358 | 7.904641 | 1.41E-05 | 0.003578 |
| HIST1H2BL | 2.681225 | 3.609267 | 0.000108 | 0.014834 |
| YDJC | 2.67856 | 2.683119 | 0.000362 | 0.030262 |
| MIR4477B | 2.673454 | 0.849886 | 0.019971 | 0.276608 |
| ECE2 | 2.669385 | 2.138939 | 0.001419 | 0.068264 |
| PEG10 | 2.668949 | 6.017819 | 0.002277 | 0.090743 |
| SAXO1 | 2.666584 | -0.48446 | 0.041598 | 0.390821 |
| GLYATL2 | 2.663214 | 3.010853 | 0.018112 | 0.264226 |
| IQANK1 | 2.657533 | 6.649768 | 4.77E-07 | 0.0004 |
| KIF4A | 2.654682 | 4.633076 | 0.000195 | 0.021141 |
| TIGD5 | 2.649116 | 2.702084 | 0.000558 | 0.038403 |
| AL139412.1 | 2.645808 | 0.162921 | 0.030252 | 0.337163 |
| SMPD5 | 2.643284 | 0.488781 | 0.042792 | 0.396941 |
| ARHGAP5-AS1 | 2.64318 | 1.546503 | 0.006139 | 0.154356 |
| LINC01687 | 2.639855 | 4.410622 | 0.003559 | 0.116542 |
| FGF7P3 | 2.639036 | 0.749511 | 0.027101 | 0.321972 |
| LINC01876 | 2.638841 | 2.710829 | 0.00031 | 0.027744 |
| B4GALNT1 | 2.638328 | 2.109379 | 0.008755 | 0.183253 |
| AC079250.1 | 2.635851 | 0.331896 | 0.023616 | 0.301561 |
| MAFG-DT | 2.635518 | 0.988334 | 0.005318 | 0.142483 |
| RNU5F-1 | 2.633544 | 1.552219 | 0.015932 | 0.246503 |
| HIST1H3D | 2.633295 | 5.124228 | 2.84E-05 | 0.006016 |
| TTK | 2.630119 | 5.22955 | 5.71E-05 | 0.009569 |
| E2F1 | 2.629284 | 2.471178 | 0.000556 | 0.038403 |
| HOGA1 | 2.628816 | 0.380288 | 0.04437 | 0.402709 |
| CBX2 | 2.628533 | 4.407454 | 0.000307 | 0.02749 |
| RTTN | 2.626514 | 6.804557 | 0.002997 | 0.106682 |
| ABCA12 | 2.624384 | 1.877653 | 0.021574 | 0.288859 |
| MT2A | 2.623108 | 4.191415 | 0.001613 | 0.07317 |
| UNC5A | 2.621 | 0.376324 | 0.025564 | 0.312382 |
| AC112907.3 | 2.618704 | 1.525631 | 0.016848 | 0.255304 |
| EPHB1 | 2.616426 | 2.145243 | 0.023003 | 0.299084 |
| AL157823.2 | 2.614835 | 0.029852 | 0.046423 | 0.411508 |
| MDH1B | 2.610135 | 3.435253 | 0.002627 | 0.098923 |
| SCN2A | 2.598814 | 3.191276 | 0.000577 | 0.039223 |
| MCM2 | 2.598085 | 5.09866 | 5.31E-06 | 0.001911 |
| FN1 | 2.59159 | 11.91881 | 0.016207 | 0.249082 |
| AC139149.1 | 2.587247 | 1.178847 | 0.002916 | 0.104652 |
| CELSR3 | 2.586656 | 4.227756 | 0.000202 | 0.021602 |
| ARHGDIG | 2.585623 | 2.192581 | 0.003103 | 0.108752 |
| COL2A1 | 2.582215 | 0.39011 | 0.010751 | 0.201568 |
| MAPK8IP2 | 2.580608 | 4.025338 | 0.000165 | 0.018638 |
| HIST1H2AI | 2.580075 | 3.814788 | 0.000117 | 0.015258 |
| LY6E | 2.575747 | 6.220887 | 0.000247 | 0.024307 |
| TLL2 | 2.571893 | 2.379321 | 0.017235 | 0.257899 |
| TONSL | 2.568418 | 3.330537 | 0.000268 | 0.025714 |
| BX322639.1 | 2.568343 | 3.674829 | 8.59E-05 | 0.012881 |
| CA12 | 2.565454 | 3.118992 | 0.009522 | 0.190815 |
| AC244131.1 | 2.562684 | 1.595134 | 0.015315 | 0.241758 |
| SLC34A3 | 2.561589 | 0.836971 | 0.048643 | 0.419715 |
| PRRT2 | 2.559241 | 4.665298 | 0.009454 | 0.189906 |
| HIST1H2AG | 2.558128 | 4.454874 | 0.001246 | 0.063203 |
| SUMO2P19 | 2.557844 | -0.07211 | 0.040431 | 0.386197 |
| TESMIN | 2.556642 | 4.171411 | 0.000174 | 0.019347 |
| UBAC2-AS1 | 2.55316 | 1.182013 | 0.007866 | 0.171951 |
| RPL26P26 | 2.550236 | 2.872085 | 0.00662 | 0.158657 |
| RPS2P55 | 2.541179 | 0.08441 | 0.015628 | 0.244006 |
| KLKP1 | 2.540829 | 2.999798 | 0.001303 | 0.064962 |
| PPP1R14B | 2.534054 | 5.242195 | 0.000118 | 0.015292 |
| C16orf54 | 2.525418 | 2.580235 | 0.01309 | 0.223646 |
| LINC00992 | 2.516158 | 2.399578 | 0.013355 | 0.225549 |
| IFIT1 | 2.516012 | 6.376651 | 0.010801 | 0.202003 |
| APOC1 | 2.513371 | 5.65575 | 0.000466 | 0.035112 |
| LINC01399 | 2.506345 | 1.34761 | 0.018413 | 0.266979 |
| UBE2MP1 | 2.503474 | -0.40877 | 0.041811 | 0.392077 |
| GLI4 | 2.503454 | 2.586698 | 0.000962 | 0.053714 |
| EME1 | 2.501679 | 1.266125 | 0.008778 | 0.183412 |
| AC011284.1 | 2.497283 | 2.102526 | 0.031845 | 0.343867 |
| LAGE3 | 2.495257 | 1.671459 | 0.002359 | 0.092805 |
| UBR5-AS1 | 2.492605 | 3.755778 | 0.000305 | 0.027462 |
| RAMP1 | 2.490072 | 5.101099 | 8.56E-05 | 0.012881 |
| MDK | 2.490054 | 6.597959 | 0.001367 | 0.066769 |
| AL139100.1 | 2.48894 | 1.691829 | 0.004857 | 0.135865 |
| SLC25A10 | 2.48883 | 2.079474 | 0.004487 | 0.13112 |
| CBS | 2.485855 | 6.531409 | 0.000343 | 0.029233 |
| ZNF385B | 2.484678 | 6.378116 | 0.017417 | 0.259234 |
| BBC3 | 2.480657 | 1.048021 | 0.007959 | 0.173273 |
| UBE2C | 2.480224 | 3.049279 | 0.000975 | 0.054087 |
| DDTL | 2.479204 | 1.83833 | 0.004173 | 0.125511 |
| RNU1-106P | 2.471109 | 0.59113 | 0.017333 | 0.258746 |
| ADCK5 | 2.469147 | 2.954048 | 0.003493 | 0.115255 |
| NR3C2 | 2.468294 | 7.319794 | 0.01629 | 0.250027 |
| NGEF | 2.465013 | 0.432903 | 0.025735 | 0.313156 |
| FSCN2 | 2.459632 | 1.977878 | 0.002548 | 0.096699 |
| MT-RNR2 | 2.458694 | 5.739731 | 0.001747 | 0.075491 |
| MEX3A | 2.45305 | 5.136263 | 0.000656 | 0.043029 |
| AL139095.2 | 2.442775 | 1.830253 | 0.009319 | 0.187892 |
| TFR2 | 2.432807 | 0.941386 | 0.005957 | 0.151464 |
| GPM6A | 2.42935 | 3.176226 | 0.037293 | 0.370677 |
| AC008429.2 | 2.427574 | 0.465469 | 0.016362 | 0.250559 |
| C8orf82 | 2.425237 | 4.453843 | 0.000109 | 0.014841 |
| HIST1H1B | 2.424537 | 5.899733 | 0.010566 | 0.199443 |
| FAM53A | 2.417892 | 0.823214 | 0.008927 | 0.184267 |
| PRKAG2-AS1 | 2.417699 | 3.903589 | 0.001068 | 0.058304 |
| NEIL3 | 2.416057 | 1.333011 | 0.011794 | 0.212405 |
| APOE | 2.415671 | 6.702683 | 0.000394 | 0.032188 |
| ZIC5 | 2.413749 | 2.406487 | 0.035599 | 0.363262 |
| MFSD3 | 2.410343 | 3.399961 | 0.000348 | 0.029489 |
| RNFT2 | 2.404065 | 3.049985 | 0.003108 | 0.108752 |
| LRP8 | 2.398622 | 3.404053 | 0.003187 | 0.109941 |
| FAM106A | 2.398561 | 2.754035 | 0.001471 | 0.069516 |
| PBK | 2.397916 | 3.136627 | 0.001217 | 0.062432 |
| FAM86C2P | 2.393448 | 2.093492 | 0.006362 | 0.156193 |
| HNRNPA1P10 | 2.390249 | 2.731734 | 0.00641 | 0.156388 |
| ERG | 2.383721 | 5.898757 | 0.021233 | 0.286235 |
| TRIP13 | 2.381172 | 3.290445 | 0.001813 | 0.077495 |
| HSD17B8 | 2.36525 | 2.960675 | 0.004603 | 0.132307 |
| CKS2 | 2.364386 | 3.833754 | 0.000483 | 0.036086 |
| PKMYT1 | 2.363601 | 1.431274 | 0.00597 | 0.151622 |
| MT-RNR1 | 2.362349 | 5.059753 | 0.001635 | 0.0735 |
| MYBL2 | 2.361423 | 3.67321 | 0.000931 | 0.052375 |
| ABHD12 | 2.358832 | 6.508187 | 0.00118 | 0.061492 |
| ESCO2 | 2.358547 | 3.426426 | 0.001418 | 0.068264 |
| TST | 2.3562 | 4.092549 | 0.000154 | 0.01779 |
| CFAP161 | 2.355723 | 1.474476 | 0.009893 | 0.19351 |
| CHAC2 | 2.350347 | 1.409959 | 0.006529 | 0.157274 |
| AC096533.1 | 2.349462 | 3.068591 | 0.041979 | 0.392769 |
| GRIN1 | 2.347221 | 2.942447 | 0.017478 | 0.259548 |
| TMEM205 | 2.345124 | 2.523247 | 0.003749 | 0.119322 |
| ZP3 | 2.340233 | 1.859302 | 0.011861 | 0.213064 |
| ESPL1 | 2.330725 | 3.377553 | 0.001109 | 0.059496 |
| PSORS1C3 | 2.328971 | 1.201881 | 0.023263 | 0.300391 |
| TENM2 | 2.328858 | 3.875087 | 0.022005 | 0.291838 |
| CMBL | 2.326367 | 5.342362 | 4.03E-05 | 0.007607 |
| NOVA2 | 2.324996 | 2.435698 | 0.014252 | 0.233118 |
| CILP2 | 2.322413 | -0.01863 | 0.023017 | 0.299099 |
| C6orf118 | 2.321788 | 0.616555 | 0.033194 | 0.35087 |
| EXOSC5 | 2.321445 | 3.13241 | 0.000779 | 0.04752 |
| OAS1 | 2.319048 | 5.256691 | 0.008328 | 0.17723 |
| ISX | 2.31901 | 2.796641 | 0.017733 | 0.261416 |
| DGAT2 | 2.316853 | 2.961399 | 0.019349 | 0.272716 |
| HIST1H2BJ | 2.315936 | 3.928759 | 0.008218 | 0.17576 |
| MALL | 2.312251 | 2.022139 | 0.019873 | 0.276107 |
| RAB39A | 2.311531 | 1.979564 | 0.009655 | 0.191514 |
| FER1L4 | 2.311145 | 2.20699 | 0.025033 | 0.309191 |
| MRPL12 | 2.30778 | -0.48642 | 0.037417 | 0.370913 |
| CA13 | 2.306418 | 4.328824 | 0.002746 | 0.100669 |
| FAM86HP | 2.291148 | 1.697763 | 0.005096 | 0.139115 |
| SNHG26 | 2.290107 | 3.24712 | 0.007522 | 0.16778 |
| TMEM38A | 2.286885 | 3.522381 | 0.00167 | 0.074024 |
| MT-TC | 2.285807 | 0.734963 | 0.025546 | 0.312308 |
| PPIAP2 | 2.285758 | 0.22358 | 0.046857 | 0.412812 |
| ESRRA | 2.28243 | 3.885696 | 0.001716 | 0.074807 |
| HIST1H4J | 2.280576 | 1.523362 | 0.018013 | 0.263517 |
| DDX11-AS1 | 2.280069 | 1.222676 | 0.010535 | 0.19938 |
| H2AFX | 2.274086 | 3.757078 | 0.00034 | 0.02912 |
| E2F2 | 2.274048 | 2.035463 | 0.00392 | 0.121993 |
| AATK | 2.268837 | 2.523612 | 0.003204 | 0.110181 |
| JOSD2 | 2.264566 | 1.728776 | 0.007483 | 0.167397 |
| SDS | 2.26141 | 3.157436 | 0.016903 | 0.255413 |
| THEM6 | 2.261305 | 4.712812 | 0.000415 | 0.032712 |
| FBXL6 | 2.259006 | 1.953261 | 0.015075 | 0.239509 |
| C2CD4C | 2.25376 | 0.177014 | 0.019561 | 0.274063 |
| PRSS16 | 2.253264 | 2.545852 | 0.006401 | 0.156384 |
| AC027698.1 | 2.25263 | 3.143759 | 0.008353 | 0.17763 |
| RHOT1P1 | 2.242441 | 2.019471 | 0.017168 | 0.257633 |
| AC087620.1 | 2.242022 | 1.655052 | 0.017239 | 0.257899 |
| MKI67 | 2.240932 | 6.920891 | 3.25E-05 | 0.006596 |
| RPUSD1 | 2.240082 | 2.852316 | 0.002993 | 0.106647 |
| ADCY1 | 2.239662 | 4.851989 | 0.011886 | 0.213108 |
| AC010306.1 | 2.238807 | 1.510277 | 0.047878 | 0.417144 |
| AC087343.1 | 2.237714 | 1.140382 | 0.038762 | 0.377485 |
| ACADS | 2.235093 | 3.310527 | 0.004856 | 0.135865 |
| RPL21 | 2.229834 | 9.978989 | 0.006965 | 0.161548 |
| GLYCTK | 2.228934 | 3.627459 | 0.025517 | 0.312219 |
| AC005682.1 | 2.228846 | 1.499808 | 0.041887 | 0.392465 |
| RPL8 | 2.224875 | 9.896258 | 0.000254 | 0.024647 |
| CEP55 | 2.22282 | 3.28519 | 0.001526 | 0.071 |
| AURKB | 2.214158 | 2.166304 | 0.008008 | 0.173474 |
| PPM1E | 2.213192 | 4.730379 | 0.023902 | 0.303289 |
| FAM110C | 2.210051 | 4.206698 | 0.022531 | 0.296001 |
| SHCBP1 | 2.208614 | 2.832512 | 0.006175 | 0.154435 |
| SLC25A36P1 | 2.202651 | 0.706855 | 0.045706 | 0.408734 |
| CDC20 | 2.201025 | 2.870354 | 0.00202 | 0.083412 |
| SNORD3B-1 | 2.198976 | 3.821889 | 0.005168 | 0.139887 |
| HNRNPCP7 | 2.197066 | 3.298189 | 0.013866 | 0.230292 |
| EXOSC4 | 2.196235 | 3.201808 | 0.004726 | 0.133949 |
| NDUFB7 | 2.195684 | 4.217026 | 0.000535 | 0.037984 |
| TPX2 | 2.193933 | 5.452681 | 0.000251 | 0.024536 |
| CYC1 | 2.18991 | 5.934654 | 0.000132 | 0.016077 |
| ZNF696 | 2.187828 | 3.512769 | 0.001557 | 0.071896 |
| ANAPC11 | 2.185669 | 4.022592 | 0.001131 | 0.06012 |
| LAGE3P1 | 2.183194 | 0.302033 | 0.022184 | 0.293638 |
| BRCA2 | 2.180441 | 5.25613 | 0.001322 | 0.065542 |
| AFDN-DT | 2.179376 | 2.528946 | 0.012308 | 0.216388 |
| CDCA7 | 2.177261 | 3.492082 | 0.019925 | 0.276298 |
| ADAMTS13 | 2.17722 | 2.188087 | 0.011192 | 0.206789 |
| MCM4 | 2.175841 | 6.530383 | 0.000329 | 0.028576 |
| SCRIB | 2.174825 | 5.779764 | 5.91E-05 | 0.009754 |
| CTXN1 | 2.172052 | 1.154395 | 0.041522 | 0.390532 |
| RNU4-25P | 2.171762 | 0.729168 | 0.020789 | 0.283343 |
| ACBD7 | 2.17153 | 1.28761 | 0.022337 | 0.294638 |
| RPSAP19 | 2.168646 | 3.506729 | 0.00162 | 0.073334 |
| CCSAP | 2.168141 | 6.34466 | 0.005535 | 0.145325 |
| PRKCA | 2.166256 | 7.741948 | 0.005519 | 0.145325 |
| EEF1D | 2.154266 | 7.791888 | 0.000365 | 0.030411 |
| ABCC5-AS1 | 2.153985 | -0.01857 | 0.033505 | 0.352439 |
| PPP1R35 | 2.147972 | 1.764323 | 0.01039 | 0.198761 |
| CAPN13 | 2.145526 | 2.538499 | 0.027591 | 0.324678 |
| CGREF1 | 2.144726 | 4.238338 | 0.003957 | 0.122456 |
| SLC29A2 | 2.144611 | 3.832888 | 0.00918 | 0.187578 |
| ACKR3 | 2.144119 | 3.571064 | 0.004032 | 0.123444 |
| PKHD1 | 2.142918 | 5.487954 | 0.039897 | 0.384405 |
| LINC01123 | 2.142571 | 3.081981 | 0.009761 | 0.192398 |
| MINCR | 2.140094 | 2.084951 | 0.012209 | 0.215605 |
| UNC93B1 | 2.138035 | 4.349197 | 0.001153 | 0.060839 |
| RN7SKP203 | 2.135635 | 9.75907 | 0.000102 | 0.014478 |
| ISOC2 | 2.13395 | 4.300162 | 0.000405 | 0.032325 |
| SDC1 | 2.133359 | 5.941044 | 0.004247 | 0.126634 |
| LAPTM4B | 2.132812 | 6.536707 | 0.02457 | 0.306333 |
| MAST1 | 2.123981 | 0.634366 | 0.024243 | 0.304917 |
| CD320 | 2.123883 | 4.009126 | 0.00143 | 0.068436 |
| VPS9D1-AS1 | 2.123802 | 2.593084 | 0.01131 | 0.207643 |
| FAM86JP | 2.123627 | 0.144011 | 0.030184 | 0.336714 |
| PBLD | 2.123145 | 6.103136 | 0.004866 | 0.135986 |
| ZBTB10 | 2.122564 | 9.720176 | 0.000745 | 0.04642 |
| RPL18AP3 | 2.121906 | 5.090492 | 0.002298 | 0.091483 |
| MAPK12 | 2.121269 | 4.961117 | 9.34E-05 | 0.013603 |
| KNDC1 | 2.121141 | 1.505717 | 0.024388 | 0.305843 |
| IER2 | 2.117725 | 4.796141 | 0.001995 | 0.082761 |
| COQ8A | 2.116993 | 5.759696 | 0.000789 | 0.047989 |
| INTS4P1 | 2.108243 | 0.909488 | 0.020837 | 0.283794 |
| COL26A1 | 2.106707 | 2.774205 | 0.031453 | 0.342118 |
| NCAM2 | 2.10643 | 4.96957 | 0.017589 | 0.260411 |
| EEF1DP1 | 2.103546 | 0.774642 | 0.041244 | 0.38947 |
| WNK4 | 2.10306 | 3.828582 | 0.017459 | 0.259384 |
| SAPCD2 | 2.101681 | 3.790354 | 0.001052 | 0.057535 |
| CRACR2B | 2.100616 | 4.975827 | 0.001782 | 0.07662 |
| EEF1DP3 | 2.10042 | 2.24312 | 0.005848 | 0.149675 |
| JMJD4 | 2.099385 | 2.822296 | 0.009428 | 0.189608 |
| ANKRD13B | 2.098558 | 3.123378 | 0.016826 | 0.255163 |
| CDC25C | 2.097976 | 1.992565 | 0.012308 | 0.216388 |
| FKBP10 | 2.093431 | 6.22623 | 0.001072 | 0.058371 |
| FAM171B | 2.092545 | 5.976647 | 0.03487 | 0.359131 |
| CILP | 2.087274 | 1.85727 | 0.02403 | 0.303586 |
| TRIB1 | 2.087067 | 8.067299 | 0.001211 | 0.062432 |
| CBWD4P | 2.086581 | 0.314218 | 0.044228 | 0.402111 |
| ABCC5 | 2.086073 | 6.869234 | 0.000132 | 0.016077 |
| AL928654.1 | 2.084758 | 0.9109 | 0.024374 | 0.305778 |
| HIST3H2A | 2.084036 | 3.597529 | 0.003187 | 0.109941 |
| SLC22A18 | 2.083097 | 1.311867 | 0.013102 | 0.223646 |
| SDC4 | 2.0814 | 6.086511 | 0.000454 | 0.034488 |
| COLEC11 | 2.078983 | 1.164871 | 0.026816 | 0.320155 |
| IQCN | 2.075739 | 4.065082 | 0.004496 | 0.13112 |
| SLC25A22 | 2.074889 | 4.312054 | 0.002054 | 0.084231 |
| IMPA1 | 2.072212 | 6.206157 | 0.000274 | 0.026052 |
| MANEAL | 2.071992 | 2.772737 | 0.004924 | 0.13695 |
| UBE2S | 2.071721 | 4.233294 | 0.000396 | 0.032238 |
| ELOCP2 | 2.068533 | 0.542047 | 0.049436 | 0.422933 |
| GAS5 | 2.062009 | 7.802899 | 0.000338 | 0.029024 |
| LINC01106 | 2.061288 | 2.984982 | 0.004787 | 0.135115 |
| ACP4 | 2.060992 | 1.256566 | 0.023335 | 0.300474 |
| MME | 2.056748 | 8.427482 | 0.000127 | 0.015774 |
| ADAM11 | 2.054372 | 1.429142 | 0.028949 | 0.331013 |
| TRAPPC6A | 2.051477 | 3.168305 | 0.003571 | 0.116716 |
| RPSAP15 | 2.046621 | 3.21281 | 0.013807 | 0.229786 |
| SLC52A2 | 2.046437 | 2.882281 | 0.00495 | 0.137306 |
| NR2C2AP | 2.044788 | 2.557806 | 0.00628 | 0.155234 |
| SGK1 | 2.04265 | 6.890832 | 0.013406 | 0.225811 |
| CHTF18 | 2.040402 | 3.408838 | 0.002402 | 0.093552 |
| AL162595.1 | 2.039621 | 0.980736 | 0.02011 | 0.277565 |
| RTN2 | 2.039615 | 1.46148 | 0.024304 | 0.305236 |
| TSNARE1 | 2.038389 | 4.342197 | 0.000498 | 0.036606 |
| SIGLEC1 | 2.037796 | 3.620417 | 0.013367 | 0.225549 |
| RAB17 | 2.037587 | 4.553228 | 0.000404 | 0.032325 |
| ASPHD1 | 2.03553 | 1.066122 | 0.023511 | 0.301106 |
| LNX2 | 2.035172 | 6.422076 | 0.005076 | 0.138928 |
| DNAJB1 | 2.032921 | 6.998547 | 0.002373 | 0.092945 |
| CYP4F30P | 2.027799 | 5.03283 | 0.019639 | 0.274587 |
| AL353796.1 | 2.027604 | 0.953698 | 0.028368 | 0.327753 |
| FIRRE | 2.027295 | 4.073247 | 0.016871 | 0.255413 |
| GINS1 | 2.026134 | 3.445299 | 0.002057 | 0.084231 |
| USP32P2 | 2.023997 | 0.39717 | 0.047841 | 0.416976 |
| TK1 | 2.023406 | 3.463831 | 0.004526 | 0.131419 |
| TMEM54 | 2.022216 | 4.144851 | 0.001275 | 0.064282 |
| RPS10P3 | 2.021523 | 0.833635 | 0.027532 | 0.324218 |
| CYHR1 | 2.021362 | 5.145829 | 0.000297 | 0.026967 |
| C19orf48 | 2.019302 | 6.844839 | 0.000948 | 0.053142 |
| MCM10 | 2.017762 | 3.132403 | 0.007681 | 0.1701 |
| KIF2C | 2.011126 | 3.049117 | 0.004172 | 0.125511 |
| CAMK2N1 | 2.010887 | 6.568836 | 0.000852 | 0.050298 |
| C1QTNF6 | 2.010172 | 3.837836 | 0.011939 | 0.213482 |
| TWNK | 2.010067 | 4.21424 | 0.001119 | 0.059661 |
| AC044787.1 | 2.006133 | 0.827037 | 0.032672 | 0.348198 |
| GSDMD | 2.006107 | 4.475285 | 0.030048 | 0.335979 |
| FOXM1 | 2.005699 | 3.851158 | 0.001553 | 0.071892 |
| GSR | 2.004201 | 7.884702 | 0.000164 | 0.018573 |
| XPO6 | 2.000296 | 7.685618 | 0.00322 | 0.110499 |
| MT1X | 1.99677 | 1.821949 | 0.034844 | 0.359131 |
| KIF18B | 1.993319 | 2.511962 | 0.006383 | 0.156371 |
| CD3EAP | 1.983385 | 1.049045 | 0.029226 | 0.332191 |
| NAT14 | 1.982572 | 2.876157 | 0.005587 | 0.146058 |
| SLC25A25-AS1 | 1.982021 | 3.811164 | 0.01307 | 0.223646 |
| SLC7A1 | 1.981441 | 7.59708 | 0.000878 | 0.050858 |
| CDCA3 | 1.98105 | 2.228238 | 0.013401 | 0.225811 |
| LSM4 | 1.977989 | 4.525369 | 0.001209 | 0.062432 |
| SNHG19 | 1.976724 | 3.098672 | 0.009614 | 0.19132 |
| NRN1 | 1.975098 | 2.873677 | 0.020558 | 0.28144 |
| AC105219.3 | 1.973966 | 1.483297 | 0.018071 | 0.263907 |
| SPC24 | 1.972475 | 3.21212 | 0.004127 | 0.124681 |
| AP001207.3 | 1.970111 | 1.253618 | 0.019906 | 0.276152 |
| DCTPP1 | 1.965467 | 3.983287 | 0.002448 | 0.094982 |
| CSKMT | 1.965257 | 2.468188 | 0.045803 | 0.40914 |
| C20orf27 | 1.959371 | 3.3922 | 0.007836 | 0.171764 |
| LAMTOR4 | 1.957747 | 4.658923 | 0.000456 | 0.034522 |
| EXO1 | 1.956676 | 2.953833 | 0.008251 | 0.176369 |
| GTPBP3 | 1.953088 | 3.397804 | 0.007072 | 0.16296 |
| RPL21P28 | 1.951792 | 3.149701 | 0.027275 | 0.322898 |
| NME3 | 1.949921 | 3.47579 | 0.003603 | 0.116872 |
| DUSP2 | 1.94988 | 2.12781 | 0.040723 | 0.387608 |
| AC010761.1 | 1.949352 | 2.602775 | 0.012077 | 0.215011 |
| GTSE1 | 1.945945 | 2.590425 | 0.009467 | 0.189937 |
| THAP12P7 | 1.945647 | 3.331704 | 0.049461 | 0.423041 |
| MSI1 | 1.942049 | 3.883776 | 0.003057 | 0.107664 |
| CABP4 | 1.941946 | 1.503412 | 0.038003 | 0.374034 |
| SMG1P4 | 1.941735 | 3.536307 | 0.005826 | 0.149346 |
| TOP1MT | 1.93739 | 4.573395 | 0.001383 | 0.067268 |
| COMMD5 | 1.936801 | 2.808632 | 0.011741 | 0.212394 |
| IMPDH1P8 | 1.935914 | 1.216706 | 0.026706 | 0.31951 |
| IQGAP3 | 1.935514 | 4.094779 | 0.001799 | 0.077225 |
| PLK1 | 1.934876 | 3.170258 | 0.00842 | 0.178217 |
| RIMBP2 | 1.93237 | 4.194659 | 0.038458 | 0.376459 |
| RPL30 | 1.931264 | 7.19855 | 0.001717 | 0.074807 |
| PSPHP1 | 1.929883 | 0.997745 | 0.032193 | 0.345994 |
| AL354920.1 | 1.929363 | 1.193542 | 0.019441 | 0.273555 |
| PPP1R14BP3 | 1.923507 | 0.966623 | 0.042359 | 0.394516 |
| AR | 1.922193 | 9.491958 | 0.000661 | 0.043231 |
| AC064807.1 | 1.922027 | 2.419398 | 0.012717 | 0.219621 |
| APTR | 1.921582 | 3.688405 | 0.018325 | 0.266032 |
| RAB42 | 1.921147 | 1.424417 | 0.030446 | 0.338057 |
| RPL18A | 1.92085 | 8.980784 | 0.001747 | 0.075491 |
| MCM7 | 1.918781 | 6.456419 | 0.000149 | 0.017659 |
| TSPO | 1.916154 | 3.821201 | 0.002491 | 0.095481 |
| MT1F | 1.915891 | 1.470933 | 0.037543 | 0.37185 |
| OAS3 | 1.913828 | 6.520996 | 0.002183 | 0.088027 |
| LRP1B | 1.913501 | 3.864968 | 0.048193 | 0.418134 |
| PSMG3-AS1 | 1.912661 | 2.612154 | 0.011886 | 0.213108 |
| ARG2 | 1.912246 | 5.930667 | 0.0414 | 0.389921 |
| E2F5 | 1.90898 | 4.713187 | 0.000765 | 0.047 |
| BYSL | 1.901729 | 3.663517 | 0.005742 | 0.148293 |
| RPS3 | 1.900829 | 10.67802 | 0.00232 | 0.092022 |
| DIRAS1 | 1.898094 | 3.32415 | 0.012341 | 0.216717 |
| POLR2H | 1.898012 | 5.336317 | 0.000357 | 0.030129 |
| MRPS12 | 1.897894 | 2.988849 | 0.007288 | 0.165177 |
| GINS3 | 1.893616 | 3.079584 | 0.00636 | 0.156193 |
| FOLR2 | 1.8907 | 2.696842 | 0.014033 | 0.231059 |
| TMEM44-AS1 | 1.890359 | 1.348694 | 0.034932 | 0.359351 |
| TACC3 | 1.889704 | 3.940169 | 0.003169 | 0.109723 |
| EFCAB11 | 1.88735 | 4.190382 | 0.006836 | 0.160585 |
| AIF1L | 1.886884 | 5.171691 | 0.006142 | 0.154356 |
| TROAP | 1.886393 | 2.725001 | 0.013726 | 0.229127 |
| CDKN3 | 1.886061 | 2.613294 | 0.013297 | 0.225288 |
| NSMF | 1.884169 | 5.160457 | 0.003869 | 0.120943 |
| POLQ | 1.883826 | 4.293848 | 0.001648 | 0.073803 |
| SLC13A4 | 1.879012 | 3.025847 | 0.020402 | 0.280098 |
| CENPX | 1.878337 | 4.981915 | 0.001457 | 0.069237 |
| CCDC86 | 1.877314 | 4.583729 | 0.011771 | 0.212405 |
| TOP2A | 1.87693 | 7.078332 | 0.001233 | 0.063045 |
| TMEM241 | 1.875539 | 4.145887 | 0.008326 | 0.17723 |
| TICRR | 1.874061 | 3.202017 | 0.008712 | 0.182683 |
| FKBPL | 1.873686 | 1.922288 | 0.033093 | 0.350714 |
| OSER1-DT | 1.873654 | 3.71642 | 0.004822 | 0.135546 |
| DLX1 | 1.873062 | 4.197486 | 0.003748 | 0.119322 |
| RPS17 | 1.870425 | 3.053254 | 0.011799 | 0.212405 |
| SHARPIN | 1.867992 | 4.238684 | 0.002513 | 0.095925 |
| RASD1 | 1.867529 | 3.361425 | 0.014308 | 0.23337 |
| CDK1 | 1.867376 | 4.715863 | 0.001042 | 0.057064 |
| SYBU | 1.865409 | 5.057686 | 0.001861 | 0.07854 |
| AGRN | 1.865393 | 6.117122 | 0.002222 | 0.088895 |
| ECSIT | 1.86383 | 4.682221 | 0.006887 | 0.160585 |
| BCL7A | 1.863231 | 4.500852 | 0.00636 | 0.156193 |
| COMTD1 | 1.862127 | 1.859633 | 0.028989 | 0.331081 |
| MEPCE | 1.861583 | 5.037393 | 0.00051 | 0.037158 |
| LMNB2 | 1.860484 | 5.368256 | 0.000748 | 0.04642 |
| PNMA2 | 1.859772 | 2.060254 | 0.045178 | 0.406287 |
| CCDC85C | 1.859165 | 4.661407 | 0.001704 | 0.074655 |
| AP1S1 | 1.859152 | 5.577315 | 0.000224 | 0.023148 |
| BRF2 | 1.859017 | 2.577218 | 0.015452 | 0.242906 |
| ASF1B | 1.858665 | 3.024806 | 0.013846 | 0.230285 |
| DPP7 | 1.856863 | 6.061042 | 0.000698 | 0.044665 |
| PANK1 | 1.856619 | 3.713717 | 0.010534 | 0.19938 |
| TRAIP | 1.856252 | 1.912035 | 0.025428 | 0.311824 |
| TIMM13 | 1.854889 | 4.215143 | 0.004025 | 0.123444 |
| LGR4 | 1.854137 | 7.375964 | 0.000515 | 0.037351 |
| CLTRN | 1.852789 | 3.020912 | 0.043347 | 0.398699 |
| DEPDC1B | 1.852399 | 2.667203 | 0.030191 | 0.336714 |
| CENPM | 1.852298 | 2.023098 | 0.033381 | 0.352064 |
| SGO1 | 1.851154 | 1.921075 | 0.022577 | 0.296149 |
| LMNB1 | 1.850702 | 5.451225 | 0.001303 | 0.064962 |
| SSPO | 1.848615 | 3.108885 | 0.020631 | 0.282089 |
| MXD3 | 1.847795 | 2.43115 | 0.012992 | 0.223134 |
| QSOX1 | 1.845498 | 5.341014 | 0.006481 | 0.156872 |
| SLC7A5 | 1.845448 | 4.105243 | 0.023502 | 0.301106 |
| C9orf116 | 1.844373 | 1.680521 | 0.034599 | 0.359025 |
| NEK2 | 1.844158 | 3.020616 | 0.007549 | 0.16821 |
| PPIF | 1.844013 | 4.856801 | 0.001577 | 0.072224 |
| CDC45 | 1.841586 | 2.473325 | 0.017872 | 0.262542 |
| SINHCAF | 1.838176 | 7.029282 | 0.002481 | 0.095472 |
| DTYMK | 1.837285 | 3.789539 | 0.005402 | 0.143501 |
| TREX1 | 1.837283 | 3.598726 | 0.006641 | 0.158657 |
| CDT1 | 1.836711 | 2.089124 | 0.037877 | 0.373247 |
| RPL7P1 | 1.834009 | 7.633654 | 0.005251 | 0.141733 |
| AC016596.1 | 1.831632 | 4.330849 | 0.002738 | 0.10065 |
| ZNHIT2 | 1.831314 | 1.418345 | 0.036972 | 0.369765 |
| H1FX-AS1 | 1.826969 | 1.467639 | 0.027285 | 0.322898 |
| AL365436.1 | 1.824286 | 1.426949 | 0.03316 | 0.35087 |
| RPL7P9 | 1.823365 | 7.282228 | 0.004285 | 0.127424 |
| C2orf15 | 1.823019 | 1.135594 | 0.041961 | 0.392729 |
| CCNA2 | 1.819783 | 4.331961 | 0.003165 | 0.109723 |
| PMS2P1 | 1.818812 | 3.38113 | 0.006827 | 0.160585 |
| PEX16 | 1.817817 | 2.873156 | 0.021487 | 0.28819 |
| CPLX1 | 1.814151 | 2.966536 | 0.008021 | 0.173474 |
| CARD14 | 1.813991 | 2.66269 | 0.016659 | 0.253532 |
| BOLA3 | 1.811451 | 2.599998 | 0.011394 | 0.208733 |
| TTC21B | 1.810195 | 7.227293 | 0.000749 | 0.04642 |
| YEATS2-AS1 | 1.809216 | 1.715025 | 0.038102 | 0.374472 |
| TIMM8A | 1.806447 | 3.540522 | 0.009967 | 0.193967 |
| STARD3NL | 1.806246 | 6.818576 | 0.000995 | 0.054859 |
| SULT1A1 | 1.8055 | 3.640758 | 0.008863 | 0.183729 |
| SNHG7 | 1.803934 | 5.736919 | 0.001417 | 0.068264 |
| BCYRN1 | 1.803771 | 3.917295 | 0.007742 | 0.170694 |
| DNMT3B | 1.800623 | 3.261897 | 0.02373 | 0.302114 |
| PARP10 | 1.800574 | 5.680976 | 0.014811 | 0.237406 |
| PYGL | 1.799106 | 5.205595 | 0.033261 | 0.351206 |
| GPAA1 | 1.796746 | 5.230496 | 0.00076 | 0.046951 |
| DDX39A | 1.79586 | 5.147705 | 0.003132 | 0.109456 |
| DECR2 | 1.795796 | 2.9231 | 0.013439 | 0.226171 |
| RN7SKP255 | 1.793893 | 5.396299 | 0.001486 | 0.0701 |
| AAMP | 1.793536 | 6.117331 | 0.005608 | 0.146453 |
| ARHGAP39 | 1.793418 | 3.305683 | 0.011783 | 0.212405 |
| CCNB1 | 1.793255 | 4.092958 | 0.005785 | 0.148738 |
| COX6C | 1.793087 | 7.062293 | 0.002043 | 0.083943 |
| BCL2L12 | 1.793008 | 1.57182 | 0.033393 | 0.352064 |
| RPS2 | 1.792679 | 9.9771 | 0.001685 | 0.074318 |
| ATP5MC1 | 1.791949 | 4.246701 | 0.002503 | 0.095637 |
| ENTR1 | 1.791626 | 5.203801 | 0.000584 | 0.039436 |
| CYB561A3 | 1.789782 | 4.127131 | 0.004458 | 0.131 |
| SNHG20 | 1.788536 | 7.903671 | 0.005741 | 0.148293 |
| TRERF1 | 1.785133 | 4.793561 | 0.004885 | 0.136301 |
| RFC3 | 1.784115 | 5.341927 | 0.000563 | 0.038546 |
| AC087752.3 | 1.783985 | 1.358104 | 0.0299 | 0.33539 |
| HSD3B7 | 1.781306 | 2.549986 | 0.039645 | 0.382953 |
| HIST1H2BN | 1.781248 | 3.461687 | 0.005816 | 0.14919 |
| RNASEH2A | 1.780871 | 3.697499 | 0.019744 | 0.275465 |
| LINC00649 | 1.779213 | 3.784982 | 0.014874 | 0.237888 |
| TLCD1 | 1.77674 | 3.311933 | 0.011994 | 0.214043 |
| LRRC61 | 1.776223 | 2.662189 | 0.040306 | 0.38563 |
| RPL7AP11 | 1.775998 | 2.478297 | 0.022624 | 0.296149 |
| DCAF13 | 1.775857 | 6.410395 | 0.001078 | 0.058432 |
| JUND | 1.775546 | 6.307008 | 0.002863 | 0.103721 |
| NCAPG | 1.775497 | 4.48844 | 0.006061 | 0.153076 |
| NDUFA4 | 1.774651 | 6.491167 | 0.001864 | 0.07854 |
| RPL7 | 1.774621 | 10.50981 | 0.003723 | 0.119296 |
| BIRC5 | 1.774589 | 2.654079 | 0.015477 | 0.242995 |
| PLIN4 | 1.773924 | 3.908865 | 0.019073 | 0.270444 |
| JAG2 | 1.773902 | 4.289253 | 0.026533 | 0.31844 |
| RFC4 | 1.772633 | 4.065548 | 0.00387 | 0.120943 |
| OPLAH | 1.771255 | 3.91572 | 0.005499 | 0.145196 |
| TUBA1C | 1.770188 | 5.12834 | 0.001892 | 0.079531 |
| BX664615.1 | 1.769696 | 1.435319 | 0.048157 | 0.418134 |
| NPM3 | 1.768972 | 3.143404 | 0.01611 | 0.24826 |
| METTL1 | 1.768676 | 2.92451 | 0.016458 | 0.251152 |
| RIDA | 1.767463 | 4.113871 | 0.009851 | 0.193123 |
| ARMC6 | 1.7618 | 4.135188 | 0.002611 | 0.098557 |
| LBH | 1.759804 | 6.122507 | 0.03548 | 0.362817 |
| AP003352.1 | 1.759766 | 2.056282 | 0.04443 | 0.402848 |
| PUSL1 | 1.757743 | 1.14624 | 0.045293 | 0.406736 |
| ZNF385C | 1.754599 | 1.385517 | 0.029061 | 0.331081 |
| FAM86EP | 1.752274 | 1.213195 | 0.046149 | 0.40992 |
| NUP93 | 1.750783 | 5.917476 | 0.006597 | 0.158567 |
| RIPK2 | 1.750054 | 4.977539 | 0.009392 | 0.189096 |
| CCHCR1 | 1.749277 | 4.241087 | 0.009311 | 0.187892 |
| SPON2 | 1.74883 | 8.313508 | 0.048183 | 0.418134 |
| CBWD3 | 1.747808 | 2.66938 | 0.015536 | 0.243401 |
| TYSND1 | 1.74742 | 4.312144 | 0.004686 | 0.133347 |
| B4GALNT3 | 1.747373 | 3.678863 | 0.029047 | 0.331081 |
| METTL27 | 1.746858 | 2.05321 | 0.030871 | 0.340084 |
| SLC13A3 | 1.744658 | 4.205961 | 0.0145 | 0.234765 |
| AC073957.3 | 1.743818 | 3.045007 | 0.026519 | 0.318384 |
| PUF60 | 1.740416 | 5.666612 | 0.000926 | 0.052282 |
| IRF7 | 1.738227 | 3.803692 | 0.018116 | 0.264226 |
| DOHH | 1.737918 | 2.012394 | 0.021413 | 0.287539 |
| AC093484.4 | 1.737448 | 2.322328 | 0.041485 | 0.390399 |
| C7orf50 | 1.736946 | 4.743586 | 0.005382 | 0.143424 |
| ACSM3 | 1.736618 | 5.193134 | 0.005058 | 0.138788 |
| LRRC20 | 1.735684 | 2.015128 | 0.031461 | 0.342118 |
| DDIT3 | 1.728271 | 4.511892 | 0.010835 | 0.202274 |
| JPT1 | 1.72696 | 5.465834 | 0.000797 | 0.048302 |
| PRR11 | 1.725626 | 3.051318 | 0.012157 | 0.215026 |
| LDHB | 1.724095 | 7.162375 | 0.018726 | 0.268576 |
| HJURP | 1.723335 | 3.444866 | 0.011838 | 0.212783 |
| NOP56 | 1.72228 | 6.43034 | 0.000867 | 0.050512 |
| TEDC2 | 1.721813 | 1.407621 | 0.046958 | 0.41299 |
| RN7SKP80 | 1.719877 | 6.13407 | 0.001652 | 0.07388 |
| SDK1 | 1.71811 | 6.261948 | 0.038088 | 0.374456 |
| PRKAR1B | 1.718075 | 4.07116 | 0.007812 | 0.171559 |
| FAM83H | 1.716551 | 6.012099 | 0.000732 | 0.046121 |
| PYCR1 | 1.715543 | 5.71217 | 0.00068 | 0.044054 |
| HNF4G | 1.715252 | 5.149225 | 0.024626 | 0.306453 |
| PABPC1L | 1.714981 | 4.549784 | 0.007311 | 0.165406 |
| CCDC137 | 1.714741 | 3.565053 | 0.006236 | 0.154763 |
| CAPN5 | 1.714263 | 7.064472 | 0.043271 | 0.398587 |
| PGAM5 | 1.711799 | 4.92693 | 0.001711 | 0.074807 |
| SLC26A1 | 1.71129 | 2.782557 | 0.026583 | 0.318713 |
| LRRC45 | 1.707075 | 3.392995 | 0.007929 | 0.172921 |
| PPP1R16A | 1.705316 | 3.620912 | 0.009401 | 0.189168 |
| KHDRBS3 | 1.704629 | 4.203747 | 0.022994 | 0.299084 |
| COX8A | 1.701806 | 2.540557 | 0.029347 | 0.332524 |
| WDR97 | 1.701654 | 1.551127 | 0.043663 | 0.399631 |
| MROH1 | 1.699369 | 4.136567 | 0.004676 | 0.133341 |
| PSPN | 1.69906 | 1.551024 | 0.045204 | 0.406287 |
| POP1 | 1.697892 | 4.301904 | 0.019448 | 0.273555 |
| KAT2A | 1.697013 | 5.6523 | 0.001515 | 0.070787 |
| NOL3 | 1.696918 | 4.452792 | 0.022484 | 0.296001 |
| DDIAS | 1.696727 | 2.322614 | 0.023781 | 0.30246 |
| LBHD1 | 1.696424 | 4.303708 | 0.006711 | 0.159463 |
| POLD1 | 1.695372 | 3.231094 | 0.014032 | 0.231059 |
| MAF1 | 1.693429 | 5.723929 | 0.002683 | 0.099949 |
| ZNF680 | 1.693329 | 7.518481 | 0.011518 | 0.210147 |
| PSPH | 1.69185 | 4.323078 | 0.008087 | 0.174164 |
| CBX4 | 1.691437 | 4.960862 | 0.00838 | 0.177868 |
| SLC25A39 | 1.690621 | 5.621819 | 0.001772 | 0.076335 |
| CHMP4C | 1.690363 | 5.230431 | 0.001287 | 0.064565 |
| PUS1 | 1.688898 | 3.673703 | 0.011733 | 0.212394 |
| RPL22L1 | 1.687826 | 5.54747 | 0.020939 | 0.284407 |
| TUBA1B | 1.687357 | 5.821196 | 0.002335 | 0.092454 |
| PPFIA3 | 1.687295 | 3.328745 | 0.038535 | 0.376729 |
| PKP3 | 1.686339 | 5.03593 | 0.00338 | 0.11282 |
| DNAJC22 | 1.68576 | 2.884954 | 0.039888 | 0.384405 |
| ADGRL1 | 1.682288 | 4.669546 | 0.010897 | 0.203209 |
| UGT8 | 1.682191 | 2.108219 | 0.033478 | 0.352421 |
| MEN1 | 1.681187 | 4.425565 | 0.004273 | 0.127282 |
| TFPT | 1.679201 | 3.474819 | 0.031536 | 0.34215 |
| SNHG15 | 1.676635 | 3.899079 | 0.010021 | 0.19446 |
| TKFC | 1.675444 | 4.065569 | 0.005777 | 0.14863 |
| HSF1 | 1.674938 | 5.452035 | 0.002182 | 0.088027 |
| LRRC75A-AS1 | 1.674613 | 9.205097 | 0.004762 | 0.13462 |
| ZC3H3 | 1.673342 | 3.69928 | 0.011008 | 0.204714 |
| CCDC124 | 1.671462 | 4.184768 | 0.017543 | 0.260076 |
| NDUFAF8 | 1.671325 | 2.903204 | 0.018798 | 0.26912 |
| RNF44 | 1.667988 | 4.889633 | 0.002887 | 0.10406 |
| MAGEH1 | 1.667098 | 6.311925 | 0.047747 | 0.416516 |
| HASPIN | 1.666823 | 1.49918 | 0.047281 | 0.414711 |
| CCDC85B | 1.660262 | 3.101545 | 0.034035 | 0.35566 |
| UNC13B | 1.659459 | 8.756798 | 0.016274 | 0.249885 |
| RN7SL4P | 1.659138 | 10.93501 | 0.012089 | 0.215026 |
| RPL7P23 | 1.658967 | 4.120281 | 0.019369 | 0.272771 |
| CDCA4 | 1.658851 | 3.058788 | 0.017621 | 0.260708 |
| TRAM2 | 1.657337 | 5.773931 | 0.025319 | 0.311048 |
| TTYH3 | 1.656512 | 5.52996 | 0.004311 | 0.128083 |
| PNP | 1.656346 | 4.048276 | 0.006875 | 0.160585 |
| MAL2 | 1.6555 | 7.085864 | 0.000926 | 0.052282 |
| PIR | 1.653212 | 3.593689 | 0.009955 | 0.193967 |
| PLEKHF2 | 1.652349 | 5.857393 | 0.00454 | 0.131422 |
| DHX37 | 1.651082 | 4.091485 | 0.005012 | 0.138066 |
| HELZ2 | 1.649995 | 5.392565 | 0.00743 | 0.166487 |
| ALG3 | 1.64971 | 3.510219 | 0.011786 | 0.212405 |
| OCLN | 1.649266 | 5.484389 | 0.002305 | 0.091512 |
| RHBDD3 | 1.648322 | 3.587249 | 0.014007 | 0.231059 |
| PMM1 | 1.647536 | 4.160849 | 0.008747 | 0.183253 |
| SLC12A9 | 1.647116 | 4.319149 | 0.005494 | 0.145155 |
| PLA2G2A | 1.646109 | 7.646079 | 0.010254 | 0.197348 |
| ZBTB45 | 1.645918 | 2.835825 | 0.0401 | 0.385073 |
| CDK4 | 1.645561 | 4.91953 | 0.003583 | 0.116728 |
| UCK2 | 1.645171 | 5.503558 | 0.00197 | 0.082307 |
| RRP12 | 1.636042 | 4.563173 | 0.00274 | 0.10065 |
| POLR1D | 1.635036 | 6.970151 | 0.023372 | 0.300575 |
| NUDCD1 | 1.634518 | 6.217811 | 0.004488 | 0.13112 |
| MCM5 | 1.633829 | 4.471384 | 0.006546 | 0.157576 |
| PWWP2B | 1.633007 | 3.389272 | 0.03046 | 0.338057 |
| RRAGD | 1.632073 | 5.962985 | 0.006896 | 0.160585 |
| TMUB1 | 1.631154 | 2.849121 | 0.031406 | 0.342028 |
| NDUFB9 | 1.630907 | 5.210244 | 0.004067 | 0.123754 |
| CIT | 1.630591 | 4.422462 | 0.005063 | 0.138788 |
| RAD21 | 1.62899 | 8.103718 | 0.000971 | 0.054083 |
| PITPNM1 | 1.628715 | 4.208069 | 0.010534 | 0.19938 |
| RPL13P12 | 1.627874 | 3.201204 | 0.019348 | 0.272716 |
| RCC1 | 1.627777 | 5.409988 | 0.003081 | 0.108201 |
| PSRC1 | 1.626804 | 1.676572 | 0.045194 | 0.406287 |
| LSM1 | 1.626069 | 3.808449 | 0.020378 | 0.279989 |
| AMOTL1 | 1.62509 | 8.469456 | 0.004159 | 0.125426 |
| FRAT2 | 1.625031 | 3.635943 | 0.010444 | 0.198928 |
| CFD | 1.624327 | 2.333331 | 0.04413 | 0.401651 |
| MTHFD2 | 1.621763 | 5.989715 | 0.002834 | 0.102999 |
| TTLL12 | 1.620611 | 6.430823 | 0.00212 | 0.086172 |
| CCNE2 | 1.619673 | 2.588235 | 0.023548 | 0.301139 |
| PGLS | 1.619355 | 4.142523 | 0.012121 | 0.215026 |
| RUVBL1 | 1.618864 | 5.4078 | 0.001692 | 0.074406 |
| CCDC74A | 1.618614 | 2.646356 | 0.047928 | 0.417415 |
| TXNRD2 | 1.617263 | 4.943599 | 0.02828 | 0.327621 |
| HIST1H4H | 1.616213 | 5.790662 | 0.002412 | 0.093712 |
| RN7SKP71 | 1.615888 | 4.594703 | 0.007869 | 0.171951 |
| LRRC56 | 1.615424 | 2.09745 | 0.0463 | 0.410837 |
| SNHG17 | 1.61499 | 4.383457 | 0.011098 | 0.205722 |
| TFAP4 | 1.613656 | 3.878644 | 0.01396 | 0.230903 |
| RPLP0 | 1.613477 | 10.70113 | 0.003263 | 0.110972 |
| DCXR | 1.613054 | 6.590779 | 0.00498 | 0.137306 |
| ZNF581 | 1.61071 | 3.397742 | 0.020044 | 0.277398 |
| TXN | 1.610085 | 7.598878 | 0.003761 | 0.1194 |
| SNORD17 | 1.610035 | 10.60382 | 0.002248 | 0.089768 |
| CENPA | 1.609961 | 1.676829 | 0.038408 | 0.376292 |
| SAMD10 | 1.608894 | 2.516104 | 0.038136 | 0.374688 |
| TIMM8B | 1.608452 | 4.781574 | 0.004522 | 0.131406 |
| PTGES2 | 1.608306 | 4.54991 | 0.004533 | 0.131422 |
| RCC2 | 1.608147 | 6.490536 | 0.002526 | 0.096081 |
| TNFSF4 | 1.606894 | 2.858511 | 0.043743 | 0.399687 |
| TMEM161A | 1.605369 | 3.112822 | 0.018483 | 0.267127 |
| SRMS | 1.605279 | 2.248742 | 0.04304 | 0.397948 |
| AC079140.2 | 1.604526 | 4.290239 | 0.019164 | 0.271007 |
| MRPS28 | 1.604322 | 2.738024 | 0.021716 | 0.290125 |
| MPP6 | 1.604073 | 7.074742 | 0.013753 | 0.229438 |
| RGS2 | 1.603892 | 7.599356 | 0.049345 | 0.422819 |
| PLA2G7 | 1.603571 | 5.77134 | 0.006195 | 0.154483 |
| SNHG1 | 1.603043 | 6.737269 | 0.006875 | 0.160585 |
| PYROXD2 | 1.602558 | 2.861672 | 0.027316 | 0.322984 |
| RRM2 | 1.602061 | 4.897154 | 0.003398 | 0.113066 |
| MAST3 | 1.600045 | 4.148061 | 0.016043 | 0.247694 |
| SDSL | 1.598015 | 3.059685 | 0.033841 | 0.354396 |
| METTL26 | 1.597224 | 3.975245 | 0.015075 | 0.239509 |
| MRPS16 | 1.597137 | 5.530928 | 0.003357 | 0.112648 |
| MID1IP1 | 1.596814 | 4.756526 | 0.00808 | 0.174109 |
| NLE1 | 1.594688 | 4.143946 | 0.007763 | 0.171034 |
| KIF20A | 1.594475 | 3.640867 | 0.022695 | 0.296523 |
| EPHX2 | 1.593475 | 6.122606 | 0.007965 | 0.173273 |
| KRTCAP3 | 1.591519 | 3.340013 | 0.033782 | 0.354067 |
| RPP25L | 1.591047 | 2.895779 | 0.025148 | 0.310162 |
| ZNF707 | 1.58891 | 3.331371 | 0.024627 | 0.306453 |
| FAM69B | 1.588121 | 2.171886 | 0.036001 | 0.365428 |
| AP001324.1 | 1.588021 | 3.865255 | 0.031015 | 0.340788 |
| POLR2L | 1.585995 | 3.461292 | 0.022492 | 0.296001 |
| GAA | 1.584253 | 5.665165 | 0.003668 | 0.118207 |
| PARPBP | 1.583762 | 3.39044 | 0.030004 | 0.335707 |
| RPSA | 1.583154 | 9.626958 | 0.0074 | 0.166175 |
| TSTA3 | 1.58197 | 5.169119 | 0.017204 | 0.257834 |
| ELMO3 | 1.581101 | 4.225234 | 0.009258 | 0.187736 |
| PCLO | 1.579926 | 7.022893 | 0.036779 | 0.368856 |
| PEX6 | 1.579089 | 4.824796 | 0.004217 | 0.126408 |
| C16orf91 | 1.578973 | 2.447862 | 0.029276 | 0.332315 |
| KNTC1 | 1.578333 | 6.225029 | 0.001856 | 0.07854 |
| GTF3A | 1.57596 | 5.827717 | 0.016103 | 0.248255 |
| PAICS | 1.575526 | 8.054994 | 0.001467 | 0.069421 |
| FEN1 | 1.574993 | 4.05102 | 0.012143 | 0.215026 |
| MACROD1 | 1.573056 | 4.070818 | 0.03527 | 0.361854 |
| ZFP41 | 1.572211 | 3.172996 | 0.034772 | 0.359131 |
| SAC3D1 | 1.569323 | 1.978801 | 0.047996 | 0.417791 |
| NUSAP1 | 1.568767 | 5.033951 | 0.002884 | 0.10406 |
| PAK4 | 1.56843 | 6.049869 | 0.003604 | 0.116872 |
| SELENOH | 1.567983 | 4.088014 | 0.009306 | 0.187892 |
| GIGYF1 | 1.566139 | 6.095956 | 0.004223 | 0.126435 |
| PCK2 | 1.566032 | 3.678843 | 0.023475 | 0.301106 |
| DNPH1 | 1.565479 | 3.07698 | 0.035874 | 0.364888 |
| SNORA5C | 1.563806 | 2.902312 | 0.021294 | 0.286718 |
| MFSD10 | 1.563387 | 3.413066 | 0.018875 | 0.26946 |
| RPS19 | 1.563037 | 8.649729 | 0.006031 | 0.152667 |
| PCAT1 | 1.561107 | 4.665124 | 0.023335 | 0.300474 |
| PAIP2B | 1.55899 | 6.353348 | 0.002057 | 0.084231 |
| CPNE3 | 1.557705 | 7.931966 | 0.003681 | 0.118381 |
| JUNB | 1.557108 | 5.991002 | 0.024151 | 0.304316 |
| VPS28 | 1.554829 | 5.42558 | 0.004465 | 0.131077 |
| ST6GAL1 | 1.553571 | 7.275454 | 0.024808 | 0.307855 |
| FASN | 1.553053 | 9.283553 | 0.007389 | 0.166175 |
| B3GAT3 | 1.551411 | 4.176793 | 0.009111 | 0.186839 |
| CENPV | 1.551135 | 4.0225 | 0.028257 | 0.327574 |
| POP7 | 1.551089 | 3.829061 | 0.01231 | 0.216388 |
| AC024293.1 | 1.549925 | 5.200944 | 0.007234 | 0.164363 |
| MAZ | 1.548593 | 3.914038 | 0.017943 | 0.263043 |
| FAM189B | 1.547108 | 4.168866 | 0.008637 | 0.181754 |
| HMMR | 1.547076 | 4.002238 | 0.017783 | 0.261695 |
| FAM83D | 1.546268 | 3.219149 | 0.038047 | 0.374252 |
| TSEN34 | 1.54508 | 4.195968 | 0.011275 | 0.207435 |
| WDR34 | 1.544258 | 3.689955 | 0.015834 | 0.2459 |
| AC024560.2 | 1.543961 | 1.966906 | 0.044789 | 0.404116 |
| PVT1 | 1.543202 | 5.910271 | 0.004821 | 0.135546 |
| P3H4 | 1.541419 | 4.124187 | 0.022047 | 0.292109 |
| CCDC144B | 1.540453 | 5.65123 | 0.003628 | 0.117237 |
| C8orf33 | 1.540372 | 5.855284 | 0.004363 | 0.129289 |
| ZNF736 | 1.540019 | 6.553099 | 0.018475 | 0.267127 |
| RAD54B | 1.538634 | 3.05105 | 0.03258 | 0.348198 |
| NHP2 | 1.537916 | 3.156181 | 0.023486 | 0.301106 |
| NAP1L2 | 1.537206 | 4.382607 | 0.013494 | 0.22665 |
| BZW2 | 1.536732 | 5.586027 | 0.006472 | 0.156814 |
| GATAD2A | 1.53591 | 6.009039 | 0.003226 | 0.110539 |
| PKN1 | 1.535509 | 6.09731 | 0.003942 | 0.122313 |
| FAM53B | 1.535441 | 4.687292 | 0.00916 | 0.187395 |
| TMEM132A | 1.534906 | 4.449921 | 0.021253 | 0.286399 |
| PTP4A1 | 1.533278 | 8.371866 | 0.028246 | 0.327574 |
| PARVB | 1.532947 | 4.856283 | 0.008992 | 0.185278 |
| RPS2P46 | 1.532291 | 7.083835 | 0.006165 | 0.154356 |
| ALYREF | 1.532274 | 3.787192 | 0.013465 | 0.226461 |
| CPAMD8 | 1.531356 | 4.683254 | 0.018976 | 0.270012 |
| RAD54L | 1.531186 | 2.123684 | 0.042204 | 0.393607 |
| RPS12 | 1.530634 | 8.582565 | 0.011655 | 0.211492 |
| SSBP4 | 1.53017 | 3.599802 | 0.031482 | 0.342118 |
| INCENP | 1.5292 | 4.193871 | 0.010079 | 0.194673 |
| ZDHHC12 | 1.527409 | 2.710246 | 0.028245 | 0.327574 |
| DUS3L | 1.527082 | 3.402263 | 0.019208 | 0.271402 |
| COA4 | 1.52692 | 5.450579 | 0.007886 | 0.172204 |
| QTRT1 | 1.524598 | 4.057142 | 0.014805 | 0.237406 |
| TBRG4 | 1.524405 | 4.955793 | 0.005547 | 0.145325 |
| SNHG8 | 1.523842 | 6.551562 | 0.01644 | 0.251152 |
| HSPA1B | 1.522176 | 6.495433 | 0.00433 | 0.128551 |
| SPR | 1.519832 | 4.691483 | 0.004682 | 0.133341 |
| XRCC2 | 1.518577 | 3.205717 | 0.029411 | 0.332645 |
| RPL10P9 | 1.517921 | 3.614724 | 0.019998 | 0.276867 |
| GRB7 | 1.517522 | 3.662586 | 0.024002 | 0.303555 |
| PPP1R26-AS1 | 1.516906 | 3.056486 | 0.026358 | 0.316894 |
| ZNF512B | 1.512168 | 4.722084 | 0.004894 | 0.136336 |
| KIF5C | 1.511337 | 8.321188 | 0.018032 | 0.263646 |
| SLC4A2 | 1.511243 | 5.235303 | 0.006197 | 0.154483 |
| MBOAT2 | 1.511227 | 9.240718 | 0.011574 | 0.210802 |
| AGPAT2 | 1.508647 | 3.837172 | 0.014771 | 0.237131 |
| GPSM1 | 1.507868 | 4.111093 | 0.024467 | 0.306233 |
| GATD3A | 1.505995 | 6.136224 | 0.002851 | 0.103526 |
| DDX12P | 1.505382 | 2.929167 | 0.030916 | 0.340359 |
| SKA3 | 1.504564 | 3.404952 | 0.02448 | 0.306233 |
| CDCA8 | 1.504355 | 2.752813 | 0.031889 | 0.343914 |
| NDUFV1 | 1.504214 | 5.916598 | 0.005139 | 0.139833 |
| NME1 | 1.502601 | 5.185171 | 0.004648 | 0.133171 |
| CTDSP1 | 1.501333 | 6.738535 | 0.02797 | 0.326784 |
| RPL18 | 1.497795 | 8.843292 | 0.005142 | 0.139833 |
| RPS21 | 1.497446 | 7.200956 | 0.014283 | 0.233291 |
| UGDH | 1.496846 | 7.284217 | 0.008326 | 0.17723 |
| ABCA2 | 1.495786 | 6.414778 | 0.011297 | 0.207619 |
| APEX2 | 1.495109 | 3.897655 | 0.011742 | 0.212394 |
| DPM2 | 1.494268 | 3.54278 | 0.019036 | 0.270223 |
| MAD2L1 | 1.494265 | 4.436368 | 0.008193 | 0.175507 |
| POMP | 1.494003 | 6.522279 | 0.015486 | 0.242995 |
| EIF3E | 1.493132 | 9.67029 | 0.00946 | 0.189906 |
| MPST | 1.49309 | 4.67103 | 0.008406 | 0.178132 |
| RPS6KA5 | 1.493029 | 5.263801 | 0.034723 | 0.359028 |
| AL356488.2 | 1.492208 | 8.36063 | 0.004058 | 0.123754 |
| FAAP100 | 1.491988 | 4.573381 | 0.006387 | 0.156371 |
| RACGAP1 | 1.491258 | 5.266878 | 0.005286 | 0.142059 |
| EPB41L4B | 1.490904 | 6.533027 | 0.005082 | 0.138928 |
| CENPF | 1.490467 | 6.646934 | 0.006139 | 0.154356 |
| AC092902.2 | 1.490104 | 3.115414 | 0.038447 | 0.376459 |
| TMEM254-AS1 | 1.489423 | 3.062222 | 0.029653 | 0.33384 |
| U2AF2 | 1.48835 | 6.273375 | 0.003567 | 0.116684 |
| CACNB1 | 1.487607 | 2.567814 | 0.041015 | 0.38918 |
| DANCR | 1.486547 | 5.026124 | 0.005398 | 0.143501 |
| CRNDE | 1.486479 | 3.973946 | 0.032368 | 0.347061 |
| SMIM19 | 1.485744 | 4.746431 | 0.026059 | 0.315355 |
| SOX12 | 1.485286 | 4.664592 | 0.014389 | 0.233916 |
| NDUFB6 | 1.484754 | 3.523368 | 0.03788 | 0.373247 |
| SSRP1 | 1.483781 | 6.911064 | 0.002568 | 0.097143 |
| MED30 | 1.483411 | 2.342434 | 0.040273 | 0.38563 |
| SCN3A | 1.483137 | 2.482542 | 0.041628 | 0.390891 |
| FIS1 | 1.481203 | 5.897758 | 0.007225 | 0.164349 |
| ZNHIT1 | 1.480842 | 5.409215 | 0.006402 | 0.156384 |
| CCDC51 | 1.480373 | 3.085256 | 0.030333 | 0.337408 |
| TRAF7 | 1.480296 | 5.261488 | 0.006859 | 0.160585 |
| PPP1R3D | 1.479861 | 4.586504 | 0.036391 | 0.367143 |
| PTTG1 | 1.479393 | 3.725177 | 0.019085 | 0.270444 |
| RRS1 | 1.478846 | 3.230207 | 0.044827 | 0.404217 |
| ACOT7 | 1.478182 | 3.341857 | 0.033139 | 0.350841 |
| TDP1 | 1.477216 | 4.655266 | 0.017008 | 0.25639 |
| RMI1 | 1.476073 | 5.291022 | 0.01196 | 0.213708 |
| AC026403.1 | 1.475584 | 4.993278 | 0.009207 | 0.187717 |
| NUDT22 | 1.474547 | 2.833089 | 0.045483 | 0.407912 |
| UTP23 | 1.474037 | 5.325955 | 0.003696 | 0.118664 |
| SLC35F2 | 1.471419 | 6.313856 | 0.009668 | 0.191514 |
| IRAK1 | 1.47087 | 6.205457 | 0.004383 | 0.129682 |
| PCED1A | 1.469784 | 4.368083 | 0.00995 | 0.193967 |
| ANKMY1 | 1.468951 | 4.209427 | 0.012115 | 0.215026 |
| PRIM1 | 1.468161 | 3.224619 | 0.030136 | 0.336526 |
| FARSA | 1.468022 | 5.103133 | 0.009706 | 0.192046 |
| COLGALT1 | 1.465916 | 6.096167 | 0.012256 | 0.216102 |
| ZNF517 | 1.464411 | 3.048941 | 0.030178 | 0.336714 |
| SPAG5 | 1.464191 | 3.934412 | 0.018301 | 0.265862 |
| WNK2 | 1.463517 | 6.04398 | 0.025455 | 0.312028 |
| ZNF285 | 1.462033 | 3.93408 | 0.014876 | 0.237888 |
| MGME1 | 1.460024 | 4.69167 | 0.012912 | 0.222307 |
| SRM | 1.45873 | 4.586756 | 0.025248 | 0.310506 |
| SNRPB | 1.457696 | 4.964634 | 0.007846 | 0.171764 |
| BCS1L | 1.457361 | 4.623313 | 0.011893 | 0.213108 |
| LRRC14 | 1.457015 | 3.933736 | 0.015082 | 0.239509 |
| PLPP1 | 1.455647 | 7.418333 | 0.014248 | 0.233118 |
| H1FX | 1.455517 | 4.514527 | 0.010488 | 0.199191 |
| CPSF1 | 1.455304 | 5.617894 | 0.005462 | 0.144617 |
| RTKN | 1.454984 | 4.667142 | 0.017843 | 0.262257 |
| EIF3H | 1.454878 | 8.066173 | 0.003734 | 0.119322 |
| DHX34 | 1.454607 | 4.498362 | 0.013672 | 0.228526 |
| ARPC1A | 1.454514 | 5.918994 | 0.00579 | 0.148744 |
| UBE2J1 | 1.452727 | 8.262012 | 0.038389 | 0.376267 |
| PLCB3 | 1.451707 | 4.989895 | 0.013385 | 0.225707 |
| TMX2 | 1.449342 | 5.838921 | 0.006406 | 0.156384 |
| MARS2 | 1.447664 | 2.80688 | 0.043931 | 0.400672 |
| MRTO4 | 1.446763 | 5.192309 | 0.009593 | 0.191135 |
| BEND4 | 1.446758 | 6.943298 | 0.038945 | 0.378517 |
| APRT | 1.444896 | 4.916174 | 0.0078 | 0.171524 |
| RPS7P11 | 1.444731 | 5.769243 | 0.009264 | 0.187736 |
| MEST | 1.443185 | 5.37219 | 0.011004 | 0.204714 |
| RPS5 | 1.442599 | 8.380299 | 0.015864 | 0.2459 |
| AZIN1-AS1 | 1.442342 | 3.445078 | 0.033537 | 0.352439 |
| FKBP4 | 1.441557 | 7.36186 | 0.003037 | 0.107337 |
| RPL13AP5 | 1.440668 | 7.334121 | 0.00717 | 0.163776 |
| ADAM9 | 1.440609 | 7.707179 | 0.014028 | 0.231059 |
| RPL28 | 1.439888 | 8.684901 | 0.012497 | 0.218 |
| KLHDC3 | 1.439488 | 5.814431 | 0.006964 | 0.161548 |
| UAP1L1 | 1.438993 | 3.995589 | 0.022619 | 0.296149 |
| SEMA6C | 1.436998 | 3.063228 | 0.037082 | 0.369947 |
| PACSIN3 | 1.436789 | 4.590144 | 0.014005 | 0.231059 |
| UCKL1 | 1.436699 | 4.195142 | 0.01548 | 0.242995 |
| TRGV9 | 1.436513 | 6.109079 | 0.035327 | 0.362026 |
| RPL24P4 | 1.434928 | 5.509262 | 0.017218 | 0.257899 |
| MRPL41 | 1.433968 | 4.402692 | 0.013808 | 0.229786 |
| HTRA2 | 1.433728 | 3.243052 | 0.029324 | 0.33243 |
| ATP5F1D | 1.433054 | 4.478428 | 0.016476 | 0.251237 |
| SLC3A2 | 1.432509 | 5.969252 | 0.017072 | 0.256745 |
| AC073861.1 | 1.431817 | 3.84362 | 0.03061 | 0.338734 |
| RPLP0P6 | 1.431523 | 6.394644 | 0.008636 | 0.181754 |
| MELK | 1.429964 | 3.406409 | 0.032266 | 0.346342 |
| QSOX2 | 1.42857 | 5.028641 | 0.005442 | 0.144242 |
| SSNA1 | 1.42744 | 3.12963 | 0.039282 | 0.381054 |
| FANCG | 1.425381 | 3.605215 | 0.028155 | 0.327357 |
| HELLS | 1.424509 | 5.674397 | 0.006852 | 0.160585 |
| H19 | 1.423592 | 4.639586 | 0.043998 | 0.400871 |
| STK25 | 1.423426 | 5.514246 | 0.006843 | 0.160585 |
| MTX2 | 1.420811 | 4.706645 | 0.027533 | 0.324218 |
| RNU4-1 | 1.420481 | 8.550513 | 0.024501 | 0.306233 |
| RPL36 | 1.420274 | 7.182851 | 0.01459 | 0.235496 |
| TMEM134 | 1.419387 | 4.629033 | 0.014589 | 0.235496 |
| CHCHD3 | 1.418441 | 7.475511 | 0.009946 | 0.193967 |
| CITED2 | 1.418396 | 6.737862 | 0.004979 | 0.137306 |
| WIZ | 1.418191 | 4.876176 | 0.015431 | 0.242761 |
| HOXB13 | 1.417854 | 7.220747 | 0.014503 | 0.234765 |
| RITA1 | 1.417747 | 3.497083 | 0.031275 | 0.34136 |
| DNAAF5 | 1.414417 | 4.221295 | 0.014323 | 0.233376 |
| DDX58 | 1.41155 | 6.631182 | 0.025183 | 0.310391 |
| SNAP47 | 1.408771 | 4.5476 | 0.014512 | 0.234768 |
| AHCY | 1.4082 | 7.409349 | 0.008587 | 0.180955 |
| AC087741.1 | 1.407323 | 3.232016 | 0.048226 | 0.41831 |
| TAF4B | 1.406354 | 4.655688 | 0.027692 | 0.325106 |
| ZNF276 | 1.406317 | 3.964777 | 0.049129 | 0.421886 |
| CLUH | 1.405634 | 5.531329 | 0.007609 | 0.169093 |
| ELOVL6 | 1.405434 | 5.261295 | 0.006298 | 0.155403 |
| MRPL24 | 1.404142 | 4.832355 | 0.010815 | 0.202003 |
| HSPBP1 | 1.40347 | 3.263119 | 0.047427 | 0.415579 |
| ATAD2 | 1.403048 | 7.186255 | 0.006756 | 0.159859 |
| SPDYE3 | 1.402983 | 3.474348 | 0.038754 | 0.377485 |
| TMEM141 | 1.402843 | 4.066191 | 0.020592 | 0.28168 |
| RRP7BP | 1.402151 | 3.880717 | 0.023004 | 0.299084 |
| TUFT1 | 1.401543 | 5.285517 | 0.006488 | 0.156951 |
| KLF15 | 1.400951 | 4.282498 | 0.024481 | 0.306233 |
| AURKA | 1.400844 | 3.321864 | 0.035284 | 0.361889 |
| RRP7A | 1.40074 | 5.038673 | 0.013475 | 0.226461 |
| CDIPT | 1.400471 | 4.757906 | 0.015799 | 0.245552 |
| TRABD | 1.398533 | 4.11706 | 0.02449 | 0.306233 |
| RUVBL2 | 1.398266 | 5.219206 | 0.007843 | 0.171764 |
| RPLP1 | 1.39826 | 9.529275 | 0.015015 | 0.239097 |
| PRMT1 | 1.397108 | 6.172923 | 0.009924 | 0.193796 |
| PODXL2 | 1.397099 | 5.175104 | 0.007417 | 0.166301 |
| NIPAL3 | 1.39541 | 7.175218 | 0.013185 | 0.224529 |
| NACC1 | 1.394794 | 4.939083 | 0.007099 | 0.162993 |
| CCNB1IP1 | 1.394781 | 5.076868 | 0.007355 | 0.165772 |
| OLFM2 | 1.394136 | 3.82789 | 0.025935 | 0.314336 |
| SQLE | 1.393451 | 5.756825 | 0.018037 | 0.263646 |
| RCAN3 | 1.393051 | 7.089638 | 0.007805 | 0.171524 |
| ZNF71 | 1.392511 | 4.382274 | 0.027934 | 0.326699 |
| IMPDH2 | 1.391209 | 7.490523 | 0.008835 | 0.183581 |
| DLGAP5 | 1.389729 | 3.498306 | 0.034594 | 0.359025 |
| AL355075.4 | 1.389619 | 14.21663 | 0.004679 | 0.133341 |
| GRWD1 | 1.38886 | 4.599395 | 0.014401 | 0.234 |
| GCDH | 1.388839 | 4.57202 | 0.023173 | 0.300022 |
| ZNF367 | 1.388431 | 3.67369 | 0.040363 | 0.385764 |
| DNAJC5 | 1.387587 | 6.298061 | 0.009841 | 0.193044 |
| RETREG1 | 1.385919 | 5.804927 | 0.011242 | 0.207278 |
| C7orf43 | 1.384462 | 2.695274 | 0.047752 | 0.416516 |
| NOL6 | 1.384218 | 5.457411 | 0.024438 | 0.306233 |
| ACAD10 | 1.383548 | 5.330701 | 0.00729 | 0.165177 |
| TUBB2A | 1.382998 | 3.053318 | 0.047782 | 0.41667 |
| BICDL2 | 1.382074 | 3.707757 | 0.039398 | 0.381788 |
| RPL41 | 1.381486 | 7.83716 | 0.013872 | 0.230292 |
| TOR1B | 1.381397 | 5.657232 | 0.006628 | 0.158657 |
| RF00100 | 1.380422 | 15.82414 | 0.007112 | 0.162997 |
| PPAT | 1.380166 | 5.071543 | 0.008778 | 0.183412 |
| SLC35B2 | 1.379727 | 4.630414 | 0.01636 | 0.250559 |
| GALNT2 | 1.379674 | 5.961021 | 0.032982 | 0.349982 |
| HMGN4 | 1.376409 | 5.949082 | 0.009274 | 0.187736 |
| FBXO21 | 1.376049 | 6.908683 | 0.008907 | 0.184196 |
| GRINA | 1.375593 | 6.054043 | 0.005846 | 0.149675 |
| BUD31 | 1.375362 | 4.012845 | 0.031291 | 0.34136 |
| TOLLIP | 1.371256 | 5.923773 | 0.015021 | 0.239097 |
| MBOAT7 | 1.37086 | 5.002701 | 0.019776 | 0.275574 |
| KATNB1 | 1.36864 | 3.598574 | 0.03116 | 0.341192 |
| RPF2 | 1.368078 | 5.966161 | 0.017631 | 0.260708 |
| REX1BD | 1.367004 | 3.340673 | 0.045186 | 0.406287 |
| PLEKHJ1 | 1.366903 | 3.978665 | 0.030816 | 0.339921 |
| IRS2 | 1.366409 | 6.542222 | 0.022645 | 0.296149 |
| LYAR | 1.366184 | 3.24787 | 0.046706 | 0.412346 |
| FBXO46 | 1.364691 | 3.936244 | 0.025275 | 0.310622 |
| FOXO3 | 1.363928 | 7.709502 | 0.014524 | 0.234768 |
| TMEM250 | 1.363331 | 3.864816 | 0.022517 | 0.296001 |
| ERLIN2 | 1.361232 | 6.923436 | 0.015195 | 0.240519 |
| CAD | 1.358831 | 5.43848 | 0.006776 | 0.159859 |
| ARL6IP6 | 1.357989 | 3.480504 | 0.036108 | 0.36565 |
| WDR5 | 1.357088 | 5.237343 | 0.011138 | 0.206134 |
| NOP16 | 1.356282 | 3.23838 | 0.042378 | 0.394592 |
| ASPM | 1.356238 | 5.794346 | 0.006635 | 0.158657 |
| POLR2F | 1.35545 | 3.707466 | 0.045868 | 0.409232 |
| RNF34 | 1.355372 | 5.503769 | 0.014043 | 0.231059 |
| PHB | 1.35361 | 5.387015 | 0.008817 | 0.183581 |
| HSPH1 | 1.353243 | 7.467613 | 0.017441 | 0.259234 |
| COX5B | 1.353242 | 6.243789 | 0.007491 | 0.167397 |
| RPS18 | 1.351707 | 9.226016 | 0.020468 | 0.280816 |
| PEX2 | 1.351556 | 6.545863 | 0.010472 | 0.19906 |
| TPD52L1 | 1.35055 | 4.139978 | 0.022726 | 0.296811 |
| MRPL23 | 1.349557 | 3.619955 | 0.029646 | 0.33384 |
| PCGF1 | 1.34931 | 3.176811 | 0.038605 | 0.376939 |
| CBR1 | 1.349035 | 4.894587 | 0.022331 | 0.294638 |
| MRPL13 | 1.348608 | 4.868241 | 0.013272 | 0.225242 |
| YEATS2 | 1.34813 | 6.538516 | 0.013945 | 0.230836 |
| NCLN | 1.347583 | 4.321445 | 0.022788 | 0.297059 |
| ARVCF | 1.34619 | 3.950931 | 0.030325 | 0.337408 |
| KIF23 | 1.3455 | 3.780966 | 0.029275 | 0.332315 |
| AC016739.1 | 1.344889 | 4.51628 | 0.030412 | 0.337843 |
| MRI1 | 1.343796 | 3.736607 | 0.03123 | 0.34136 |
| NUP62 | 1.342931 | 5.205484 | 0.012587 | 0.218706 |
| COQ5 | 1.342259 | 4.775953 | 0.011046 | 0.205093 |
| SPPL2B | 1.341184 | 4.486319 | 0.015735 | 0.245117 |
| SSH3 | 1.338592 | 5.170255 | 0.026626 | 0.319007 |
| TRAF2 | 1.338363 | 3.436869 | 0.040724 | 0.387608 |
| ZFAS1 | 1.338269 | 6.193665 | 0.031035 | 0.340788 |
| DSN1 | 1.337918 | 4.442205 | 0.041152 | 0.38947 |
| RPL13A | 1.337196 | 10.56281 | 0.010589 | 0.199631 |
| RPL37A | 1.336949 | 9.000015 | 0.011094 | 0.205722 |
| EPHB4 | 1.334938 | 5.658897 | 0.031195 | 0.34136 |
| XYLB | 1.334639 | 4.47211 | 0.016967 | 0.255956 |
| C19orf70 | 1.333116 | 3.401252 | 0.04444 | 0.402848 |
| MRPS18B | 1.332935 | 5.529244 | 0.021162 | 0.285723 |
| SLC37A4 | 1.332295 | 3.629432 | 0.03673 | 0.368727 |
| PLA2G6 | 1.330746 | 4.256471 | 0.018955 | 0.270012 |
| FOSB | 1.329514 | 5.355183 | 0.046071 | 0.40976 |
| GALNT3 | 1.329416 | 6.678237 | 0.013233 | 0.225127 |
| HSPD1 | 1.329167 | 8.882964 | 0.008809 | 0.183581 |
| FAM234B | 1.328955 | 6.140602 | 0.007311 | 0.165406 |
| CTSD | 1.328604 | 6.822086 | 0.00958 | 0.191135 |
| RPS7 | 1.32845 | 9.417562 | 0.013283 | 0.225242 |
| KIF11 | 1.327813 | 5.153981 | 0.013107 | 0.223646 |
| CENPE | 1.32601 | 5.798361 | 0.033893 | 0.354614 |
| TMEM129 | 1.325892 | 4.85992 | 0.012617 | 0.218882 |
| SCD | 1.325669 | 7.212049 | 0.041989 | 0.392769 |
| LETM1 | 1.325519 | 5.883442 | 0.009006 | 0.185342 |
| TRMT1 | 1.324408 | 4.527828 | 0.028808 | 0.330485 |
| DHFR | 1.324272 | 5.195859 | 0.010546 | 0.199443 |
| ENGASE | 1.32405 | 4.998079 | 0.043946 | 0.400672 |
| MRPL58 | 1.323519 | 4.126171 | 0.040315 | 0.38563 |
| LBR | 1.323378 | 6.400731 | 0.013908 | 0.230669 |
| DDX54 | 1.323241 | 5.236896 | 0.012151 | 0.215026 |
| SLC6A8 | 1.322576 | 5.317837 | 0.010504 | 0.199191 |
| CBX8 | 1.321879 | 3.734595 | 0.033537 | 0.352439 |
| SELENOW | 1.31941 | 6.767189 | 0.009094 | 0.186598 |
| RPL6P27 | 1.318835 | 4.485738 | 0.032786 | 0.34887 |
| GART | 1.318302 | 6.912982 | 0.008296 | 0.176991 |
| HOOK2 | 1.31739 | 5.105905 | 0.022436 | 0.295493 |
| IGSF9 | 1.316951 | 3.735724 | 0.045634 | 0.408444 |
| ZNF623 | 1.316869 | 6.13286 | 0.01081 | 0.202003 |
| B4GALT2 | 1.316717 | 4.452182 | 0.031723 | 0.343304 |
| THOP1 | 1.314531 | 4.952095 | 0.011716 | 0.212324 |
| SIPA1L3 | 1.313473 | 6.49985 | 0.028573 | 0.32902 |
| DDX49 | 1.312033 | 4.38317 | 0.023289 | 0.300391 |
| LPCAT1 | 1.311161 | 6.419712 | 0.032733 | 0.348524 |
| ARHGAP19 | 1.311099 | 4.416238 | 0.018873 | 0.26946 |
| FPGS | 1.310004 | 4.735538 | 0.015105 | 0.239768 |
| PES1 | 1.308455 | 5.698883 | 0.019762 | 0.275572 |
| ATF3 | 1.30762 | 5.253832 | 0.026378 | 0.317029 |
| POLD2 | 1.307447 | 5.589783 | 0.020734 | 0.282947 |
| DNASE2 | 1.307283 | 4.742567 | 0.027992 | 0.326784 |
| EME2 | 1.307136 | 4.23313 | 0.028105 | 0.326919 |
| PCYT2 | 1.307134 | 4.531572 | 0.025119 | 0.309991 |
| SLC39A10 | 1.306646 | 7.445187 | 0.028044 | 0.32679 |
| FKBP11 | 1.306574 | 4.140052 | 0.047373 | 0.415316 |
| SNHG16 | 1.305478 | 6.061998 | 0.011356 | 0.208264 |
| ARHGAP11A | 1.305312 | 4.0732 | 0.031474 | 0.342118 |
| CHAF1A | 1.305148 | 4.27694 | 0.023372 | 0.300575 |
| PPA1 | 1.304056 | 7.051553 | 0.01334 | 0.225549 |
| CKS1B | 1.303541 | 3.672266 | 0.04764 | 0.416274 |
| CERS6 | 1.303123 | 7.236553 | 0.015069 | 0.239509 |
| PRPF4 | 1.30047 | 5.309372 | 0.020059 | 0.277455 |
| RNF187 | 1.299529 | 5.779218 | 0.01295 | 0.222625 |
| MYL6B | 1.299496 | 3.981333 | 0.046987 | 0.41299 |
| RXRA | 1.299491 | 5.842504 | 0.010045 | 0.194673 |
| HDHD5 | 1.299378 | 4.938414 | 0.016668 | 0.253558 |
| SH3YL1 | 1.298497 | 6.446374 | 0.023032 | 0.299103 |
| RTN3 | 1.298488 | 7.123537 | 0.009213 | 0.187717 |
| NPRL2 | 1.298332 | 3.985124 | 0.031785 | 0.343495 |
| CDC6 | 1.296883 | 3.625871 | 0.046193 | 0.410102 |
| SLC38A10 | 1.294232 | 5.870243 | 0.00736 | 0.165787 |
| FIBP | 1.293899 | 5.118012 | 0.016608 | 0.252873 |
| PRPF19 | 1.293894 | 6.518471 | 0.015431 | 0.242761 |
| SS18L2 | 1.292448 | 3.907488 | 0.031142 | 0.341192 |
| HSPE1 | 1.292105 | 4.070273 | 0.028836 | 0.330485 |
| TATDN1 | 1.291815 | 5.494407 | 0.014913 | 0.238222 |
| TOMM40 | 1.290689 | 4.377637 | 0.028745 | 0.330292 |
| FTL | 1.290107 | 9.420986 | 0.032245 | 0.346342 |
| PABPC1 | 1.289286 | 10.83183 | 0.010255 | 0.197348 |
| MARCKSL1 | 1.289272 | 7.349693 | 0.008481 | 0.179277 |
| RPS9 | 1.286946 | 8.766565 | 0.024371 | 0.305778 |
| TRIAP1 | 1.286506 | 4.240774 | 0.033756 | 0.354043 |
| ZNF787 | 1.286066 | 3.754952 | 0.038603 | 0.376939 |
| TCEA2 | 1.284002 | 3.552654 | 0.044032 | 0.401077 |
| ATP5MF | 1.283939 | 4.699012 | 0.019312 | 0.272467 |
| PCBD1 | 1.283382 | 6.307805 | 0.029626 | 0.33384 |
| PGAP3 | 1.282281 | 4.0755 | 0.028197 | 0.327429 |
| RPL35A | 1.280815 | 7.723994 | 0.021387 | 0.287539 |
| RABEPK | 1.280556 | 4.407749 | 0.02972 | 0.333985 |
| RPL7A | 1.279296 | 10.54569 | 0.016758 | 0.254471 |
| PDIA4 | 1.279218 | 7.608452 | 0.007998 | 0.173432 |
| NXPE3 | 1.278808 | 5.443675 | 0.018474 | 0.267127 |
| EEF2 | 1.278195 | 11.34981 | 0.024541 | 0.306233 |
| C9orf64 | 1.27704 | 4.347095 | 0.033501 | 0.352439 |
| UNC119B | 1.276853 | 6.011413 | 0.029988 | 0.335707 |
| C12orf49 | 1.27588 | 5.01386 | 0.027118 | 0.321972 |
| GRK6 | 1.275151 | 4.775802 | 0.03518 | 0.361471 |
| ARHGDIA | 1.27471 | 6.514585 | 0.012636 | 0.218938 |
| BRAT1 | 1.274686 | 4.734527 | 0.022783 | 0.297059 |
| RPL6 | 1.274614 | 9.616147 | 0.013689 | 0.228703 |
| SFXN4 | 1.273601 | 4.826158 | 0.023857 | 0.303138 |
| MRPL49 | 1.272932 | 5.493432 | 0.029724 | 0.333985 |
| NELFA | 1.272797 | 4.226114 | 0.029579 | 0.333558 |
| TIMM10 | 1.271367 | 3.975728 | 0.040958 | 0.388958 |
| ZNF16 | 1.269907 | 3.96593 | 0.043472 | 0.399117 |
| TRMT2A | 1.269709 | 4.401739 | 0.031457 | 0.342118 |
| PMVK | 1.268971 | 4.238013 | 0.034922 | 0.359351 |
| REPIN1 | 1.26831 | 5.674518 | 0.013053 | 0.223646 |
| ZSCAN21 | 1.26667 | 3.230992 | 0.049766 | 0.424168 |
| HIST1H1E | 1.263884 | 6.070039 | 0.009761 | 0.192398 |
| PRDX2 | 1.263646 | 6.544463 | 0.0157 | 0.244777 |
| BCAT2 | 1.263621 | 4.449606 | 0.043973 | 0.400748 |
| SESN1 | 1.262975 | 6.1861 | 0.042873 | 0.397258 |
| MRPS26 | 1.26219 | 4.155622 | 0.032638 | 0.348198 |
| ERCC2 | 1.259755 | 3.74169 | 0.046028 | 0.409485 |
| STMN1 | 1.258645 | 6.505376 | 0.014089 | 0.231342 |
| RRP1 | 1.258514 | 4.163253 | 0.02947 | 0.332762 |
| TSR3 | 1.257672 | 3.522415 | 0.044487 | 0.402977 |
| ACAT1 | 1.257584 | 6.275464 | 0.045104 | 0.406102 |
| TXNRD1 | 1.25751 | 7.126374 | 0.018699 | 0.268576 |
| RAN | 1.256925 | 8.283646 | 0.00883 | 0.183581 |
| SIRT7 | 1.256394 | 3.96942 | 0.034607 | 0.359025 |
| GRHL2 | 1.255628 | 8.104701 | 0.011216 | 0.207023 |
| PLOD3 | 1.2551 | 4.917653 | 0.023919 | 0.303391 |
| EYA2 | 1.25127 | 4.928178 | 0.02306 | 0.299239 |
| PRDX6 | 1.250877 | 7.706327 | 0.028943 | 0.331013 |
| TUBB4B | 1.250472 | 6.853382 | 0.025118 | 0.309991 |
| LGMN | 1.25003 | 7.186573 | 0.028855 | 0.330485 |
| RAB11FIP1 | 1.249219 | 6.503741 | 0.027762 | 0.325682 |
| HMGB2 | 1.247589 | 6.00122 | 0.032008 | 0.344547 |
| MYBBP1A | 1.247493 | 5.417065 | 0.020065 | 0.277455 |
| ECI1 | 1.246644 | 4.199217 | 0.036914 | 0.369765 |
| TALDO1 | 1.244386 | 6.85074 | 0.013097 | 0.223646 |
| SOCS6 | 1.24435 | 6.381713 | 0.018979 | 0.270012 |
| UQCRH | 1.243964 | 6.385238 | 0.017421 | 0.259234 |
| NDUFAF6 | 1.243661 | 5.05194 | 0.021724 | 0.290125 |
| TRAF4 | 1.242807 | 5.394169 | 0.024544 | 0.306233 |
| EIF4A3 | 1.240656 | 5.765567 | 0.015252 | 0.241314 |
| ELOB | 1.239322 | 5.817722 | 0.011838 | 0.212783 |
| NUBP2 | 1.239133 | 3.943452 | 0.037123 | 0.370141 |
| ABT1 | 1.237628 | 4.114735 | 0.039403 | 0.381788 |
| VARS | 1.23747 | 5.051287 | 0.025524 | 0.312219 |
| HNRNPF | 1.236482 | 7.876414 | 0.021117 | 0.285351 |
| AC010343.1 | 1.235076 | 5.954282 | 0.02828 | 0.327621 |
| TRIM28 | 1.234644 | 6.907174 | 0.012564 | 0.218654 |
| TP53I11 | 1.234553 | 6.354515 | 0.016341 | 0.250473 |
| SLC50A1 | 1.234457 | 4.823983 | 0.049183 | 0.422124 |
| CHRAC1 | 1.234269 | 4.476053 | 0.041369 | 0.389808 |
| JRK | 1.234027 | 5.062546 | 0.018531 | 0.267565 |
| TMPO | 1.232549 | 7.049663 | 0.027567 | 0.324502 |
| PFKM | 1.231111 | 5.989802 | 0.0154 | 0.242646 |
| EIF6 | 1.231055 | 4.172243 | 0.032393 | 0.347061 |
| TFDP1 | 1.230734 | 5.858375 | 0.036302 | 0.366642 |
| PTDSS1 | 1.230575 | 5.92391 | 0.014006 | 0.231059 |
| TMEM179B | 1.230315 | 3.894671 | 0.045802 | 0.40914 |
| NELFB | 1.22988 | 4.364242 | 0.037939 | 0.37372 |
| PARL | 1.227158 | 5.025499 | 0.026175 | 0.315552 |
| NIPSNAP1 | 1.226468 | 5.249948 | 0.018236 | 0.26515 |
| SH3GLB2 | 1.22638 | 6.343501 | 0.026503 | 0.31838 |
| SNRPD2 | 1.224079 | 6.070801 | 0.0233 | 0.300425 |
| RAB4A | 1.224001 | 6.31414 | 0.031263 | 0.34136 |
| FBRSL1 | 1.223246 | 5.155215 | 0.041858 | 0.392352 |
| DUS1L | 1.223215 | 5.798388 | 0.012445 | 0.217493 |
| MRPL47 | 1.222447 | 4.823257 | 0.033674 | 0.353462 |
| RANBP1 | 1.222317 | 5.935718 | 0.02035 | 0.279814 |
| TRMT61B | 1.221501 | 4.328859 | 0.030569 | 0.338606 |
| RPL35 | 1.220935 | 9.085483 | 0.023967 | 0.303528 |
| MRPL14 | 1.22045 | 4.60961 | 0.038945 | 0.378517 |
| BRI3 | 1.220435 | 5.121355 | 0.016887 | 0.255413 |
| DKC1 | 1.219975 | 6.075807 | 0.014259 | 0.233118 |
| DPH1 | 1.21991 | 4.153135 | 0.045881 | 0.409232 |
| BRI3BP | 1.219764 | 5.711043 | 0.043503 | 0.399117 |
| ZDHHC16 | 1.217022 | 4.652866 | 0.025358 | 0.311409 |
| ZNF667 | 1.216808 | 5.469314 | 0.027261 | 0.322892 |
| NAA38 | 1.216398 | 4.389997 | 0.032703 | 0.348421 |
| MLST8 | 1.216335 | 3.897958 | 0.048157 | 0.418134 |
| SLC25A1 | 1.215867 | 5.25417 | 0.017743 | 0.26146 |
| PC | 1.215131 | 4.413144 | 0.037729 | 0.37283 |
| MRPS33 | 1.214697 | 4.596255 | 0.041197 | 0.38947 |
| FASTKD2 | 1.214349 | 6.921252 | 0.038343 | 0.376085 |
| GATA2 | 1.213886 | 6.880655 | 0.043537 | 0.399239 |
| HDHD3 | 1.212643 | 4.064427 | 0.043155 | 0.398114 |
| DNAJC19 | 1.212262 | 4.719736 | 0.028385 | 0.327753 |
| MYH14 | 1.211893 | 5.328787 | 0.020857 | 0.28387 |
| MDH2 | 1.211347 | 6.361983 | 0.016783 | 0.254624 |
| RPL12 | 1.21065 | 9.858328 | 0.025583 | 0.312479 |
| RPS3A | 1.209726 | 10.38476 | 0.02917 | 0.331774 |
| NR2F6 | 1.209492 | 4.65114 | 0.023124 | 0.299763 |
| PMPCA | 1.209463 | 5.148892 | 0.020144 | 0.277799 |
| PIM3 | 1.209427 | 4.82121 | 0.042979 | 0.397717 |
| AKAP1 | 1.209322 | 6.902649 | 0.011253 | 0.207313 |
| TIMELESS | 1.209114 | 4.443735 | 0.034838 | 0.359131 |
| TOMM22 | 1.209035 | 5.453621 | 0.027164 | 0.322305 |
| TM2D2 | 1.208989 | 5.11885 | 0.032375 | 0.347061 |
| RANGAP1 | 1.20883 | 5.61953 | 0.019641 | 0.274587 |
| HDGF | 1.208751 | 7.079727 | 0.014788 | 0.237258 |
| MSRB1 | 1.207857 | 3.885596 | 0.046785 | 0.412688 |
| NCAPD2 | 1.207445 | 6.008264 | 0.017963 | 0.263119 |
| WDHD1 | 1.204958 | 4.100492 | 0.040145 | 0.385263 |
| MLLT1 | 1.20482 | 4.307853 | 0.034707 | 0.359025 |
| ATP5F1B | 1.204731 | 8.034692 | 0.01741 | 0.259234 |
| TUFM | 1.204053 | 6.192652 | 0.016219 | 0.249152 |
| PDAP1 | 1.20328 | 5.742413 | 0.018897 | 0.26967 |
| PLPP5 | 1.201603 | 7.132479 | 0.025727 | 0.313156 |
| SMYD5 | 1.201148 | 4.312488 | 0.036428 | 0.367154 |
| DENND1C | 1.201087 | 4.04919 | 0.037421 | 0.370913 |
| MRPL16 | 1.200676 | 4.464581 | 0.036993 | 0.369765 |
| BCR | 1.20026 | 5.475354 | 0.019083 | 0.270444 |
| FLAD1 | 1.199875 | 4.472332 | 0.034775 | 0.359131 |
| CAMSAP3 | 1.199405 | 4.843883 | 0.025396 | 0.311653 |
| TMEM164 | 1.19812 | 6.027752 | 0.013022 | 0.223424 |
| PSMB5 | 1.197838 | 4.681192 | 0.035597 | 0.363262 |
| MCCC1 | 1.196592 | 5.915626 | 0.019038 | 0.270223 |
| EMG1 | 1.196079 | 4.743952 | 0.045273 | 0.406663 |
| SDF2 | 1.195419 | 5.001585 | 0.02326 | 0.300391 |
| C1orf109 | 1.195194 | 4.689362 | 0.027953 | 0.326784 |
| MRPL15 | 1.195109 | 5.072877 | 0.038427 | 0.376368 |
| SRC | 1.192449 | 6.122868 | 0.037779 | 0.372948 |
| USP54 | 1.192179 | 7.543901 | 0.031866 | 0.343883 |
| APTX | 1.191786 | 4.752816 | 0.036405 | 0.367143 |
| COX7A2 | 1.190398 | 5.649598 | 0.045546 | 0.408025 |
| DESI1 | 1.189815 | 5.549568 | 0.019207 | 0.271402 |
| SRRT | 1.188719 | 6.330796 | 0.014445 | 0.234378 |
| POLR2K | 1.188098 | 6.158181 | 0.029023 | 0.331081 |
| ELOC | 1.187636 | 5.182214 | 0.032581 | 0.348198 |
| YIPF2 | 1.187558 | 4.414641 | 0.037745 | 0.372867 |
| MRPS34 | 1.186418 | 4.816363 | 0.026021 | 0.315052 |
| POLR3H | 1.186303 | 4.817001 | 0.023137 | 0.299763 |
| EIF4G1 | 1.184576 | 8.816786 | 0.015384 | 0.242604 |
| CDK19 | 1.183196 | 7.239455 | 0.046027 | 0.409485 |
| POLR1C | 1.182931 | 4.217056 | 0.04411 | 0.40157 |
| SCARNA10 | 1.182079 | 10.67064 | 0.022405 | 0.295217 |
| FASTKD1 | 1.181793 | 5.09486 | 0.02853 | 0.328636 |
| BUB1 | 1.181026 | 4.51926 | 0.04665 | 0.412028 |
| CPNE1 | 1.179809 | 4.98158 | 0.030025 | 0.335832 |
| GRPEL1 | 1.179301 | 5.113951 | 0.027634 | 0.324844 |
| TRUB2 | 1.179093 | 4.632133 | 0.030551 | 0.338518 |
| GUK1 | 1.179083 | 5.722154 | 0.026999 | 0.321447 |
| JUN | 1.177742 | 7.57217 | 0.036799 | 0.368944 |
| EPS8L2 | 1.17755 | 5.555705 | 0.025477 | 0.312028 |
| COASY | 1.176342 | 4.893231 | 0.02907 | 0.331081 |
| CHID1 | 1.175304 | 4.679828 | 0.029463 | 0.332762 |
| ESRP1 | 1.175054 | 7.558016 | 0.023971 | 0.303528 |
| CYCS | 1.174242 | 6.585186 | 0.032957 | 0.349948 |
| PSAP | 1.172928 | 9.364595 | 0.023956 | 0.303528 |
| H2AFZ | 1.172863 | 5.475449 | 0.023548 | 0.301139 |
| COX6A1 | 1.171784 | 4.712014 | 0.03152 | 0.342118 |
| RABL6 | 1.171525 | 5.79129 | 0.033458 | 0.352373 |
| TMEM41A | 1.168905 | 4.536646 | 0.042099 | 0.393128 |
| AP2M1 | 1.168679 | 7.229477 | 0.026233 | 0.31572 |
| RPL10A | 1.168513 | 8.932576 | 0.034685 | 0.359025 |
| HMGA1 | 1.168102 | 4.800242 | 0.036734 | 0.368727 |
| ISG20L2 | 1.166515 | 5.070902 | 0.026065 | 0.315355 |
| HPRT1 | 1.165291 | 5.039822 | 0.02807 | 0.326845 |
| TIMM50 | 1.160858 | 4.829306 | 0.032837 | 0.349092 |
| SIAH2 | 1.159768 | 5.143074 | 0.024176 | 0.30441 |
| RPL7L1 | 1.158828 | 7.780302 | 0.017426 | 0.259234 |
| SUMO3 | 1.158109 | 6.102884 | 0.035266 | 0.361854 |
| ABCF3 | 1.158075 | 5.198678 | 0.033638 | 0.353237 |
| FAM136A | 1.156764 | 5.875831 | 0.021095 | 0.285351 |
| TSC22D4 | 1.156285 | 4.666672 | 0.037331 | 0.370784 |
| SGSH | 1.155846 | 4.914821 | 0.041038 | 0.38918 |
| RPS24 | 1.155523 | 9.675871 | 0.048392 | 0.419082 |
| TMEM120B | 1.155365 | 4.69004 | 0.032958 | 0.349948 |
| APEX1 | 1.153971 | 6.51414 | 0.022202 | 0.293766 |
| ANLN | 1.153731 | 4.876832 | 0.04941 | 0.422933 |
| IVD | 1.153358 | 6.928417 | 0.027245 | 0.32281 |
| MARVELD2 | 1.152589 | 5.19388 | 0.028754 | 0.330292 |
| RPS29 | 1.149564 | 5.839865 | 0.032155 | 0.345699 |
| SNAPC4 | 1.149441 | 4.070055 | 0.047516 | 0.415773 |
| ZNF282 | 1.145585 | 4.962376 | 0.033302 | 0.351427 |
| CPT1A | 1.144425 | 6.968642 | 0.043699 | 0.399631 |
| TOB2 | 1.144243 | 6.663625 | 0.021897 | 0.291411 |
| HMGCL | 1.143457 | 4.595452 | 0.040295 | 0.38563 |
| DHCR7 | 1.142257 | 4.791677 | 0.030383 | 0.337636 |
| PRDX3 | 1.141993 | 6.917231 | 0.018173 | 0.264506 |
| UQCRFS1 | 1.141869 | 5.827952 | 0.025011 | 0.309191 |
| C1QBP | 1.141664 | 6.291945 | 0.04072 | 0.387608 |
| AZIN1 | 1.141163 | 8.432724 | 0.024037 | 0.303586 |
| PCNA | 1.138147 | 5.878618 | 0.023321 | 0.300474 |
| AKT1S1 | 1.137115 | 4.501256 | 0.048085 | 0.417896 |
| CCT7 | 1.134671 | 7.677326 | 0.019682 | 0.274795 |
| PSMD2 | 1.134658 | 6.818709 | 0.019894 | 0.276125 |
| RPL23 | 1.134331 | 9.5535 | 0.021413 | 0.287539 |
| RAD23A | 1.133274 | 5.8058 | 0.033542 | 0.352439 |
| PRR15L | 1.132585 | 5.256081 | 0.045502 | 0.407975 |
| AACS | 1.130237 | 5.558049 | 0.027703 | 0.325106 |
| EIF3B | 1.130057 | 7.553161 | 0.028314 | 0.327745 |
| FOXK1 | 1.129987 | 6.335386 | 0.023871 | 0.303138 |
| ZC3H15 | 1.128251 | 6.306243 | 0.034012 | 0.355535 |
| VIRMA | 1.125361 | 7.256832 | 0.024142 | 0.304316 |
| CBX3 | 1.12534 | 6.863546 | 0.030192 | 0.336714 |
| LRPPRC | 1.122262 | 8.7287 | 0.018918 | 0.269861 |
| PRDX1 | 1.121864 | 7.37346 | 0.021078 | 0.285351 |
| MCM3 | 1.12151 | 5.640645 | 0.024258 | 0.304992 |
| ARF5 | 1.120157 | 5.899931 | 0.027161 | 0.322305 |
| TRMT112 | 1.11955 | 5.457536 | 0.03708 | 0.369947 |
| LINC00665 | 1.116498 | 5.482204 | 0.039549 | 0.382541 |
| ZFAND1 | 1.114655 | 6.514658 | 0.028516 | 0.328584 |
| LRSAM1 | 1.113921 | 4.882038 | 0.03179 | 0.343495 |
| EIF2B5 | 1.113828 | 5.787801 | 0.0256 | 0.312483 |
| LCLAT1 | 1.112199 | 6.801201 | 0.037004 | 0.369765 |
| GATC | 1.111603 | 5.118585 | 0.035967 | 0.365401 |
| TMC4 | 1.11152 | 5.243102 | 0.028493 | 0.328428 |
| INSIG1 | 1.110421 | 5.681394 | 0.041266 | 0.38947 |
| PPP4C | 1.108801 | 4.581517 | 0.044553 | 0.403337 |
| DDX21 | 1.107224 | 7.449548 | 0.033194 | 0.35087 |
| FAM32A | 1.107213 | 5.14941 | 0.041015 | 0.38918 |
| SLC25A33 | 1.106418 | 4.774069 | 0.047421 | 0.415579 |
| RPS20 | 1.105242 | 9.720302 | 0.035422 | 0.362429 |
| ZNF7 | 1.103651 | 5.40195 | 0.036916 | 0.369765 |
| GPAT4 | 1.101459 | 5.972183 | 0.032837 | 0.349092 |
| NUP210 | 1.101017 | 5.878965 | 0.043097 | 0.398085 |
| PRPF31 | 1.099844 | 4.675768 | 0.047073 | 0.413528 |
| POFUT1 | 1.099147 | 6.067272 | 0.021933 | 0.291691 |
| COX7A2L | 1.09858 | 6.055632 | 0.031222 | 0.34136 |
| NT5C3A | 1.097326 | 5.803363 | 0.03665 | 0.368495 |
| RPL14 | 1.097175 | 8.021307 | 0.032667 | 0.348198 |
| MRRF | 1.095726 | 4.957129 | 0.034701 | 0.359025 |
| APH1A | 1.095268 | 5.665718 | 0.033144 | 0.350841 |
| ING5 | 1.094966 | 4.723878 | 0.041102 | 0.38947 |
| RBM19 | 1.093632 | 5.483748 | 0.032092 | 0.345125 |
| SRP9 | 1.09358 | 7.724019 | 0.0353 | 0.361948 |
| PGAM1 | 1.09304 | 6.328605 | 0.035201 | 0.36148 |
| CENPB | 1.092243 | 5.044732 | 0.049415 | 0.422933 |
| KRI1 | 1.090453 | 4.512449 | 0.048692 | 0.419721 |
| RPLP2 | 1.089479 | 7.657649 | 0.034409 | 0.358045 |
| SERF2 | 1.088688 | 5.291787 | 0.035671 | 0.363691 |
| AGO2 | 1.087903 | 5.61653 | 0.038712 | 0.377335 |
| LONP1 | 1.087468 | 5.312319 | 0.032593 | 0.348198 |
| MLXIP | 1.086202 | 6.69816 | 0.039756 | 0.383698 |
| TXN2 | 1.084177 | 5.28019 | 0.030934 | 0.340454 |
| TP53INP1 | 1.084055 | 8.55447 | 0.038845 | 0.378091 |
| HGS | 1.082997 | 5.306816 | 0.035329 | 0.362026 |
| NCAPG2 | 1.082314 | 5.202244 | 0.033877 | 0.354553 |
| RPL31 | 1.081442 | 8.151282 | 0.044562 | 0.403337 |
| TFRC | 1.079918 | 6.886853 | 0.032616 | 0.348198 |
| SNORD3A | 1.07915 | 7.651221 | 0.042047 | 0.393003 |
| MAN1B1 | 1.079142 | 5.663265 | 0.026993 | 0.321447 |
| AFMID | 1.077946 | 4.688384 | 0.048952 | 0.421202 |
| AP1M2 | 1.075508 | 5.57957 | 0.037596 | 0.372101 |
| CKB | 1.073534 | 6.819154 | 0.035608 | 0.363262 |
| WDR12 | 1.072788 | 5.569819 | 0.032581 | 0.348198 |
| KEAP1 | 1.072349 | 5.164567 | 0.043171 | 0.398114 |
| RNGTT | 1.071432 | 5.318675 | 0.042741 | 0.396784 |
| PRDX5 | 1.071259 | 5.432385 | 0.042486 | 0.395168 |
| RPL9 | 1.070161 | 9.132119 | 0.046417 | 0.411508 |
| FBL | 1.067904 | 6.091728 | 0.047244 | 0.414589 |
| NPRL3 | 1.067108 | 4.63196 | 0.046966 | 0.41299 |
| RPL3 | 1.063929 | 10.69586 | 0.042718 | 0.396679 |
| ANGEL1 | 1.063524 | 4.822102 | 0.042767 | 0.396824 |
| TANC1 | 1.062067 | 7.094035 | 0.041346 | 0.389788 |
| ASXL1 | 1.061315 | 6.827956 | 0.028169 | 0.327357 |
| CYB561 | 1.061038 | 6.985824 | 0.038879 | 0.378211 |
| NDUFS3 | 1.058761 | 4.73821 | 0.049628 | 0.423942 |
| DPY30 | 1.058034 | 4.991332 | 0.038626 | 0.376949 |
| KAT14 | 1.05585 | 4.803123 | 0.049587 | 0.423809 |
| NLN | 1.055078 | 6.018383 | 0.043235 | 0.398402 |
| ZNF251 | 1.054128 | 4.876469 | 0.043122 | 0.398085 |
| BTBD2 | 1.052674 | 5.141892 | 0.049408 | 0.422933 |
| AHSA1 | 1.051322 | 5.905149 | 0.03453 | 0.35887 |
| MTCH1 | 1.048503 | 6.565325 | 0.034747 | 0.359131 |
| PARP1 | 1.047106 | 7.03984 | 0.037295 | 0.370677 |
| TPD52L2 | 1.046577 | 6.137635 | 0.036608 | 0.368324 |
| EPCAM | 1.045503 | 7.676601 | 0.032251 | 0.346342 |
| DCAF12 | 1.041589 | 6.244843 | 0.046828 | 0.412812 |
| ODF2 | 1.040382 | 5.14464 | 0.042945 | 0.397671 |
| EYA3 | 1.036724 | 6.518772 | 0.043731 | 0.399687 |
| TRAPPC9 | 1.036209 | 5.873974 | 0.048303 | 0.418873 |
| GLUD1 | 1.036108 | 8.087067 | 0.034879 | 0.359131 |
| MTDH | 1.033362 | 7.853999 | 0.037391 | 0.370913 |
| ACTG1 | 1.030323 | 10.11822 | 0.031285 | 0.34136 |
| PRKCSH | 1.030202 | 6.901996 | 0.041571 | 0.390804 |
| ARL6IP1 | 1.030063 | 7.962451 | 0.031128 | 0.341168 |
| APLP2 | 1.027333 | 9.896103 | 0.038264 | 0.375414 |
| POLR1A | 1.026382 | 6.216035 | 0.040851 | 0.388265 |
| AP2A2 | 1.024959 | 5.834895 | 0.04282 | 0.397095 |
| BCL2L13 | 1.024566 | 6.456951 | 0.038485 | 0.37662 |
| TPI1 | 1.021057 | 7.664217 | 0.041008 | 0.38918 |
| WDR3 | 1.020034 | 6.117436 | 0.041303 | 0.389651 |
| TAF2 | 1.017225 | 6.54067 | 0.044213 | 0.402111 |
| YWHAZ | 1.016911 | 9.609471 | 0.046517 | 0.411743 |
| NUP205 | 1.015052 | 6.808479 | 0.040754 | 0.387773 |
| SOD1 | 1.014449 | 7.2038 | 0.039981 | 0.384868 |
| LTBR | 1.013025 | 5.739685 | 0.042089 | 0.393128 |
| NRBP2 | 1.010651 | 5.246626 | 0.044915 | 0.404608 |
| ACAD9 | 1.009603 | 5.49153 | 0.045792 | 0.40914 |
| HIST1H2BK | 1.009011 | 5.939965 | 0.040013 | 0.384928 |
| C6orf106 | 1.008399 | 6.565707 | 0.047589 | 0.416077 |
| UBB | 1.006545 | 6.751925 | 0.043137 | 0.398098 |
| TNPO2 | 1.001159 | 6.434416 | 0.040154 | 0.385263 |
| RAPGEF2 | -1.00209 | 6.435449 | 0.043013 | 0.397824 |
| AC093010.3 | -1.01775 | 5.534964 | 0.049261 | 0.422488 |
| TWSG1 | -1.02118 | 6.23364 | 0.039989 | 0.384868 |
| SH3BGRL | -1.0291 | 6.023049 | 0.040264 | 0.38563 |
| SMARCA1 | -1.03683 | 6.399221 | 0.044836 | 0.404217 |
| GALNT10 | -1.03971 | 5.631617 | 0.039591 | 0.382541 |
| NR2F2 | -1.04117 | 6.707856 | 0.028341 | 0.327753 |
| CPLANE1 | -1.04689 | 7.530746 | 0.043174 | 0.398114 |
| C1GALT1 | -1.0524 | 4.741537 | 0.04924 | 0.422412 |
| TANC2 | -1.05452 | 6.428042 | 0.044786 | 0.404116 |
| CLIC4 | -1.05993 | 7.832035 | 0.040447 | 0.386241 |
| VPS36 | -1.06081 | 5.981436 | 0.04298 | 0.397717 |
| ANO6 | -1.06113 | 6.20036 | 0.041166 | 0.38947 |
| PHACTR2 | -1.07086 | 6.649865 | 0.03697 | 0.369765 |
| SESTD1 | -1.07748 | 5.91584 | 0.029408 | 0.332645 |
| MTUS1 | -1.07787 | 7.025173 | 0.040349 | 0.385739 |
| TP53INP2 | -1.08062 | 4.789534 | 0.039037 | 0.379204 |
| CCDC66 | -1.08196 | 5.507768 | 0.041497 | 0.390404 |
| FSTL1 | -1.08911 | 8.942433 | 0.035841 | 0.364665 |
| EPB41L2 | -1.09 | 6.04755 | 0.040686 | 0.387608 |
| ARHGEF17 | -1.09264 | 4.806683 | 0.041929 | 0.392642 |
| FER | -1.09496 | 6.05657 | 0.0262 | 0.315552 |
| GPR155 | -1.09505 | 4.80721 | 0.044683 | 0.403967 |
| CCDC88A | -1.10165 | 5.597195 | 0.03295 | 0.349948 |
| PKD2 | -1.10286 | 5.953495 | 0.027889 | 0.326498 |
| ZNRF2 | -1.10943 | 5.412179 | 0.028983 | 0.331081 |
| STK17B | -1.113 | 6.160006 | 0.026171 | 0.315552 |
| PALLD | -1.12652 | 7.9455 | 0.043387 | 0.398699 |
| STRADB | -1.12978 | 4.596064 | 0.034998 | 0.359897 |
| PARD3B | -1.13038 | 5.978538 | 0.028936 | 0.331013 |
| FAM111A | -1.13134 | 7.361038 | 0.044344 | 0.402709 |
| SCAPER | -1.13373 | 5.548202 | 0.027232 | 0.322774 |
| BEX4 | -1.13399 | 4.691785 | 0.039457 | 0.382098 |
| GPR180 | -1.13441 | 4.998913 | 0.041065 | 0.389329 |
| CAMK2D | -1.1416 | 7.115119 | 0.023974 | 0.303528 |
| RORA | -1.14246 | 6.247017 | 0.019584 | 0.274206 |
| DOCK11 | -1.14606 | 5.375758 | 0.042952 | 0.397671 |
| PTPRG | -1.14638 | 6.162592 | 0.031229 | 0.34136 |
| LITAF | -1.14683 | 6.515792 | 0.017057 | 0.256745 |
| MXRA7 | -1.1582 | 6.485083 | 0.028614 | 0.329268 |
| ARHGAP31 | -1.16297 | 4.768545 | 0.028254 | 0.327574 |
| LPGAT1 | -1.16308 | 6.52189 | 0.022304 | 0.294438 |
| PHF11 | -1.16403 | 4.341385 | 0.038525 | 0.376729 |
| SPP1 | -1.16649 | 6.258495 | 0.039064 | 0.379361 |
| SEMA5A | -1.17316 | 5.195219 | 0.021292 | 0.286718 |
| QSER1 | -1.17651 | 6.187042 | 0.025222 | 0.310506 |
| IL13RA1 | -1.17761 | 6.682584 | 0.029681 | 0.33394 |
| MYLK | -1.17905 | 8.548295 | 0.045219 | 0.406287 |
| ITGA1 | -1.18048 | 7.407462 | 0.021498 | 0.28822 |
| PDGFRB | -1.18068 | 6.297377 | 0.018849 | 0.26935 |
| SGPP1 | -1.18608 | 4.566261 | 0.043124 | 0.398085 |
| COL15A1 | -1.18768 | 6.305123 | 0.022596 | 0.296149 |
| ARHGEF37 | -1.18916 | 5.922757 | 0.027999 | 0.326784 |
| EIF4E3 | -1.18961 | 5.60687 | 0.019688 | 0.274795 |
| AAK1 | -1.1905 | 6.325668 | 0.044183 | 0.402026 |
| DPYD | -1.19448 | 4.812699 | 0.029273 | 0.332315 |
| FOXO1 | -1.19466 | 4.871612 | 0.021996 | 0.291829 |
| TCF4 | -1.1968 | 7.028674 | 0.013053 | 0.223646 |
| TIMP3 | -1.19778 | 7.627464 | 0.046745 | 0.412445 |
| ABCA5 | -1.1995 | 6.55961 | 0.023852 | 0.303138 |
| MXRA8 | -1.20169 | 4.281646 | 0.039784 | 0.383753 |
| ITGA9 | -1.20584 | 5.0305 | 0.04795 | 0.417501 |
| ARHGEF40 | -1.20762 | 4.615911 | 0.034201 | 0.35696 |
| PLAT | -1.20801 | 4.402091 | 0.034207 | 0.35696 |
| LMO4 | -1.20877 | 4.538815 | 0.029352 | 0.332524 |
| AHNAK2 | -1.21464 | 4.992494 | 0.023424 | 0.30089 |
| CALD1 | -1.21546 | 9.569177 | 0.017551 | 0.260076 |
| FAS | -1.21676 | 4.494588 | 0.045896 | 0.409263 |
| NR3C1 | -1.2171 | 6.731108 | 0.032391 | 0.347061 |
| WDFY2 | -1.22323 | 3.82991 | 0.049686 | 0.424019 |
| JAK2 | -1.22471 | 5.790629 | 0.036599 | 0.368324 |
| ZDHHC14 | -1.22551 | 4.930022 | 0.036073 | 0.365594 |
| SERPINF1 | -1.22696 | 5.5236 | 0.031044 | 0.340788 |
| ANKRD36B | -1.23314 | 6.415983 | 0.041747 | 0.391688 |
| ENPP4 | -1.23353 | 6.426726 | 0.018235 | 0.26515 |
| CYP4V2 | -1.23481 | 5.270876 | 0.028849 | 0.330485 |
| F2R | -1.23574 | 5.189749 | 0.020571 | 0.281508 |
| SEMA3C | -1.23592 | 7.290007 | 0.03538 | 0.362338 |
| ZNF552 | -1.23732 | 5.603491 | 0.034211 | 0.35696 |
| UFSP2 | -1.24087 | 4.536884 | 0.030319 | 0.337408 |
| AHNAK | -1.24149 | 11.53168 | 0.038249 | 0.375414 |
| TMTC2 | -1.24409 | 5.802652 | 0.015922 | 0.246503 |
| TMEM30B | -1.24431 | 4.951118 | 0.04378 | 0.399844 |
| STOM | -1.24487 | 5.588715 | 0.028329 | 0.327745 |
| ATP2B4 | -1.24497 | 6.666284 | 0.029908 | 0.33539 |
| CAPN2 | -1.25045 | 7.02814 | 0.014384 | 0.233916 |
| ERAP1 | -1.25169 | 5.904199 | 0.012045 | 0.214711 |
| SLFN11 | -1.25239 | 4.351976 | 0.042586 | 0.39588 |
| RNF144A | -1.25893 | 5.846791 | 0.013285 | 0.225242 |
| KITLG | -1.2605 | 5.11576 | 0.025686 | 0.313156 |
| PELI2 | -1.26142 | 4.642714 | 0.041216 | 0.38947 |
| KIAA0040 | -1.26243 | 5.046376 | 0.015534 | 0.243401 |
| TNFSF10 | -1.26284 | 6.685666 | 0.027011 | 0.321485 |
| CEP112 | -1.26453 | 4.442778 | 0.049504 | 0.423304 |
| TGFBR2 | -1.26491 | 6.482678 | 0.015282 | 0.241461 |
| ZNF483 | -1.27169 | 5.109171 | 0.029455 | 0.332762 |
| MAGI3 | -1.28359 | 6.252649 | 0.017551 | 0.260076 |
| SORL1 | -1.28502 | 7.780111 | 0.010391 | 0.198761 |
| SLCO2A1 | -1.28784 | 3.916082 | 0.034063 | 0.355846 |
| CNN2 | -1.28992 | 4.769166 | 0.029422 | 0.332657 |
| LRRC8C | -1.29511 | 4.238992 | 0.024492 | 0.306233 |
| TBC1D1 | -1.2961 | 5.455719 | 0.014944 | 0.23853 |
| TSC22D3 | -1.29752 | 7.01604 | 0.044759 | 0.404116 |
| PMP22 | -1.29931 | 4.612468 | 0.034228 | 0.357031 |
| SEC14L2 | -1.29962 | 4.803167 | 0.044781 | 0.404116 |
| DAAM2 | -1.30526 | 3.907744 | 0.036476 | 0.367528 |
| NID1 | -1.30556 | 5.539885 | 0.012813 | 0.220838 |
| AOC3 | -1.30885 | 4.210217 | 0.031374 | 0.341786 |
| PELI1 | -1.30971 | 4.832579 | 0.015706 | 0.244777 |
| PODN | -1.31224 | 4.346044 | 0.046898 | 0.412944 |
| IL33 | -1.31591 | 4.260435 | 0.037992 | 0.374027 |
| MCC | -1.31609 | 5.35593 | 0.012846 | 0.221282 |
| LYST | -1.31649 | 6.626459 | 0.006147 | 0.154356 |
| SNX24 | -1.32427 | 3.624926 | 0.041914 | 0.392608 |
| LAMA2 | -1.32491 | 6.505059 | 0.01982 | 0.275737 |
| EDIL3 | -1.32643 | 4.921362 | 0.012771 | 0.220443 |
| NEDD4 | -1.32687 | 5.426522 | 0.016311 | 0.250234 |
| PDGFD | -1.32802 | 4.197626 | 0.028441 | 0.328042 |
| UACA | -1.33116 | 6.989474 | 0.00611 | 0.153975 |
| EOGT | -1.33306 | 5.190004 | 0.02088 | 0.283934 |
| HK2 | -1.33429 | 6.246527 | 0.026907 | 0.32091 |
| PEAK1 | -1.33738 | 6.589951 | 0.005934 | 0.151082 |
| CA5B | -1.34012 | 4.690773 | 0.02899 | 0.331081 |
| CTBS | -1.34222 | 5.010501 | 0.04216 | 0.393308 |
| NCALD | -1.34413 | 4.451091 | 0.022396 | 0.295217 |
| MFGE8 | -1.34983 | 5.186905 | 0.036081 | 0.365594 |
| APOBEC3C | -1.3507 | 3.965197 | 0.030744 | 0.339401 |
| LGALS3 | -1.35133 | 5.520524 | 0.011866 | 0.213064 |
| ODF2L | -1.35191 | 5.620769 | 0.014519 | 0.234768 |
| ATP2C2 | -1.35509 | 3.808976 | 0.036065 | 0.365594 |
| RBPMS | -1.36123 | 5.655572 | 0.035687 | 0.363744 |
| RGPD8 | -1.36416 | 3.054427 | 0.045831 | 0.40914 |
| HIVEP1 | -1.36594 | 7.320074 | 0.013931 | 0.230831 |
| ZNF655 | -1.36607 | 5.400386 | 0.030719 | 0.339346 |
| RGL1 | -1.36687 | 5.077198 | 0.019158 | 0.271007 |
| PLCE1 | -1.36798 | 5.218676 | 0.006977 | 0.161713 |
| MYL9 | -1.37005 | 5.243494 | 0.048579 | 0.419675 |
| ARMCX2 | -1.37058 | 4.605724 | 0.033187 | 0.35087 |
| ETS1 | -1.37197 | 5.932812 | 0.006881 | 0.160585 |
| MLLT3 | -1.37487 | 5.262213 | 0.010508 | 0.199191 |
| RNF152 | -1.37574 | 5.825902 | 0.033464 | 0.352373 |
| CREB5 | -1.37722 | 3.275708 | 0.04971 | 0.424091 |
| ADAMTS9 | -1.37771 | 4.068741 | 0.018787 | 0.26912 |
| FBLN5 | -1.38184 | 4.986366 | 0.037281 | 0.370677 |
| PLN | -1.38338 | 4.568333 | 0.041376 | 0.389808 |
| DZIP1 | -1.38392 | 3.947263 | 0.037952 | 0.373747 |
| FAM69A | -1.38528 | 3.547592 | 0.04649 | 0.411743 |
| TEK | -1.38752 | 3.306901 | 0.043375 | 0.398699 |
| FAM129A | -1.38808 | 7.863168 | 0.039498 | 0.382391 |
| PYGO1 | -1.38951 | 5.50957 | 0.005672 | 0.147249 |
| HACD4 | -1.38955 | 4.410291 | 0.027109 | 0.321972 |
| ATXN1 | -1.39117 | 6.647192 | 0.007204 | 0.164099 |
| FBN1 | -1.39359 | 7.372434 | 0.01046 | 0.199044 |
| GCH1 | -1.39744 | 3.545051 | 0.043388 | 0.398699 |
| PHLDB1 | -1.39815 | 5.634036 | 0.010074 | 0.194673 |
| MYOF | -1.40187 | 6.283885 | 0.006729 | 0.159509 |
| PRKD3 | -1.40318 | 5.719157 | 0.013264 | 0.225242 |
| ZNF615 | -1.40363 | 6.80557 | 0.017169 | 0.257633 |
| NYNRIN | -1.40401 | 3.557094 | 0.024025 | 0.303586 |
| INHBA | -1.40414 | 6.234017 | 0.047613 | 0.41615 |
| SCD5 | -1.40514 | 3.587244 | 0.030852 | 0.340084 |
| PTPRB | -1.40551 | 5.757393 | 0.008056 | 0.173701 |
| F8 | -1.40819 | 5.025037 | 0.009592 | 0.191135 |
| FAT4 | -1.40837 | 6.275378 | 0.039921 | 0.38453 |
| CCPG1 | -1.40867 | 4.482221 | 0.018928 | 0.269893 |
| TRPM4 | -1.41305 | 6.532668 | 0.021072 | 0.285351 |
| WNT2B | -1.4137 | 4.276204 | 0.018351 | 0.266191 |
| ZNF577 | -1.41708 | 6.891033 | 0.029678 | 0.33394 |
| CLGN | -1.42015 | 4.561853 | 0.027414 | 0.323601 |
| RUFY3 | -1.42471 | 5.990222 | 0.004845 | 0.135752 |
| DGKA | -1.42479 | 3.809317 | 0.036195 | 0.366191 |
| TLN2 | -1.42515 | 5.175853 | 0.031955 | 0.3443 |
| PCOLCE | -1.42737 | 3.083825 | 0.034675 | 0.359025 |
| MMP2 | -1.42848 | 6.467515 | 0.033198 | 0.35087 |
| SLC22A5 | -1.4288 | 5.747726 | 0.043501 | 0.399117 |
| LOX | -1.42988 | 4.206901 | 0.014753 | 0.237128 |
| DLC1 | -1.43082 | 5.447863 | 0.005506 | 0.14526 |
| FLRT2 | -1.43272 | 4.882604 | 0.011644 | 0.211403 |
| TMTC1 | -1.43305 | 4.525912 | 0.036756 | 0.368833 |
| ENO2 | -1.43334 | 3.141858 | 0.035378 | 0.362338 |
| VEGFA | -1.43346 | 6.761451 | 0.005544 | 0.145325 |
| C1R | -1.43403 | 6.170943 | 0.047673 | 0.416358 |
| DGKH | -1.43503 | 5.600772 | 0.011137 | 0.206134 |
| ABCC3 | -1.43684 | 3.019608 | 0.041773 | 0.391825 |
| C1QTNF1 | -1.44002 | 2.802085 | 0.044624 | 0.403687 |
| FBLN2 | -1.44097 | 4.026964 | 0.045874 | 0.409232 |
| LTBP4 | -1.44192 | 5.577997 | 0.020214 | 0.278396 |
| NAV3 | -1.44229 | 5.015675 | 0.015114 | 0.239795 |
| DCLK2 | -1.44281 | 3.420011 | 0.037435 | 0.370913 |
| ZEB2 | -1.4432 | 6.558712 | 0.003041 | 0.107337 |
| GPRASP1 | -1.44657 | 4.912181 | 0.009752 | 0.192398 |
| SLC22A15 | -1.44678 | 2.686797 | 0.038254 | 0.375414 |
| ITGA6 | -1.44731 | 6.818486 | 0.004071 | 0.123754 |
| PRKG1 | -1.44784 | 4.821939 | 0.009528 | 0.190831 |
| KCTD1 | -1.44895 | 4.652678 | 0.018965 | 0.270012 |
| PBX1 | -1.45089 | 7.173737 | 0.00661 | 0.158657 |
| CTSK | -1.45161 | 5.521861 | 0.020357 | 0.279814 |
| ARL10 | -1.45474 | 3.786702 | 0.019157 | 0.271007 |
| ANGPT1 | -1.45564 | 3.586848 | 0.045679 | 0.408706 |
| FBXL7 | -1.45889 | 3.858354 | 0.036505 | 0.367607 |
| SPOCK2 | -1.46041 | 3.462706 | 0.031158 | 0.341192 |
| RUNX1 | -1.46176 | 5.56887 | 0.005424 | 0.143913 |
| SC5D | -1.46295 | 7.687242 | 0.012646 | 0.218938 |
| S100A6 | -1.46359 | 5.615696 | 0.024043 | 0.303586 |
| WIPF1 | -1.46478 | 5.269464 | 0.00917 | 0.187494 |
| ACER2 | -1.46678 | 3.890203 | 0.044683 | 0.403967 |
| STXBP6 | -1.46937 | 4.850206 | 0.028784 | 0.330335 |
| ID4 | -1.46987 | 4.563603 | 0.00764 | 0.169424 |
| PDGFC | -1.47059 | 4.99199 | 0.019109 | 0.270672 |
| RHOBTB1 | -1.47108 | 4.688177 | 0.014434 | 0.234306 |
| GXYLT2 | -1.47137 | 4.287796 | 0.018655 | 0.268442 |
| HCP5 | -1.47261 | 3.549024 | 0.048155 | 0.418134 |
| CAV1 | -1.47471 | 5.413366 | 0.007737 | 0.170694 |
| TNS1 | -1.47517 | 8.181167 | 0.012641 | 0.218938 |
| SERPINB8 | -1.47565 | 2.13516 | 0.046391 | 0.411435 |
| IKZF1 | -1.47573 | 3.844282 | 0.027176 | 0.322329 |
| BNC2 | -1.47643 | 4.631482 | 0.015592 | 0.243775 |
| RECK | -1.47854 | 4.147806 | 0.015134 | 0.240005 |
| CYBRD1 | -1.47872 | 7.228224 | 0.00733 | 0.165434 |
| DHRS7 | -1.48008 | 7.605726 | 0.036361 | 0.367018 |
| PPP4R1L | -1.48307 | 2.405744 | 0.045826 | 0.40914 |
| CDK6 | -1.48392 | 5.639683 | 0.004158 | 0.125426 |
| LPCAT2 | -1.48398 | 4.440749 | 0.009235 | 0.187736 |
| UBASH3B | -1.48563 | 2.853652 | 0.028998 | 0.331081 |
| RHOJ | -1.48679 | 3.354223 | 0.026669 | 0.319293 |
| PDE8A | -1.48878 | 5.406334 | 0.003669 | 0.118207 |
| ST3GAL6 | -1.48914 | 3.698526 | 0.021381 | 0.287539 |
| SCRG1 | -1.49061 | 2.798444 | 0.038606 | 0.376939 |
| MFAP4 | -1.49174 | 4.985063 | 0.027611 | 0.32475 |
| CDH6 | -1.49326 | 3.906131 | 0.023612 | 0.301561 |
| TIMP2 | -1.49357 | 6.889837 | 0.005426 | 0.143913 |
| ZNF879 | -1.4963 | 3.01536 | 0.028172 | 0.327357 |
| LRMP | -1.49856 | 2.119689 | 0.041864 | 0.392352 |
| ADGRL4 | -1.49949 | 4.297646 | 0.011484 | 0.209702 |
| ITIH5 | -1.50057 | 4.271584 | 0.026127 | 0.315499 |
| OAF | -1.50067 | 3.14505 | 0.029535 | 0.333278 |
| TARSL2 | -1.5014 | 4.786609 | 0.006509 | 0.157226 |
| SYTL4 | -1.5041 | 4.612482 | 0.025031 | 0.309191 |
| DPYSL3 | -1.50452 | 8.08763 | 0.003623 | 0.117186 |
| LHFPL6 | -1.50526 | 5.109163 | 0.008201 | 0.175507 |
| NHSL2 | -1.50529 | 2.653476 | 0.046854 | 0.412812 |
| ITGA3 | -1.50648 | 4.160993 | 0.012228 | 0.215723 |
| PTGS2 | -1.50684 | 5.284177 | 0.048082 | 0.417896 |
| GLIPR1 | -1.50749 | 4.510833 | 0.007617 | 0.169135 |
| PPL | -1.50834 | 5.171111 | 0.01214 | 0.215026 |
| NHS | -1.50971 | 3.969371 | 0.014665 | 0.23621 |
| LAMA4 | -1.50983 | 6.30093 | 0.003746 | 0.119322 |
| ANXA2 | -1.51734 | 7.40415 | 0.00335 | 0.112578 |
| ATP2A3 | -1.51821 | 3.626087 | 0.029053 | 0.331081 |
| TNS2 | -1.51835 | 5.251247 | 0.003449 | 0.114336 |
| TSPAN5 | -1.51971 | 3.477232 | 0.017402 | 0.259234 |
| MYCT1 | -1.52002 | 2.855761 | 0.035994 | 0.365428 |
| CSRP1 | -1.52104 | 7.642539 | 0.008555 | 0.180386 |
| ST6GALNAC3 | -1.52373 | 2.111508 | 0.04242 | 0.394766 |
| AKAP7 | -1.52452 | 4.464045 | 0.008036 | 0.173474 |
| HERC3 | -1.52581 | 6.906524 | 0.005536 | 0.145325 |
| GJC1 | -1.52782 | 4.761196 | 0.008796 | 0.183581 |
| PPIC | -1.52927 | 3.20463 | 0.026546 | 0.318458 |
| IGFBP3 | -1.53056 | 7.029682 | 0.009611 | 0.19132 |
| SSBP2 | -1.53066 | 4.854967 | 0.023995 | 0.303555 |
| LDB2 | -1.53362 | 3.860758 | 0.011536 | 0.210326 |
| AGPAT4 | -1.53522 | 3.465598 | 0.018006 | 0.263517 |
| MRVI1 | -1.5355 | 5.985309 | 0.014692 | 0.236481 |
| FHL2 | -1.53597 | 4.68149 | 0.022238 | 0.294071 |
| GAS7 | -1.53684 | 4.813068 | 0.008989 | 0.185278 |
| RMDN2 | -1.5386 | 3.299259 | 0.024911 | 0.308571 |
| MEF2C | -1.54416 | 5.415254 | 0.004199 | 0.125956 |
| VSTM4 | -1.54431 | 4.574573 | 0.017135 | 0.257469 |
| SYNE1 | -1.54786 | 7.645653 | 0.004063 | 0.123754 |
| PRRG4 | -1.54848 | 4.259139 | 0.022503 | 0.296001 |
| CDC42EP3 | -1.54907 | 5.955115 | 0.011434 | 0.208908 |
| RNU1-27P | -1.5526 | 8.457617 | 0.020772 | 0.283243 |
| FRMD4B | -1.55274 | 5.779019 | 0.002034 | 0.083736 |
| PCDHB11 | -1.55285 | 3.378072 | 0.036401 | 0.367143 |
| MACC1 | -1.55378 | 3.988507 | 0.024415 | 0.306072 |
| KLHL3 | -1.55639 | 2.628652 | 0.046931 | 0.41299 |
| SYNE2 | -1.5582 | 8.476904 | 0.004975 | 0.137306 |
| AP002026.1 | -1.55994 | 3.434295 | 0.021393 | 0.287539 |
| TMLHE | -1.56181 | 2.357703 | 0.031838 | 0.343867 |
| SULT2B1 | -1.56204 | 1.930148 | 0.044426 | 0.402848 |
| GPR82 | -1.56246 | 2.071968 | 0.03553 | 0.363109 |
| DIXDC1 | -1.56285 | 5.576019 | 0.012791 | 0.220564 |
| ZNF613 | -1.56418 | 5.958908 | 0.010764 | 0.201716 |
| GNG2 | -1.56493 | 3.652298 | 0.014094 | 0.231342 |
| WLS | -1.56673 | 6.446774 | 0.009964 | 0.193967 |
| DACT1 | -1.56809 | 4.090381 | 0.030525 | 0.33834 |
| TNC | -1.56833 | 6.802533 | 0.01516 | 0.240123 |
| ADGRA2 | -1.56862 | 4.787837 | 0.005894 | 0.15052 |
| MYOM2 | -1.56899 | 2.046491 | 0.044269 | 0.402383 |
| TFPI | -1.56914 | 4.472702 | 0.007092 | 0.162963 |
| SAR1B | -1.57081 | 7.388294 | 0.006403 | 0.156384 |
| GSN | -1.57099 | 7.220968 | 0.004956 | 0.137306 |
| RARRES1 | -1.57161 | 3.888379 | 0.013576 | 0.227808 |
| AFAP1L2 | -1.57455 | 4.25943 | 0.008919 | 0.184267 |
| MAP1A | -1.57695 | 4.759551 | 0.005395 | 0.143501 |
| LRP4 | -1.5786 | 2.975157 | 0.034343 | 0.357577 |
| MBNL3 | -1.58045 | 5.356876 | 0.004246 | 0.126634 |
| SULF2 | -1.58117 | 4.281969 | 0.012566 | 0.218654 |
| MSRB3 | -1.58238 | 6.125086 | 0.011085 | 0.205709 |
| ADAMTS5 | -1.58255 | 3.888701 | 0.011927 | 0.213475 |
| CDH11 | -1.58581 | 6.637116 | 0.011611 | 0.211171 |
| AF131215.6 | -1.58599 | 2.880851 | 0.039136 | 0.379851 |
| HEPH | -1.59562 | 4.201272 | 0.017282 | 0.258208 |
| PRUNE2 | -1.5992 | 8.668421 | 0.012217 | 0.215647 |
| CD44 | -1.60001 | 7.681313 | 0.012599 | 0.218706 |
| BMP4 | -1.60025 | 3.668982 | 0.042299 | 0.394064 |
| CAV2 | -1.60183 | 3.979376 | 0.009958 | 0.193967 |
| ST8SIA1 | -1.60555 | 3.46787 | 0.013643 | 0.228351 |
| DST | -1.60676 | 9.177388 | 0.003348 | 0.112578 |
| LTBP1 | -1.61127 | 7.07069 | 0.021896 | 0.291411 |
| MAP6 | -1.61258 | 3.207988 | 0.018804 | 0.26912 |
| ZNF781 | -1.61408 | 1.862725 | 0.046633 | 0.411986 |
| AHR | -1.6166 | 6.645078 | 0.001979 | 0.082451 |
| GVINP1 | -1.61775 | 3.726521 | 0.022981 | 0.299084 |
| ARHGAP29 | -1.61799 | 7.031098 | 0.004981 | 0.137306 |
| SH3PXD2A | -1.61956 | 6.52949 | 0.001844 | 0.078296 |
| AC116366.1 | -1.62103 | 2.49589 | 0.0242 | 0.304598 |
| FLT1 | -1.62374 | 5.305489 | 0.00487 | 0.136005 |
| NREP | -1.62834 | 6.266146 | 0.001404 | 0.068007 |
| LIMCH1 | -1.62849 | 8.752012 | 0.005907 | 0.150552 |
| FHL1 | -1.62868 | 6.656166 | 0.013022 | 0.223424 |
| VIM | -1.62882 | 7.719603 | 0.001859 | 0.07854 |
| SLFN13 | -1.63079 | 1.755452 | 0.037046 | 0.369936 |
| IL15 | -1.63316 | 2.060949 | 0.034306 | 0.3573 |
| C1QTNF7 | -1.63465 | 3.886729 | 0.037729 | 0.37283 |
| TMEM232 | -1.63472 | 2.325391 | 0.041235 | 0.38947 |
| VCAN | -1.63623 | 8.48162 | 0.006178 | 0.154435 |
| CDKN1C | -1.63637 | 1.684405 | 0.035746 | 0.363909 |
| AMY2B | -1.63983 | 3.459449 | 0.016753 | 0.254471 |
| ATP1B1 | -1.64241 | 5.377616 | 0.001802 | 0.077275 |
| SYNE3 | -1.64384 | 1.987234 | 0.03854 | 0.376729 |
| EPHA3 | -1.64476 | 6.845639 | 0.018569 | 0.267662 |
| TMEM45B | -1.64478 | 3.799509 | 0.040863 | 0.388269 |
| CFH | -1.64708 | 6.107802 | 0.004493 | 0.13112 |
| LARP6 | -1.64714 | 3.245295 | 0.018621 | 0.268146 |
| MSRA | -1.64919 | 2.581622 | 0.025271 | 0.310622 |
| DDR2 | -1.6501 | 7.031816 | 0.001925 | 0.080637 |
| RIMS1 | -1.65113 | 4.995692 | 0.007324 | 0.165406 |
| BTC | -1.65213 | 3.143288 | 0.017233 | 0.257899 |
| ENPP1 | -1.65291 | 5.21773 | 0.003957 | 0.122456 |
| CELF2 | -1.65297 | 4.956634 | 0.003191 | 0.109941 |
| KCNK6 | -1.65497 | 1.879301 | 0.035241 | 0.361772 |
| ZNF365 | -1.65759 | 2.390243 | 0.048543 | 0.419675 |
| DCLK1 | -1.6589 | 4.030335 | 0.008507 | 0.179487 |
| QPCT | -1.66004 | 1.628621 | 0.03469 | 0.359025 |
| GHR | -1.66354 | 4.428827 | 0.012262 | 0.216102 |
| ANGPT2 | -1.66435 | 3.626682 | 0.017129 | 0.257469 |
| MOB3B | -1.66597 | 3.169328 | 0.016402 | 0.250772 |
| PLSCR4 | -1.66597 | 4.250426 | 0.014855 | 0.237878 |
| CCND2 | -1.66769 | 7.237041 | 0.004597 | 0.132259 |
| NDRG2 | -1.6691 | 4.893144 | 0.001897 | 0.079639 |
| TMEM173 | -1.67063 | 3.094192 | 0.013868 | 0.230292 |
| EPHB6 | -1.67121 | 2.324092 | 0.025242 | 0.310506 |
| GBP3 | -1.67183 | 4.852386 | 0.018544 | 0.26763 |
| AL359220.1 | -1.67334 | 1.616604 | 0.044907 | 0.404608 |
| EPB41L3 | -1.6773 | 4.920472 | 0.002978 | 0.10643 |
| F2RL2 | -1.68283 | 2.857889 | 0.021433 | 0.287693 |
| NES | -1.68321 | 5.33777 | 0.001629 | 0.073412 |
| GBP4 | -1.68611 | 4.889934 | 0.006076 | 0.153341 |
| CDKN2B | -1.68842 | 3.857928 | 0.013301 | 0.225288 |
| LAMP5 | -1.68885 | 2.080611 | 0.045219 | 0.406287 |
| SLC9A9 | -1.68912 | 4.529411 | 0.005265 | 0.141733 |
| PCDHGA7 | -1.68947 | 2.984127 | 0.03203 | 0.344575 |
| CCDC9B | -1.69225 | 3.888077 | 0.008829 | 0.183581 |
| ANO1 | -1.69345 | 5.32843 | 0.004699 | 0.133627 |
| GPR135 | -1.69642 | 3.192183 | 0.031236 | 0.34136 |
| LRRK2 | -1.70278 | 5.046771 | 0.003456 | 0.11447 |
| PTPRM | -1.70411 | 6.570643 | 0.006726 | 0.159509 |
| CACNA1C | -1.70528 | 4.526664 | 0.006156 | 0.154356 |
| NT5E | -1.70665 | 4.29596 | 0.008017 | 0.173474 |
| FILIP1L | -1.70712 | 6.048108 | 0.003585 | 0.116728 |
| FAM13A-AS1 | -1.70785 | 4.309119 | 0.016057 | 0.247767 |
| RASD2 | -1.70987 | 2.667222 | 0.021406 | 0.287539 |
| MEIS1 | -1.7101 | 5.343753 | 0.017187 | 0.257799 |
| PDE5A | -1.71199 | 6.98701 | 0.003164 | 0.109723 |
| MEIS2 | -1.71315 | 4.531105 | 0.01645 | 0.251152 |
| SIX1 | -1.71487 | 4.519268 | 0.027068 | 0.321914 |
| CPNE8 | -1.71547 | 3.143652 | 0.010567 | 0.199443 |
| CD96 | -1.71556 | 2.28202 | 0.048778 | 0.420162 |
| KIAA0513 | -1.71828 | 4.344675 | 0.002407 | 0.093624 |
| MEG3 | -1.71948 | 4.711194 | 0.003918 | 0.121993 |
| GPR161 | -1.72079 | 2.3038 | 0.028047 | 0.32679 |
| SIDT1 | -1.72126 | 4.519464 | 0.030348 | 0.337459 |
| OLFML2A | -1.72186 | 4.015098 | 0.004023 | 0.123444 |
| ARHGAP20 | -1.72333 | 2.521849 | 0.019801 | 0.275678 |
| CCDC71L | -1.72543 | 3.186102 | 0.01405 | 0.231059 |
| COL8A1 | -1.72688 | 6.788017 | 0.005371 | 0.143255 |
| BCAS1 | -1.72694 | 5.414017 | 0.02906 | 0.331081 |
| TRO | -1.72848 | 1.860286 | 0.024521 | 0.306233 |
| CSRNP3 | -1.72887 | 4.30385 | 0.023509 | 0.301106 |
| L3MBTL4 | -1.72976 | 2.123074 | 0.024525 | 0.306233 |
| ATP8B2 | -1.7321 | 6.55063 | 0.001265 | 0.063891 |
| RIC3 | -1.73367 | 4.588311 | 0.010568 | 0.199443 |
| LINC00654 | -1.7338 | 1.90077 | 0.028083 | 0.326878 |
| SP140L | -1.73434 | 3.237491 | 0.02183 | 0.29109 |
| TSHZ2 | -1.73453 | 5.552417 | 0.001157 | 0.060965 |
| SAMD5 | -1.73487 | 4.772516 | 0.012366 | 0.216941 |
| KCNMA1 | -1.73496 | 6.303603 | 0.005099 | 0.139115 |
| HS3ST1 | -1.73507 | 3.625013 | 0.017067 | 0.256745 |
| ANO2 | -1.7366 | 1.691427 | 0.045555 | 0.408025 |
| PLD1 | -1.7409 | 4.044119 | 0.004489 | 0.13112 |
| IRAK3 | -1.74189 | 4.143357 | 0.003232 | 0.110574 |
| ADCY5 | -1.74293 | 3.65759 | 0.017299 | 0.258351 |
| FRMD6 | -1.74316 | 4.853405 | 0.005124 | 0.139679 |
| KLF5 | -1.74526 | 4.397964 | 0.015328 | 0.241854 |
| AC105942.1 | -1.74779 | 1.161143 | 0.043015 | 0.397824 |
| WNT4 | -1.75285 | 2.570649 | 0.017509 | 0.259794 |
| ACTA2 | -1.7546 | 7.430495 | 0.007003 | 0.161982 |
| MAP3K20 | -1.75631 | 6.725287 | 0.003705 | 0.118819 |
| STARD4-AS1 | -1.75659 | 3.66293 | 0.009271 | 0.187736 |
| PITPNM3 | -1.75852 | 2.606782 | 0.029164 | 0.331774 |
| FMN1 | -1.76049 | 5.011701 | 0.001718 | 0.074807 |
| TPM1-AS | -1.76113 | 3.903986 | 0.012624 | 0.218895 |
| HECW2 | -1.7617 | 3.479339 | 0.016136 | 0.24855 |
| BCL2 | -1.76426 | 4.784308 | 0.002336 | 0.092454 |
| RGS5 | -1.76559 | 8.130152 | 0.00168 | 0.074279 |
| ROR2 | -1.76608 | 2.884467 | 0.026771 | 0.319846 |
| CXCL9 | -1.76828 | 3.840066 | 0.047237 | 0.414589 |
| CASC1 | -1.77 | 1.763379 | 0.047707 | 0.416439 |
| PRELID2 | -1.77082 | 2.976187 | 0.009828 | 0.192944 |
| SEMA3A | -1.77347 | 2.561698 | 0.01779 | 0.261695 |
| ABCG2 | -1.77587 | 4.112913 | 0.006474 | 0.156814 |
| WTIP | -1.77617 | 2.0309 | 0.026132 | 0.315499 |
| SYT11 | -1.77689 | 4.827564 | 0.002523 | 0.096075 |
| PCDH18 | -1.77699 | 5.503848 | 0.003825 | 0.12062 |
| DCN | -1.77733 | 8.552492 | 0.004975 | 0.137306 |
| MAMDC2-AS1 | -1.78168 | 1.408388 | 0.031251 | 0.34136 |
| DZIP1L | -1.78486 | 2.081502 | 0.018702 | 0.268576 |
| NLGN4Y | -1.78637 | 4.698092 | 0.011889 | 0.213108 |
| COL12A1 | -1.78644 | 8.050154 | 0.000282 | 0.026365 |
| OGN | -1.78739 | 5.724028 | 0.010781 | 0.201814 |
| PDGFRA | -1.78807 | 5.845472 | 0.001624 | 0.073334 |
| HEY2 | -1.79033 | 1.875243 | 0.033441 | 0.352354 |
| AC107072.2 | -1.79285 | 0.755924 | 0.048341 | 0.419059 |
| DSC2 | -1.79416 | 6.92778 | 0.010012 | 0.1944 |
| TTLL7 | -1.7943 | 5.356919 | 0.000711 | 0.045331 |
| SLC1A2 | -1.79546 | 3.124671 | 0.037258 | 0.370677 |
| TGFBR3 | -1.79804 | 5.199675 | 0.009063 | 0.186067 |
| ARHGAP28 | -1.80193 | 5.278169 | 0.020181 | 0.278163 |
| HADHAP1 | -1.80656 | 3.439752 | 0.028322 | 0.327745 |
| KLHL5 | -1.80937 | 5.305779 | 0.000456 | 0.034522 |
| TPBG | -1.80941 | 3.017278 | 0.01327 | 0.225242 |
| NEK10 | -1.81021 | 2.661334 | 0.031631 | 0.342825 |
| GFPT2 | -1.81297 | 2.622764 | 0.01309 | 0.223646 |
| RNF180 | -1.81299 | 4.385196 | 0.002193 | 0.088111 |
| AC119744.1 | -1.81324 | 3.357445 | 0.030208 | 0.336776 |
| NLRP1 | -1.8161 | 4.041862 | 0.004951 | 0.137306 |
| APCDD1 | -1.81695 | 4.738603 | 0.001534 | 0.071207 |
| PAPLN | -1.8171 | 3.042302 | 0.018241 | 0.26515 |
| SERPING1 | -1.8185 | 5.773741 | 0.001216 | 0.062432 |
| PROS1 | -1.82083 | 4.718868 | 0.000815 | 0.04882 |
| ANGPTL7 | -1.82124 | 1.837693 | 0.030509 | 0.338321 |
| PLA2G4F | -1.8223 | 3.631011 | 0.040056 | 0.384966 |
| AL117190.1 | -1.82261 | 1.394 | 0.034597 | 0.359025 |
| STARD9 | -1.826 | 4.589031 | 0.001671 | 0.074024 |
| CLDN18 | -1.82815 | 0.636034 | 0.048064 | 0.417896 |
| MYH11 | -1.82972 | 9.791318 | 0.026682 | 0.319334 |
| AL121790.2 | -1.83126 | 3.924873 | 0.046859 | 0.412812 |
| IQUB | -1.83155 | 2.494667 | 0.028044 | 0.32679 |
| OR7E47P | -1.83209 | 3.070081 | 0.029973 | 0.335707 |
| ITM2A | -1.83407 | 2.999396 | 0.00811 | 0.174204 |
| HSPA2 | -1.83471 | 1.569184 | 0.028882 | 0.330578 |
| KLF12 | -1.8362 | 5.117444 | 0.000879 | 0.050858 |
| AGT | -1.83632 | 1.794614 | 0.02952 | 0.333217 |
| STXBP5-AS1 | -1.83635 | 2.779887 | 0.037179 | 0.37038 |
| SORT1 | -1.837 | 6.562649 | 0.000735 | 0.046121 |
| AL121790.1 | -1.83798 | 5.132149 | 0.032883 | 0.349476 |
| FRMD3 | -1.83829 | 3.586539 | 0.004768 | 0.134695 |
| SELP | -1.83829 | 2.975787 | 0.008371 | 0.177817 |
| MAMDC2 | -1.84372 | 3.029467 | 0.007173 | 0.163776 |
| ATP1B2 | -1.84455 | 1.520274 | 0.024967 | 0.308824 |
| DKK2 | -1.8459 | 3.367149 | 0.019457 | 0.273563 |
| NACAD | -1.84645 | 0.630339 | 0.04552 | 0.408025 |
| EBF1 | -1.84707 | 3.63549 | 0.005321 | 0.142483 |
| MLKL | -1.84784 | 2.744938 | 0.016193 | 0.249019 |
| CLDN8 | -1.85239 | 5.317847 | 0.035555 | 0.36326 |
| SLC25A21 | -1.85347 | 0.568435 | 0.048571 | 0.419675 |
| IGFBP7 | -1.85485 | 7.107667 | 0.001111 | 0.059535 |
| PARM1 | -1.8562 | 6.456708 | 0.00428 | 0.127391 |
| TCF21 | -1.85726 | 3.06985 | 0.023023 | 0.299099 |
| PRORSD1P | -1.86046 | 1.769639 | 0.021211 | 0.286161 |
| GPC3 | -1.86062 | 2.385798 | 0.0113 | 0.207619 |
| AC015712.2 | -1.86321 | 4.044815 | 0.01242 | 0.217394 |
| MITF | -1.86547 | 4.809296 | 0.001446 | 0.06888 |
| CACNA1C-AS4 | -1.86579 | 0.700499 | 0.04805 | 0.417896 |
| RAB3C | -1.86719 | 1.457481 | 0.036648 | 0.368495 |
| ADAM23 | -1.86924 | 1.94753 | 0.014666 | 0.23621 |
| IGFBP5 | -1.86984 | 8.491295 | 0.000216 | 0.022717 |
| 6-Sep | -1.87273 | 4.368563 | 0.000833 | 0.049409 |
| KCNQ3 | -1.87488 | 2.095976 | 0.026989 | 0.321447 |
| SFRP4 | -1.87795 | 6.664936 | 0.01791 | 0.262787 |
| PINLYP | -1.8786 | 0.466676 | 0.043699 | 0.399631 |
| FJX1 | -1.87978 | 1.405852 | 0.038605 | 0.376939 |
| PALMD | -1.8824 | 4.423882 | 0.00718 | 0.163776 |
| NME9 | -1.88241 | 1.571992 | 0.028044 | 0.32679 |
| ZNF804A | -1.88407 | 1.209259 | 0.036055 | 0.365594 |
| PCDHGA5 | -1.88455 | 3.668727 | 0.037811 | 0.372994 |
| AEBP1 | -1.88764 | 7.387606 | 0.001188 | 0.061653 |
| PPP1R12B | -1.88823 | 8.357858 | 0.000542 | 0.03802 |
| CCDC181 | -1.88863 | 0.846564 | 0.032418 | 0.347213 |
| C5orf56 | -1.88866 | 3.622009 | 0.003838 | 0.12062 |
| OLFML1 | -1.88876 | 3.198825 | 0.007085 | 0.162963 |
| RAD17P2 | -1.88929 | 0.712238 | 0.040725 | 0.387608 |
| LAYN | -1.88955 | 3.408509 | 0.011256 | 0.207313 |
| IQGAP2 | -1.89141 | 7.688815 | 0.001237 | 0.063154 |
| UHRF2P1 | -1.892 | 2.426938 | 0.037855 | 0.373213 |
| GTF2IP7 | -1.89297 | 2.728892 | 0.017707 | 0.261262 |
| LRRC6 | -1.89419 | 1.611286 | 0.017831 | 0.262193 |
| NEGR1 | -1.89429 | 4.48486 | 0.009211 | 0.187717 |
| ATL1 | -1.89723 | 4.085379 | 0.011019 | 0.20481 |
| TRIB2 | -1.89788 | 5.465758 | 0.001082 | 0.058432 |
| OVGP1 | -1.89919 | 1.929786 | 0.020638 | 0.282089 |
| ACTA2-AS1 | -1.89972 | 2.55676 | 0.04473 | 0.40411 |
| C3orf80 | -1.90219 | 0.780919 | 0.036503 | 0.367607 |
| DACT3 | -1.90236 | 2.984678 | 0.020208 | 0.278396 |
| ZNF662 | -1.90373 | 3.075638 | 0.024481 | 0.306233 |
| DRP2 | -1.90711 | 2.217971 | 0.014483 | 0.234765 |
| SYP | -1.90963 | 1.299637 | 0.028666 | 0.329644 |
| RBMS1 | -1.91115 | 5.605258 | 0.000313 | 0.027884 |
| LRP2BP | -1.91177 | 4.131907 | 0.002551 | 0.096705 |
| FMOD | -1.91193 | 4.955361 | 0.000915 | 0.052282 |
| C3orf70 | -1.91203 | 3.623181 | 0.012178 | 0.215275 |
| AC015712.1 | -1.9134 | 0.738904 | 0.037807 | 0.372994 |
| GBP2 | -1.91411 | 5.452541 | 0.003245 | 0.110682 |
| PCDHGA6 | -1.91686 | 3.286117 | 0.038044 | 0.374252 |
| UST | -1.91718 | 3.111227 | 0.005274 | 0.141853 |
| SERPINA1 | -1.91765 | 3.763198 | 0.002875 | 0.103951 |
| SNRPGP4 | -1.91983 | 1.693817 | 0.021979 | 0.291829 |
| CCL21 | -1.92106 | 0.898879 | 0.029131 | 0.331551 |
| CCR2 | -1.92107 | 2.075932 | 0.025024 | 0.309191 |
| CHN2 | -1.92211 | 4.155003 | 0.004026 | 0.123444 |
| AQP4-AS1 | -1.92275 | 1.628029 | 0.04915 | 0.421959 |
| EMCN | -1.92602 | 4.469507 | 0.001687 | 0.074318 |
| JAM2 | -1.92748 | 4.519457 | 0.001641 | 0.073584 |
| TMEM121B | -1.92889 | 2.731896 | 0.023144 | 0.299763 |
| CES1 | -1.93234 | 3.162331 | 0.036537 | 0.367819 |
| CSDC2 | -1.9324 | 1.734101 | 0.034355 | 0.357594 |
| ABCA10 | -1.93317 | 3.71846 | 0.003028 | 0.107337 |
| SLCO3A1 | -1.93318 | 2.954042 | 0.007181 | 0.163776 |
| SATB2 | -1.93512 | 3.831507 | 0.013411 | 0.225811 |
| MFAP2 | -1.93808 | 1.741085 | 0.031599 | 0.342615 |
| ACSL6 | -1.93852 | 1.142717 | 0.046104 | 0.409791 |
| SLC39A8 | -1.93882 | 4.658993 | 0.001611 | 0.07317 |
| DIO3OS | -1.94398 | 1.122736 | 0.04614 | 0.40992 |
| MALT1 | -1.94562 | 8.275578 | 0.008265 | 0.176559 |
| INMT | -1.94602 | 3.946658 | 0.013113 | 0.223646 |
| SSC5D | -1.94645 | 3.396601 | 0.008397 | 0.178052 |
| FAT3 | -1.94869 | 5.133687 | 0.000724 | 0.045732 |
| GSTM4 | -1.95139 | 2.271285 | 0.030098 | 0.336223 |
| ACOT11 | -1.95197 | 2.739636 | 0.010072 | 0.194673 |
| PAPPA | -1.95232 | 4.601953 | 0.003385 | 0.11282 |
| ZNF578 | -1.95514 | 1.22462 | 0.048605 | 0.419691 |
| LRCH2 | -1.95606 | 3.695341 | 0.00975 | 0.192398 |
| KCNJ8 | -1.95684 | 2.683084 | 0.006012 | 0.152406 |
| MMRN1 | -1.95759 | 2.808101 | 0.026228 | 0.31572 |
| KCNN3 | -1.95818 | 2.736687 | 0.005043 | 0.1387 |
| ADAM33 | -1.95986 | 2.887056 | 0.028067 | 0.326845 |
| EBF2 | -1.9599 | 2.436638 | 0.021967 | 0.291783 |
| RGS7BP | -1.96582 | 2.282399 | 0.015758 | 0.245234 |
| CYYR1 | -1.96713 | 3.596115 | 0.002643 | 0.099327 |
| ESM1 | -1.96775 | 3.688608 | 0.019539 | 0.274051 |
| MAOB | -1.96833 | 4.862358 | 0.000481 | 0.036029 |
| NEBL | -1.96912 | 5.189994 | 0.011778 | 0.212405 |
| MISP3 | -1.96937 | 1.895678 | 0.026174 | 0.315552 |
| ILDR2 | -1.96977 | 2.66339 | 0.015523 | 0.243401 |
| CMYA5 | -1.97358 | 5.116198 | 0.001427 | 0.068436 |
| ARMCX1 | -1.97527 | 3.041967 | 0.007199 | 0.164097 |
| TNFAIP8L3 | -1.97672 | 1.725138 | 0.018305 | 0.265862 |
| ANTXR1 | -1.97918 | 8.254962 | 0.000373 | 0.03097 |
| AC023908.3 | -1.98363 | 1.104624 | 0.034658 | 0.359025 |
| BOC | -1.98383 | 4.430496 | 0.004015 | 0.123444 |
| SULF1 | -1.98546 | 7.981684 | 0.001316 | 0.065312 |
| NFASC | -1.98716 | 5.984484 | 0.002158 | 0.087422 |
| SNTB1 | -1.98771 | 3.123176 | 0.003837 | 0.12062 |
| CRISPLD2 | -1.98793 | 6.242659 | 0.000279 | 0.026292 |
| MRGPRF | -1.98874 | 1.077769 | 0.044809 | 0.404186 |
| AC009962.1 | -1.98925 | 0.322673 | 0.048373 | 0.419059 |
| AC007161.1 | -1.98928 | 0.301566 | 0.040223 | 0.385605 |
| ISM1 | -1.98957 | 2.051888 | 0.019076 | 0.270444 |
| MICA | -1.99207 | 2.286491 | 0.01499 | 0.239047 |
| HCG11 | -1.99416 | 3.043933 | 0.005681 | 0.147383 |
| LIFR | -1.9943 | 8.552744 | 0.003768 | 0.1194 |
| CD55 | -1.99484 | 4.570411 | 0.002986 | 0.10657 |
| CELF2-AS1 | -1.99643 | 0.773115 | 0.030514 | 0.338321 |
| PSTPIP2 | -1.99984 | 3.288207 | 0.015928 | 0.246503 |
| SH3TC2 | -2.00007 | 1.661783 | 0.043696 | 0.399631 |
| TTC22 | -2.00332 | 1.006682 | 0.038391 | 0.376267 |
| NTN4 | -2.00347 | 5.36725 | 0.00036 | 0.030262 |
| CPQ | -2.00456 | 4.6568 | 0.000287 | 0.026382 |
| SLAMF6 | -2.00627 | 1.110017 | 0.040205 | 0.385543 |
| FLRT3 | -2.00725 | 4.144556 | 0.00812 | 0.174204 |
| PDZRN4 | -2.00727 | 2.496422 | 0.03483 | 0.359131 |
| GPR176 | -2.00796 | 4.287191 | 0.001833 | 0.078227 |
| NECAB1 | -2.00996 | 1.60607 | 0.038955 | 0.378517 |
| TBX2 | -2.01094 | 3.924209 | 0.001276 | 0.064282 |
| NDST3 | -2.01242 | 1.192049 | 0.02674 | 0.319695 |
| RGS22 | -2.0142 | 3.063688 | 0.014918 | 0.238222 |
| FERMT1 | -2.01464 | 3.214192 | 0.023127 | 0.299763 |
| IGKV1-5 | -2.0156 | 0.523327 | 0.04324 | 0.398402 |
| RAP2C-AS1 | -2.016 | 1.90887 | 0.026792 | 0.31998 |
| GSTP1 | -2.01639 | 4.990754 | 0.002715 | 0.100592 |
| REEP1 | -2.01672 | 2.658353 | 0.03733 | 0.370784 |
| NLGN3 | -2.0176 | 1.809605 | 0.022535 | 0.296001 |
| FAM204BP | -2.0206 | 0.668689 | 0.036292 | 0.366642 |
| DNAJC15 | -2.02106 | 3.369974 | 0.004393 | 0.129847 |
| ADAM20 | -2.02211 | 1.016501 | 0.044222 | 0.402111 |
| FSIP1 | -2.02441 | 2.052313 | 0.047035 | 0.413305 |
| SLC8A1 | -2.02529 | 6.480188 | 0.000687 | 0.044328 |
| NAALAD2 | -2.02771 | 2.261593 | 0.024049 | 0.303586 |
| RPS15AP30 | -2.02795 | 0.217162 | 0.040029 | 0.384928 |
| FAP | -2.02941 | 4.573898 | 0.002304 | 0.091512 |
| GPRC5C | -2.03236 | 3.808692 | 0.00732 | 0.165406 |
| AL158206.1 | -2.03249 | 3.765534 | 0.012424 | 0.217394 |
| TTC9 | -2.0326 | 2.99014 | 0.006928 | 0.161028 |
| CASC15 | -2.03268 | 3.434255 | 0.003347 | 0.112578 |
| ABCC11 | -2.03445 | 1.704176 | 0.035726 | 0.363815 |
| ROR1 | -2.0346 | 3.458285 | 0.003742 | 0.119322 |
| CCDC144NL-AS1 | -2.03626 | 2.977877 | 0.007667 | 0.169913 |
| PRKACB | -2.03963 | 6.874243 | 0.00033 | 0.028576 |
| AC138207.8 | -2.03997 | 1.625953 | 0.013585 | 0.227825 |
| SHISA6 | -2.04072 | 2.868712 | 0.034609 | 0.359025 |
| FCMR | -2.0409 | 1.115501 | 0.043071 | 0.398085 |
| RIMS2 | -2.04311 | 4.482195 | 0.005556 | 0.145449 |
| AL157373.2 | -2.0472 | 1.385085 | 0.024633 | 0.306453 |
| NGFR | -2.05043 | 2.783844 | 0.023718 | 0.302071 |
| NALCN | -2.05302 | 2.118628 | 0.016378 | 0.250655 |
| WFDC1 | -2.05722 | 3.861561 | 0.016782 | 0.254624 |
| CLEC1A | -2.05746 | 0.832171 | 0.023642 | 0.301667 |
| EVC2 | -2.05818 | 2.04167 | 0.009137 | 0.187259 |
| CYS1 | -2.05893 | 0.945787 | 0.034516 | 0.35887 |
| SCRN1 | -2.06173 | 5.375608 | 0.000132 | 0.016077 |
| AC097534.2 | -2.06396 | 0.166253 | 0.032437 | 0.347236 |
| TMEM220-AS1 | -2.06412 | 0.400721 | 0.030301 | 0.337378 |
| NSUN7 | -2.06417 | 3.820197 | 0.022304 | 0.294438 |
| SLC44A3-AS1 | -2.06494 | 1.26809 | 0.019492 | 0.273834 |
| PRKCQ | -2.06573 | 1.005955 | 0.019768 | 0.275572 |
| BHMT2 | -2.06903 | 2.205355 | 0.014052 | 0.231059 |
| AC105383.1 | -2.06935 | 0.8513 | 0.034811 | 0.359131 |
| PROM1 | -2.06961 | 2.252484 | 0.037438 | 0.370913 |
| CNTD1 | -2.07226 | 1.704861 | 0.039439 | 0.382037 |
| HMCN1 | -2.07588 | 6.794168 | 0.002036 | 0.083736 |
| PLEKHG1 | -2.07647 | 4.191517 | 0.00052 | 0.037459 |
| CBFA2T3 | -2.07731 | 1.933419 | 0.013663 | 0.228487 |
| ABLIM3 | -2.07779 | 4.280012 | 0.003339 | 0.112578 |
| AC018462.1 | -2.08303 | 1.728942 | 0.015774 | 0.245278 |
| FOXF1 | -2.08435 | 2.339611 | 0.043953 | 0.400672 |
| HLF | -2.08633 | 3.606952 | 0.009006 | 0.185342 |
| ARMH4 | -2.08723 | 2.710473 | 0.003252 | 0.110781 |
| ANK2 | -2.0941 | 5.013073 | 0.000366 | 0.030411 |
| LCP1 | -2.09416 | 8.088397 | 0.000488 | 0.036225 |
| COL10A1 | -2.09416 | 5.372212 | 5.15E-05 | 0.008889 |
| RNF133 | -2.09769 | 0.792629 | 0.018163 | 0.264492 |
| FOXC1 | -2.09853 | 2.789562 | 0.006755 | 0.159859 |
| AC108058.1 | -2.09907 | 1.546673 | 0.012146 | 0.215026 |
| PRICKLE2 | -2.10069 | 4.055981 | 0.000647 | 0.042509 |
| RERG | -2.10248 | 4.49273 | 0.00403 | 0.123444 |
| GLT8D2 | -2.10301 | 3.83363 | 0.001171 | 0.061232 |
| ATOH8 | -2.10326 | 2.263226 | 0.016144 | 0.248559 |
| PRIMA1 | -2.10333 | 1.347764 | 0.02155 | 0.288816 |
| MATN2 | -2.10694 | 6.518606 | 0.000673 | 0.043783 |
| SCN7A | -2.10782 | 5.125737 | 0.000974 | 0.054087 |
| MACROD2-AS1 | -2.10813 | 0.470737 | 0.033087 | 0.350714 |
| PARP6 | -2.11005 | 4.206259 | 0.000351 | 0.029669 |
| ASB2 | -2.11102 | 2.165098 | 0.031324 | 0.341465 |
| TMEM100 | -2.11293 | 1.529832 | 0.042118 | 0.393128 |
| IL7R | -2.11344 | 3.877702 | 0.006983 | 0.161729 |
| TENM3 | -2.11411 | 4.317387 | 0.007343 | 0.165605 |
| PABPC5 | -2.11434 | 1.66875 | 0.012121 | 0.215026 |
| SEMA6D | -2.11474 | 3.674609 | 0.001906 | 0.079925 |
| CDKN2B-AS1 | -2.11487 | 0.773197 | 0.03164 | 0.342825 |
| LEPR | -2.11885 | 5.543385 | 0.000113 | 0.015067 |
| AC104365.1 | -2.12084 | 2.227418 | 0.035589 | 0.363262 |
| JPH4 | -2.12294 | 1.283686 | 0.017203 | 0.257834 |
| KAT2B | -2.12558 | 5.952046 | 0.000109 | 0.014841 |
| DMKN | -2.12607 | 1.569351 | 0.013648 | 0.228351 |
| NMNAT2 | -2.12818 | 2.357305 | 0.01669 | 0.253774 |
| LINC00844 | -2.12822 | 1.82861 | 0.034691 | 0.359025 |
| AL592490.1 | -2.12915 | 2.745432 | 0.042601 | 0.395919 |
| MXRA5 | -2.13084 | 6.733651 | 0.000109 | 0.014841 |
| ANKRD37 | -2.13119 | 3.529481 | 0.011434 | 0.208908 |
| MTND5P2 | -2.13308 | 0.041621 | 0.044588 | 0.403461 |
| SLC16A7 | -2.13582 | 2.877077 | 0.003418 | 0.113539 |
| ACKR1 | -2.13674 | 2.036647 | 0.012538 | 0.2184 |
| TEX41 | -2.14138 | 0.46287 | 0.031523 | 0.342118 |
| RNF144A-AS1 | -2.14175 | 0.716565 | 0.02428 | 0.305047 |
| ENDOD1 | -2.14632 | 7.745843 | 0.006718 | 0.159463 |
| GDF7 | -2.14936 | 3.265031 | 0.017016 | 0.25639 |
| PPARGC1A | -2.15003 | 2.576869 | 0.024226 | 0.304813 |
| TTLL7-IT1 | -2.15717 | 0.688505 | 0.029439 | 0.33274 |
| STOX2 | -2.15927 | 3.272912 | 0.003954 | 0.122456 |
| CDHR3 | -2.16159 | 3.078004 | 0.002882 | 0.10406 |
| FGF2 | -2.16503 | 3.686225 | 0.003851 | 0.12076 |
| PREX2 | -2.16541 | 5.518036 | 0.000621 | 0.041039 |
| WDR86 | -2.16625 | 0.298969 | 0.026198 | 0.315552 |
| B3GLCT | -2.16677 | 3.228718 | 0.00151 | 0.070761 |
| NRG4 | -2.16703 | 3.458375 | 0.007772 | 0.171121 |
| LDHAL6B | -2.16911 | 0.772208 | 0.021114 | 0.285351 |
| HHAT | -2.16935 | 2.657023 | 0.005545 | 0.145325 |
| DKK3 | -2.16943 | 6.0017 | 0.000397 | 0.032238 |
| PLCB1 | -2.16951 | 5.797191 | 0.00347 | 0.114733 |
| IGHA1 | -2.17129 | 5.636153 | 0.014129 | 0.231768 |
| FGF14 | -2.17599 | 3.044269 | 0.00364 | 0.117503 |
| ZNF215 | -2.17895 | 1.529551 | 0.02793 | 0.326699 |
| CMAHP | -2.18082 | 4.112312 | 0.000599 | 0.040267 |
| RUNX2 | -2.18112 | 3.781783 | 0.000748 | 0.04642 |
| PDE7B | -2.18376 | 3.385111 | 0.002223 | 0.088895 |
| PLCE1-AS1 | -2.18433 | -0.29022 | 0.035979 | 0.365415 |
| TBX2-AS1 | -2.18949 | 0.410562 | 0.027025 | 0.321536 |
| ACAN | -2.19289 | 1.086734 | 0.023043 | 0.299138 |
| CYP1B1 | -2.19442 | 6.209203 | 0.000181 | 0.02006 |
| NEXN | -2.19785 | 5.167797 | 0.000495 | 0.036487 |
| OSMR | -2.1991 | 5.424242 | 0.000234 | 0.023626 |
| AOX1 | -2.19991 | 4.286537 | 0.014488 | 0.234765 |
| CACNA2D1 | -2.19992 | 5.351796 | 0.000401 | 0.032293 |
| KCNMA1-AS1 | -2.19994 | 1.382017 | 0.033718 | 0.353753 |
| GAPDHP42 | -2.20134 | 0.410453 | 0.021226 | 0.286235 |
| ZNF423 | -2.20224 | 2.69501 | 0.00245 | 0.094982 |
| ZNF185 | -2.2053 | 4.613366 | 0.000382 | 0.031499 |
| MYRFL | -2.20579 | 1.771747 | 0.026153 | 0.315552 |
| AC016582.3 | -2.20626 | -0.15764 | 0.031042 | 0.340788 |
| MIR646HG | -2.20838 | 0.161605 | 0.045534 | 0.408025 |
| ADCY2 | -2.20907 | 4.227997 | 0.015864 | 0.2459 |
| AC012409.1 | -2.20965 | 0.261849 | 0.036031 | 0.365594 |
| DTHD1 | -2.21057 | 0.590791 | 0.020296 | 0.279393 |
| RAB27A | -2.21142 | 7.538911 | 0.010772 | 0.201746 |
| PLEKHH2 | -2.21381 | 4.305148 | 0.000299 | 0.027111 |
| FGF1 | -2.21402 | 2.036294 | 0.00804 | 0.173474 |
| TLL1 | -2.21566 | 2.048551 | 0.011551 | 0.210481 |
| ADH4 | -2.2159 | 1.636699 | 0.040079 | 0.384977 |
| CYP1B1-AS1 | -2.218 | 1.133856 | 0.048706 | 0.419721 |
| PDGFRL | -2.21928 | 1.397824 | 0.017761 | 0.261605 |
| SSPN | -2.22849 | 3.762067 | 0.00083 | 0.049409 |
| PHKA2-AS1 | -2.22852 | 0.152384 | 0.034275 | 0.357213 |
| ESRRG | -2.22983 | 3.477359 | 0.007984 | 0.173411 |
| AC113383.1 | -2.23315 | -0.38002 | 0.042081 | 0.393128 |
| AL357060.1 | -2.23626 | 0.824187 | 0.012601 | 0.218706 |
| FAM135A | -2.24259 | 7.370502 | 2.68E-05 | 0.005858 |
| WNK3 | -2.24288 | 5.08758 | 4.27E-05 | 0.008008 |
| FA2H | -2.24776 | -0.11447 | 0.042657 | 0.396218 |
| MOXD1 | -2.25431 | 5.713725 | 0.00264 | 0.099327 |
| AL161457.2 | -2.25432 | 1.674957 | 0.022281 | 0.294363 |
| CCL28 | -2.25438 | 1.348828 | 0.018984 | 0.270012 |
| NRCAM | -2.25455 | 4.634047 | 0.005536 | 0.145325 |
| CDC14B | -2.25519 | 5.335208 | 9.03E-05 | 0.013317 |
| MUC12 | -2.25735 | 1.55501 | 0.018488 | 0.267127 |
| SHB | -2.2577 | 1.425858 | 0.009453 | 0.189906 |
| SYT1 | -2.25845 | 5.886314 | 0.006764 | 0.159859 |
| AIMP1P1 | -2.25967 | 0.941729 | 0.021112 | 0.285351 |
| LRFN5 | -2.26218 | 2.479569 | 0.033571 | 0.352642 |
| CABCOCO1 | -2.26725 | 2.017382 | 0.023872 | 0.303138 |
| EDN1 | -2.2683 | 2.817553 | 0.015626 | 0.244006 |
| EPHA6 | -2.27059 | 5.349702 | 0.031279 | 0.34136 |
| HAVCR1P1 | -2.27096 | 1.15205 | 0.028842 | 0.330485 |
| ABCA6 | -2.27451 | 4.389207 | 0.00011 | 0.014937 |
| C8orf34-AS1 | -2.27856 | 2.526661 | 0.039559 | 0.382541 |
| TMEM178A | -2.27861 | 1.576456 | 0.007213 | 0.164199 |
| PROX1 | -2.27872 | 2.616204 | 0.005138 | 0.139833 |
| ITGA6-AS1 | -2.27904 | 0.018598 | 0.029297 | 0.33243 |
| LINC00996 | -2.27926 | 0.442849 | 0.01707 | 0.256745 |
| WHAMMP2 | -2.27996 | 1.460428 | 0.006994 | 0.16189 |
| KCNMB1 | -2.2814 | 3.332747 | 0.009588 | 0.191135 |
| EFNA5 | -2.28631 | 3.382012 | 0.005627 | 0.146628 |
| AC092821.1 | -2.28837 | 2.03111 | 0.025755 | 0.313156 |
| CLDN11 | -2.28857 | 2.71508 | 0.002367 | 0.092805 |
| OACYLP | -2.29189 | 2.818594 | 0.030975 | 0.340778 |
| ZEB1 | -2.29308 | 6.532064 | 1.36E-05 | 0.003502 |
| CDKL5 | -2.29517 | 4.498455 | 0.000411 | 0.032697 |
| PCDH10 | -2.29581 | 4.607154 | 0.005662 | 0.147104 |
| GUSBP5 | -2.29718 | 0.594671 | 0.025464 | 0.312028 |
| ZNF680P1 | -2.29864 | 0.970538 | 0.035602 | 0.363262 |
| CX3CR1 | -2.30124 | 3.271229 | 0.001351 | 0.066402 |
| LMO3 | -2.30145 | 4.189276 | 0.00033 | 0.028576 |
| RUNDC3B | -2.30232 | 2.443924 | 0.002988 | 0.10657 |
| FADS2 | -2.30577 | 4.71821 | 0.000151 | 0.01779 |
| AP000897.2 | -2.30638 | 0.90266 | 0.015474 | 0.242995 |
| MGP | -2.30752 | 7.818026 | 0.00051 | 0.037158 |
| Z99289.1 | -2.30927 | 0.783209 | 0.015691 | 0.244769 |
| NAV2 | -2.30991 | 3.986004 | 0.000361 | 0.030262 |
| SUSD5 | -2.31076 | 1.750048 | 0.010301 | 0.197997 |
| ACSS3 | -2.31124 | 3.365364 | 0.001373 | 0.066958 |
| C8orf34 | -2.312 | 3.311417 | 0.008799 | 0.183581 |
| SPOCK3 | -2.31285 | 4.927103 | 0.044282 | 0.402388 |
| XK | -2.31389 | 0.827417 | 0.016045 | 0.247694 |
| PTGER2 | -2.31833 | 1.987261 | 0.03087 | 0.340084 |
| MBL1P | -2.31836 | 1.711806 | 0.009781 | 0.192538 |
| PCDHGA3 | -2.32082 | 1.706124 | 0.04469 | 0.403967 |
| AL137003.2 | -2.32104 | 2.308529 | 0.005042 | 0.1387 |
| NBAT1 | -2.3239 | 0.014735 | 0.020897 | 0.284043 |
| PTPRQ | -2.32407 | -0.62069 | 0.039385 | 0.381788 |
| CPB1 | -2.32484 | 2.168962 | 0.036722 | 0.368727 |
| PDE10A | -2.32555 | 6.059539 | 0.011426 | 0.208908 |
| OMD | -2.32685 | 4.209285 | 0.000315 | 0.027936 |
| PYHIN1 | -2.32834 | 1.384913 | 0.007275 | 0.165056 |
| PDE8B | -2.32898 | 4.82404 | 0.000459 | 0.034635 |
| FKBP9P1 | -2.32981 | 0.224752 | 0.028644 | 0.3295 |
| TESPA1 | -2.33293 | 1.941228 | 0.008684 | 0.182216 |
| AC064836.3 | -2.33647 | -0.2574 | 0.030458 | 0.338057 |
| UBE2QL1 | -2.33868 | 1.688409 | 0.008501 | 0.179472 |
| AL357054.4 | -2.34087 | 0.171657 | 0.016479 | 0.251237 |
| TRPC6 | -2.34214 | 3.108611 | 0.006182 | 0.154435 |
| NKX3-2 | -2.34394 | -0.1277 | 0.040258 | 0.38563 |
| AMPH | -2.34749 | 1.553638 | 0.007603 | 0.169093 |
| SLC25A18 | -2.35072 | 2.340233 | 0.015764 | 0.245234 |
| DMD | -2.35301 | 6.604269 | 0.001528 | 0.071 |
| PLAC8 | -2.3545 | 0.720528 | 0.015905 | 0.246418 |
| GSTM5 | -2.35749 | 1.826292 | 0.042622 | 0.396004 |
| LINC00920 | -2.35843 | 0.541811 | 0.016459 | 0.251152 |
| FAM177B | -2.35904 | 0.361068 | 0.041246 | 0.38947 |
| HSPA12A | -2.36 | 3.513671 | 0.000334 | 0.028777 |
| ZNF454 | -2.3614 | 1.615173 | 0.003478 | 0.114884 |
| PRDM5 | -2.3617 | 3.771731 | 0.000255 | 0.024666 |
| FAM198B-AS1 | -2.36511 | 4.088509 | 0.001173 | 0.061232 |
| CCDC146 | -2.36649 | 4.034614 | 0.001349 | 0.066402 |
| SNORA35B | -2.36739 | 1.70818 | 0.008497 | 0.179472 |
| GLYATL1B | -2.37108 | 3.674224 | 0.011382 | 0.208617 |
| MTND4P12 | -2.37319 | 3.96971 | 0.002719 | 0.100592 |
| TSPAN2 | -2.37581 | 4.312301 | 0.000305 | 0.027462 |
| GREM2 | -2.37642 | 3.730629 | 0.009214 | 0.187717 |
| TRMT9B | -2.37912 | 4.014816 | 0.005766 | 0.148467 |
| FFAR4 | -2.37975 | 0.747852 | 0.009878 | 0.193435 |
| AC055733.2 | -2.38029 | 0.964311 | 0.013212 | 0.22488 |
| FYB2 | -2.38137 | 3.95312 | 0.004099 | 0.124368 |
| RUNX1T1 | -2.38172 | 4.064265 | 9.18E-05 | 0.01348 |
| TMEM47 | -2.38297 | 6.34785 | 2.83E-05 | 0.006016 |
| FBXO32 | -2.3836 | 7.212809 | 3.16E-06 | 0.001344 |
| SCN3B | -2.38501 | 1.563571 | 0.007964 | 0.173273 |
| LRRIQ1 | -2.38574 | 4.668854 | 0.007997 | 0.173432 |
| AC008937.2 | -2.38607 | -0.08582 | 0.025744 | 0.313156 |
| ABCA9 | -2.38967 | 3.256953 | 0.000932 | 0.052375 |
| ARHGEF28 | -2.3897 | 4.005765 | 0.000162 | 0.018395 |
| RORC | -2.39152 | 3.752039 | 0.010082 | 0.194673 |
| CSMD2 | -2.39195 | 2.020013 | 0.010192 | 0.19646 |
| AC022537.1 | -2.39296 | -0.62774 | 0.040569 | 0.387088 |
| SATB1 | -2.39324 | 5.538868 | 0.000106 | 0.014765 |
| CDH2 | -2.39405 | 3.279532 | 0.000593 | 0.039916 |
| SLPI | -2.3957 | 3.68192 | 0.000693 | 0.04449 |
| CDH3 | -2.4015 | 3.850863 | 0.000238 | 0.023923 |
| MPP2 | -2.40432 | 1.156732 | 0.025923 | 0.314318 |
| DIRAS3 | -2.40437 | 0.904127 | 0.005972 | 0.151622 |
| KCNAB1 | -2.40474 | 4.202188 | 0.000121 | 0.015424 |
| AC254633.1 | -2.4062 | -0.24821 | 0.033102 | 0.350714 |
| GAS1 | -2.40648 | 2.542484 | 0.001773 | 0.076335 |
| AC103702.1 | -2.40853 | -0.24911 | 0.028095 | 0.326915 |
| GRIA3 | -2.40937 | 1.424569 | 0.010038 | 0.194673 |
| LINC00865 | -2.41106 | 1.510012 | 0.008764 | 0.183326 |
| AL645933.2 | -2.41639 | 0.395819 | 0.02729 | 0.322898 |
| ROR1-AS1 | -2.42174 | -0.27742 | 0.048368 | 0.419059 |
| AC011595.1 | -2.42349 | -0.19691 | 0.036108 | 0.36565 |
| AC012613.2 | -2.42518 | 1.37254 | 0.026509 | 0.31838 |
| PAM | -2.42564 | 7.280717 | 8.49E-06 | 0.00265 |
| PDCD6IPP2 | -2.42589 | -0.93757 | 0.046007 | 0.409485 |
| DAPK2 | -2.4321 | 2.589761 | 0.005372 | 0.143255 |
| PHYHIP | -2.4325 | 0.573761 | 0.011303 | 0.207619 |
| PCDHGA2 | -2.43374 | 2.521544 | 0.016196 | 0.249019 |
| DLGAP2 | -2.43521 | 2.315433 | 0.017703 | 0.261262 |
| GCNT1 | -2.4355 | 5.998632 | 0.00218 | 0.088027 |
| FREM2 | -2.4361 | 4.846206 | 0.009981 | 0.194025 |
| AC007750.1 | -2.43753 | -0.22382 | 0.024314 | 0.305254 |
| SLIT2 | -2.43772 | 5.932181 | 3.92E-06 | 0.001549 |
| NTRK3 | -2.43781 | 0.851377 | 0.010062 | 0.194673 |
| SYT6 | -2.43784 | 1.13291 | 0.010422 | 0.198777 |
| CD3D | -2.44356 | 0.927889 | 0.015163 | 0.240123 |
| NMD3P1 | -2.44386 | -0.41594 | 0.031882 | 0.343914 |
| TIMP4 | -2.45042 | 0.296994 | 0.018851 | 0.26935 |
| SRPX | -2.45645 | 2.031006 | 0.00962 | 0.191333 |
| SMIM10 | -2.46083 | 1.068511 | 0.004562 | 0.131678 |
| ETV1 | -2.46121 | 4.623513 | 6.58E-05 | 0.010658 |
| NAT1 | -2.46138 | 2.481116 | 0.003857 | 0.120769 |
| MUC3A | -2.46461 | 2.458809 | 0.005764 | 0.148467 |
| PRR15 | -2.46547 | -0.06672 | 0.015266 | 0.241314 |
| SOX10 | -2.46801 | 1.608896 | 0.013918 | 0.230725 |
| GDA | -2.46934 | 1.571174 | 0.022607 | 0.296149 |
| SV2A | -2.47135 | 1.71918 | 0.005232 | 0.141387 |
| ITGB4 | -2.47217 | 3.77709 | 0.000187 | 0.020504 |
| C1QTNF2 | -2.47293 | 0.170502 | 0.018459 | 0.267127 |
| PGR | -2.478 | 4.8674 | 0.000293 | 0.026722 |
| COL21A1 | -2.4807 | 2.905426 | 0.001388 | 0.067417 |
| TGFB2 | -2.49057 | 5.063923 | 0.000104 | 0.014597 |
| PRELP | -2.49098 | 5.779466 | 4.64E-06 | 0.001734 |
| AL354890.1 | -2.49176 | -0.12602 | 0.043602 | 0.399485 |
| EYA1 | -2.49231 | 3.994071 | 0.003507 | 0.115282 |
| FAM198B | -2.49261 | 7.246875 | 4.94E-06 | 0.001807 |
| LY75 | -2.49364 | 1.130993 | 0.009284 | 0.18781 |
| DDIT4L | -2.49414 | 1.323012 | 0.013362 | 0.225549 |
| TFPI2 | -2.49456 | 0.887714 | 0.006225 | 0.154706 |
| AL157911.1 | -2.49799 | 0.72223 | 0.034522 | 0.35887 |
| MIR100HG | -2.49956 | 5.119095 | 4.61E-05 | 0.008295 |
| DENND2C | -2.50002 | 2.584672 | 0.001868 | 0.078612 |
| PLA2G4A | -2.50276 | 4.24339 | 0.000148 | 0.017641 |
| AL121839.1 | -2.50615 | 0.087753 | 0.046188 | 0.410102 |
| SV2C | -2.50705 | 3.434514 | 0.000166 | 0.018644 |
| FAM162B | -2.50869 | 1.73899 | 0.003762 | 0.1194 |
| HOXB2 | -2.50875 | 1.429169 | 0.005567 | 0.145628 |
| MAGI2-AS3 | -2.50918 | 4.600229 | 2.52E-05 | 0.005622 |
| TMEM200B | -2.50934 | 1.723194 | 0.006288 | 0.155284 |
| ADH1C | -2.50995 | 3.596448 | 0.011719 | 0.212324 |
| ADGRL3 | -2.51026 | 2.961813 | 0.000918 | 0.052282 |
| AC087632.1 | -2.51097 | -1.12116 | 0.044081 | 0.401411 |
| AC022034.1 | -2.51677 | 3.55018 | 0.004567 | 0.131719 |
| ADAMTSL3 | -2.51925 | 4.578217 | 0.000342 | 0.029233 |
| PIFO | -2.52329 | 2.741594 | 0.007944 | 0.17313 |
| AL137145.1 | -2.52716 | 1.438088 | 0.004673 | 0.133341 |
| C8orf88 | -2.53106 | 0.312962 | 0.019362 | 0.272771 |
| FIGN | -2.53723 | 3.768139 | 0.00012 | 0.015406 |
| TSLP | -2.53807 | 0.478971 | 0.04611 | 0.409791 |
| MAML2 | -2.5397 | 5.793791 | 1.44E-05 | 0.003634 |
| AQP4 | -2.54017 | 1.835266 | 0.01967 | 0.274795 |
| CARMN | -2.54384 | 6.642242 | 0.000693 | 0.04449 |
| Z98884.1 | -2.54834 | -0.2285 | 0.028216 | 0.327538 |
| AC026355.1 | -2.55005 | 0.505092 | 0.036193 | 0.366191 |
| GLI1 | -2.55325 | 1.756558 | 0.006466 | 0.156814 |
| WSCD2 | -2.56262 | 1.770426 | 0.021822 | 0.29109 |
| ZNF536 | -2.56382 | 0.995528 | 0.004071 | 0.123754 |
| ABCB5 | -2.56993 | 1.54972 | 0.005908 | 0.150552 |
| SLC8A1-AS1 | -2.57106 | 1.011418 | 0.00818 | 0.1754 |
| ARMCX4 | -2.57619 | 4.021205 | 0.000219 | 0.022847 |
| SNHG18 | -2.57792 | 1.194857 | 0.007854 | 0.171825 |
| ADH6 | -2.57813 | 2.289911 | 0.003243 | 0.110682 |
| ABCC9 | -2.58227 | 5.429311 | 9.90E-07 | 0.000642 |
| GATA3 | -2.58444 | 1.82371 | 0.001359 | 0.066574 |
| RERG-AS1 | -2.58611 | -0.30412 | 0.035508 | 0.362997 |
| MAP3K20-AS1 | -2.58917 | 1.873746 | 0.006646 | 0.158657 |
| CIART | -2.58944 | 0.911067 | 0.008833 | 0.183581 |
| CSGALNACT1 | -2.59055 | 6.049868 | 9.96E-05 | 0.014307 |
| PRKG2 | -2.59094 | 2.982345 | 0.003781 | 0.119684 |
| RBBP4P2 | -2.59759 | 0.688914 | 0.036766 | 0.368833 |
| SPINK13 | -2.59958 | -0.45825 | 0.025243 | 0.310506 |
| AC093849.1 | -2.60064 | 0.21307 | 0.046982 | 0.41299 |
| PID1 | -2.6013 | 4.432173 | 0.00516 | 0.139887 |
| RBMS3 | -2.60145 | 5.744093 | 1.44E-06 | 0.000786 |
| HOXB9 | -2.60549 | -0.08282 | 0.049627 | 0.423942 |
| AC024257.3 | -2.6063 | -0.52432 | 0.037142 | 0.370226 |
| GUCY1A2 | -2.61278 | 7.341083 | 0.000117 | 0.015258 |
| ARAP2 | -2.61577 | 5.450873 | 8.62E-07 | 0.000592 |
| AC011891.1 | -2.6161 | -0.57137 | 0.024615 | 0.306453 |
| ITGA8 | -2.61826 | 6.796461 | 0.002859 | 0.103696 |
| AL139147.1 | -2.61884 | -0.11007 | 0.049194 | 0.422124 |
| CXCL1 | -2.62167 | 0.997935 | 0.002745 | 0.100669 |
| RASGRP2 | -2.6223 | 1.790358 | 0.002011 | 0.083115 |
| LMCD1-AS1 | -2.62256 | 1.295723 | 0.006457 | 0.156814 |
| VANGL2 | -2.62484 | 1.987294 | 0.001814 | 0.077495 |
| AC005828.2 | -2.62551 | 0.062888 | 0.022051 | 0.292109 |
| CCDC36 | -2.63047 | 0.468535 | 0.007551 | 0.16821 |
| AC005999.1 | -2.63164 | 0.466357 | 0.032374 | 0.347061 |
| SYT16 | -2.63285 | -0.32058 | 0.041352 | 0.389788 |
| KCNMA1-AS3 | -2.63422 | 0.715649 | 0.006334 | 0.156065 |
| AC135352.1 | -2.63535 | 0.071781 | 0.009543 | 0.19102 |
| GUCY2C | -2.63664 | 1.9705 | 0.031649 | 0.342825 |
| FAM131B | -2.63743 | 0.021132 | 0.022623 | 0.296149 |
| ANO4 | -2.64871 | 2.883404 | 0.002259 | 0.090121 |
| ASTN1 | -2.65137 | 0.71535 | 0.011216 | 0.207023 |
| DPYD-AS1 | -2.65366 | -0.0481 | 0.035633 | 0.36341 |
| GLRB | -2.65462 | 2.335939 | 0.003109 | 0.108752 |
| CHRD | -2.65647 | 1.780539 | 0.002656 | 0.099612 |
| PDLIM4 | -2.65658 | 2.374805 | 0.002933 | 0.105145 |
| AP000941.1 | -2.65823 | 0.576768 | 0.009914 | 0.193796 |
| NR2F1-AS1 | -2.65954 | 3.164911 | 0.000582 | 0.039436 |
| TMEM132C | -2.6606 | 1.034829 | 0.011407 | 0.208745 |
| AC068481.1 | -2.66194 | -0.40516 | 0.022931 | 0.298689 |
| EPHA1-AS1 | -2.66206 | -0.54177 | 0.048034 | 0.417896 |
| KCNJ15 | -2.66362 | 1.468696 | 0.002192 | 0.088111 |
| RPL12P20 | -2.66912 | 2.52479 | 0.003158 | 0.109723 |
| GRM6 | -2.67082 | -0.22138 | 0.043352 | 0.398699 |
| LAMB3 | -2.67273 | 2.460543 | 0.005879 | 0.150357 |
| C3orf14 | -2.67529 | 3.777735 | 0.004792 | 0.135151 |
| NDST4 | -2.67955 | 1.996125 | 0.010341 | 0.198223 |
| C9orf147 | -2.68659 | 0.814562 | 0.006346 | 0.156193 |
| LINC01235 | -2.68737 | -0.45852 | 0.017647 | 0.26082 |
| AC144833.1 | -2.68743 | 2.956729 | 0.044848 | 0.404218 |
| BEST3 | -2.69021 | 1.588891 | 0.004484 | 0.13112 |
| TMEM92 | -2.69232 | -0.27716 | 0.029624 | 0.33384 |
| AL445523.1 | -2.69302 | 0.359007 | 0.028778 | 0.330335 |
| SHC4 | -2.6931 | 1.631953 | 0.003364 | 0.112648 |
| AC110491.1 | -2.69594 | 1.768681 | 0.035432 | 0.362429 |
| MYOT | -2.69613 | 0.900781 | 0.005168 | 0.139887 |
| MIR3189 | -2.69728 | 1.524331 | 0.014626 | 0.235857 |
| MOGAT2 | -2.69874 | 1.362711 | 0.040506 | 0.386594 |
| IGFBP6 | -2.7008 | 3.158342 | 0.005325 | 0.142483 |
| PCGEM1 | -2.70126 | 3.984788 | 0.032309 | 0.346702 |
| L1TD1 | -2.70692 | 0.319939 | 0.026932 | 0.321096 |
| GCSAM | -2.70926 | 2.022489 | 0.00268 | 0.099942 |
| CNGB3 | -2.70979 | 1.471125 | 0.007235 | 0.164363 |
| AC110760.2 | -2.71197 | 1.510773 | 0.008645 | 0.181754 |
| SNCA | -2.71217 | 3.262216 | 0.000244 | 0.024154 |
| FHL5 | -2.71223 | 2.599215 | 0.000951 | 0.053197 |
| SNCAIP | -2.71262 | 2.687638 | 0.000226 | 0.023223 |
| CYP3A4 | -2.71448 | 0.481587 | 0.036247 | 0.366295 |
| L1CAM | -2.71737 | 1.883296 | 0.017435 | 0.259234 |
| RXFP1 | -2.71782 | 0.224491 | 0.00761 | 0.169093 |
| SYNDIG1 | -2.71785 | 0.508802 | 0.021827 | 0.29109 |
| KCNT2 | -2.71794 | 2.569702 | 0.000398 | 0.032238 |
| LRRN4CL | -2.71981 | 2.138141 | 0.010105 | 0.194998 |
| MATN3 | -2.72084 | 2.259545 | 0.00266 | 0.099612 |
| PTHLH | -2.72901 | -0.19862 | 0.046 | 0.409485 |
| LYST-AS1 | -2.73201 | -1.26018 | 0.049025 | 0.421619 |
| DNAH8 | -2.73306 | 5.650585 | 0.005801 | 0.148933 |
| CD1C | -2.73678 | 0.313859 | 0.045444 | 0.407666 |
| ADAMTS9-AS2 | -2.74038 | 3.465198 | 0.000964 | 0.05374 |
| C3orf52 | -2.74439 | 3.872319 | 0.000532 | 0.037984 |
| CLEC5A | -2.74609 | 0.786073 | 0.003802 | 0.120239 |
| SGCD | -2.74672 | 6.363459 | 0.00054 | 0.03802 |
| FAM153B | -2.75171 | 1.806192 | 0.021842 | 0.291135 |
| HOXD11 | -2.7539 | 0.219657 | 0.026724 | 0.319616 |
| MS4A4E | -2.75466 | 1.621944 | 0.001218 | 0.062432 |
| AC013726.1 | -2.75576 | 0.09155 | 0.032565 | 0.348198 |
| ADAMTSL1 | -2.7565 | 3.819981 | 0.000551 | 0.038156 |
| BHLHE40-AS1 | -2.7576 | -0.35076 | 0.03784 | 0.373175 |
| AC023421.2 | -2.76248 | 0.187838 | 0.028438 | 0.328042 |
| MIR578 | -2.7643 | 0.004065 | 0.018838 | 0.26935 |
| DIO2 | -2.76676 | 6.695616 | 0.000228 | 0.023223 |
| CRYZL2P | -2.76948 | 3.536971 | 0.000446 | 0.034365 |
| FLT3 | -2.76984 | 0.004866 | 0.030482 | 0.338189 |
| COL24A1 | -2.77063 | 1.350149 | 0.001688 | 0.074318 |
| PRRX1 | -2.77313 | 6.259866 | 1.78E-07 | 0.000175 |
| AC093225.1 | -2.77511 | 0.323984 | 0.0343 | 0.3573 |
| AL031123.2 | -2.77869 | 0.69376 | 0.008036 | 0.173474 |
| AC092652.1 | -2.78203 | -0.91223 | 0.025877 | 0.314077 |
| AL031770.1 | -2.78419 | 0.148079 | 0.018165 | 0.264492 |
| DNM3OS | -2.78547 | 1.832573 | 0.000561 | 0.038437 |
| RBMS3-AS2 | -2.78584 | 0.14739 | 0.02539 | 0.311653 |
| CRYZL2P-SEC16B | -2.78801 | 0.561833 | 0.011827 | 0.212783 |
| AC007336.1 | -2.78879 | 0.823212 | 0.003386 | 0.11282 |
| GRHL3 | -2.7903 | 1.934033 | 0.031003 | 0.340778 |
| SPARCL1 | -2.79066 | 9.142429 | 1.32E-05 | 0.00346 |
| CNTN1 | -2.80702 | 6.014334 | 1.01E-05 | 0.002903 |
| STK33 | -2.81587 | 1.917012 | 0.018493 | 0.267127 |
| AL449283.1 | -2.82279 | 0.042807 | 0.006892 | 0.160585 |
| PCDH9 | -2.82676 | 4.203263 | 1.47E-05 | 0.003656 |
| SHISAL1 | -2.82746 | 1.745293 | 0.004078 | 0.123854 |
| MPPED2 | -2.82961 | 3.982996 | 7.07E-05 | 0.0113 |
| TAX1BP3 | -2.8319 | -0.68349 | 0.043923 | 0.400672 |
| NUDT10 | -2.83319 | 2.745787 | 0.009856 | 0.193123 |
| AC007751.1 | -2.83389 | -0.52764 | 0.042232 | 0.393761 |
| HCN4 | -2.83578 | 0.366498 | 0.019941 | 0.27642 |
| MTCO3P11 | -2.83652 | 0.337073 | 0.037755 | 0.372867 |
| TMPRSS13 | -2.83819 | 0.473161 | 0.004611 | 0.132436 |
| RBFOX1 | -2.83868 | 0.257235 | 0.031593 | 0.342615 |
| TAC1 | -2.83995 | -0.54498 | 0.044375 | 0.402709 |
| SFRP1 | -2.84918 | 6.207423 | 9.31E-06 | 0.002734 |
| VCAN-AS1 | -2.85012 | 0.159489 | 0.004662 | 0.133341 |
| AL139805.1 | -2.85031 | 0.879902 | 0.022757 | 0.296986 |
| SCIN | -2.85157 | 3.07329 | 7.53E-05 | 0.011761 |
| GRK5-IT1 | -2.85169 | -0.75986 | 0.042116 | 0.393128 |
| SEMA3D | -2.85476 | 6.59978 | 0.00301 | 0.107046 |
| TRPC4 | -2.85664 | 3.348402 | 0.009244 | 0.187736 |
| CT62 | -2.85785 | 2.144645 | 0.023951 | 0.303528 |
| ADGRL2 | -2.86137 | 6.965721 | 7.69E-06 | 0.002491 |
| AP002884.4 | -2.86332 | -0.74602 | 0.034277 | 0.357213 |
| CSTA | -2.86661 | 1.481513 | 0.001354 | 0.066444 |
| SEZ6L | -2.86816 | 0.031726 | 0.024689 | 0.306926 |
| MMP10 | -2.87176 | 2.214166 | 0.02465 | 0.30656 |
| CCL22 | -2.87464 | 0.713324 | 0.014867 | 0.237888 |
| EMILIN3 | -2.8751 | 0.757069 | 0.026282 | 0.316201 |
| PTCHD1 | -2.8761 | 2.774674 | 0.007402 | 0.166175 |
| SAMD13 | -2.87906 | 1.420345 | 0.022658 | 0.296149 |
| MUC4 | -2.88271 | 2.312097 | 0.000617 | 0.040876 |
| COL22A1 | -2.88273 | 1.743729 | 0.011585 | 0.210879 |
| NLGN1-AS1 | -2.88491 | 1.168751 | 0.030865 | 0.340084 |
| SLC9C1 | -2.88603 | 2.418544 | 0.008846 | 0.183713 |
| REEP2 | -2.88652 | 0.687231 | 0.003925 | 0.122013 |
| LINC02005 | -2.88706 | 1.135361 | 0.03368 | 0.353462 |
| ALDH1A3 | -2.89879 | 7.486573 | 0.000548 | 0.038039 |
| KRT23 | -2.90082 | -0.05087 | 0.024872 | 0.30831 |
| THSD4 | -2.90302 | 6.558345 | 7.69E-07 | 0.000539 |
| PLAG1 | -2.90547 | 2.504864 | 0.000348 | 0.029489 |
| DGKB | -2.90846 | 1.353316 | 0.014204 | 0.232554 |
| PRDX3P4 | -2.91447 | 0.08797 | 0.029649 | 0.33384 |
| Z97200.1 | -2.91946 | 0.797524 | 0.022756 | 0.296986 |
| GFRA1 | -2.91981 | 4.630351 | 1.14E-05 | 0.003159 |
| SAMD3 | -2.93091 | 2.166496 | 0.001029 | 0.056564 |
| TMEM200A | -2.93901 | 3.841671 | 2.16E-05 | 0.005016 |
| PRR16 | -2.94409 | 5.361515 | 0.002342 | 0.092582 |
| PTPRD-AS1 | -2.94645 | -0.43844 | 0.026018 | 0.315052 |
| KIAA1210 | -2.95031 | 1.816003 | 0.024081 | 0.303878 |
| LINC00457 | -2.95425 | 1.241945 | 0.029375 | 0.332562 |
| AC103740.1 | -2.95491 | 0.67908 | 0.007408 | 0.166204 |
| AC022075.1 | -2.95583 | 1.172723 | 0.00343 | 0.113824 |
| LINC01695 | -2.95744 | 2.533205 | 0.003732 | 0.119322 |
| AF165147.1 | -2.95941 | 5.971787 | 0.000811 | 0.04881 |
| C9orf92 | -2.96104 | -0.95603 | 0.048508 | 0.419487 |
| MAPK4 | -2.96139 | 3.129785 | 4.80E-05 | 0.008506 |
| IL23A | -2.96504 | -1.0666 | 0.049108 | 0.421886 |
| TPTEP1 | -2.96508 | 2.512584 | 0.000109 | 0.014841 |
| FRRS1L | -2.96705 | 3.167692 | 0.000435 | 0.033784 |
| LINC01725 | -2.96821 | 2.701637 | 0.006041 | 0.152792 |
| SATB1-AS1 | -2.97039 | 1.653626 | 0.006375 | 0.156371 |
| AC112722.1 | -2.97057 | 0.117941 | 0.004596 | 0.132259 |
| AL049838.1 | -2.97405 | -0.2264 | 0.014096 | 0.231342 |
| GJB2 | -2.97582 | 1.648041 | 0.001668 | 0.074024 |
| KCND3 | -2.97726 | 6.164917 | 7.03E-05 | 0.011287 |
| TCEAL7 | -2.97982 | 1.863608 | 0.001714 | 0.074807 |
| EFS | -2.98224 | 1.647815 | 0.00074 | 0.046312 |
| CEACAM1 | -2.98253 | 3.619344 | 0.000401 | 0.032293 |
| EDNRA | -2.9878 | 5.697573 | 9.76E-08 | 0.000105 |
| NRXN3 | -2.98801 | 5.886063 | 0.001263 | 0.063875 |
| ROBO2 | -2.99568 | 4.129387 | 0.001423 | 0.068378 |
| CHI3L2 | -2.99591 | 1.100658 | 0.002386 | 0.093111 |
| MS4A14 | -2.99726 | 1.738431 | 0.000274 | 0.026052 |
| CPS1 | -2.99905 | 1.738465 | 0.000923 | 0.052282 |
| CAMK4 | -2.99998 | 5.029087 | 0.000152 | 0.01779 |
| SFRP2 | -3.0011 | 5.12289 | 9.09E-08 | 0.000101 |
| HEPACAM2 | -3.00333 | 2.51417 | 0.004193 | 0.125911 |
| JCHAIN | -3.0069 | 5.781429 | 0.000816 | 0.04882 |
| STEAP4 | -3.00802 | 7.297573 | 0.000241 | 0.024081 |
| RASSF6 | -3.01034 | 3.735855 | 1.48E-05 | 0.003656 |
| LPAR1 | -3.01202 | 4.359031 | 5.34E-06 | 0.001911 |
| ITGB6 | -3.01291 | 3.407501 | 8.58E-05 | 0.012881 |
| AC007255.1 | -3.01397 | -0.54458 | 0.027493 | 0.323968 |
| MIPOL1 | -3.01536 | 8.015552 | 5.12E-05 | 0.008889 |
| CYP4B1 | -3.01634 | 0.001528 | 0.021821 | 0.29109 |
| LRRTM4 | -3.01645 | 4.049425 | 0.02363 | 0.301623 |
| AC096677.2 | -3.01846 | 0.448962 | 0.008426 | 0.178217 |
| MFAP5 | -3.01955 | 2.40534 | 0.009763 | 0.192398 |
| AP001094.2 | -3.0218 | 0.68422 | 0.013774 | 0.229671 |
| AL357568.2 | -3.02252 | -0.29699 | 0.02582 | 0.313837 |
| MAGI2 | -3.02258 | 4.24447 | 2.72E-05 | 0.005878 |
| FOXJ1 | -3.02304 | -0.45123 | 0.029046 | 0.331081 |
| SCN2B | -3.02525 | -0.63297 | 0.03486 | 0.359131 |
| GABRG3-AS1 | -3.02587 | 1.560757 | 0.019683 | 0.274795 |
| TXLNB | -3.03026 | 1.821355 | 0.000184 | 0.020175 |
| AC009509.3 | -3.03253 | -1.13733 | 0.025902 | 0.314274 |
| AC104938.1 | -3.03881 | 0.029396 | 0.02997 | 0.335707 |
| KCTD16 | -3.03911 | 1.991785 | 0.000868 | 0.050512 |
| AC110760.1 | -3.04045 | -1.40864 | 0.038157 | 0.374688 |
| AL139393.2 | -3.04119 | 1.039221 | 0.001283 | 0.064517 |
| PATE1 | -3.04319 | 2.794732 | 0.003602 | 0.116872 |
| HECW1 | -3.04589 | 2.418125 | 0.001144 | 0.060529 |
| DNASE2B | -3.04791 | 2.349868 | 0.001515 | 0.070787 |
| PTCHD4 | -3.05179 | 2.376047 | 0.006761 | 0.159859 |
| ANKRD20A7P | -3.05392 | 1.248895 | 0.041596 | 0.390821 |
| RPL32P1 | -3.05501 | -0.01651 | 0.011918 | 0.213441 |
| GSTM2 | -3.05631 | 3.1765 | 0.000664 | 0.043356 |
| AC007001.1 | -3.05735 | -0.88225 | 0.019021 | 0.270223 |
| PIGR | -3.0582 | 3.484138 | 0.004068 | 0.123754 |
| FIBIN | -3.06006 | 4.533132 | 3.31E-06 | 0.001368 |
| AC064799.2 | -3.06502 | 0.851134 | 0.007074 | 0.16296 |
| AL354984.2 | -3.06759 | -0.13743 | 0.006456 | 0.156814 |
| FBN2 | -3.07225 | 3.307223 | 5.74E-05 | 0.009576 |
| TMPRSS11F | -3.07374 | 0.604348 | 0.043429 | 0.398866 |
| FGF10 | -3.07806 | 1.052884 | 0.016902 | 0.255413 |
| LAMC2 | -3.07879 | 2.944303 | 0.000832 | 0.049409 |
| CADM2 | -3.08669 | 4.182198 | 0.04127 | 0.38947 |
| MS4A1 | -3.08688 | 1.521944 | 0.004071 | 0.123754 |
| APOF | -3.09886 | 0.617921 | 0.007987 | 0.173411 |
| GDF15 | -3.10202 | 6.046383 | 4.47E-05 | 0.008126 |
| MYO3B | -3.1035 | 0.220968 | 0.015572 | 0.243676 |
| DCHS2 | -3.10745 | 1.905265 | 0.006257 | 0.154955 |
| RPS17P8 | -3.11263 | 1.774042 | 0.010715 | 0.201007 |
| AP001282.1 | -3.1141 | 0.305749 | 0.024881 | 0.30831 |
| Z93022.1 | -3.11595 | -0.63305 | 0.015011 | 0.239097 |
| AL669831.3 | -3.1201 | -0.82542 | 0.02262 | 0.296149 |
| CCL19 | -3.12464 | 1.124035 | 0.023679 | 0.30183 |
| OR2C3 | -3.12711 | 0.551431 | 0.03938 | 0.381788 |
| SYT9 | -3.12787 | 2.712492 | 0.00291 | 0.104559 |
| DEFB1 | -3.12865 | -0.43354 | 0.048183 | 0.418134 |
| KCNA2 | -3.13054 | -0.81083 | 0.034827 | 0.359131 |
| AC069444.1 | -3.13877 | -0.93324 | 0.047292 | 0.414711 |
| PRG4 | -3.13999 | 0.533479 | 0.002482 | 0.095472 |
| KLHL14 | -3.14426 | 1.142106 | 0.014712 | 0.236687 |
| APOBEC4 | -3.14968 | -0.27554 | 0.012788 | 0.220564 |
| DCDC2C | -3.15151 | -0.42222 | 0.017701 | 0.261262 |
| ABCA8 | -3.15319 | 5.653223 | 5.48E-07 | 0.000428 |
| AC073591.1 | -3.15431 | 0.10254 | 0.029742 | 0.334077 |
| OR13G1 | -3.15471 | -0.33683 | 0.020679 | 0.282394 |
| LINC01697 | -3.16126 | 2.474709 | 0.006822 | 0.160585 |
| C4orf19 | -3.1613 | 0.383422 | 0.001571 | 0.072142 |
| CDHR1 | -3.1627 | 0.019459 | 0.014069 | 0.231226 |
| ZNF474 | -3.16401 | -0.89541 | 0.03419 | 0.35696 |
| SPIB | -3.16806 | -1.30077 | 0.046005 | 0.409485 |
| C10orf113 | -3.1691 | -0.11184 | 0.006094 | 0.153686 |
| CIAPIN1P | -3.17273 | -0.48422 | 0.02559 | 0.312479 |
| MKX | -3.17485 | 4.757549 | 0.003303 | 0.111802 |
| TRIM31 | -3.17582 | -1.16601 | 0.045183 | 0.406287 |
| SLC22A10 | -3.18249 | 0.590753 | 0.019402 | 0.273131 |
| AL008723.3 | -3.18269 | 0.652131 | 0.006663 | 0.15871 |
| LINC01480 | -3.18431 | 1.07044 | 0.001123 | 0.059791 |
| DACT3-AS1 | -3.19225 | -1.2852 | 0.048657 | 0.419715 |
| IGKV1-16 | -3.19575 | -1.29318 | 0.037283 | 0.370677 |
| HOXD10 | -3.19641 | 1.988335 | 0.000615 | 0.040876 |
| AL008723.1 | -3.19933 | 1.415386 | 0.009822 | 0.192944 |
| AP001533.1 | -3.20095 | -0.56843 | 0.006256 | 0.154955 |
| NR2F1 | -3.2014 | 4.406758 | 2.95E-05 | 0.006183 |
| KIAA1211L | -3.20304 | 2.704115 | 0.000223 | 0.023148 |
| AC015922.2 | -3.2063 | -0.76266 | 0.030366 | 0.337552 |
| PLEKHS1 | -3.20802 | 5.918223 | 0.00315 | 0.109723 |
| AC087385.1 | -3.20851 | -0.76072 | 0.034663 | 0.359025 |
| AC069079.1 | -3.20869 | -0.55555 | 0.019144 | 0.271007 |
| C6orf141 | -3.20969 | -0.37289 | 0.016382 | 0.250655 |
| CYTL1 | -3.21489 | 0.124376 | 0.01243 | 0.2174 |
| AL354993.1 | -3.21562 | 1.075317 | 0.000695 | 0.044565 |
| AC108463.3 | -3.21612 | 0.054899 | 0.014959 | 0.238654 |
| AC010478.1 | -3.2174 | 3.504868 | 0.012686 | 0.219302 |
| MTND1P36 | -3.21805 | -0.35682 | 0.040846 | 0.388265 |
| AC006145.1 | -3.22398 | 1.264408 | 0.000779 | 0.04752 |
| HLA-F-AS1 | -3.22814 | 0.48069 | 0.005686 | 0.147398 |
| AL133370.1 | -3.23058 | 0.274753 | 0.010668 | 0.200703 |
| FAM189A2 | -3.23389 | 4.321046 | 0.000413 | 0.032699 |
| TFF3 | -3.23573 | 3.334445 | 0.014272 | 0.233221 |
| AL449403.1 | -3.23865 | -0.61865 | 0.046538 | 0.411763 |
| GABRE | -3.23961 | 3.683471 | 1.16E-05 | 0.003159 |
| P2RX2 | -3.24318 | -0.10931 | 0.014913 | 0.238222 |
| AC013460.1 | -3.24376 | -0.51933 | 0.037292 | 0.370677 |
| ESR1 | -3.24762 | 4.719675 | 1.05E-06 | 0.000643 |
| SLC27A6 | -3.2511 | 2.364514 | 0.010368 | 0.198617 |
| AC026124.1 | -3.25327 | 1.158451 | 0.000736 | 0.046121 |
| DSC3 | -3.25479 | 2.731558 | 0.012415 | 0.217394 |
| COL17A1 | -3.25554 | 2.675105 | 0.000411 | 0.032697 |
| SNAP25 | -3.25566 | 1.691403 | 0.001846 | 0.078296 |
| ABCB1 | -3.25667 | 4.016989 | 7.86E-05 | 0.012048 |
| AC078899.4 | -3.27283 | 0.052276 | 0.021955 | 0.291734 |
| ANKRD26P4 | -3.27372 | -0.94707 | 0.027088 | 0.321949 |
| NRXN1 | -3.27444 | 3.454072 | 0.000855 | 0.050312 |
| GPC5 | -3.27842 | 0.102561 | 0.003304 | 0.111802 |
| TMEM26 | -3.28533 | 4.384167 | 8.74E-06 | 0.00268 |
| AC105450.1 | -3.29151 | -0.22351 | 0.025622 | 0.312642 |
| TBILA | -3.29173 | 2.31449 | 0.001452 | 0.069089 |
| AC073109.1 | -3.29324 | 4.063481 | 0.009321 | 0.187892 |
| CYP4F8 | -3.29651 | 3.208312 | 0.000713 | 0.045331 |
| CADPS | -3.29799 | 4.663664 | 0.000544 | 0.038032 |
| AL354861.3 | -3.29818 | -0.78094 | 0.044449 | 0.402848 |
| AC010904.2 | -3.29884 | -0.9063 | 0.02919 | 0.331892 |
| PRELID3BP8 | -3.30209 | 1.872159 | 0.031943 | 0.344274 |
| HSPA8P16 | -3.30856 | -0.06866 | 0.043328 | 0.398699 |
| CLCA2 | -3.31288 | -1.02619 | 0.048851 | 0.420613 |
| GFRA2 | -3.31638 | 0.27502 | 0.018727 | 0.268576 |
| PCP4L1 | -3.31979 | 0.3752 | 0.003544 | 0.116153 |
| SLIT2-IT1 | -3.32325 | 0.016204 | 0.00599 | 0.151962 |
| AC104695.2 | -3.32673 | -1.33944 | 0.037612 | 0.372101 |
| AC097478.1 | -3.33342 | 2.888517 | 0.005049 | 0.138741 |
| MIR135A2 | -3.34011 | -1.00225 | 0.048942 | 0.421202 |
| AL035706.1 | -3.34458 | -0.75878 | 0.019896 | 0.276125 |
| AC244394.2 | -3.34491 | -1.33386 | 0.042461 | 0.395044 |
| LINC01750 | -3.3468 | -0.42483 | 0.034424 | 0.358093 |
| ANXA3 | -3.3492 | 5.397853 | 0.000284 | 0.026382 |
| TTN | -3.35241 | 9.148225 | 0.000112 | 0.015052 |
| RASAL1 | -3.35495 | -0.86537 | 0.021892 | 0.291411 |
| SERPINB5 | -3.36076 | 0.975193 | 0.010448 | 0.198928 |
| NOV | -3.36214 | 4.779668 | 1.30E-06 | 0.000742 |
| PLG | -3.36299 | 0.82615 | 0.010575 | 0.19948 |
| SPOCD1 | -3.36315 | -0.52034 | 0.011626 | 0.211171 |
| NELL1 | -3.36422 | 2.223593 | 0.000861 | 0.050426 |
| SPINK8 | -3.36503 | -0.07634 | 0.019515 | 0.273939 |
| ZDHHC4P1 | -3.36655 | -1.30769 | 0.040079 | 0.384977 |
| HNRNPA3P15 | -3.3725 | -0.85628 | 0.020083 | 0.277479 |
| PCAT18 | -3.37329 | 4.161157 | 0.043374 | 0.398699 |
| UBE2L2 | -3.37394 | -0.8399 | 0.043185 | 0.398114 |
| SCGN | -3.37677 | 4.226502 | 0.000158 | 0.018136 |
| ETV4 | -3.38204 | 2.046688 | 0.006163 | 0.154356 |
| AL122017.1 | -3.38465 | -1.28682 | 0.029763 | 0.334094 |
| ADGRG5 | -3.38702 | -0.50701 | 0.007056 | 0.162763 |
| WFDC2 | -3.38768 | 1.192209 | 0.000174 | 0.019347 |
| SEC24AP1 | -3.39465 | 0.209036 | 0.001993 | 0.082761 |
| AC008700.1 | -3.40041 | -1.4574 | 0.040664 | 0.387608 |
| LINC01436 | -3.40235 | -0.72876 | 0.037417 | 0.370913 |
| TMEM246 | -3.40385 | 2.644592 | 4.43E-05 | 0.008096 |
| AC034234.1 | -3.40432 | 0.859673 | 0.022406 | 0.295217 |
| AC108206.1 | -3.40505 | 0.377424 | 0.019955 | 0.276491 |
| TMPRSS11E | -3.41342 | 3.150801 | 0.044932 | 0.404661 |
| DIO3 | -3.42149 | -0.81936 | 0.038146 | 0.374688 |
| ZNF300P1 | -3.42604 | 2.553021 | 0.000215 | 0.022717 |
| PURG | -3.4302 | 0.173994 | 0.002734 | 0.10065 |
| AC005692.1 | -3.43194 | -0.44894 | 0.030099 | 0.336223 |
| SYT13 | -3.43364 | 2.811702 | 0.004244 | 0.126634 |
| PRDM8 | -3.43388 | 2.547779 | 0.000285 | 0.026382 |
| RSU1P3 | -3.43818 | -0.79312 | 0.01152 | 0.210147 |
| OR52T1P | -3.43883 | -0.91917 | 0.04997 | 0.425697 |
| AC144450.1 | -3.44008 | 2.189167 | 0.00521 | 0.140913 |
| AP003117.1 | -3.44089 | -0.9475 | 0.027468 | 0.323897 |
| INHBE | -3.44403 | -1.4225 | 0.039658 | 0.382969 |
| CLVS1 | -3.44853 | -1.41667 | 0.045366 | 0.407082 |
| PEBP4 | -3.45297 | 1.412396 | 0.001221 | 0.062511 |
| CHRM3 | -3.45477 | 3.682457 | 4.42E-05 | 0.008096 |
| TCEAL2 | -3.45765 | 1.12023 | 0.001507 | 0.070719 |
| IGSF11 | -3.46028 | 0.010338 | 0.036949 | 0.369765 |
| NDUFAF4P3 | -3.46688 | -1.06334 | 0.016026 | 0.247627 |
| SPAG6 | -3.46708 | 1.398481 | 0.004889 | 0.136321 |
| SLC26A5 | -3.46958 | 3.269401 | 0.000361 | 0.030262 |
| ZNF204P | -3.47643 | 2.792837 | 3.56E-05 | 0.007036 |
| VIPR2 | -3.47765 | 2.083663 | 0.001344 | 0.06622 |
| AC006566.1 | -3.47866 | 0.419602 | 0.027304 | 0.322957 |
| AC079336.2 | -3.4822 | -1.0573 | 0.027483 | 0.323954 |
| ZNF676 | -3.49065 | -0.05885 | 0.009731 | 0.192322 |
| THSD7B | -3.49228 | 2.159345 | 0.00048 | 0.036029 |
| AC099343.2 | -3.49235 | -0.50375 | 0.024829 | 0.307997 |
| AP000533.2 | -3.49791 | -1.37892 | 0.045752 | 0.409041 |
| FAM167A | -3.50007 | 1.406918 | 9.00E-05 | 0.013317 |
| KRT13 | -3.50138 | 0.541792 | 0.01043 | 0.198805 |
| WT1-AS | -3.50531 | 0.856453 | 0.000534 | 0.037984 |
| CALCA | -3.50935 | 0.891976 | 0.016909 | 0.255413 |
| LEFTY2 | -3.51294 | 0.732231 | 0.000838 | 0.049658 |
| AL139156.3 | -3.51563 | -1.58311 | 0.040203 | 0.385543 |
| RTN1 | -3.5219 | 5.757735 | 3.35E-06 | 0.001372 |
| KRT7 | -3.52483 | 2.73557 | 0.001142 | 0.060529 |
| CHST9 | -3.52559 | 3.722149 | 0.001146 | 0.060536 |
| ANO3 | -3.52632 | -0.2726 | 0.015042 | 0.239316 |
| SCG2 | -3.52644 | 5.378929 | 0.000604 | 0.040438 |
| C2orf40 | -3.52873 | 2.579761 | 0.00027 | 0.025835 |
| CXCL14 | -3.53337 | 5.326336 | 0.000844 | 0.0499 |
| CTAGE9 | -3.53527 | 2.393591 | 0.003358 | 0.112648 |
| MALRD1 | -3.53961 | -0.10337 | 0.005698 | 0.147485 |
| AC108751.4 | -3.53964 | 0.443021 | 0.012482 | 0.217969 |
| NDNF | -3.54009 | 4.84905 | 0.000228 | 0.023223 |
| S100G | -3.54791 | -2.12628 | 0.049815 | 0.424484 |
| IQSEC3 | -3.55006 | 0.148894 | 0.001292 | 0.064687 |
| ARSJ | -3.55219 | 3.828664 | 3.46E-06 | 0.001398 |
| MIR4300HG | -3.55228 | 1.165002 | 0.003254 | 0.110781 |
| LPAR3 | -3.5524 | 4.952031 | 0.000686 | 0.044328 |
| AL445123.1 | -3.55649 | 0.480119 | 0.011406 | 0.208745 |
| PAGE4 | -3.55683 | 2.206854 | 0.010245 | 0.197348 |
| AC136424.2 | -3.55769 | -1.57138 | 0.03 | 0.335707 |
| AJAP1 | -3.56597 | 1.735413 | 5.79E-05 | 0.009612 |
| IGFN1 | -3.57399 | 1.041646 | 0.000503 | 0.036842 |
| KRT6A | -3.57472 | -1.12932 | 0.027698 | 0.325106 |
| RPS15AP34 | -3.57598 | -0.66702 | 0.049349 | 0.422819 |
| NTNG1 | -3.57836 | 2.587455 | 0.000114 | 0.015074 |
| AC020571.1 | -3.58435 | 3.58062 | 0.002735 | 0.10065 |
| NTF4 | -3.58794 | -0.53528 | 0.022107 | 0.292731 |
| AL391097.1 | -3.59032 | 0.699824 | 0.003735 | 0.119322 |
| NXPH4 | -3.59797 | 3.717215 | 0.001461 | 0.06932 |
| WDR72 | -3.60085 | -1.30703 | 0.043101 | 0.398085 |
| C2CD4A | -3.6044 | 2.183814 | 0.041335 | 0.389788 |
| IGFBP7-AS1 | -3.61415 | -1.50011 | 0.04004 | 0.384928 |
| TM4SF18 | -3.61497 | 5.105047 | 7.96E-06 | 0.002546 |
| LRRC7 | -3.61672 | 5.077662 | 0.001625 | 0.073334 |
| IL24 | -3.61704 | -0.64876 | 0.011995 | 0.214043 |
| WIF1 | -3.62229 | 1.731762 | 0.014321 | 0.233376 |
| ELF5 | -3.62911 | 3.62564 | 0.001671 | 0.074024 |
| ANPEP | -3.63585 | 6.129848 | 0.004428 | 0.130544 |
| LINC00161 | -3.63594 | 2.9477 | 0.002497 | 0.095505 |
| PLA2G4D | -3.64222 | 0.348344 | 0.017355 | 0.25896 |
| HOXA7 | -3.64485 | -0.19798 | 0.012156 | 0.215026 |
| CLEC9A | -3.64762 | -1.06398 | 0.039576 | 0.382541 |
| PADI3 | -3.65198 | -1.08695 | 0.025216 | 0.310506 |
| AC243960.1 | -3.65348 | -0.63052 | 0.034779 | 0.359131 |
| DUSP15 | -3.65726 | -1.73677 | 0.02328 | 0.300391 |
| AC008056.2 | -3.65919 | -0.49064 | 0.037704 | 0.37283 |
| AC106881.1 | -3.66404 | -1.0747 | 0.019553 | 0.274063 |
| SDC2 | -3.67867 | 7.732496 | 1.94E-07 | 0.000186 |
| GRIN3A | -3.68034 | 5.287309 | 0.000148 | 0.017641 |
| CLCA4 | -3.6855 | -0.58788 | 0.010338 | 0.198223 |
| OR51A8P | -3.6917 | 0.044581 | 0.006157 | 0.154356 |
| CAPN8 | -3.69667 | -0.85741 | 0.0412 | 0.38947 |
| AC080013.6 | -3.69675 | -1.70021 | 0.036884 | 0.36969 |
| ISL1 | -3.69786 | 3.210927 | 0.002177 | 0.088027 |
| EFCAB1 | -3.70962 | -0.01727 | 0.014192 | 0.232464 |
| ARHGAP42P1 | -3.71079 | 0.060486 | 0.006919 | 0.161013 |
| AC007666.1 | -3.71391 | -1.0241 | 0.019789 | 0.275637 |
| ZNF157 | -3.71984 | -1 | 0.03394 | 0.354945 |
| GRIK3 | -3.72267 | 0.877088 | 0.001563 | 0.071971 |
| TNS4 | -3.72821 | 2.404421 | 0.000735 | 0.046121 |
| MIR4500HG | -3.73554 | 0.444826 | 0.006662 | 0.15871 |
| RPL7L1P9 | -3.73812 | 0.07534 | 0.001374 | 0.066958 |
| GTF2F2P1 | -3.74177 | 0.237557 | 0.000402 | 0.032293 |
| FMO1 | -3.74905 | -1.00618 | 0.015848 | 0.2459 |
| AC006270.3 | -3.74999 | -0.81039 | 0.012405 | 0.217394 |
| NWD2 | -3.75139 | 2.442576 | 0.005633 | 0.146628 |
| OVCH1 | -3.75165 | -0.65566 | 0.018719 | 0.268576 |
| AL357793.2 | -3.7519 | -1.67403 | 0.041448 | 0.390164 |
| LUZP2 | -3.7522 | 4.85928 | 3.10E-05 | 0.006423 |
| AL157394.1 | -3.75303 | 0.868413 | 0.000448 | 0.034365 |
| PIP | -3.75475 | 2.324739 | 0.033393 | 0.352064 |
| CACNA1A | -3.75594 | 1.741738 | 0.013367 | 0.225549 |
| ELAVL4 | -3.75793 | 0.111494 | 0.006523 | 0.157274 |
| AP000688.2 | -3.76097 | -0.52491 | 0.01539 | 0.242604 |
| SLITRK2 | -3.76901 | 0.331585 | 0.002905 | 0.104503 |
| FER1L6 | -3.76945 | -0.18274 | 0.005326 | 0.142483 |
| ADAD1P2 | -3.77116 | -1.63182 | 0.037339 | 0.370784 |
| ARPP21 | -3.78327 | -0.2703 | 0.022261 | 0.294209 |
| AC010680.1 | -3.78511 | -1.13756 | 0.039587 | 0.382541 |
| PMP2 | -3.78962 | 2.144129 | 0.000526 | 0.037792 |
| TYRP1 | -3.80487 | 0.859827 | 0.002491 | 0.095481 |
| AP000238.1 | -3.805 | -0.49594 | 0.016738 | 0.254398 |
| RNU12-2P | -3.80986 | -1.92167 | 0.049659 | 0.423993 |
| IL1RAPL2 | -3.81644 | -0.92599 | 0.013369 | 0.225549 |
| DCLRE1CP1 | -3.81888 | 0.444204 | 0.012704 | 0.219504 |
| ZNF385D | -3.82122 | 2.311454 | 6.47E-05 | 0.010529 |
| RPL12P30 | -3.82138 | -0.49136 | 0.02722 | 0.322736 |
| LGR5 | -3.82273 | -0.00891 | 0.012533 | 0.2184 |
| LGALS17A | -3.82509 | -1.10648 | 0.025679 | 0.313156 |
| GREM1 | -3.834 | 6.899969 | 7.84E-05 | 0.012048 |
| HHIP | -3.8365 | 5.6382 | 9.30E-07 | 0.000614 |
| SPTSSB | -3.84025 | 3.722144 | 3.84E-05 | 0.007411 |
| OR52Y1P | -3.84157 | 1.604092 | 0.005306 | 0.142483 |
| LY86-AS1 | -3.8451 | -1.90477 | 0.0475 | 0.415773 |
| HMGB3P10 | -3.84652 | -1.90943 | 0.027983 | 0.326784 |
| LINC01266 | -3.84918 | 2.898009 | 0.001171 | 0.061232 |
| AL157392.4 | -3.85086 | -1.31946 | 0.010607 | 0.199861 |
| HUS1B | -3.8526 | -1.31788 | 0.03377 | 0.354067 |
| THAP12P2 | -3.85517 | 0.103652 | 0.003052 | 0.107636 |
| CARM1P1 | -3.85757 | 0.78427 | 0.001726 | 0.074851 |
| SERPINB11 | -3.85879 | 1.632305 | 0.002788 | 0.101882 |
| MUCL1 | -3.86168 | 0.178001 | 0.024146 | 0.304316 |
| AL390816.2 | -3.86287 | -1.29824 | 0.034712 | 0.359025 |
| AC007159.1 | -3.86655 | -0.2894 | 0.041214 | 0.38947 |
| AC044810.2 | -3.87389 | -0.18688 | 0.004542 | 0.131422 |
| AC130404.1 | -3.8744 | -0.16871 | 0.02111 | 0.285351 |
| AC108463.1 | -3.87509 | -1.09142 | 0.02265 | 0.296149 |
| AC245100.1 | -3.87914 | -1.54383 | 0.031497 | 0.342118 |
| EYA4 | -3.88384 | 3.383507 | 0.00032 | 0.028262 |
| KCND3-AS1 | -3.89945 | 0.400828 | 0.009275 | 0.187736 |
| THSD4-AS1 | -3.90039 | 1.814806 | 0.000253 | 0.024647 |
| KIF5A | -3.90817 | 1.761196 | 0.001467 | 0.069421 |
| CST1 | -3.90844 | 1.579074 | 0.000217 | 0.022749 |
| AC017007.2 | -3.90998 | -1.85273 | 0.023665 | 0.30183 |
| FAM213AP1 | -3.90998 | -1.53546 | 0.040131 | 0.385263 |
| PTPRZ1 | -3.9103 | 2.291946 | 1.60E-05 | 0.003894 |
| OR51A5P | -3.92033 | 0.240306 | 0.023882 | 0.303152 |
| PCYT1B | -3.92055 | -0.13285 | 0.003203 | 0.110181 |
| MUC16 | -3.9223 | -0.37725 | 0.028591 | 0.329116 |
| AC016730.1 | -3.93094 | 4.377284 | 0.00334 | 0.112578 |
| REM1 | -3.93426 | 0.153533 | 0.001081 | 0.058432 |
| ADTRP | -3.93993 | 1.339543 | 0.000813 | 0.04882 |
| VDAC2P1 | -3.94883 | 1.159929 | 0.004756 | 0.134568 |
| KANK4 | -3.94943 | 0.18528 | 0.001431 | 0.068436 |
| RN7SKP240 | -3.95176 | -1.8342 | 0.03667 | 0.368495 |
| MMP13 | -3.95265 | -0.02791 | 0.005344 | 0.142859 |
| EGF | -3.95389 | 3.917407 | 0.000147 | 0.017544 |
| SLC18A1 | -3.95445 | 1.523734 | 0.024859 | 0.308264 |
| GABRP | -3.95669 | 1.763944 | 0.000216 | 0.022717 |
| RN7SL68P | -3.96432 | -1.47659 | 0.023232 | 0.300391 |
| AC026355.2 | -3.96532 | -0.50183 | 0.010915 | 0.203321 |
| CFAP221 | -3.98405 | -1.45885 | 0.025924 | 0.314318 |
| TPH1 | -3.98456 | 4.043084 | 1.71E-05 | 0.004112 |
| ZNF812P | -3.99682 | 1.384591 | 0.000328 | 0.028576 |
| NIPAL4 | -3.99947 | -0.46328 | 0.003108 | 0.108752 |
| OR51C4P | -4.00772 | -0.59926 | 0.023133 | 0.299763 |
| ITGB8 | -4.01859 | 6.133942 | 1.83E-10 | 4.84E-07 |
| CACNA1C-AS1 | -4.02288 | -1.45406 | 0.021633 | 0.28925 |
| ZNF90 | -4.0267 | 0.791518 | 0.002453 | 0.094985 |
| LRRC37A7P | -4.02994 | -0.93522 | 0.0082 | 0.175507 |
| FAM83E | -4.03314 | 2.10867 | 0.000426 | 0.033339 |
| AL020997.2 | -4.03598 | -1.42328 | 0.017878 | 0.262542 |
| GAS1RR | -4.04304 | 1.306512 | 0.000491 | 0.036269 |
| SERPINA3 | -4.04475 | 1.169802 | 0.007109 | 0.162997 |
| KRT17 | -4.04954 | 0.157972 | 0.006683 | 0.158983 |
| AC091607.1 | -4.05029 | -1.74741 | 0.029308 | 0.33243 |
| PPIAP10 | -4.05183 | -1.40396 | 0.019034 | 0.270223 |
| KCNAB1-AS2 | -4.05485 | -1.13404 | 0.021567 | 0.288859 |
| HLX-AS1 | -4.05904 | -1.39721 | 0.023226 | 0.300391 |
| AC012236.1 | -4.06035 | -2.16071 | 0.046945 | 0.41299 |
| APOD | -4.06586 | 6.785078 | 1.17E-13 | 1.00E-09 |
| AC093001.1 | -4.06796 | 5.036907 | 0.008922 | 0.184267 |
| CLDN1 | -4.07058 | 5.056191 | 7.73E-10 | 1.66E-06 |
| AL450344.2 | -4.07148 | -0.54383 | 0.005465 | 0.144617 |
| AL138899.1 | -4.07373 | -1.14334 | 0.008357 | 0.17763 |
| SLC30A8 | -4.088 | 1.862068 | 0.00329 | 0.11167 |
| AC005336.2 | -4.09941 | 3.436878 | 0.004127 | 0.124681 |
| SNORD113-3 | -4.10116 | -1.36838 | 0.009271 | 0.187736 |
| GRIN2A | -4.10314 | -0.22556 | 0.010398 | 0.198761 |
| SNORA70E | -4.10903 | -1.11052 | 0.014613 | 0.235764 |
| GRM3 | -4.11113 | -0.68205 | 0.011117 | 0.205956 |
| PGC | -4.11352 | 2.65746 | 4.40E-05 | 0.008096 |
| HRASLS5 | -4.11676 | -0.88031 | 0.006626 | 0.158657 |
| TFF1 | -4.11938 | 1.640889 | 0.000977 | 0.054127 |
| SMIM22 | -4.12635 | 0.670057 | 0.003607 | 0.116872 |
| PLCE1-AS2 | -4.13325 | -1.06835 | 0.01502 | 0.239097 |
| LINC01790 | -4.13784 | 3.490442 | 0.002868 | 0.103792 |
| AC092490.1 | -4.13963 | -2.09554 | 0.028035 | 0.32679 |
| AC141930.1 | -4.14052 | 1.317578 | 0.005258 | 0.141733 |
| KRT15 | -4.14245 | 3.061901 | 0.000535 | 0.037984 |
| AC116096.1 | -4.14313 | 1.492367 | 0.000427 | 0.033339 |
| GSTM1 | -4.14588 | 1.076196 | 0.000583 | 0.039436 |
| MGAM | -4.149 | 1.072537 | 0.000604 | 0.040438 |
| NLRP2 | -4.14936 | -0.84248 | 0.012597 | 0.218706 |
| PTN | -4.14948 | 4.982084 | 4.51E-06 | 0.001734 |
| INSM1 | -4.15125 | 1.53337 | 0.00028 | 0.026292 |
| AC023908.1 | -4.15186 | -1.33111 | 0.040813 | 0.388122 |
| OR51S1 | -4.15391 | 0.452223 | 0.01104 | 0.205093 |
| ASCL1 | -4.15449 | 1.533392 | 0.004755 | 0.134568 |
| TSPAN19 | -4.16728 | 1.155827 | 0.014352 | 0.233643 |
| CRISP3 | -4.17029 | 5.060223 | 5.24E-05 | 0.008995 |
| REG1A | -4.1791 | 1.900623 | 0.018619 | 0.268146 |
| MAEL | -4.18708 | -0.62649 | 0.005162 | 0.139887 |
| SLC7A14 | -4.19442 | 0.09574 | 0.000617 | 0.040876 |
| AL354733.1 | -4.19604 | -1.27717 | 0.038772 | 0.377485 |
| FCF1P8 | -4.19667 | -1.61933 | 0.020357 | 0.279814 |
| AC104971.1 | -4.19755 | -0.42011 | 0.004173 | 0.125511 |
| HSPA8P4 | -4.21231 | -2.04345 | 0.03261 | 0.348198 |
| FAM81B | -4.21675 | -0.57145 | 0.009775 | 0.192529 |
| AC010894.3 | -4.22229 | 0.948473 | 2.40E-05 | 0.005463 |
| TARID | -4.22271 | 1.151499 | 0.004495 | 0.13112 |
| AC005064.1 | -4.22776 | 3.454736 | 0.000667 | 0.043458 |
| LINC01821 | -4.23674 | -0.55804 | 0.023742 | 0.302152 |
| EMX2 | -4.23687 | 1.091029 | 0.009884 | 0.193454 |
| AC139722.1 | -4.2459 | -1.23671 | 0.035928 | 0.365223 |
| KRT4 | -4.24966 | -1.56964 | 0.021026 | 0.285351 |
| OR51A3P | -4.25014 | 0.914656 | 0.008868 | 0.183729 |
| RBP4 | -4.25052 | 2.37893 | 0.000873 | 0.050756 |
| OR51N1P | -4.25162 | 0.70263 | 0.015266 | 0.241314 |
| LINC00857 | -4.25749 | -0.38979 | 0.00131 | 0.065139 |
| AC013652.2 | -4.25749 | -0.75028 | 0.009305 | 0.187892 |
| AC037441.1 | -4.25968 | -2.00226 | 0.020479 | 0.280816 |
| AQP9 | -4.26412 | 3.449064 | 1.29E-05 | 0.003414 |
| PLP1 | -4.26444 | 3.416741 | 0.000288 | 0.026382 |
| TRIM77BP | -4.27154 | -1.9893 | 0.03075 | 0.339401 |
| SORCS1 | -4.27523 | 1.766784 | 2.65E-06 | 0.001282 |
| FAM83B | -4.2798 | 1.30302 | 0.000289 | 0.026382 |
| NXPE4 | -4.28024 | 2.171037 | 0.001591 | 0.072593 |
| AC104365.2 | -4.28083 | 2.986248 | 0.005485 | 0.145028 |
| DCDC2 | -4.2862 | 2.991584 | 2.30E-05 | 0.005298 |
| DIO2-AS1 | -4.28747 | -1.21354 | 0.028842 | 0.330485 |
| PPP1R1A | -4.29955 | -1.22167 | 0.010495 | 0.199191 |
| SOX14 | -4.30162 | -1.19438 | 0.013113 | 0.223646 |
| AC010638.1 | -4.3061 | -0.16961 | 0.00209 | 0.085246 |
| GRIA2 | -4.3093 | 1.424457 | 0.000213 | 0.022696 |
| TRABD2A | -4.31045 | 2.425433 | 0.000102 | 0.014478 |
| PIRT | -4.3117 | 0.085543 | 0.000926 | 0.052282 |
| OR9A3P | -4.31906 | -1.95397 | 0.0385 | 0.376654 |
| CACNA1C-AS3 | -4.32104 | -1.95131 | 0.027881 | 0.326498 |
| TAGLN3 | -4.32397 | -1.19123 | 0.012526 | 0.218396 |
| CFTR | -4.3343 | 3.473629 | 0.010809 | 0.202003 |
| SERPINE4P | -4.33771 | 0.231915 | 0.005635 | 0.146628 |
| AC008758.2 | -4.34534 | -1.15927 | 0.006459 | 0.156814 |
| ODAM | -4.34595 | -0.66664 | 0.007611 | 0.169093 |
| SLITRK3 | -4.34639 | 1.433931 | 0.003593 | 0.116872 |
| TEPP | -4.35463 | -1.14793 | 0.009566 | 0.191135 |
| AL158151.1 | -4.35597 | -1.9292 | 0.01961 | 0.274369 |
| RNU7-57P | -4.36023 | -0.12041 | 0.00445 | 0.131 |
| AC126323.6 | -4.36632 | -1.13879 | 0.048064 | 0.417896 |
| CLEC1B | -4.37785 | -1.90159 | 0.028704 | 0.329966 |
| AP001610.2 | -4.38507 | -0.61766 | 0.037784 | 0.372948 |
| AC027335.1 | -4.38563 | -1.90864 | 0.025696 | 0.313156 |
| ANKS4B | -4.38662 | -0.09342 | 0.004102 | 0.124368 |
| AL158166.2 | -4.40852 | -1.44314 | 0.015962 | 0.24675 |
| RNU4-59P | -4.41127 | -1.0968 | 0.014307 | 0.23337 |
| OR7E154P | -4.41396 | -1.1123 | 0.006863 | 0.160585 |
| AC124854.1 | -4.42212 | -0.81879 | 0.008426 | 0.178217 |
| LINC00276 | -4.42234 | 3.589593 | 0.000991 | 0.054812 |
| DACT2 | -4.4286 | 0.700376 | 0.00078 | 0.04752 |
| AC087516.2 | -4.42884 | -0.20965 | 0.003287 | 0.11167 |
| RNU6-35P | -4.43298 | -1.86371 | 0.049043 | 0.421619 |
| S100B | -4.43813 | 1.375853 | 0.000124 | 0.015668 |
| HS3ST3A1 | -4.43944 | 0.087339 | 0.000481 | 0.036029 |
| AC010983.1 | -4.44213 | 3.88839 | 0.001623 | 0.073334 |
| ZNF883 | -4.45974 | 3.010653 | 8.11E-08 | 9.55E-05 |
| OR51A7 | -4.46405 | -0.56471 | 0.009364 | 0.18865 |
| FCRL2 | -4.46646 | -1.84353 | 0.046883 | 0.412921 |
| AC093305.1 | -4.47329 | -0.54168 | 0.007907 | 0.172543 |
| IL13RA2 | -4.48642 | 3.012919 | 0.000102 | 0.014478 |
| AC016877.3 | -4.48836 | -1.37303 | 0.009273 | 0.187736 |
| RNA5SP490 | -4.4999 | -1.04888 | 0.005715 | 0.147817 |
| KRT5 | -4.50047 | 3.242904 | 0.000334 | 0.028777 |
| CTSE | -4.50838 | -1.80063 | 0.018343 | 0.266191 |
| CNKSR2 | -4.51105 | 4.853539 | 5.08E-05 | 0.008889 |
| FGF14-AS2 | -4.51251 | -0.30666 | 0.006929 | 0.161028 |
| SGIP1 | -4.51382 | 4.158379 | 1.15E-11 | 4.09E-08 |
| MYOZ3 | -4.52992 | 1.491815 | 0.000414 | 0.032711 |
| LPA | -4.53686 | 0.525819 | 0.000606 | 0.040438 |
| AP002759.1 | -4.54293 | -0.9952 | 0.041573 | 0.390804 |
| NSG2 | -4.54942 | 1.599045 | 0.000121 | 0.015419 |
| TPO | -4.55098 | 2.388406 | 1.16E-05 | 0.003159 |
| UOX | -4.55211 | -0.48647 | 0.002457 | 0.095024 |
| AC079075.1 | -4.55872 | -0.26592 | 0.01791 | 0.262787 |
| RNU6-1128P | -4.56632 | -2.39879 | 0.046522 | 0.411743 |
| CYYR1-AS1 | -4.56681 | 2.021895 | 0.001245 | 0.063203 |
| SLC16A12 | -4.5675 | 1.462589 | 0.000618 | 0.040876 |
| TP63 | -4.56863 | 3.348304 | 3.12E-06 | 0.001344 |
| LINC01579 | -4.5911 | -2.39099 | 0.044714 | 0.404069 |
| AL513164.1 | -4.59221 | -2.39017 | 0.03889 | 0.378211 |
| ORM2 | -4.60086 | 0.565068 | 0.012184 | 0.215275 |
| PPIAP20 | -4.60511 | -2.37331 | 0.038693 | 0.377258 |
| ACSM1 | -4.60982 | 6.744638 | 0.000422 | 0.033152 |
| CEACAM5 | -4.61022 | 0.111542 | 0.020302 | 0.279393 |
| TM6SF2 | -4.61182 | -2.36835 | 0.041225 | 0.38947 |
| AC100814.1 | -4.61245 | -2.3718 | 0.0439 | 0.400672 |
| TRIM29 | -4.61426 | 3.251537 | 0.000192 | 0.020894 |
| AC009806.1 | -4.62903 | -2.35731 | 0.037063 | 0.369947 |
| ADCYAP1 | -4.63412 | 0.251544 | 0.002713 | 0.100592 |
| AC008250.1 | -4.63496 | -1.70238 | 0.020867 | 0.28387 |
| AC027308.1 | -4.64028 | -0.18869 | 0.026292 | 0.316209 |
| AC025434.1 | -4.6532 | 0.751174 | 0.00625 | 0.154955 |
| C2orf70 | -4.6712 | -1.67273 | 0.00771 | 0.170527 |
| AC008808.2 | -4.67185 | -0.8734 | 0.027656 | 0.324938 |
| TIMD4 | -4.67306 | 0.139315 | 0.002362 | 0.092805 |
| EMX2OS | -4.67895 | 0.310323 | 0.004454 | 0.131 |
| LINC02492 | -4.68176 | 0.004338 | 0.002096 | 0.085403 |
| AC013268.1 | -4.68501 | -2.33032 | 0.032718 | 0.348474 |
| AC009063.2 | -4.68588 | -1.202 | 0.01554 | 0.243401 |
| ENPP6 | -4.69489 | 1.509228 | 8.37E-05 | 0.012728 |
| OR10A5 | -4.69835 | -2.29833 | 0.049124 | 0.421886 |
| MLIP | -4.70234 | -2.29838 | 0.040791 | 0.388021 |
| HSD17B6 | -4.70285 | 4.895175 | 1.45E-08 | 2.08E-05 |
| IL1RL2 | -4.70387 | -0.84672 | 0.004497 | 0.13112 |
| OR51F2 | -4.70872 | 1.275912 | 0.004427 | 0.130544 |
| AC008687.2 | -4.71356 | -2.29652 | 0.042896 | 0.397368 |
| ALOX15B | -4.71822 | 6.406812 | 2.49E-05 | 0.005616 |
| LINC02392 | -4.71848 | -2.30592 | 0.044483 | 0.402977 |
| COL6A4P1 | -4.72575 | -2.29265 | 0.029757 | 0.334094 |
| AMER3 | -4.74785 | 0.08184 | 0.017631 | 0.260708 |
| RNU7-172P | -4.75792 | -2.26009 | 0.047672 | 0.416358 |
| SP8 | -4.75803 | 0.516421 | 0.001565 | 0.071984 |
| KCNJ1 | -4.76334 | -2.25871 | 0.027437 | 0.323711 |
| OR51F5P | -4.76335 | -0.52572 | 0.005941 | 0.151162 |
| AC008056.1 | -4.76344 | 0.365324 | 0.001696 | 0.074502 |
| AC048380.1 | -4.76809 | -2.24922 | 0.032633 | 0.348198 |
| GRIN2B | -4.771 | -0.08544 | 0.003962 | 0.122487 |
| LINC01567 | -4.77878 | -0.78127 | 0.004513 | 0.131251 |
| HNF4A | -4.78943 | -0.52169 | 0.018626 | 0.268146 |
| AL139393.1 | -4.79916 | -2.2338 | 0.039702 | 0.38328 |
| AC023906.3 | -4.80108 | -2.23417 | 0.029384 | 0.332562 |
| AC064875.1 | -4.80346 | 3.414229 | 3.28E-06 | 0.001368 |
| ENOX1-AS2 | -4.80506 | -1.56052 | 0.00563 | 0.146628 |
| SNORD114-31 | -4.8052 | -2.22128 | 0.039818 | 0.38397 |
| AC007639.1 | -4.80797 | -2.22577 | 0.026994 | 0.321447 |
| AC109779.1 | -4.81032 | 4.373998 | 0.000412 | 0.032699 |
| AC073062.1 | -4.81416 | 3.982152 | 0.00045 | 0.034365 |
| LINC01918 | -4.81597 | -1.09702 | 0.004653 | 0.133198 |
| AP000977.1 | -4.82518 | -1.09866 | 0.001658 | 0.073968 |
| TFAP2B | -4.85583 | -1.5085 | 0.024137 | 0.304316 |
| C1orf140 | -4.86764 | -2.17808 | 0.022645 | 0.296149 |
| ALDH1A2 | -4.8678 | 5.160553 | 2.18E-09 | 4.17E-06 |
| RNU6-26P | -4.88241 | -1.49203 | 0.006226 | 0.154706 |
| RPL13AP17 | -4.88257 | -0.40487 | 0.003876 | 0.121042 |
| FP700111.1 | -4.89084 | -2.15142 | 0.042288 | 0.394064 |
| LINC00923 | -4.89855 | -0.39178 | 0.002011 | 0.083115 |
| AC005972.2 | -4.899 | -2.16165 | 0.02299 | 0.299084 |
| AC078922.1 | -4.90215 | -1.47442 | 0.026668 | 0.319293 |
| TMPRSS12 | -4.90686 | -1.477 | 0.011186 | 0.206788 |
| AC112721.1 | -4.90697 | -2.14671 | 0.036217 | 0.366191 |
| AL356258.1 | -4.90706 | -1.0221 | 0.001258 | 0.063725 |
| MMP26 | -4.90749 | 2.079317 | 0.00455 | 0.131441 |
| KERA | -4.90853 | -1.01188 | 0.002477 | 0.095472 |
| RNU6-536P | -4.91034 | -2.14226 | 0.024529 | 0.306233 |
| ABCB11 | -4.91049 | 0.19911 | 0.0078 | 0.171524 |
| RNU6-83P | -4.91799 | -2.14957 | 0.021099 | 0.285351 |
| AL590438.1 | -4.91961 | -1.47101 | 0.006384 | 0.156371 |
| XKR4 | -4.92536 | 1.553665 | 1.48E-05 | 0.003656 |
| RNU6-851P | -4.93296 | -2.12655 | 0.037608 | 0.372101 |
| AQP2 | -4.93346 | -0.1237 | 0.014668 | 0.23621 |
| LDLRAD1 | -4.94597 | -2.11505 | 0.020496 | 0.280816 |
| CNR1 | -4.9564 | 1.457576 | 0.000112 | 0.015031 |
| ATE1-AS1 | -4.95983 | -0.63054 | 0.001097 | 0.058987 |
| CLDN2 | -4.96862 | 0.43501 | 0.001168 | 0.061232 |
| IL12A | -4.97487 | -2.10028 | 0.018157 | 0.264492 |
| SH3TC2-DT | -4.97666 | -2.0908 | 0.035006 | 0.359897 |
| MMP7 | -4.98685 | 5.152891 | 1.91E-05 | 0.004533 |
| CAPN6 | -4.99652 | 3.003172 | 2.50E-06 | 0.001231 |
| AC106865.1 | -5.00451 | -0.60032 | 0.00184 | 0.078296 |
| LINC00621 | -5.00531 | -2.06079 | 0.049311 | 0.422814 |
| KRT7-AS | -5.01374 | -0.0797 | 0.0008 | 0.048322 |
| SLC22A24 | -5.01751 | -0.56313 | 0.013275 | 0.225242 |
| OR51A6P | -5.03218 | 0.154935 | 0.002752 | 0.100783 |
| GNAS-AS1 | -5.03522 | -2.05014 | 0.024755 | 0.307414 |
| OR51H1 | -5.04484 | 0.888718 | 0.000897 | 0.051622 |
| CYP4F2 | -5.05925 | 1.229552 | 0.003501 | 0.115282 |
| CADM3 | -5.06428 | 0.503865 | 9.39E-05 | 0.013603 |
| AC011601.1 | -5.06915 | -2.02546 | 0.020146 | 0.277799 |
| AC017002.3 | -5.07995 | -1.32533 | 0.004807 | 0.135343 |
| AP003063.1 | -5.08801 | -2.01403 | 0.019564 | 0.274063 |
| AC021088.1 | -5.11263 | -1.98104 | 0.024762 | 0.307414 |
| AC005410.2 | -5.11513 | -1.98712 | 0.015849 | 0.2459 |
| Z82210.1 | -5.11805 | -0.48551 | 0.012272 | 0.216162 |
| MYH1 | -5.11921 | -1.98866 | 0.027355 | 0.32311 |
| AC138305.1 | -5.12658 | 2.756228 | 5.58E-06 | 0.001936 |
| LINC02580 | -5.13201 | -1.98192 | 0.046486 | 0.411743 |
| AL096803.1 | -5.13268 | 1.45879 | 0.000855 | 0.050312 |
| AC114284.1 | -5.1419 | 3.143404 | 7.61E-06 | 0.002489 |
| LINC01088 | -5.14696 | 4.538034 | 2.54E-07 | 0.00023 |
| LINC01587 | -5.14964 | -1.95814 | 0.048093 | 0.417896 |
| HOXB5 | -5.1551 | -1.2588 | 0.004456 | 0.131 |
| AC104590.1 | -5.15572 | -0.464 | 0.000538 | 0.03802 |
| AL392103.1 | -5.16134 | -1.94101 | 0.020796 | 0.283343 |
| LINC01210 | -5.16141 | 1.127719 | 0.000322 | 0.028333 |
| SLC22A25 | -5.16546 | 0.600706 | 0.000228 | 0.023223 |
| AC092979.1 | -5.16871 | -1.94157 | 0.010675 | 0.200703 |
| RN7SL471P | -5.17622 | -1.93582 | 0.046581 | 0.411952 |
| RNY1P6 | -5.17966 | -1.23478 | 0.007086 | 0.162963 |
| AC226118.1 | -5.19302 | -1.92207 | 0.010468 | 0.19906 |
| LINC00608 | -5.20151 | -1.92151 | 0.043642 | 0.399631 |
| MIR205HG | -5.20193 | 4.603945 | 6.15E-07 | 0.000466 |
| DMBT1 | -5.20797 | -1.92059 | 0.040897 | 0.38849 |
| VIP | -5.21538 | 2.527653 | 0.004503 | 0.131168 |
| CNTN5 | -5.21874 | 1.675704 | 0.000254 | 0.024647 |
| PCDH11Y | -5.2301 | 0.964247 | 0.008034 | 0.173474 |
| GPC5-IT1 | -5.23293 | -1.89445 | 0.032031 | 0.344575 |
| AC021534.1 | -5.23932 | -0.37587 | 0.002381 | 0.093027 |
| AC087277.1 | -5.24283 | -1.87496 | 0.018449 | 0.267127 |
| RRM2P3 | -5.24533 | -1.88231 | 0.009821 | 0.192944 |
| AC011726.1 | -5.24944 | -1.87426 | 0.012942 | 0.222602 |
| AC015908.1 | -5.2602 | -1.86213 | 0.014839 | 0.237736 |
| AC010967.1 | -5.26366 | -1.86571 | 0.017143 | 0.257478 |
| EEF1GP4 | -5.27416 | 1.733217 | 0.000113 | 0.015059 |
| LINC00525 | -5.29667 | -1.8394 | 0.022593 | 0.296149 |
| SERTM2 | -5.29902 | 4.532854 | 6.85E-07 | 0.000501 |
| AP001922.6 | -5.3125 | -1.82265 | 0.009991 | 0.194104 |
| AC079117.1 | -5.34858 | 1.467742 | 0.001724 | 0.074851 |
| CES1P1 | -5.35822 | -1.78802 | 0.036952 | 0.369765 |
| SOHLH2 | -5.35892 | 1.070388 | 0.00124 | 0.063185 |
| RN7SKP128 | -5.3605 | -0.61868 | 0.001185 | 0.061555 |
| SIM1 | -5.36593 | 0.627549 | 0.003837 | 0.12062 |
| AL096803.2 | -5.36688 | 0.804624 | 0.000763 | 0.047 |
| PABPC5-AS1 | -5.38021 | -1.76648 | 0.009291 | 0.187845 |
| SELENOKP3 | -5.38123 | -1.77091 | 0.010113 | 0.195038 |
| BX322635.1 | -5.38125 | -1.7756 | 0.006213 | 0.154647 |
| NPR3 | -5.38218 | 5.929855 | 2.83E-07 | 0.00025 |
| AC022733.1 | -5.39211 | 0.490026 | 0.007177 | 0.163776 |
| SOX2 | -5.39248 | 0.290676 | 3.96E-05 | 0.007555 |
| AL023775.1 | -5.39766 | -0.21919 | 0.000278 | 0.026292 |
| AC005077.2 | -5.41078 | 0.860602 | 7.11E-05 | 0.011305 |
| LINC02137 | -5.42345 | -1.72844 | 0.012095 | 0.215026 |
| RS1 | -5.42379 | -1.72691 | 0.013277 | 0.225242 |
| MTND3P12 | -5.42839 | -0.54546 | 0.001117 | 0.059656 |
| AP002856.4 | -5.42916 | -1.72631 | 0.010064 | 0.194673 |
| KC6 | -5.43566 | -1.72613 | 0.028311 | 0.327745 |
| RNU6-1318P | -5.43911 | -0.54071 | 0.000153 | 0.01779 |
| CYP27C1 | -5.44896 | -1.00768 | 0.000571 | 0.038961 |
| Z82206.1 | -5.49094 | -1.68383 | 0.004631 | 0.132896 |
| TNN | -5.49994 | 0.60541 | 0.003032 | 0.107337 |
| PURPL | -5.50563 | 1.879746 | 3.26E-05 | 0.006596 |
| FAM163A | -5.50818 | -1.67328 | 0.025426 | 0.311824 |
| CCDC175 | -5.52578 | 1.115475 | 0.000375 | 0.031017 |
| ZMAT4 | -5.52672 | 0.188282 | 0.000244 | 0.024154 |
| WT1 | -5.5275 | 1.217904 | 2.58E-05 | 0.005711 |
| PPP3R2 | -5.53597 | 1.118337 | 0.000748 | 0.04642 |
| MUC15 | -5.54547 | 0.625693 | 2.65E-05 | 0.005836 |
| CD1E | -5.55758 | 0.661549 | 0.000491 | 0.036269 |
| CACNA1G | -5.56721 | 1.713689 | 5.26E-05 | 0.008995 |
| GDI2P2 | -5.57035 | -1.62 | 0.005263 | 0.141733 |
| CXCL6 | -5.57184 | -1.60704 | 0.005062 | 0.138788 |
| LINC00958 | -5.58468 | -1.60677 | 0.004013 | 0.123444 |
| AL365226.2 | -5.58634 | 0.870225 | 0.000606 | 0.040438 |
| ADGRF1 | -5.58774 | 1.026676 | 5.87E-06 | 0.002016 |
| AC025431.1 | -5.59002 | 0.249013 | 0.001863 | 0.07854 |
| NEFM | -5.59285 | 0.490957 | 0.000532 | 0.037984 |
| HNRNPA1P40 | -5.60405 | -1.59116 | 0.00383 | 0.12062 |
| CHRM3-AS2 | -5.60431 | 2.335856 | 3.32E-08 | 4.22E-05 |
| OR51T1 | -5.62407 | -0.01991 | 0.00082 | 0.049008 |
| LINC02215 | -5.62997 | -1.56293 | 0.020867 | 0.28387 |
| GRP | -5.6483 | -1.55299 | 0.003041 | 0.107337 |
| FCRL1 | -5.65222 | -1.54259 | 0.020391 | 0.280054 |
| LRRC9 | -5.65749 | 6.049829 | 1.48E-06 | 0.000794 |
| AC015914.1 | -5.6809 | -1.52026 | 0.003062 | 0.107664 |
| VGLL3 | -5.69263 | 8.408044 | 4.80E-09 | 7.49E-06 |
| ERICH5 | -5.73994 | 2.198793 | 5.47E-06 | 0.001916 |
| SOSTDC1 | -5.7435 | -1.47554 | 0.016331 | 0.250427 |
| RPSAP32 | -5.75913 | -0.72165 | 0.001557 | 0.071896 |
| STAC2 | -5.76105 | 0.114028 | 3.66E-05 | 0.00715 |
| OLIG1 | -5.78747 | -1.41784 | 0.020668 | 0.282394 |
| LINC02234 | -5.79846 | -1.41465 | 0.013795 | 0.229786 |
| LINC01673 | -5.80064 | -1.41065 | 0.004587 | 0.132168 |
| AC011411.1 | -5.80536 | -1.41403 | 0.002688 | 0.10004 |
| RNU6-208P | -5.8187 | -2.94439 | 0.049749 | 0.424146 |
| CYP24A1 | -5.8595 | 0.21168 | 7.18E-05 | 0.011371 |
| B3GNTL1P1 | -5.86657 | -2.91243 | 0.049651 | 0.423993 |
| TNFSF11 | -5.88156 | -2.90368 | 0.047831 | 0.416976 |
| RNA5SP501 | -5.88513 | -2.90199 | 0.048412 | 0.419082 |
| LZTS1-AS1 | -5.90292 | -1.33076 | 0.001408 | 0.068037 |
| AC025423.5 | -5.91748 | -1.30838 | 0.003988 | 0.123088 |
| OR2D3 | -5.92487 | -2.87438 | 0.0485 | 0.419487 |
| AC022447.6 | -5.92487 | -2.87438 | 0.048489 | 0.419487 |
| TCEAL5 | -5.93203 | -0.5677 | 0.000153 | 0.01779 |
| FAM9B | -5.93703 | -2.87205 | 0.04912 | 0.421886 |
| MIR920 | -5.93781 | -2.87147 | 0.046498 | 0.411743 |
| AL021937.3 | -5.93815 | 2.783388 | 0.000117 | 0.015258 |
| AC005162.1 | -5.93852 | -2.86641 | 0.042532 | 0.39549 |
| SERPINA5 | -5.94078 | 0.291853 | 0.000511 | 0.037158 |
| AL450270.1 | -5.95764 | -2.85073 | 0.045835 | 0.40914 |
| AP000534.1 | -5.96674 | -0.53225 | 0.003921 | 0.121993 |
| ENPP3 | -5.97977 | 7.04096 | 3.17E-06 | 0.001344 |
| CHRNA9 | -5.98868 | -2.82809 | 0.049671 | 0.423993 |
| GPC5-AS1 | -5.98961 | 1.385075 | 5.98E-05 | 0.009832 |
| AC027031.2 | -6.01632 | -0.49309 | 8.81E-05 | 0.013162 |
| TMEM26-AS1 | -6.02026 | -0.48885 | 0.000154 | 0.01779 |
| LINC01507 | -6.02319 | 1.560528 | 0.000453 | 0.034469 |
| TGM3 | -6.02955 | 2.418212 | 1.18E-05 | 0.003179 |
| MIR4474 | -6.03963 | -2.79788 | 0.038859 | 0.378119 |
| CHL1 | -6.03972 | 6.027205 | 5.95E-14 | 6.81E-10 |
| RPL9P16 | -6.05049 | -1.19721 | 0.000717 | 0.045447 |
| MYOC | -6.06693 | 3.236646 | 3.32E-05 | 0.006607 |
| AL391097.2 | -6.06993 | -1.18046 | 0.000766 | 0.047 |
| AC090877.1 | -6.07017 | -2.77943 | 0.036679 | 0.368495 |
| LSM1P1 | -6.07952 | -2.77405 | 0.035417 | 0.362429 |
| UNGP1 | -6.08613 | -2.76592 | 0.035965 | 0.365401 |
| AP003117.2 | -6.10246 | -1.14896 | 0.00282 | 0.102833 |
| GABRA2 | -6.11462 | 3.321657 | 9.30E-06 | 0.002734 |
| AC006041.1 | -6.1235 | -1.13252 | 0.013624 | 0.22816 |
| AL132642.1 | -6.13817 | -2.73409 | 0.032968 | 0.349948 |
| AC006355.1 | -6.15237 | -2.72569 | 0.037155 | 0.370247 |
| IFNA21 | -6.15252 | -2.71356 | 0.048782 | 0.420162 |
| EDDM3A | -6.15658 | -1.10168 | 0.006647 | 0.158657 |
| SNORD113-6 | -6.15848 | -2.70995 | 0.046624 | 0.411986 |
| NAA11 | -6.16057 | 1.712655 | 5.46E-06 | 0.001916 |
| SERPINB4 | -6.16199 | -0.35549 | 0.000183 | 0.020175 |
| HNRNPA3P13 | -6.16372 | -2.70687 | 0.044534 | 0.403293 |
| AC092824.1 | -6.18969 | -2.69099 | 0.049412 | 0.422933 |
| OR51G2 | -6.19515 | 0.810214 | 0.001037 | 0.056926 |
| MS4A6E | -6.19615 | -2.68309 | 0.045961 | 0.409485 |
| ARHGAP23P1 | -6.2191 | -2.68417 | 0.043403 | 0.398729 |
| AL121656.1 | -6.2212 | -2.67121 | 0.034631 | 0.359025 |
| RNU6-512P | -6.22186 | -2.67119 | 0.046973 | 0.41299 |
| AC087762.1 | -6.22516 | -2.66496 | 0.0435 | 0.399117 |
| AC026780.1 | -6.23465 | -2.66314 | 0.028871 | 0.330562 |
| AC021733.1 | -6.24275 | -2.65442 | 0.045926 | 0.409423 |
| AC002069.2 | -6.25135 | -2.64896 | 0.030994 | 0.340778 |
| AP005120.1 | -6.26149 | -2.64611 | 0.027075 | 0.321914 |
| AP001599.1 | -6.27531 | -2.63406 | 0.028438 | 0.328042 |
| AC068389.1 | -6.29003 | -2.62821 | 0.029794 | 0.334338 |
| ZSWIM5P2 | -6.30008 | -2.6114 | 0.034862 | 0.359131 |
| CNGB1 | -6.30402 | -2.61239 | 0.041743 | 0.391688 |
| AC017074.2 | -6.31481 | -2.60558 | 0.031435 | 0.342118 |
| AC104779.1 | -6.31934 | 0.649101 | 1.00E-05 | 0.00289 |
| AL139095.3 | -6.32332 | -2.59305 | 0.04596 | 0.409485 |
| FCRL3 | -6.3252 | 0.281228 | 9.23E-06 | 0.002734 |
| SERPINB3 | -6.32798 | 0.293392 | 0.001433 | 0.068476 |
| SST | -6.33124 | -2.59494 | 0.023998 | 0.303555 |
| MIR5586 | -6.3336 | -2.59013 | 0.040031 | 0.384928 |
| AL109930.1 | -6.33973 | -2.5863 | 0.028387 | 0.327753 |
| LINC02148 | -6.33973 | -2.5863 | 0.028382 | 0.327753 |
| AC026333.3 | -6.34426 | -2.59004 | 0.030002 | 0.335707 |
| OVCH1-AS1 | -6.34539 | -2.58909 | 0.024959 | 0.308824 |
| AC037487.3 | -6.35528 | -2.57626 | 0.0246 | 0.306453 |
| RPL12P7 | -6.36355 | -2.571 | 0.029096 | 0.331257 |
| DSG3 | -6.36418 | -0.9258 | 0.004421 | 0.130544 |
| RN7SKP99 | -6.38457 | -2.55053 | 0.035836 | 0.364665 |
| VAX1 | -6.38764 | -2.55852 | 0.026126 | 0.315499 |
| AC004839.1 | -6.39145 | -2.55298 | 0.036226 | 0.366191 |
| OR7E115P | -6.39888 | -0.88711 | 0.004044 | 0.123695 |
| AC022816.1 | -6.40554 | -2.53741 | 0.031903 | 0.343949 |
| CACNA2D3-AS1 | -6.42161 | -2.53245 | 0.027918 | 0.326699 |
| INMT-MINDY4 | -6.4318 | -2.52248 | 0.03864 | 0.376949 |
| AC119751.4 | -6.44495 | -2.51156 | 0.020091 | 0.277479 |
| AP006295.1 | -6.45218 | -2.50694 | 0.023411 | 0.30089 |
| AC007424.1 | -6.45567 | -0.83544 | 0.000244 | 0.024154 |
| AC137770.1 | -6.45947 | -2.51121 | 0.036671 | 0.368495 |
| GPX5 | -6.46062 | -0.82786 | 0.00347 | 0.114733 |
| OLFM3 | -6.46151 | -2.49479 | 0.025193 | 0.310391 |
| IGFL2-AS1 | -6.46177 | -2.50054 | 0.020113 | 0.277565 |
| MTND2P23 | -6.46526 | -2.49536 | 0.024764 | 0.307414 |
| OR51H2P | -6.46872 | -0.07699 | 0.001343 | 0.06622 |
| FGF14-IT1 | -6.46938 | -2.49263 | 0.027328 | 0.323021 |
| COX5BP2 | -6.4736 | -0.81828 | 0.000426 | 0.033339 |
| GAP43 | -6.47929 | 1.876037 | 1.17E-05 | 0.003159 |
| ALDH3A1 | -6.47957 | -2.4887 | 0.019879 | 0.276107 |
| AC087672.2 | -6.48733 | -2.48369 | 0.026615 | 0.318979 |
| SLC14A2-AS1 | -6.48749 | -0.80492 | 0.000203 | 0.021602 |
| OR51L1 | -6.48768 | -2.47763 | 0.023438 | 0.300964 |
| KRT18P60 | -6.4982 | -2.46733 | 0.031072 | 0.340878 |
| OLFM4 | -6.50697 | 4.437374 | 1.33E-07 | 0.000138 |
| AC007091.1 | -6.52478 | -0.77095 | 0.000439 | 0.034052 |
| AC111194.2 | -6.53619 | -2.4425 | 0.018748 | 0.268765 |
| BANK1 | -6.5388 | 7.371087 | 2.09E-09 | 4.17E-06 |
| SLC17A3 | -6.54026 | -2.44514 | 0.018493 | 0.267127 |
| PGBD4P8 | -6.54912 | 1.768362 | 2.71E-05 | 0.005878 |
| RNU6-1108P | -6.55899 | -2.42155 | 0.032263 | 0.346342 |
| TGM4 | -6.56423 | 6.098866 | 2.76E-06 | 0.001294 |
| MIR3164 | -6.56949 | -2.41468 | 0.02121 | 0.286161 |
| RNU6ATAC18P | -6.57692 | -2.41242 | 0.021126 | 0.285361 |
| AL590103.1 | -6.5773 | -2.40949 | 0.020685 | 0.282394 |
| OR2AG2 | -6.57997 | -2.40483 | 0.029275 | 0.332315 |
| VAT1L | -6.5863 | 1.187109 | 1.40E-05 | 0.003578 |
| AC093084.1 | -6.5893 | -2.40964 | 0.048654 | 0.419715 |
| AC005410.1 | -6.59202 | -2.3967 | 0.030604 | 0.338734 |
| AC010457.1 | -6.59643 | -2.39089 | 0.032805 | 0.348964 |
| AL133244.1 | -6.61103 | -2.39715 | 0.047508 | 0.415773 |
| OR51A4 | -6.61526 | 1.215278 | 4.36E-05 | 0.00809 |
| LINC01139 | -6.61595 | -0.69335 | 0.000131 | 0.016077 |
| AC009229.3 | -6.61687 | -2.39003 | 0.048571 | 0.419675 |
| AC111198.1 | -6.61999 | -2.37759 | 0.048371 | 0.419059 |
| AC019068.1 | -6.6217 | -2.38479 | 0.047254 | 0.414589 |
| MIR548I2 | -6.63005 | -2.37367 | 0.016889 | 0.255413 |
| CACNA1C-AS2 | -6.63976 | -2.36629 | 0.021102 | 0.285351 |
| MTND6P17 | -6.64156 | -2.35789 | 0.027616 | 0.32475 |
| CHL1-AS1 | -6.64675 | -0.65997 | 0.000125 | 0.015724 |
| HEPACAM | -6.64791 | -2.35367 | 0.029939 | 0.335631 |
| AL441964.1 | -6.64802 | -2.36111 | 0.012975 | 0.222952 |
| LINC02587 | -6.65097 | -2.36693 | 0.044744 | 0.404116 |
| RNU6ATAC10P | -6.65495 | -2.35877 | 0.014032 | 0.231059 |
| BEX1 | -6.66588 | -0.64325 | 7.53E-05 | 0.011761 |
| AC007068.2 | -6.67542 | -2.33729 | 0.04336 | 0.398699 |
| HOXB8 | -6.68353 | 0.126824 | 6.01E-05 | 0.009836 |
| AC104072.1 | -6.68488 | -2.33337 | 0.015569 | 0.243676 |
| LINC01388 | -6.68557 | 0.135513 | 3.89E-06 | 0.001549 |
| MIR548N | -6.68666 | -2.32212 | 0.031519 | 0.342118 |
| AC002069.1 | -6.69046 | -2.3244 | 0.043687 | 0.399631 |
| CRABP1 | -6.6924 | -2.32581 | 0.015744 | 0.245151 |
| LINC02185 | -6.69345 | -2.32503 | 0.013099 | 0.223646 |
| AC004692.2 | -6.69897 | -2.32812 | 0.042845 | 0.397109 |
| ARHGEF7-IT1 | -6.70092 | -2.31754 | 0.01398 | 0.231059 |
| WNT6 | -6.70303 | -2.31841 | 0.023256 | 0.300391 |
| CFTR-AS1 | -6.70446 | -2.32147 | 0.046548 | 0.411763 |
| AC098649.1 | -6.7065 | 0.157788 | 0.000892 | 0.051436 |
| AL157896.1 | -6.70806 | -2.319 | 0.04531 | 0.406782 |
| GCG | -6.71105 | 1.547364 | 1.68E-06 | 0.00088 |
| AC105031.3 | -6.71882 | -2.3116 | 0.042768 | 0.396824 |
| AC092994.1 | -6.72001 | -2.29904 | 0.044448 | 0.402848 |
| BX293995.1 | -6.72065 | -2.30114 | 0.041609 | 0.390821 |
| AC099499.1 | -6.72239 | -2.30914 | 0.042156 | 0.393308 |
| AC104389.1 | -6.72763 | -2.29382 | 0.043322 | 0.398699 |
| AL034351.1 | -6.72951 | -2.29306 | 0.017574 | 0.260309 |
| AL121820.2 | -6.7311 | -2.29647 | 0.012078 | 0.215011 |
| AC105053.1 | -6.73304 | -2.30178 | 0.040616 | 0.387322 |
| SNORD116-10 | -6.73673 | -2.29006 | 0.013851 | 0.230285 |
| AC091860.2 | -6.73839 | -2.2953 | 0.045638 | 0.408444 |
| AL355922.4 | -6.74229 | -2.27862 | 0.047148 | 0.414087 |
| KLK7 | -6.75147 | -2.27549 | 0.0177 | 0.261262 |
| AC012519.1 | -6.75631 | -2.26909 | 0.046722 | 0.412346 |
| CDH22 | -6.763 | -2.28382 | 0.042843 | 0.397109 |
| KCNK2 | -6.76432 | 1.354136 | 2.39E-08 | 3.16E-05 |
| AP000755.2 | -6.76633 | -2.27592 | 0.038771 | 0.377485 |
| RPF2P2 | -6.77243 | -2.26282 | 0.038395 | 0.376267 |
| LINC02532 | -6.77667 | -2.2687 | 0.03701 | 0.369765 |
| RNY4P36 | -6.78596 | -2.25348 | 0.010674 | 0.200703 |
| AC114752.1 | -6.78768 | -2.24798 | 0.016095 | 0.248247 |
| KRT8P49 | -6.79578 | -2.24447 | 0.01229 | 0.216376 |
| AC073359.1 | -6.80209 | -2.23788 | 0.014723 | 0.236766 |
| RNU6-729P | -6.80245 | -2.2534 | 0.042011 | 0.392769 |
| RNU6-815P | -6.80394 | -2.24435 | 0.041943 | 0.392671 |
| SUMO2P15 | -6.80733 | -2.2472 | 0.036988 | 0.369765 |
| CDH19 | -6.81448 | 4.13943 | 1.26E-08 | 1.88E-05 |
| AC107926.1 | -6.81569 | -2.23868 | 0.034922 | 0.359351 |
| SGO1P1 | -6.82027 | 0.26048 | 3.23E-05 | 0.006596 |
| OR52S1P | -6.83543 | -2.21176 | 0.035925 | 0.365223 |
| AC009271.1 | -6.85041 | -2.21158 | 0.033127 | 0.350841 |
| LINC01896 | -6.85869 | -2.20825 | 0.035704 | 0.363809 |
| AP003043.1 | -6.85927 | -2.20592 | 0.037049 | 0.369936 |
| LINC02422 | -6.86013 | -2.19446 | 0.009507 | 0.190643 |
| AL354754.1 | -6.86205 | -2.19269 | 0.013728 | 0.229127 |
| ISL2 | -6.86246 | -2.19289 | 0.009579 | 0.191135 |
| AC073587.1 | -6.87524 | -2.17707 | 0.038629 | 0.376949 |
| AC027419.2 | -6.8839 | -2.17736 | 0.031093 | 0.340931 |
| SALL3 | -6.88506 | 0.321693 | 5.20E-07 | 0.000415 |
| AC026780.2 | -6.8859 | -2.18146 | 0.032763 | 0.348743 |
| RN7SKP283 | -6.88922 | -2.18393 | 0.033416 | 0.352191 |
| PRSS1 | -6.88978 | -2.16504 | 0.022242 | 0.294071 |
| GATA3-AS1 | -6.89214 | -2.16771 | 0.010319 | 0.198124 |
| OR51I2 | -6.89435 | -2.16414 | 0.013314 | 0.22539 |
| AC016550.3 | -6.897 | -2.17118 | 0.036423 | 0.367154 |
| AC126182.2 | -6.90308 | -2.15947 | 0.03169 | 0.343162 |
| AC105031.2 | -6.90805 | -2.1656 | 0.030696 | 0.33925 |
| AL356473.1 | -6.91589 | -2.14824 | 0.031341 | 0.341542 |
| RNU6-1222P | -6.92122 | -2.14025 | 0.037268 | 0.370677 |
| AL355601.1 | -6.92151 | -2.14467 | 0.009826 | 0.192944 |
| KRT8P36 | -6.92198 | -2.14203 | 0.016169 | 0.248834 |
| AC010546.1 | -6.92693 | -2.13394 | 0.047686 | 0.416359 |
| GPR3 | -6.93896 | -2.13197 | 0.008103 | 0.17417 |
| TMPOP1 | -6.94286 | -2.13086 | 0.027986 | 0.326784 |
| GPR15 | -6.94365 | -2.13319 | 0.041273 | 0.38947 |
| RPL5P16 | -6.95086 | -2.12653 | 0.006569 | 0.158016 |
| AC022690.2 | -6.95102 | -2.13091 | 0.029372 | 0.332562 |
| AC066616.2 | -6.95102 | 2.493908 | 1.89E-06 | 0.000954 |
| RN7SKP57 | -6.95715 | -2.11508 | 0.010422 | 0.198777 |
| NPAS4 | -6.96123 | -2.11192 | 0.011933 | 0.213475 |
| RPL21P106 | -6.96709 | -2.10341 | 0.033056 | 0.350557 |
| AC002486.1 | -6.97099 | -2.11346 | 0.029706 | 0.333985 |
| SLC15A1 | -6.97272 | -0.36031 | 8.62E-06 | 0.002667 |
| AC092506.1 | -6.97405 | -2.11111 | 0.008098 | 0.17417 |
| FCRLA | -6.97853 | -2.11014 | 0.026764 | 0.319846 |
| LINC02277 | -6.97935 | -2.10793 | 0.015296 | 0.241561 |
| AC010343.3 | -6.98427 | -2.0891 | 0.039224 | 0.380593 |
| MIR31HG | -6.98639 | -2.09688 | 0.006864 | 0.160585 |
| GVINP2 | -7.0115 | -2.07519 | 0.025711 | 0.313156 |
| HMGB3P18 | -7.01654 | -2.06397 | 0.030677 | 0.339144 |
| LINC01378 | -7.01772 | -2.07063 | 0.025481 | 0.312028 |
| AP002075.1 | -7.02606 | 2.42128 | 1.83E-06 | 0.000939 |
| AL596218.1 | -7.02712 | -2.07247 | 0.025858 | 0.313958 |
| AC010980.2 | -7.02863 | -2.06268 | 0.006776 | 0.159859 |
| CRISP2 | -7.04389 | -2.04458 | 0.015685 | 0.244769 |
| UGT2B7 | -7.05033 | -0.28426 | 0.000259 | 0.024875 |
| AC022726.1 | -7.05192 | -2.04821 | 0.005748 | 0.148332 |
| GABRG2 | -7.05841 | -2.03362 | 0.026194 | 0.315552 |
| CEACAMP2 | -7.06633 | -2.02989 | 0.008024 | 0.173474 |
| AC010425.1 | -7.06918 | -2.02213 | 0.030289 | 0.337353 |
| CELF2-AS2 | -7.07357 | -2.02289 | 0.006858 | 0.160585 |
| AL049649.1 | -7.07587 | -2.01905 | 0.025746 | 0.313156 |
| PNLIPRP3 | -7.08121 | -2.01336 | 0.027776 | 0.325729 |
| MKX-AS1 | -7.08382 | -2.02651 | 0.023248 | 0.300391 |
| AC068993.1 | -7.08786 | -2.00709 | 0.016942 | 0.25569 |
| CRAT37 | -7.09016 | -2.01341 | 0.004716 | 0.133888 |
| TYR | -7.10351 | -2.0005 | 0.011797 | 0.212405 |
| LINC02253 | -7.10434 | -1.99284 | 0.029319 | 0.33243 |
| RNU6-71P | -7.1049 | -1.99595 | 0.024275 | 0.305047 |
| AL445647.1 | -7.10514 | 1.412374 | 6.99E-05 | 0.011274 |
| AC016885.3 | -7.11075 | -1.99162 | 0.023694 | 0.301883 |
| RN7SL292P | -7.11236 | -1.99539 | 0.021579 | 0.288859 |
| LHFPL1 | -7.11473 | -1.99355 | 0.004649 | 0.133171 |
| RNU6-809P | -7.11798 | -1.98516 | 0.008752 | 0.183253 |
| LINC02027 | -7.12025 | -1.97933 | 0.036932 | 0.369765 |
| OR52A1 | -7.12292 | -1.98423 | 0.021408 | 0.287539 |
| MARCOL | -7.12343 | -1.99131 | 0.021617 | 0.289184 |
| MIR3126 | -7.13567 | -1.98041 | 0.004711 | 0.133844 |
| LINC02518 | -7.152 | -1.96658 | 0.004366 | 0.129289 |
| LINC01187 | -7.16002 | -1.95364 | 0.007477 | 0.167397 |
| AC005077.1 | -7.16076 | -1.95978 | 0.021661 | 0.289512 |
| PNMA5 | -7.16088 | -1.95305 | 0.005539 | 0.145325 |
| WNT7A | -7.17193 | -1.94174 | 0.007009 | 0.162022 |
| AC119150.1 | -7.17446 | -1.94082 | 0.009662 | 0.191514 |
| MTCO1P51 | -7.17456 | -1.94701 | 0.01959 | 0.274206 |
| AC010207.1 | -7.1758 | -1.94016 | 0.010623 | 0.200046 |
| SLC22A2 | -7.18038 | -1.93961 | 0.004964 | 0.137306 |
| AC005009.1 | -7.18101 | -1.93343 | 0.00916 | 0.187395 |
| STK32A-AS1 | -7.18635 | -1.9337 | 0.004229 | 0.126435 |
| AC004945.1 | -7.18642 | -1.93861 | 0.006441 | 0.156814 |
| RN7SKP112 | -7.18644 | -1.93068 | 0.010866 | 0.20274 |
| AC016550.2 | -7.19075 | -1.93548 | 0.020077 | 0.277479 |
| AC063947.1 | -7.19322 | -1.92846 | 0.006515 | 0.157255 |
| MIR4300 | -7.19571 | -1.92794 | 0.018467 | 0.267127 |
| LINC00945 | -7.19853 | -1.91826 | 0.022527 | 0.296001 |
| RN7SKP184 | -7.20313 | -1.92343 | 0.003847 | 0.12076 |
| SPATA8 | -7.20336 | -1.91306 | 0.024734 | 0.307376 |
| AC113331.1 | -7.21007 | -1.91111 | 0.019315 | 0.272467 |
| AL162408.1 | -7.2127 | -1.92054 | 0.018107 | 0.264226 |
| MTND2P9 | -7.21551 | -1.90701 | 0.019017 | 0.270223 |
| RNU4-31P | -7.22013 | -1.90396 | 0.005084 | 0.138928 |
| AC021127.1 | -7.22403 | -1.91025 | 0.017726 | 0.261416 |
| AC092573.1 | -7.23277 | -1.90197 | 0.017489 | 0.259609 |
| WWC2-AS2 | -7.24167 | -1.89682 | 0.017413 | 0.259234 |
| KRT73 | -7.24668 | -1.88052 | 0.018668 | 0.268524 |
| SAA2 | -7.24779 | -1.89051 | 0.017019 | 0.25639 |
| AC121154.1 | -7.25143 | -1.88614 | 0.017429 | 0.259234 |
| AC073522.1 | -7.25352 | -1.87955 | 0.016405 | 0.250772 |
| OR5P2 | -7.26594 | -1.87239 | 0.003752 | 0.119322 |
| C4BPA | -7.26786 | -1.8685 | 0.003766 | 0.1194 |
| AC104088.1 | -7.27443 | -1.85713 | 0.0059 | 0.150552 |
| AF123462.1 | -7.27647 | -1.85934 | 0.008384 | 0.177868 |
| Z82196.2 | -7.28179 | -1.84962 | 0.019501 | 0.273851 |
| HOXB-AS3 | -7.29833 | -1.84695 | 0.003339 | 0.112578 |
| RNU6-549P | -7.31045 | -1.82498 | 0.017938 | 0.263043 |
| MORF4L1P5 | -7.31391 | -1.83479 | 0.003811 | 0.120418 |
| KLRK1 | -7.32535 | -1.82229 | 0.002462 | 0.095025 |
| AC007656.1 | -7.33103 | -1.81465 | 0.002662 | 0.099612 |
| AL731892.1 | -7.33629 | -1.8029 | 0.013622 | 0.22816 |
| RNU6-1194P | -7.35057 | -1.79172 | 0.018975 | 0.270012 |
| ST8SIA2 | -7.35274 | -1.79521 | 0.014016 | 0.231059 |
| SLC6A2 | -7.35479 | -1.79388 | 0.002823 | 0.102833 |
| MIOXP1 | -7.35792 | -1.78912 | 0.004726 | 0.133949 |
| MTCYBP23 | -7.3588 | 1.954323 | 1.05E-06 | 0.000643 |
| AP001604.1 | -7.36307 | -1.78843 | 0.01359 | 0.227825 |
| SPHKAP | -7.37506 | -1.78088 | 0.002157 | 0.087422 |
| SLC22A6 | -7.3791 | -1.77247 | 0.007014 | 0.162026 |
| AMTN | -7.38374 | -1.77219 | 0.002676 | 0.099924 |
| TM4SF4 | -7.38824 | -1.77177 | 0.013547 | 0.227437 |
| AC004543.1 | -7.39393 | -1.76202 | 0.006525 | 0.157274 |
| AL031283.3 | -7.395 | -1.76648 | 0.01317 | 0.224394 |
| AC007346.1 | -7.41215 | -1.74548 | 0.006462 | 0.156814 |
| AL772284.1 | -7.41881 | -1.74576 | 0.006612 | 0.158657 |
| MIR4675 | -7.42095 | -1.73247 | 0.017991 | 0.263417 |
| NKAIN3 | -7.42585 | -1.73362 | 0.006304 | 0.155437 |
| ARNTL2-AS1 | -7.43189 | -1.72887 | 0.002187 | 0.088048 |
| RPL32P35 | -7.43813 | -1.71832 | 0.009051 | 0.185944 |
| AC093281.1 | -7.44664 | -1.72209 | 0.012363 | 0.216941 |
| ABCC12 | -7.45887 | -1.70415 | 0.012496 | 0.218 |
| HBBP1 | -7.46104 | -1.69765 | 0.023566 | 0.301142 |
| HNRNPA1P71 | -7.46118 | -1.7015 | 0.006836 | 0.160585 |
| SLC30A3 | -7.46372 | -1.6986 | 0.00483 | 0.135652 |
| LINC00840 | -7.47135 | -1.68977 | 0.015426 | 0.242761 |
| OR6A2 | -7.47442 | -1.68906 | 0.005538 | 0.145325 |
| S100A2 | -7.47486 | -1.69377 | 0.001596 | 0.072666 |
| AC091826.1 | -7.47663 | -1.68434 | 0.023339 | 0.300474 |
| RNU105B | -7.49401 | -1.6767 | 0.002722 | 0.100592 |
| SNRPGP1 | -7.50663 | -1.66931 | 0.001836 | 0.078255 |
| FOXD3 | -7.52606 | -1.64508 | 0.007093 | 0.162963 |
| EQTN | -7.53293 | -1.63977 | 0.005157 | 0.139887 |
| OR51F3P | -7.53597 | 0.167804 | 3.19E-05 | 0.006553 |
| AC026523.1 | -7.54051 | -1.63488 | 0.002965 | 0.106101 |
| OR52A5 | -7.55815 | -1.61939 | 0.010404 | 0.198764 |
| RNU1-63P | -7.57098 | -1.60917 | 0.009665 | 0.191514 |
| BGLT3 | -7.57098 | -1.60917 | 0.009657 | 0.191514 |
| AC004805.3 | -7.58171 | -1.60102 | 0.001584 | 0.072427 |
| AC008948.1 | -7.58323 | -1.59842 | 0.009641 | 0.191514 |
| LINC01241 | -7.59908 | -1.58384 | 0.00992 | 0.193796 |
| BNIP3P41 | -7.60275 | -1.57993 | 0.010559 | 0.199443 |
| AC106786.1 | -7.60561 | -1.57915 | 0.00198 | 0.082451 |
| AC027104.1 | -7.61672 | -1.57424 | 0.001499 | 0.070643 |
| AC079380.1 | -7.62236 | -1.56798 | 0.000925 | 0.052282 |
| LINC02404 | -7.63299 | -1.55844 | 0.007845 | 0.171764 |
| DCAF12L1 | -7.63499 | -1.5615 | 0.001214 | 0.062432 |
| UBE2FP2 | -7.63615 | 1.931275 | 8.00E-06 | 0.002546 |
| RNU4-18P | -7.63934 | -1.54971 | 0.008865 | 0.183729 |
| AL356133.2 | -7.65995 | -1.53398 | 0.007715 | 0.170532 |
| AC002504.1 | -7.67299 | -1.52696 | 0.009981 | 0.194025 |
| AL391704.1 | -7.71407 | -1.4883 | 0.006683 | 0.158983 |
| AC008619.1 | -7.73914 | -1.46894 | 0.000573 | 0.039062 |
| AL136131.2 | -7.7457 | -1.45965 | 0.000862 | 0.050426 |
| LINC02315 | -7.7518 | -1.45156 | 0.002532 | 0.09622 |
| AC121342.1 | -7.75554 | 0.383123 | 4.61E-06 | 0.001734 |
| ATP5F1AP4 | -7.76414 | -1.44181 | 0.006965 | 0.161548 |
| MEIS1-AS3 | -7.77235 | -1.44198 | 0.00072 | 0.045538 |
| AC099788.1 | -7.77868 | -1.42883 | 0.001731 | 0.074991 |
| NAT2 | -7.79324 | -1.41721 | 0.006276 | 0.155234 |
| AL389895.1 | -7.80546 | -1.4033 | 0.009652 | 0.191514 |
| AL035555.1 | -7.81673 | -1.39733 | 0.002713 | 0.100592 |
| AC106883.1 | -7.819 | -1.39672 | 0.005357 | 0.143076 |
| OR51E2 | -7.82557 | 7.960149 | 3.78E-12 | 2.16E-08 |
| CHP2 | -7.8485 | -1.37122 | 0.001343 | 0.06622 |
| AC087518.1 | -7.86371 | -1.35754 | 0.004977 | 0.137306 |
| AC040174.1 | -7.86728 | 2.89528 | 8.34E-08 | 9.55E-05 |
| AL355578.1 | -7.89888 | -1.32587 | 0.000558 | 0.038403 |
| AC022690.1 | -7.90709 | -1.31941 | 0.004506 | 0.131168 |
| LINC01758 | -7.91537 | -1.31196 | 0.002035 | 0.083736 |
| PSMC1P7 | -7.91546 | 2.179983 | 9.37E-05 | 0.013603 |
| SERTM1 | -7.93038 | -1.30603 | 0.004677 | 0.133341 |
| NBPF22P | -7.93097 | -1.30479 | 0.004342 | 0.128789 |
| LINC01435 | -7.93918 | -1.29043 | 0.000519 | 0.037417 |
| AC006305.1 | -7.93967 | -1.29164 | 0.001069 | 0.058305 |
| AC087430.1 | -7.97078 | -1.26621 | 0.000541 | 0.03802 |
| NEUROD4 | -7.97571 | -1.25574 | 0.004807 | 0.135343 |
| AP001880.2 | -7.98334 | -1.25116 | 0.003967 | 0.122543 |
| AC138305.2 | -7.98625 | 0.606053 | 1.36E-05 | 0.003502 |
| TPTE2P2 | -7.99984 | -1.23423 | 0.001196 | 0.061986 |
| CNTN6 | -8.00423 | 0.617482 | 9.10E-07 | 0.000613 |
| NCAPGP1 | -8.00975 | -1.23197 | 0.000542 | 0.03802 |
| AL357833.1 | -8.01354 | -1.22214 | 0.001172 | 0.061232 |
| LTF | -8.04065 | 6.319878 | 2.92E-22 | 1.00E-17 |
| LINC02120 | -8.07843 | -1.1647 | 0.003835 | 0.12062 |
| OR5P4P | -8.0795 | -1.16626 | 0.000226 | 0.023223 |
| AC090825.1 | -8.08384 | -1.16484 | 0.000182 | 0.020136 |
| NPAP1 | -8.12494 | -1.12461 | 0.000402 | 0.032293 |
| HSPA8P6 | -8.13487 | -1.11776 | 0.000247 | 0.024307 |
| AC114878.2 | -8.14156 | -1.10995 | 0.000248 | 0.024307 |
| LINC02211 | -8.15895 | -1.09987 | 0.002517 | 0.095964 |
| ANKRD1 | -8.16982 | -1.08895 | 0.002819 | 0.102833 |
| CLDN16 | -8.18522 | -1.07109 | 0.000224 | 0.023148 |
| GPR20 | -8.19576 | -1.06188 | 0.000162 | 0.018395 |
| AP000793.1 | -8.2176 | -1.04057 | 0.002599 | 0.098198 |
| GOLGA2P11 | -8.22969 | -1.0358 | 0.002147 | 0.087188 |
| FTLP17 | -8.23243 | -1.02692 | 0.000581 | 0.039436 |
| PACERR | -8.23692 | -1.02738 | 7.97E-05 | 0.012166 |
| AC063955.1 | -8.25398 | -1.01086 | 0.001998 | 0.0828 |
| LINC01358 | -8.30583 | -0.96283 | 0.000216 | 0.022717 |
| RPP40P1 | -8.31672 | -0.95292 | 0.000317 | 0.028089 |
| AC106754.1 | -8.33043 | -0.94457 | 0.001672 | 0.074024 |
| NPM1P8 | -8.33176 | -0.94061 | 4.70E-05 | 0.008417 |
| ENAM | -8.33207 | -0.94272 | 0.001723 | 0.074851 |
| PAEP | -8.39307 | -0.88307 | 0.001503 | 0.070719 |
| SLC13A2 | -8.42146 | -0.858 | 3.82E-05 | 0.007411 |
| AC027419.1 | -8.45824 | -0.82215 | 0.001507 | 0.070719 |
| UCN3 | -8.48194 | -0.80558 | 0.001212 | 0.062432 |
| AC119751.3 | -8.50448 | -0.78317 | 0.000429 | 0.033399 |
| SPINK2 | -8.52159 | -0.76604 | 0.000281 | 0.026343 |
| AL049555.1 | -8.54018 | -0.75037 | 1.88E-05 | 0.004475 |
| LINC00397 | -8.58259 | -0.70807 | 0.001132 | 0.06012 |
| ST13P20 | -8.59614 | -0.69658 | 0.000159 | 0.01815 |
| LINC01698 | -8.6298 | -0.66525 | 0.000903 | 0.051873 |
| AC092167.1 | -8.6521 | -0.64682 | 0.000104 | 0.014597 |
| IGLV1-44 | -8.65979 | -0.64024 | 1.10E-05 | 0.003092 |
| LINC02475 | -8.66803 | -0.63055 | 0.000117 | 0.015258 |
| RPL9P15 | -8.78971 | -0.51553 | 0.000924 | 0.052282 |
| SPRR2G | -8.80627 | -0.5057 | 0.000503 | 0.036842 |
| AC126177.2 | -8.86169 | -0.45026 | 3.65E-05 | 0.00715 |
| AC026888.1 | -8.88616 | -0.42817 | 7.60E-06 | 0.002489 |
| RNU6-679P | -8.89228 | -0.41966 | 0.000866 | 0.050512 |
| GLP1R | -8.90163 | 2.293591 | 3.50E-07 | 0.0003 |
| LINC01194 | -8.95873 | -0.3588 | 0.000441 | 0.034109 |
| AC078842.2 | -8.96805 | -0.35232 | 4.52E-06 | 0.001734 |
| HAUS1P2 | -8.98954 | -0.33178 | 0.000323 | 0.028333 |
| RHOXF2B | -9.01355 | -0.31095 | 0.000125 | 0.015724 |
| SLC5A7 | -9.04882 | -0.27583 | 1.26E-06 | 0.000735 |
| LINC01105 | -9.05258 | -0.27193 | 0.000279 | 0.026292 |
| CRISP1 | -9.07223 | -0.25172 | 0.000107 | 0.014765 |
| AL133299.1 | -9.08172 | -0.24379 | 0.000105 | 0.014714 |
| SLC28A3 | -9.17283 | -0.15871 | 2.80E-06 | 0.001294 |
| RFPL3 | -9.18642 | -0.14587 | 0.000196 | 0.021174 |
| NPAP1P4 | -9.23557 | -0.09805 | 7.69E-05 | 0.011902 |
| MTCO1P14 | -9.29422 | -0.04321 | 7.02E-06 | 0.002386 |
| SEMG2 | -9.39806 | 5.169283 | 2.65E-09 | 4.78E-06 |
| GFRA3 | -9.55867 | 0.20929 | 7.42E-07 | 0.000531 |
| MTCO3P23 | -9.5908 | 0.239417 | 7.60E-05 | 0.011807 |
| KCTD8 | -9.67914 | 0.325349 | 1.32E-06 | 0.000742 |
| OR51C1P | -9.72189 | 5.694197 | 1.19E-11 | 4.09E-08 |
| MTND5P32 | -9.74618 | 2.328881 | 1.66E-08 | 2.29E-05 |
| CCDC60 | -9.94522 | 0.580783 | 4.19E-09 | 6.85E-06 |
| AL590032.1 | -10.0793 | 0.711764 | 2.87E-05 | 0.006046 |
| LINC01031 | -10.0877 | 0.720112 | 9.61E-06 | 0.002798 |
| AC040174.2 | -10.1605 | 0.790352 | 6.24E-07 | 0.000466 |
| SLC34A2 | -10.2015 | 0.829743 | 6.94E-10 | 1.66E-06 |
| SEMG1 | -10.2198 | 7.552925 | 1.73E-10 | 4.84E-07 |
| RNU4-69P | -10.4259 | 1.049303 | 1.69E-06 | 0.00088 |
| LINC02067 | -10.8825 | 3.456869 | 7.46E-10 | 1.66E-06 |
| MTND5P5 | -12.781 | 3.37648 | 9.19E-12 | 3.95E-08 |

Table6 Transcription Factor Prediction Results

GENECARD: HCFC1 SP1 POLR2A DPF2 ZNF580 ATF2 JUND KLF17 NCOR1 ZNF184 ATF3 ZNF600 ZIC2 KLF9 ZNF10 BRCA1 YY1 HIC1 ZBTB10 REST ZNF207 CEBPB MAZ MBD2 ZNF592 MNT SIN3A TCF12 TBP ARNT ZNF217 ZNF639 ZNF341 MAX ZEB1 ZNF223 KLF7 MXD4 SMAD5 KMT2B KLF11 TAF9B ETS1 SAP130 ZNF610 HOMEZ CTBP1 ZFP64 MAFF SP2 MIER3 THAP11 NKRF SMARCA5 ZSCAN9 SIN3B ZNF316 NBN RXRA SKIL CBFB TCF7L2 ZBTB33 USF2 MAFK DMAP1 ZFP91 ZSCAN5A CBX1 ZNF843 ATF1 CREM HLTF SUPT5H ZNF205 KDM5A ZNF146 ZBTB48 ZBTB40 KLF16 BCL11B HDAC2 ZNF318 ZZZ3 MYC TAF1 ZSCAN23 PRDM10 ELF1 MLLT1 EP400 FOXK2 BHLHE40 ZNF189 ZNF24 FOXM1 SOX5 ZNF2 NRF1 NCOA1 MAFG ZC3H8 INSM2 RAD51 IKZF1 ZFP37 RB1 DIDO1 HNF4A EGR1 RFX5 GATAD1 ZNF547 HBP1 MXD3 HDGF HDAC1 RBPJ TAF7 ZNF48 ZHX1 MIER2 ATF4 TOE1 STAT1 GLIS1 ZBTB21 RLF NFIB LARP7 ZNF398 TFAP4 PRDM4 LCORL ZNF18 ZFP69B GATA4 RXRB CSDE1 NR2F6 ZKSCAN1 IKZF5 ELF3 ZFX KLF1 SP7 PHF20 L3MBTL2 KDM3A NFIA KLF5 GABPB1 JUN NFRKB TRIM28 ZNF366 ARID1B ZNF561 ARID4B BCL6B NFATC3 ZNF687 GMEB1 E2F4 KLF10 NCOA6 CREB1 YY2 ZNF324 MLX ARID3A CHD1 TBL1XR1 CTCF ZSCAN21 SP3 ZSCAN4 MIXL1 ZBTB8A ZNF660 ZNF280D DACH1 SP5 ZXDB KHSRP EGR2 TARDBP ZEB2 GTF2F1 HNRNPL XRCC5 TGIF2 ZNF629 NFKBIZ DDX20 MTA1 ZNF692 U2AF2 ZHX2 KDM4B CHD2 PATZ1 OSR2 RNF2 IKZF2 KAT8 IRF2 ZBTB26 MYNN TRIM22 GLIS2 ZBTB17 ATF7 SOX6 ZNF143 GABPA EED TFDP1 ZNF501 YBX3 MTA3 PHF8 U2AF1 CBX3 ZSCAN18 RUNX3 POLR2B KLF8 RELB FOXA3 TCF7 TSHZ1 LEF1 ZBTB20 USF1 ZNF121 ZSCAN29 ZNF777 ZNF302 EZH2 AHR ZBTB11 RBFOX2 AFF1 NR2F2 NR2C1 EP300 ZBTB25 ZFHX2 PML ZGPAT OVOL3 SKI TEAD1 EMSY SCRT2 SREBF2 ZNF444 ZNF335 MIER1 E2F1 BCLAF1 NR2F1 ERF MGA WT1 UBTF

PROMO: ZIC2 C/EBPalpha MYBAS1 ZESTE AGL3 CART-1 C-JUN MUEBP-C2 PAX-2A MYF-3 C-MYC AP-2alpha YY1 CRX UBX DBP ZF5 MNB1A ZEN-1 PAX-8 P53 SP1 MYOD USF-1 E2F FOXP3 TMF ISL-1 NF-AT2 DSXF MF3 MZF-1 ZEN-2 ANTP E2F-1 STAT1beta POU1F1A OCSBF-1 HNF-3 GAGA POU4F NCX NF-AT1 DSXM PAX-6 CREMTAU GA-BF C/EBPbeta PAX-9B CACBP R2 NF-1 ELK-1 C/EBPdelta LIM1 MSX-1 VDR CREMTAU2 LVC COE1 RC2 MAFG BTEB4 PEA3 PBF MYB2 AP-3 STAT5A ZIC1 HB STAT4 P300 ABI4 C/EBP C-MYC HBP-1A NF-1 EN-1 DEF FACB ZIC3 UNC86 C-ETS-1 PAX-44 HES-1 E2 EIIAE-A N-MYC NF-AT1

JASPAR: Alx1 ALX3 Alx4 AR ARGFX Arid3a Arid3b Arid5a Arnt ARNT2 ARNT::HIF1A Arntl Arx ASCL1 Ascl2 Atf1 ATF2 Atf3 ATF3 ATF4 ATF6 ATF7 Atoh1 ATOH7 BACH1 Bach1::Mafk BACH2 BARHL1 BARHL2 BARX1 BARX2 BATF BATF3 BATF::JUN Bcl11B BCL6 BCL6B Bhlha15 BHLHA15 BHLHE22 BHLHE23 BHLHE40 BHLHE41 BNC2 BSX CDX1 CDX2 CDX4 CEBPA CEBPB CEBPD CEBPE CEBPG CLOCK CREB1 CREB3 CREB3L1 Creb3l2 CREB3L4 Creb5 CREM Crx CTCF CTCFL CUX1 CUX2 DBP Ddit3::Cebpa DLX1 Dlx2 Dlx3 Dlx4 Dlx5 DLX6 Dmbx1 Dmrt1 DMRT3 DMRTA1 DMRTA2 DMRTC2 DPRX DRGX Dux DUX4 DUXA E2F1 E2F2 E2F3 E2F4 E2F6 E2F7 E2F8 EBF1 Ebf2 EBF3 EGR1 EGR2 EGR3 EGR4 EHF ELF1 ELF2 ELF3 ELF4 Elf5 ELK1 ELK1::HOXA1 ELK1::HOXB13 ELK1::SREBF2 ELK3 ELK4 EMX1 EMX2 EN1 EN2 EOMES ERF ERF::FIGLA ERF::FOXI1 ERF::FOXO1 ERF::HOXB13 ERF::NHLH1 ERF::SREBF2 Erg ESR1 ESR2 ESRRA ESRRB Esrrg ESX1 ETS1 ETS2 ETV1 ETV2 ETV2::DRGX ETV2::FIGLA ETV2::FOXI1 ETV2::HOXB13 ETV3 ETV4 ETV5 ETV5::DRGX ETV5::FIGLA ETV5::FOXI1 ETV5::FOXO1 ETV5::HOXA2 ETV6 ETV7 EVX1 EVX2 EWSR1-FLI1 FERD3L FEV FIGLA FLI1 FLI1::DRGX FLI1::FOXI1 FOS FOSB::JUN FOSB::JUNB FOS::JUN FOS::JUNB FOS::JUND FOSL1 FOSL1::JUN FOSL1::JUNB FOSL1::JUND FOSL2 FOSL2::JUN FOSL2::JUNB FOSL2::JUND FOXA1 FOXA2 FOXA3 FOXB1 FOXC1 FOXC2 FOXD1 FOXD2 FOXD3 FOXE1 Foxf1 FOXF2 FOXG1 FOXH1 FOXI1 Foxj2 FOXJ2::ELF1 Foxj3 FOXK1 FOXK2 FOXL1 Foxl2 Foxn1 FOXN3 Foxo1 FOXO1::ELF1 FOXO1::ELK1 FOXO1::ELK3 FOXO1::FLI1 Foxo3 FOXO4 FOXO6 FOXP1 FOXP2 FOXP3 Foxq1 GABPA GATA1 GATA1::TAL1 GATA2 Gata3 GATA4 GATA5 GATA6 GBX1 GBX2 GCM1 GCM2 GFI1 Gfi1B Gli1 Gli2 GLI3 GLIS1 GLIS2 GLIS3 Gmeb1 GMEB2 GRHL1 GRHL2 GSC GSC2 GSX1 GSX2 Hand1::Tcf3 HAND2 HES1 HES2 HES5 HES6 HES7 HESX1 HEY1 HEY2 Hic1 HIC2 HIF1A HINFP HLF HMBOX1 Hmx1 Hmx2 Hmx3 Hnf1A HNF1A HNF1B HNF4A HNF4G HOXA1 HOXA10 Hoxa11 Hoxa13 HOXA2 HOXA4 HOXA5 HOXA6 HOXA7 HOXA9 HOXB13 HOXB2 HOXB2::ELK1 HOXB3 HOXB4 HOXB5 HOXB6 HOXB7 HOXB8 HOXB9 HOXC10 HOXC11 HOXC12 HOXC13 HOXC4 HOXC8 HOXC9 HOXD10 HOXD11 HOXD12 HOXD12::ELK1 Hoxd13 HOXD3 HOXD4 HOXD8 HOXD9 HSF1 HSF2 HSF4 IKZF1 Ikzf3 INSM1 Irf1 IRF2 IRF3 IRF4 IRF5 IRF6 IRF7 IRF8 IRF9 Isl1 ISL2 ISX JDP2 Jun JUN JUNB JUND JUN::JUNB KLF1 KLF10 KLF11 KLF12 KLF13 KLF14 KLF15 KLF16 KLF17 KLF2 KLF3 KLF4 KLF5 KLF6 KLF7 KLF9 LBX1 LBX2 Lef1 Lhx1 LHX2 Lhx3 Lhx4 LHX5 LHX6 Lhx8 LHX9 LIN54 LMX1A LMX1B MAF MAFA Mafb MAFF Mafg MAFG::NFE2L1 MAFK MAF::NFE2 MAX MAX::MYC MAZ Mecom MEF2A MEF2B MEF2C MEF2D MEIS1 MEIS2 MEIS3 MEOX1 MEOX2 MGA MGA::EVX1 MITF mix-a MIXL1 MLX Mlxip MLXIPL MNT MNX1 MSANTD3 MSC Msgn1 MSX1 MSX2 Msx3 MTF1 MXI1 MYB MYBL1 MYBL2 MYC MYCN MYF5 MYF6 MYOD1 MYOG MZF1 NEUROD1 Neurod2 NEUROG1 NEUROG2 Nfat5 Nfatc1 Nfatc2 NFATC3 NFATC4 NFE2 Nfe2l2 NFIA NFIB NFIC NFIC::TLX1 NFIL3 NFIX NFKB1 NFKB2 NFYA NFYB NFYC NHLH1 NHLH2 Nkx2-1 NKX2-2 NKX2-3 NKX2-4 NKX2-5 NKX2-8 Nkx3-1 Nkx3-2 NKX6-1 NKX6-2 NKX6-3 Nobox NOTO Npas2 Npas4 NR1D1 NR1D2 Nr1H2 NR1H2::RXRA Nr1h3::Rxra Nr1H4 NR1H4::RXRA NR1I2 NR1I3 NR2C1 NR2C2 Nr2e1 Nr2e3 NR2F1 NR2F2 Nr2f6 Nr2F6 NR2F6 NR3C1 NR3C2 NR4A1 NR4A2 NR4A2::RXRA NR5A1 Nr5A2 NR6A1 Nrf1 NRL OLIG1 Olig2 OLIG2 OLIG3 ONECUT1 ONECUT2 ONECUT3 OSR1 OSR2 OTX1 OTX2 OVOL1 OVOL2 PATZ1 PAX1 PAX2 PAX3 PAX4 PAX5 PAX6 Pax7 PAX9 PBX1 PBX2 PBX3 PDX1 PHOX2A PHOX2B PITX1 PITX2 PITX3 PKNOX1 PKNOX2 PLAG1 Plagl1 PLAGL2 POU1F1 POU2F1 POU2F1::SOX2 POU2F2 POU2F3 POU3F1 POU3F2 POU3F3 POU3F4 POU4F1 POU4F2 POU4F3 POU5F1 POU5F1B Pou5f1::Sox2 POU6F1 POU6F2 PPARA::RXRA PPARD PPARG Pparg::Rxra PRDM1 Prdm14 Prdm15 Prdm4 Prdm5 PRDM9 PROP1 PROX1 PRRX1 PRRX2 Ptf1a Ptf1A RARA RARA::RXRA RARA::RXRG Rarb RARB Rarg RARG RAX RAX2 RBPJ Rbpjl REL RELA RELB REST RFX1 RFX2 RFX3 RFX4 RFX5 Rfx6 RFX7 Rhox11 RHOXF1 RORA RORB RORC RREB1 Runx1 RUNX2 RUNX3 Rxra RXRA::VDR RXRB RXRG SATB1 SCRT1 SCRT2 SHOX Shox2 SIX1 SIX2 Six3 Six4 SMAD2 SMAD3 Smad4 SMAD5 SNAI1 SNAI2 SNAI3 SOHLH2 Sox1 SOX10 Sox11 SOX12 SOX13 SOX14 SOX15 Sox17 SOX18 SOX2 SOX21 Sox3 SOX4 Sox5 Sox6 SOX8 SOX9 SP1 SP2 SP3 SP4 SP5 SP8 SP9 SPDEF Spi1 SPIB SPIC Spz1 SREBF1 SREBF2 SRF SRY STAT1 STAT1::STAT2 Stat2 STAT3 Stat4 Stat5a Stat5a::Stat5b Stat5b Stat6 TAL1::TCF3 TBP TBR1 TBX1 TBX15 TBX18 TBX19 TBX2 TBX20 TBX21 TBX3 TBX4 TBX5 Tbx6 TBXT Tcf12 TCF12 Tcf21 TCF21 TCF3 TCF4 TCF7 TCF7L1 TCF7L2 TCFL5 TEAD1 TEAD2 TEAD3 TEAD4 TEF TFAP2A TFAP2B TFAP2C TFAP2E TFAP4 TFAP4::ETV1 TFAP4::FLI1 TFCP2 Tfcp2l1 TFDP1 TFE3 TFEB TFEC TGIF1 TGIF2 TGIF2LX TGIF2LY THAP1 Thap11 THRA THRB TLX2 TP53 TP63 TP73 TRPS1 TWIST1 Twist2 UNCX USF1 USF2 VAX1 VAX2 Vdr VENTX VEZF1 VSX1 VSX2 Wt1 XBP1 Yy1 YY2 ZBED1 ZBED2 ZBTB12 ZBTB14 ZBTB18 ZBTB26 ZBTB32 ZBTB33 ZBTB6 ZBTB7A ZBTB7B ZBTB7C ZEB1 ZFP14 Zfp335 ZFP42 ZFP57 Zfx ZIC1 Zic1::Zic2 Zic2 Zic3 ZIC4 ZIC5 ZIM3 ZKSCAN1 ZKSCAN3 ZKSCAN5 ZNF135 ZNF136 ZNF140 ZNF143 ZNF148 ZNF16 ZNF189 ZNF211 ZNF214 ZNF24 ZNF257 ZNF263 ZNF274 ZNF281 ZNF282 ZNF317 ZNF320 ZNF324 ZNF331 ZNF341 ZNF343 ZNF354A ZNF354C ZNF382 ZNF384 ZNF410 ZNF416 ZNF417 ZNF418 Znf423 ZNF449 ZNF454 ZNF460 ZNF528 ZNF530 ZNF549 ZNF574 ZNF582 ZNF610 ZNF652 ZNF667 ZNF669 ZNF675 ZNF680 ZNF682 ZNF684 ZNF692 ZNF701 ZNF707 ZNF708 ZNF740 ZNF75D ZNF76 ZNF768 ZNF784 ZNF8 ZNF816 ZNF85 ZNF93 ZSCAN29 ZSCAN31 ZSCAN4

Table7 Genes differentially expressed in both mRNA and m7G

| **gene** | **Fold change (mRNA)** | **p-value** | **Fold change (m7G)** |
| --- | --- | --- | --- |
| CTD-2583A14.10 | 5.3664573 | 0.03925 | -3 |
| RELN | 4.0183689 | 0.00095 | -3.304347826 |
| SESN2 | 3.1777156 | 0.00005 | -2.783783784 |
| AP5Z1 | 3.1619619 | 0.0008 | -2.666666667 |
| COLCA1 | 2.8868777 | 0.00005 | 4.166666667 |
| ACTA2 | 2.8743386 | 0.00815 | -3 |
| CDKN1A | 2.793725 | 0.00005 | 2.082530797 |
| GPNMB | 2.7926988 | 0.00005 | -3 |
| SIRT6 | 2.7062404 | 0.00005 | -2.585897436 |
| ICA1L | 2.5481673 | 0.0013 | 3 |
| CTH | 2.4770964 | 0.00005 | -3 |
| BMP7 | 2.4564247 | 0.0001 | 2.563433714 |
| ID1 | 2.4539571 | 0.00005 | -2.441383688 |
| LSS | 2.4000093 | 0.00005 | 3.361111111 |
| ATP2C2 | 2.379684 | 0.00005 | -3 |
| ROM1 | 2.3288537 | 0.0099 | 2.995098039 |
| C5orf28 | 2.268392 | 0.0012 | 2.489191919 |
| TBPL1 | 2.2426741 | 0.00525 | -2.80952381 |
| GLRX2 | 2.2289743 | 0.0001 | -2.833333333 |
| WARS | 2.2132248 | 0.00005 | -88.91208791 |
| AEN | 2.21301 | 0.00005 | 6 |
| HMGCS1 | 2.2048492 | 0.00005 | -2.160852559 |
| SURF2 | 2.1927936 | 0.00155 | -3 |
| TXNIP | 2.1900898 | 0.00005 | -3 |
| TMEM14A | 2.125865 | 0.0094 | -2.567375887 |
| THAP8 | 2.1017776 | 0.01305 | -2.16547619 |
| MAPK14 | 2.066329 | 0.0036 | -6.143116085 |
| PSAT1 | 2.0638527 | 0.00005 | -52.11396993 |
| MSMO1 | 2.0630231 | 0.00005 | -242.5102273 |
| ZNF419 | 2.0310419 | 0.00015 | -2.666666667 |
| ADM2 | 1.9993652 | 0.00005 | 2.833333333 |
| TSEN54 | 1.9921869 | 0.01175 | -2.666666667 |
| NRF1 | 1.9871338 | 0.0023 | -3 |
| GDF15 | 1.939175 | 0.00005 | -13.60824525 |
| FAM73B | 1.9361007 | 0.00205 | -2.870173659 |
| TRPV6 | 1.9318204 | 0.00005 | -2.470402954 |
| ABTB2 | 1.9124911 | 0.0009 | -5 |
| CASP3 | 1.8931609 | 0.00015 | -5.142857143 |
| ATP6AP1 | 1.8564069 | 0.00005 | -2.611819806 |
| IGF2R | 1.8483065 | 0.00015 | -3 |
| GRB10 | 1.8004893 | 0.00085 | -5 |
| CDIP1 | 1.7956946 | 0.0002 | -2.682692308 |
| DGKQ | 1.7873696 | 0.02245 | -31.87795842 |
| SIGIRR | 1.7861831 | 0.0273 | -3 |
| YRDC | 1.7856818 | 0.00465 | -3 |
| SLC7A11 | 1.7805538 | 0.00005 | -48.44444444 |
| DSEL | 1.7789488 | 0.00005 | -2.361111111 |
| DDC | 1.7780785 | 0.0398 | 3 |
| CHID1 | 1.7728863 | 0.00045 | -2.594589198 |
| HSF1 | 1.7586922 | 0.00005 | -3 |
| TSC22D3 | 1.7535858 | 0.00175 | -2.049218998 |
| TECPR2 | 1.7513252 | 0.0429 | -3 |
| TMEM101 | 1.7505254 | 0.0115 | -2.962962963 |
| PHLDA3 | 1.7477369 | 0.00765 | 2.642392392 |
| DHRS7 | 1.7453822 | 0.00005 | -3 |
| PLXNA1 | 1.7438416 | 0.0084 | -3 |
| ANKRD13D | 1.7414572 | 0.0049 | 3 |
| RABEP2 | 1.7295503 | 0.0113 | -257.233539 |
| XBP1 | 1.7293501 | 0.00005 | -2.094847021 |
| NBPF1 | 1.7260365 | 0.0031 | 2.051374418 |
| ASNS | 1.7238509 | 0.0001 | -2.904188948 |
| PKIB | 1.7190053 | 0.00005 | -2.423736969 |
| SNX17 | 1.705518 | 0.0111 | -2.592592593 |
| RNF187 | 1.7045005 | 0.0001 | 4.620889709 |
| NSDHL | 1.7043741 | 0.0004 | -3.881481481 |
| HERPUD1 | 1.7033017 | 0.0002 | 2.143102517 |
| UNC50 | 1.6983031 | 0.01695 | -3 |
| GARS | 1.6926105 | 0.0169 | 2.335978836 |
| DOCK3 | 1.6887971 | 0.00015 | -2.960784314 |
| ATP2B1 | 1.6869603 | 0.00395 | 2.508805031 |
| TMEM184B | 1.6813464 | 0.0304 | -6.82302121 |
| TOLLIP | 1.6813289 | 0.00405 | -6 |
| KCTD10 | 1.6771433 | 0.01315 | -2.294498098 |
| ATP6V0B | 1.6686715 | 0.00055 | -2.504658385 |
| LRRC47 | 1.6665713 | 0.0011 | -2.111111111 |
| SRD5A3 | 1.6515504 | 0.00675 | -2.666666667 |
| ELOF1 | 1.6498101 | 0.00265 | 2.354747475 |
| LSM10 | 1.6467049 | 0.0089 | -2.120645429 |
| ODC1 | 1.6392149 | 0.00005 | -5.633288697 |
| PYCR2 | 1.6376136 | 0.0001 | 3 |
| CRTAP | 1.6375126 | 0.00425 | -2.487179487 |
| MLST8 | 1.6269535 | 0.00015 | 2.017316962 |
| SRM | 1.6185389 | 0.00005 | 2.252173913 |
| USP3 | 1.6147278 | 0.0015 | -3 |
| POLB | 1.6056807 | 0.01675 | -2.5 |
| GABARAPL1 | 1.6039921 | 0.00275 | -2.528326023 |
| SLC39A11 | 1.5950736 | 0.0131 | -15.89366824 |
| KCTD15 | 1.5942479 | 0.0204 | -3 |
| ECI1 | 1.5915969 | 0.0011 | -175.4333273 |
| SQLE | 1.5904941 | 0.00005 | -32.07411846 |
| HID1 | 1.5888765 | 0.0003 | -2.685185185 |
| NIPAL1 | 1.5873915 | 0.0235 | 2.666666667 |
| ZMAT3 | 1.5865423 | 0.00015 | 2.666666667 |
| DRG2 | 1.5837307 | 0.0025 | -6 |
| SLC3A2 | 1.5836593 | 0.00095 | -2.084158354 |
| EMC10 | 1.5781048 | 0.0009 | 2.27369761 |
| MED19 | 1.5751629 | 0.00645 | -2.659259259 |
| SLCO5A1 | 1.5723997 | 0.03615 | -2.833333333 |
| SEC61A1 | 1.5719464 | 0.0004 | -2.583333333 |
| EPN3 | 1.5716097 | 0.0336 | 2.833333333 |
| SHANK2 | 1.5699112 | 0.0251 | -2.791666667 |
| IMPDH1 | 1.5685451 | 0.00735 | 3 |
| PRR12 | 1.5653215 | 0.00265 | -2.761904762 |
| JTB | 1.5627977 | 0.01695 | -2.108884529 |
| ZNF335 | 1.5540458 | 0.0061 | -3 |
| TFE3 | 1.5537097 | 0.00455 | -2.675 |
| CCDC85A | 1.5525277 | 0.0367 | -3 |
| CXXC1 | 1.5506005 | 0.026 | -2.645467489 |
| TP53INP1 | 1.5496625 | 0.0005 | -3.666666667 |
| KLHL28 | 1.5475972 | 0.04925 | -2.555555556 |
| SLC1A5 | 1.5430203 | 0.0063 | -2.144531726 |
| MAGEA6 | 1.5389304 | 0.0001 | -2.560763889 |
| COA5 | 1.5353356 | 0.0334 | -2.200980392 |
| HYOU1 | 1.5325658 | 0.00075 | -2.396825397 |
| SLC7A5 | 1.5324702 | 0.00075 | -78.47415357 |
| DDX19A | 1.5299177 | 0.0022 | -2.638945233 |
| ID3 | 1.5270613 | 0.0001 | -3.159846547 |
| SARS | 1.5254533 | 0.00005 | -2.006151528 |
| LCOR | 1.5248021 | 0.0391 | -2.416750209 |
| SLC52A2 | 1.5241068 | 0.03385 | -4.464228911 |
| VGLL4 | 1.5231658 | 0.01545 | 2.444444444 |
| PPAP2A | 1.5210989 | 0.01255 | 3.600982724 |
| RPS27L | 1.5189064 | 0.0037 | -2.791666667 |
| LRRC8D | 1.5128008 | 0.03585 | -2.005503634 |
| B4GALT2 | 1.5103878 | 0.00555 | -2.926119079 |
| PCK2 | 1.5082902 | 0.0002 | -2.040515499 |
| AFAP1 | 1.507779 | 0.01395 | 2.666666667 |
| MNX1 | 1.5072701 | 0.03405 | -2.666666667 |
| XPO6 | 1.506327 | 0.028 | -2.666666667 |
| TARS | 1.5044269 | 0.0002 | -2.462962963 |
| PYURF | 1.5039723 | 0.026 | -2.674603175 |
| OOEP | -6.716874 | 0.02845 | 2.171882575 |
| FRY | -4.383156 | 0.0004 | -3 |
| SLITRK6 | -3.926789 | 0.00005 | -2.687119857 |
| SEMA6D | -3.636146 | 0.00005 | 5.625 |
| ZBTB7C | -3.451085 | 0.00005 | 2.5 |
| TFPI | -3.344499 | 0.00005 | -2.666666667 |
| CCDC148 | -3.234539 | 0.0005 | -3 |
| TLL1 | -2.830408 | 0.00005 | -2.444444444 |
| EFEMP2 | -2.779124 | 0.023 | 5 |
| FN1 | -2.625241 | 0.00005 | 7.461250966 |
| DQX1 | -2.52911 | 0.0218 | -3 |
| DAZAP2 | -2.498617 | 0.00005 | -15.58164708 |
| KANK1 | -2.477337 | 0.00865 | -3 |
| ACVR2B | -2.469707 | 0.00005 | -3 |
| FAM19A2 | -2.45428 | 0.04175 | -3 |
| PDZD2 | -2.43944 | 0.04625 | 2.666666667 |
| RBPMS | -2.415715 | 0.00005 | 2.5 |
| DLG2 | -2.36102 | 0.00065 | -2.131864623 |
| NFIB | -2.317742 | 0.00395 | -2.01375543 |
| CDK14 | -2.311389 | 0.01785 | -3 |
| VPS33B | -2.263696 | 0.0086 | -2.881481481 |
| AGK | -2.234776 | 0.0033 | -2.666666667 |
| METTL15 | -2.225084 | 0.01995 | -3 |
| TGFBR3 | -2.15454 | 0.0002 | -2.461988304 |
| TVP23C | -2.130674 | 0.0052 | -18.68322981 |
| KLK2 | -2.117218 | 0.00005 | -2.276136459 |
| PRIM2 | -2.115649 | 0.0007 | -3 |
| CNP | -2.115018 | 0.00715 | -5.5 |
| NHSL1 | -2.107847 | 0.04095 | -3 |
| NUDT11 | -2.057539 | 0.00205 | -2.75 |
| FRYL | -2.053365 | 0.00515 | -2.390144678 |
| MPP3 | -2.040284 | 0.00145 | 3 |
| IPMK | -2.039846 | 0.00005 | 3.582905983 |
| SESN3 | -2.039379 | 0.00005 | -3.120892172 |
| C18orf54 | -2.000485 | 0.04665 | -2.696969697 |
| PPA1 | -1.969762 | 0.00005 | -2.149833103 |
| CENPF | -1.95977 | 0.0084 | -3 |
| SEMA4A | -1.950285 | 0.00105 | -2.527777778 |
| CALD1 | -1.941891 | 0.00005 | -3.83279242 |
| LUC7L3 | -1.924949 | 0.00005 | -2.099386724 |
| ISPD | -1.917305 | 0.00005 | -2.8875 |
| HMGCS2 | -1.906032 | 0.0018 | -2.342815645 |
| MCM3 | -1.880891 | 0.00005 | -3 |
| ERCC4 | -1.847438 | 0.00715 | -3 |
| RSF1 | -1.843539 | 0.026 | -2.023188799 |
| SH3RF3 | -1.842495 | 0.005 | -3 |
| SPATA6 | -1.827785 | 0.00755 | -3 |
| ACOT9 | -1.810615 | 0.0462 | -2.200177798 |
| LIG1 | -1.80766 | 0.011 | 2.748756219 |
| NASP | -1.800704 | 0.00005 | -2.61965812 |
| ANP32E | -1.769937 | 0.00005 | -2.736111111 |
| RALBP1 | -1.738113 | 0.002 | 2.904314888 |
| FERMT2 | -1.734124 | 0.00535 | -5.032258065 |
| PBX1 | -1.728102 | 0.01345 | -3 |
| SAT1 | -1.727534 | 0.00035 | -124.4873768 |
| PIK3C3 | -1.726407 | 0.01955 | 2.460393318 |
| GPR126 | -1.723019 | 0.0101 | 26.50893418 |
| CEP63 | -1.720146 | 0.01225 | -2.611111111 |
| PRUNE2 | -1.713339 | 0.0011 | -2.111111111 |
| ANLN | -1.709086 | 0.00275 | -5.566685052 |
| STX2 | -1.708165 | 0.0226 | 4.796777297 |
| BDP1 | -1.707946 | 0.00005 | 3 |
| ANKRD24 | -1.702642 | 0.037 | -3 |
| LUZP2 | -1.698798 | 0.00115 | 2.319277359 |
| PNN | -1.69523 | 0.0153 | 2.652777778 |
| KCNH2 | -1.690586 | 0.00075 | 3 |
| CDC45 | -1.687942 | 0.00595 | -2.666666667 |
| STMN1 | -1.686892 | 0.00015 | -2.094014237 |
| KLHDC9 | -1.684415 | 0.0472 | -2.325358852 |
| ETV1 | -1.68437 | 0.0043 | -3 |
| TRMT6 | -1.684191 | 0.0116 | -3 |
| CCDC25 | -1.684104 | 0.04835 | -2.313297214 |
| KNTC1 | -1.679946 | 0.0125 | -3 |
| NUDT6 | -1.678737 | 0.02285 | -2.224786325 |
| SNRPD1 | -1.67485 | 0.00015 | -2.666666667 |
| ZNF367 | -1.667548 | 0.00005 | -2.916666667 |
| EXOSC9 | -1.655649 | 0.021 | -3 |
| DIAPH3 | -1.652793 | 0.00005 | -2.776360544 |
| DCP1A | -1.648354 | 0.0274 | -3 |
| RAB13 | -1.63079 | 0.00005 | -20.74529915 |
| TSPAN14 | -1.6296 | 0.0201 | -2.677419355 |
| HMGB2 | -1.627429 | 0.00005 | -5.321985816 |
| ANKRD34A | -1.625871 | 0.00615 | -2.554331129 |
| RUFY3 | -1.622002 | 0.0118 | -2.333333333 |
| RCN3 | -1.616912 | 0.0283 | -3 |
| ANP32B | -1.612282 | 0.00005 | -2.286223948 |
| SFPQ | -1.60957 | 0.00075 | -2.995726496 |
| FAM216A | -1.600841 | 0.0094 | -2.988888889 |
| BBS9 | -1.594972 | 0.0302 | 3 |
| LGI2 | -1.594011 | 0.0001 | -3 |
| PPP3CA | -1.593075 | 0.00915 | -3.571473343 |
| RFK | -1.590027 | 0.01375 | -3 |
| TM2D1 | -1.582968 | 0.03175 | -2.308791209 |
| PIGK | -1.580671 | 0.02425 | -2.296296296 |
| NES | -1.571001 | 0.00005 | -2.763366656 |
| FANCM | -1.567949 | 0.03145 | -3 |
| PRC1 | -1.563945 | 0.0166 | -2.560648148 |
| NDRG1 | -1.556818 | 0.02295 | -3.098039216 |
| RAD51AP1 | -1.541834 | 0.01675 | -4.875 |
| PTGR2 | -1.54021 | 0.0245 | -2.918881119 |
| TBCA | -1.530907 | 0.00005 | -3 |
| PRKAR1A | -1.524068 | 0.00395 | -2.077908689 |
| PTMA | -1.518376 | 0.00005 | -13.81276778 |
| NEK2 | -1.517545 | 0.00295 | -2.58974359 |
| ZNF644 | -1.514191 | 0.02695 | -3 |
| NAPG | -1.50924 | 0.0028 | -5 |
| CCDC34 | -1.500843 | 0.01175 | -2.992907801 |

Table8 KEGG Enrichment Results

| KEGG Term | ID | Input number | Background number | P-Value | Input gene | gene ratio |
| --- | --- | --- | --- | --- | --- | --- |
| Metabolic pathways | hsa01100 | 6 | 1433 | 0.144863 | SAT1\|HMGCS2\|AGK\|PIGK\|RFK\|ISPD | 0.061856 |
| Transcriptional misregulation in cancer | hsa05202 | 3 | 186 | 0.011563 | ETV1\|CDK14\|PBX1 | 0.030928 |
| DNA replication | hsa03030 | 2 | 36 | 0.003992 | MCM3\|PRIM2 | 0.020619 |
| Fanconi anemia pathway | hsa03460 | 2 | 54 | 0.008497 | FANCM\|ERCC4 | 0.020619 |
| RNA degradation | hsa03018 | 2 | 79 | 0.01718 | EXOSC9\|DCP1A | 0.020619 |
| Cell cycle | hsa04110 | 2 | 124 | 0.03889 | CDC45\|MCM3 | 0.020619 |
| Vascular smooth muscle contraction | hsa04270 | 2 | 132 | 0.043456 | CALD1\|CNP | 0.020619 |
| Systemic lupus erythematosus | hsa05322 | 2 | 133 | 0.04404 | HIST2H2AA4\|SNRPD1 | 0.020619 |
| Fluid shear stress and atherosclerosis | hsa05418 | 2 | 139 | 0.047606 | ACVR2B\|CNP | 0.020619 |
| RNA transport | hsa03013 | 2 | 165 | 0.06419 | SMN2\|PNN | 0.020619 |
| cGMP-PKG signaling pathway | hsa04022 | 2 | 167 | 0.065537 | PPP3CA\|CNP | 0.020619 |
| Axon guidance | hsa04360 | 2 | 181 | 0.075221 | SEMA4A\|PPP3CA | 0.020619 |
| Regulation of actin cytoskeleton | hsa04810 | 2 | 214 | 0.099651 | FN1\|DIAPH3 | 0.020619 |
| MAPK signaling pathway | hsa04010 | 2 | 295 | 0.166623 | PPP3CA\|STMN1 | 0.020619 |
| Pathways in cancer | hsa05200 | 2 | 530 | 0.378199 | RALBP1\|FN1 | 0.020619 |
| Riboflavin metabolism | hsa00740 | 1 | 8 | 0.021975 | RFK | 0.010309 |
| Synthesis and degradation of ketone bodies | hsa00072 | 1 | 10 | 0.026793 | HMGCS2 | 0.010309 |
| Terpenoid backbone biosynthesis | hsa00900 | 1 | 22 | 0.055212 | HMGCS2 | 0.010309 |
| Mannose type O-glycan biosynthesis | hsa00515 | 1 | 23 | 0.057543 | ISPD | 0.010309 |
| Renin-angiotensin system | hsa04614 | 1 | 23 | 0.057543 | KLK2 | 0.010309 |
| Glycosylphosphatidylinositol (GPI)-anchor biosynthesis | hsa00563 | 1 | 25 | 0.062188 | PIGK | 0.010309 |
| Butanoate metabolism | hsa00650 | 1 | 28 | 0.069112 | HMGCS2 | 0.010309 |
| Pentose and glucuronate interconversions | hsa00040 | 1 | 34 | 0.08281 | ISPD | 0.010309 |
| Ferroptosis | hsa04216 | 1 | 40 | 0.096308 | SAT1 | 0.010309 |
| Nucleotide excision repair | hsa03420 | 1 | 47 | 0.111808 | ERCC4 | 0.010309 |
| Valine, leucine and isoleucine degradation | hsa00280 | 1 | 48 | 0.114 | HMGCS2 | 0.010309 |
| Endocrine and other factor-regulated calcium reabsorption | hsa04961 | 1 | 50 | 0.118369 | KLK2 | 0.010309 |
| Arginine and proline metabolism | hsa00330 | 1 | 50 | 0.118369 | SAT1 | 0.010309 |
| Amyotrophic lateral sclerosis (ALS) | hsa05014 | 1 | 51 | 0.120546 | PPP3CA | 0.010309 |
| VEGF signaling pathway | hsa04370 | 1 | 59 | 0.137768 | PPP3CA | 0.010309 |
| Glycerolipid metabolism | hsa00561 | 1 | 61 | 0.142021 | AGK | 0.010309 |
| Cortisol synthesis and secretion | hsa04927 | 1 | 65 | 0.150466 | PBX1 | 0.010309 |
| Long-term potentiation | hsa04720 | 1 | 67 | 0.154657 | PPP3CA | 0.010309 |
| Amphetamine addiction | hsa05031 | 1 | 68 | 0.156745 | PPP3CA | 0.010309 |
| Renin secretion | hsa04924 | 1 | 69 | 0.158827 | PPP3CA | 0.010309 |
| p53 signaling pathway | hsa04115 | 1 | 72 | 0.165045 | SESN3 | 0.010309 |
| Bacterial invasion of epithelial cells | hsa05100 | 1 | 74 | 0.169165 | FN1 | 0.010309 |
| Pancreatic cancer | hsa05212 | 1 | 75 | 0.171218 | RALBP1 | 0.010309 |
| PPAR signaling pathway | hsa03320 | 1 | 76 | 0.173265 | HMGCS2 | 0.010309 |
| Complement and coagulation cascades | hsa04610 | 1 | 79 | 0.179377 | TFPI | 0.010309 |
| B cell receptor signaling pathway | hsa04662 | 1 | 82 | 0.185445 | PPP3CA | 0.010309 |
| ECM-receptor interaction | hsa04512 | 1 | 86 | 0.193466 | FN1 | 0.010309 |
| PD-L1 expression and PD-1 checkpoint pathway in cancer | hsa05235 | 1 | 89 | 0.19943 | PPP3CA | 0.010309 |
| Longevity regulating pathway | hsa04211 | 1 | 89 | 0.19943 | SESN3 | 0.010309 |
| mRNA surveillance pathway | hsa03015 | 1 | 91 | 0.203383 | PNN | 0.010309 |
| Th1 and Th2 cell differentiation | hsa04658 | 1 | 92 | 0.205351 | PPP3CA | 0.010309 |
| Small cell lung cancer | hsa05222 | 1 | 93 | 0.207315 | FN1 | 0.010309 |
| TGF-beta signaling pathway | hsa04350 | 1 | 94 | 0.209274 | ACVR2B | 0.010309 |
| Amoebiasis | hsa05146 | 1 | 95 | 0.211229 | FN1 | 0.010309 |
| AGE-RAGE signaling pathway in diabetic complications | hsa04933 | 1 | 100 | 0.220929 | FN1 | 0.010309 |
| T cell receptor signaling pathway | hsa04660 | 1 | 103 | 0.226692 | PPP3CA | 0.010309 |
| C-type lectin receptor signaling pathway | hsa04625 | 1 | 104 | 0.228604 | PPP3CA | 0.010309 |
| Glucagon signaling pathway | hsa04922 | 1 | 106 | 0.232414 | PPP3CA | 0.010309 |
| Th17 cell differentiation | hsa04659 | 1 | 107 | 0.234311 | PPP3CA | 0.010309 |
| Glutamatergic synapse | hsa04724 | 1 | 114 | 0.247466 | PPP3CA | 0.010309 |
| Yersinia infection | hsa05135 | 1 | 121 | 0.260397 | FN1 | 0.010309 |
| Osteoclast differentiation | hsa04380 | 1 | 128 | 0.273108 | PPP3CA | 0.010309 |
| Oocyte meiosis | hsa04114 | 1 | 128 | 0.273108 | PPP3CA | 0.010309 |
| Dopaminergic synapse | hsa04728 | 1 | 131 | 0.278489 | PPP3CA | 0.010309 |
| Natural killer cell mediated cytotoxicity | hsa04650 | 1 | 131 | 0.278489 | PPP3CA | 0.010309 |
| Oxidative phosphorylation | hsa00190 | 1 | 133 | 0.282055 | PPA1 | 0.010309 |
| Spliceosome | hsa03040 | 1 | 135 | 0.285603 | SNRPD1 | 0.010309 |
| Insulin signaling pathway | hsa04910 | 1 | 137 | 0.289134 | PRKAR1A | 0.010309 |
| Signaling pathways regulating pluripotency of stem cells | hsa04550 | 1 | 140 | 0.294398 | ACVR2B | 0.010309 |
| Oxytocin signaling pathway | hsa04921 | 1 | 153 | 0.316765 | PPP3CA | 0.010309 |
| Cushing syndrome | hsa04934 | 1 | 155 | 0.320143 | PBX1 | 0.010309 |
| Wnt signaling pathway | hsa04310 | 1 | 160 | 0.328517 | PPP3CA | 0.010309 |
| Cellular senescence | hsa04218 | 1 | 160 | 0.328517 | PPP3CA | 0.010309 |
| Necroptosis | hsa04217 | 1 | 162 | 0.331838 | HIST2H2AA4 | 0.010309 |
| Tight junction | hsa04530 | 1 | 170 | 0.344959 | RAB13 | 0.010309 |
| Alzheimer disease | hsa05010 | 1 | 171 | 0.346581 | PPP3CA | 0.010309 |
| Tuberculosis | hsa05152 | 1 | 179 | 0.359416 | PPP3CA | 0.010309 |
| Alcoholism | hsa05034 | 1 | 180 | 0.361003 | HIST2H2AA4 | 0.010309 |
| Kaposi sarcoma-associated herpesvirus infection | hsa05167 | 1 | 186 | 0.370442 | PPP3CA | 0.010309 |
| Calcium signaling pathway | hsa04020 | 1 | 193 | 0.381279 | PPP3CA | 0.010309 |
| Focal adhesion | hsa04510 | 1 | 199 | 0.390422 | FN1 | 0.010309 |
| Proteoglycans in cancer | hsa05205 | 1 | 203 | 0.396442 | FN1 | 0.010309 |
| Human immunodeficiency virus 1 infection | hsa05170 | 1 | 212 | 0.409774 | PPP3CA | 0.010309 |
| Human T-cell leukemia virus 1 infection | hsa05166 | 1 | 219 | 0.419941 | PPP3CA | 0.010309 |
| Human cytomegalovirus infection | hsa05163 | 1 | 225 | 0.428518 | PPP3CA | 0.010309 |
| Ras signaling pathway | hsa04014 | 1 | 232 | 0.438365 | RALBP1 | 0.010309 |
| Cytokine-cytokine receptor interaction | hsa04060 | 1 | 294 | 0.51857 | ACVR2B | 0.010309 |
| MicroRNAs in cancer | hsa05206 | 1 | 299 | 0.524521 | STMN1 | 0.010309 |
| Human papillomavirus infection | hsa05165 | 1 | 330 | 0.559823 | FN1 | 0.010309 |
| PI3K-Akt signaling pathway | hsa04151 | 1 | 354 | 0.585361 | FN1 | 0.010309 |

METTL1 Mutant Sites

NM_005371(E107/I108/R109/N140/T238/E239/E240A)

ATGGCAGCCGAGACTCGGAACGTGGCCGGAGCAGAGGCCCCACCGCCCCAGAAGCGCTACTACCGGCAACGTGCTCACTCCAACCCCATGGCGGACCACACGCTGCGCTACCCTGTGAAGCCAGAGGAGATGGACTGGTCTGAGCTATACCCAGAGTTCTTCGCTCCACTCACTCAAAATCAGAGCCACGATGACCCAAAGGATAAGAAAGAAAAGAGAGCTCAGGCCCAAGTGGAGTTTGCAGACATAGGCTGTGGCTATGGTGGCCTGTTAGTGGAACTGTCACCGCTGTTCCCAGACACACTTATTCTGGGTCTGGCGGCCGCGGTGAAGGTCTCAGACTATGTACAAGACCGGATTCGGGCCCTACGCGCAGCTCCTGCAGGTGGCTTCCAGAACATCGCCTGTCTCCGTAGCGCTGCCATGAAGCACCTTCCTAACTTCTTCTACAAGGGCCAGCTGACAAAGATGTTCTTCCTCTTCCCCGACCCACATTTCAAGCGGACAAAGCACAAGTGGCGAATCATCAGTCCCACCCTGCTAGCAGAATATGCCTACGTGCTAAGAGTTGGGGGGCTGGTGTATACCATAACCGATGTGCTGGAGCTACACGACTGGATGTGCACTCATTTCGAAGAGCACCCACTGTTTGAGCGTGTGCCTCTGGAGGACCTGAGTGAAGACCCCGTTGTGGGACATCTAGGCACCTCAGCTGCGGCGGGGAAGAAAGTTCTACGTAATGGAGGGAAGAATTTCCCAGCCATCTTCCGAAGAATACAAGATCCCGTCCTCCAGGCAGTGACCTCCCAAACCAGCCTGCCTGGTCACTGA
